# On-DNA Synthesis of Multisubstituted Indoles

**DOI:** 10.1021/acs.orglett.3c03602

**Published:** 2023-12-18

**Authors:** András Gy. Németh, Levente Kollár, Krisztina Németh, Gitta Schlosser, Annamária Minus, György M. Keserű

**Affiliations:** ‡ Medicinal Chemistry Research Group, HUN-REN Research Centre for Natural Sciences, H-1117 Budapest, Hungary; § Department of Organic Chemistry and Technology, Budapest University of Technology and Economics, H-1111 Budapest, Hungary; ∥ National Laboratory for Drug Research and Development, H-1117 Budapest, Hungary; ⊥ Centre for Structure Study, HUN-REN Research Centre for Natural Sciences, H-1117 Budapest, Hungary; # MTA-ELTE Lendület Ion Mobility Mass Spectrometry Research Group, Eötvös University, Budapest H-1117, Hungary; ∇ Institute of Enzymology, HUN-REN Research Centre for Natural Sciences, H-1117 Budapest, Hungary

## Abstract

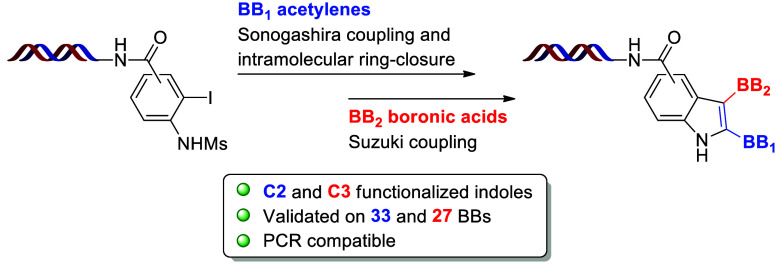

The increasing role of the DNA-encoded library technology in early phase drug discovery represents a significant demand for DNA-compatible synthetic methods for therapeutically relevant heterocycles. Herein, we report the first on-DNA synthesis of multisubstituted indoles via a cascade reaction of Sonogashira coupling and intramolecular ring closure. Further functionalization by Suzuki coupling at the third position exploits a diverse chemical space. The high fidelity of the method also enabled the construction of an indole-based mock library.

DNA-encoded library (DEL) technology is now an important hit discovery tool that covers an unprecedentedly large part of the bioactive chemical space through the effective screening of billions of molecules. Since its inception 3 decades ago, the pool of DNA-compatible chemical transformations has been significantly extended, facilitating the on-DNA synthesis of numerous heterocycles.^1−4^ The indole scaffold is a privileged structure as a result of its appearance in many natural products, synthetic ligands, and drugs.^5−9^ On the basis of the substitutional pattern of U.S. Food and Drug Administration (FDA)-approved drugs, synthetic options for functionalization at the second and third positions are preferred.^7^ Lately, a handful of on-DNA methods were reported for the selenylation, sulfenylation, and sulfonylation of indoles at the second and third positions.^10−13^ Also, multistep condensation with aldehydes and indole–styrene couplings or cross-dehydrogenative couplings at the second and third positions may lead to alkylated derivatives.^14−17^ Despite the growing interest in indole-containing DELs, no method for on-DNA indole synthesis has been reported thus far.^18−20^

On the basis of the handbook of DEL, traditional methods, such as Fischer indole synthesis, applying high temperatures and strongly acidic or inert conditions, are either not feasible on-DNA or propose detrimental reaction conditions to DNA.^21,22^ Recently, Kazmierski et al. reported potent IDO1 inhibitors identified by indole-based DEL screening (Scheme 1a).^23^ Although the strategy of applying Larock indole synthesis has been revealed, no experimental details were disclosed. In contrast to the reported method, we considered an alternative synthetic approach that would not hinder the pharmaceutically essential third position of indole but would exploit the less important sixth position for DNA attachment (Scheme 1b). Inspired by the work of Sakamoto et al. on the off-DNA cyclization of 2-ethynyl sulfonamides^24^ and the reports of Satz et al. and Neri et al. on the on-DNA Sonogashira couplings,^25,26^ we envisioned a cascade reaction starting from 2-iodo sulfonamides to indoles in one step. Subsequent iodination at the third position of indole would enable further functionalization by Suzuki couplings. This approach would also enable the introduction of amino acids at the fifth or sixth position of indole, offering another position for variability.

**Scheme 1:**
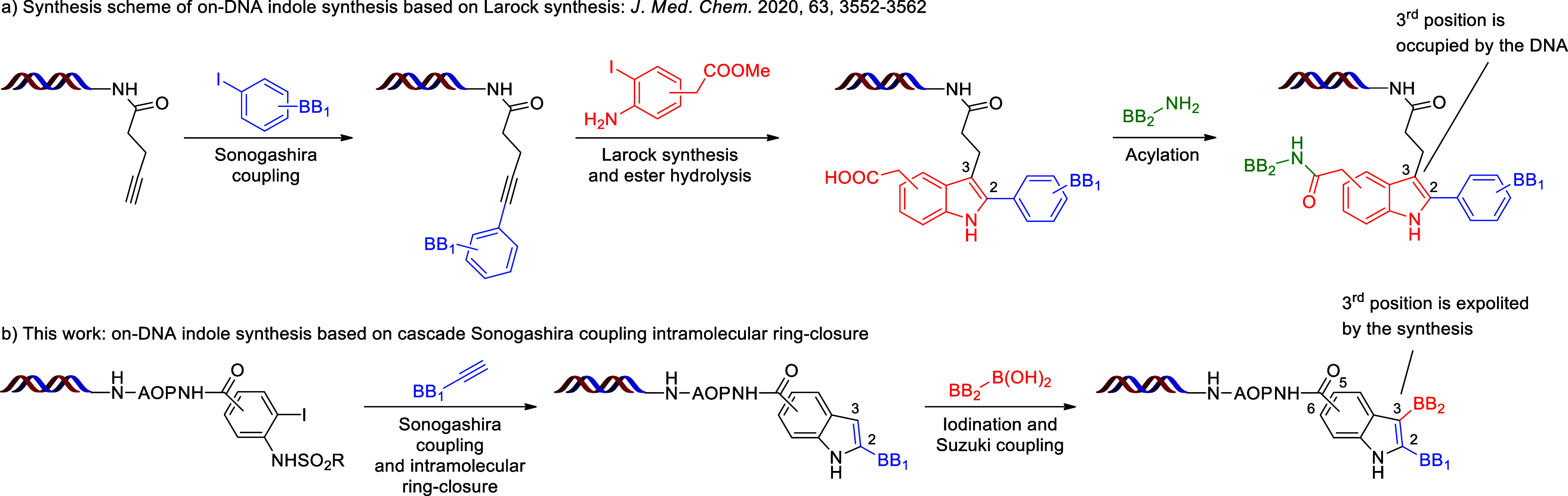
Strategy for the On-DNA Synthesis of Multisubstituted Indoles

Initially, we investigated the cascade Sonogashira coupling and intramolecular ring closure using DNA-conjugated *ortho*-iodo *N*-methanesulfonamide (**1a**) and phenylacetylene (**2**). Starting from recently published DNA-compatible Sonogashira couplings, we first applied a Pd(OAc)_2_/TPPTS precatalyst system in the presence of CuSO_4_, sodium ascorbate, and K_2_CO_3_ using DMAc as the organic co-solvent at 75 °C for 3 h (entry 1 in Table 1). These conditions enabled full conversion respective to the starting material, resulting in a 9% yield of the desired indole **3a** and 78% yield of the intermediates **4** and **5** based on high-performance liquid chromatography–mass spectrometry (HPLC–MS) (see the Supporting Information). Further experiments revealed that only dimethyl sulfoxide (DMSO) was a suitable co-solvent promoting not only the Sonogashira coupling but also the sequential ring closure, resulting in a 73% yield of product **3a** (entry 2 in Table 1). Other co-solvents, such as MeCN and *N*,*N*-dimethylformamide (DMF), facilitated the Sonogashira coupling only (entries 3 and 4 in Table 1). Investigating the effect of the base, we found that both Cs_2_CO_3_ and K_3_PO_4_ provided the desired indole in good yields (entries 5 and 6 in Table 1). *N*,*N*-Diisopropylethylamine (DIPEA), however, inhibited the formation of the indole core (entry 7 in Table 1). Switching from the precatalyst system to the simple addition of sSPhos Pd G2 and CuSO_4_ at 85 °C for 2 h enhanced the yield to 78% (entry 8 in Table 1). Finally, we probed various sulfonamides, such as NTs (**1b**), NNs (**1c**), and NTf (**1d**), in the reaction (entries 9–11 in Table 1), with compounds **1b** and **1d** leading to the corresponding products **4b** or **4d** and **5b** or **5d** as major products and compound **1c** yielding product **3a** in a moderate 52% yield.

**Table 1:**
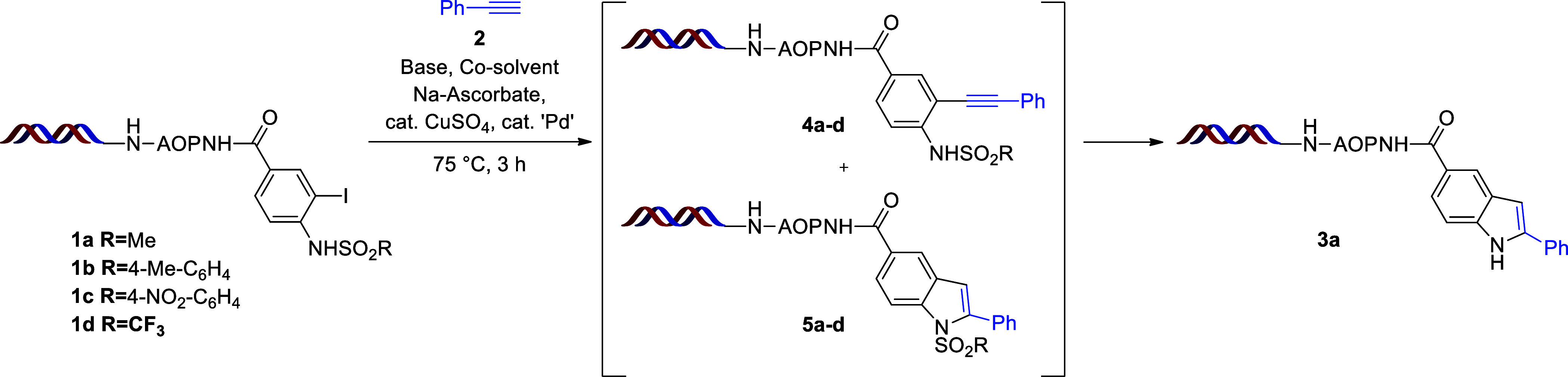
Optimization of the Sonogashira Coupling and Ring-Closure Cascade Reaction^a^

entry	co-solvent	base	yield (**3a**) (%)^b^	yield (**4** + **5**) (%)^b^
1	DMAc	K_2_CO_3_	9	78
2	DMSO	K_2_CO_3_	73	0
3	MeCN	K_2_CO_3_	9	81
4	DMF	K_2_CO_3_	3	93
5	DMSO	Cs_2_CO_3_	71	0
6	DMSO	K_3_PO_4_	74	0
7	DMSO	DIPEA	0	>99
8^c^	DMSO	K_2_CO_3_	78	0
9 (**1b**)^c^	DMSO	K_2_CO_3_	22	78
10 (**1c**, 98)^c^^,^^d^	DMSO	K_2_CO_3_	52	16
11 (**1d**, 97)^c^^,^^d^	DMSO	K_2_CO_3_	2	88

a Reaction conditions: compound **1** (10 nmol, 500 μM in H_2_O), compound **2** (300 equiv, 100 mM in co-solvent), base (500 equiv, 500 mM in H_2_O or co-solvent for DIPEA), sodium ascorbate (25 equiv, 50 mM in H_2_O), and precatalyst [0.6 equiv, 1 mM Pd(OAc)_2_, 10 mM TPPTS, and 2 mM CuSO_4_·5H_2_O in 9:1 H_2_O/DMA] for 3 h at 75 °C.

b Yields were determined by HPLC–MS.

c sSPhos Pd G2 (0.6 equiv, 10 mM in H_2_O) and CuSO_4_·5H_2_O (1.2 equiv, 20 mM in H_2_O) for 2 h at 85 °C.

d Conversion in parentheses.

With the optimized conditions in hand, we evaluated the scope of the method on a diverse set of aromatic, heteroaromatic, and aliphatic acetylenes (**6**) starting from the N-Ms compound **1a** (Scheme 2). Mostly, we observed high to quantitative conversions leading to moderate to good yields. HPLC–MS results indicated only minor deiodination, and no spontaneous desulfonylation of the starting material was observed.

**Scheme 2:**
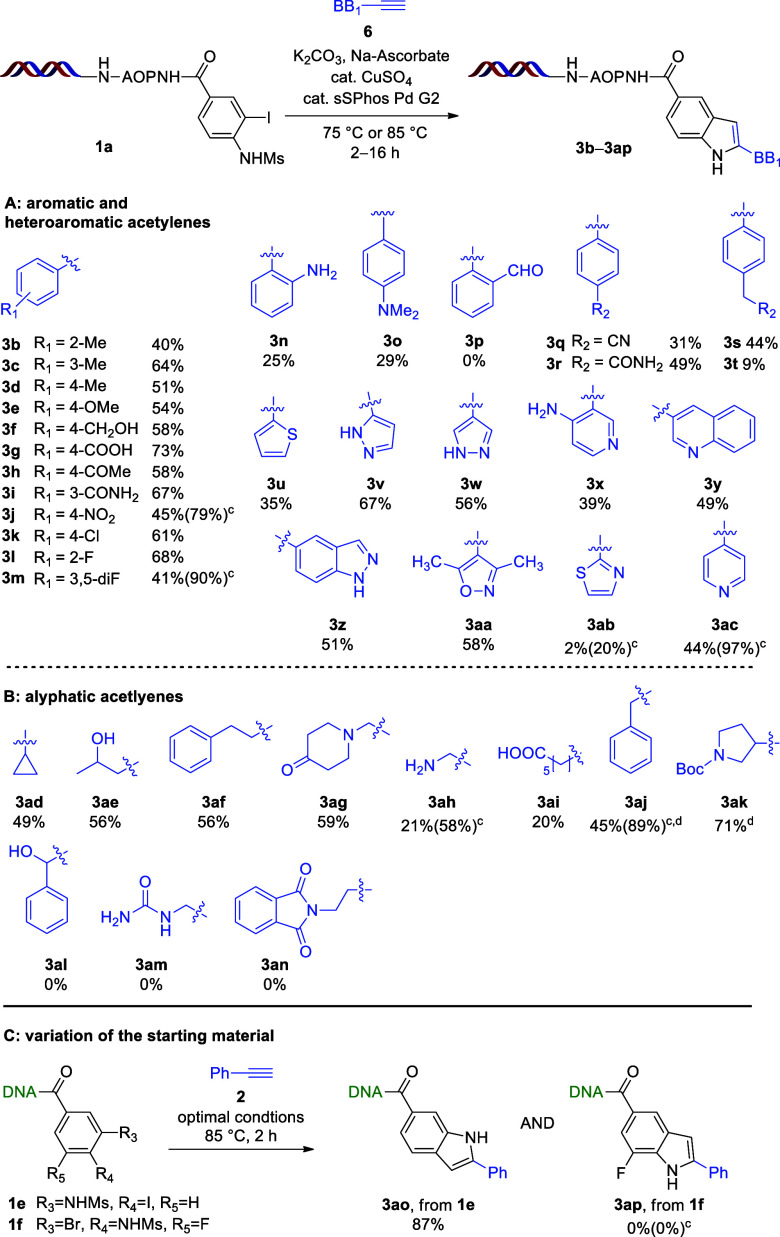
Scope of Acetylenes in the Cascade Sonogashira Coupling and Intramolecular Ring Closure^,^ Reaction conditions: compound **1a** (10 nmol, 500 μM in H_2_O), compound **6** (300 equiv, 100 mM in DMA), K_2_CO_3_ (500 equiv, 500 mM in H_2_O), Na ascorbate (25 equiv, 50 mM in H_2_O), sSPhos Pd G2 (0.6 equiv, 10 mM in H_2_O), and CuSO_4_·5H_2_O (1.2 equiv, 20 mM in H_2_O) for 2 h at 85 °C for aromatic and heteroaromatic acetylenes and for 8 h at 85 °C for aliphatic acetylenes. Yields were determined by HPLC–MS. Conversions in parentheses. Reaction was conducted at 75 °C overnight.

The developed method tolerated the presence of alkyl, alkoxy, hydroxymethyl, COOH, COMe, CONH_2_, and NO_2_ groups and halogen atoms (**3b**–**3m**). Amine-containing derivatives **3n** and **3o** were obtained in 25 and 29% yields, respectively, as a result of the formation of unidentified side products. In the case of the 2-formyl derivative (**3p**), we observed the full conversion of the starting material **1a** in the Sonogashira coupling; however, the reaction conditions did not promote the ring closure to the desired indole. CN-containing **3q** underwent partial hydrolysis, presumably resulting in terminal amide **3r**, a phenomenon that was almost totally suppressed in the case of benzylic CN (**3s** and **3t**). The applied reaction conditions enabled the incorporation of thiophene, pyrazoles, aminopyridine, quinoline, and indazole rings in the second position in 35–67% yields (**3u**–**3z**). Notably, steric hindrance around the reaction center did not compromise the formation of indole, demonstrated by multisubstituted oxazole **3aa** obtained in 58% yield. In the case of thiazole derivative **3ab**, the Sonogashira coupling was not initiated. We observed 10% deiodination in the case of pyridine derivative **3ac**, eventually leading to a 44% yield. Aliphatic acetylenes required 85 °C for 8 h or 75 °C for overnight reaction times to effectively promote the ring closure. We obtained cyclopropyl **3ad**, pent-2-ol **3ae**, ethylene benzene **3af**, and piperidine-4-one **3ag** derivatives in 49, 56, 56, and 59% yields, respectively, at 85 °C after overnight reaction. Propargyl amine provided the expected product **3ah** in 21% yield as a result of low conversion in the Sonogashira coupling. In the case of octanoic acid, we observed full conversion; however, inefficient ring closure provided product **3ai** in 20% yield. Benzylic derivative **3aj** and *N*-Boc-protected tetrahydropyrrole **3ak** were synthesized at 75 °C overnight, leading to yields of 45 and 71%, respectively. Under the applied reaction conditions, benzylic alcohol derivative **3al** was completely degraded, while in the case of the urea and phthalimide derivatives (**3am** and **3an**), we only obtained unidentified side products. Changing the position of iodine and the NHM group on the starting material provided expected indole **3ao** in 87% yield. When the halogen atom was changed to bromine (**3ap**), however, the starting material remained intact.

Applying the on-DNA iodination conditions of Lu et al.,^27^ we briefly optimized the reaction conditions for the Suzuki coupling starting from iodoindole (**7**) and phenylboronic acid (**8**)^26^ (see the Supporting Information for the complete optimization). The nature of the organic co-solvent and the base greatly influences the efficiency of the Suzuki coupling, with the main side reaction being the deiodination of the starting material to product **3a**. In the presence of DMSO as a co-solvent, we obtained the desired product **9a** in 17% yield, experiencing substantial deiodination of the starting material (entry 1 in Table 2). Switching to DMAc or DMF suppressed the side reaction and also enhanced the yield of product **9a** to 55 and 53%, respectively. The best results were obtained using dioxane, leading to a 60% yield (entries 2–4 in Table 2). With regard to the base, Et_3_N favored the deiodination and the application of K_3_PO_4_ led to only moderate yields (entries 5 and 6 in Table 2). Further experiments revealed that KOAc and KF are both interchangeable with K_2_CO_3_, giving flexibility in synthetic design (entries 7 and 8 in Table 2). Eventually, the best results were obtained when we adjusted the reaction temperature and time to 65 °C and 1 h, leading to a 63% yield of product **9a** (entry 9 in Table 2).

**Table 2:**
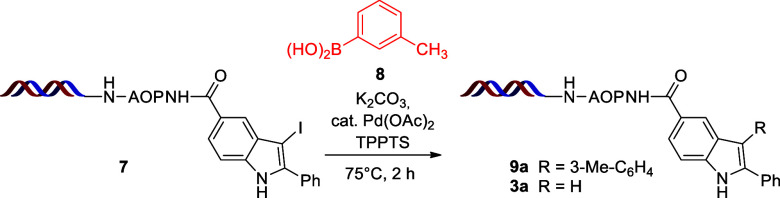
Optimization of the Suzuki Coupling^a^

entry	co-solvent	base	yield (**9a**) (%)^b^	yield (**3a**) (%)^b^
1	DMSO	K_2_CO_3_	17	63
2	DMAc	K_2_CO_3_	55	26
3	DMF	K_2_CO_3_	53	30
4	dioxane	K_2_CO_3_	60	25
5	dioxane	Et3N	30	49
6	dioxane	K_3_PO_4_	45	40
7	dioxane	KOAc	59	24
8	dioxane	KF	60	24
9^c^	dioxane	K_2_CO_3_	63	29

a Reaction conditions: compound **7** (10 nmol, 500 μM in H_2_O), compound **8** (200 equiv, 100 mM in co-solvent), base (500 equiv, 500 mM in H_2_O or co-solvent for Et_3_N), and precatalyst [0.6 equiv, 1 mM Pd(OAc)_2_ and 10 mM TPPTS in 9:1 H_2_O/DMA] for 2 h at 75 °C.

b Yields were determined by HPLC-MS.

c At 65 °C for 1 h.

Aside from phenylboronic acid, the optimized method tolerated a wide range of functional groups, such as alkyl, OCF_3_, CF_3_, halogen atoms, hydroxymethyl, nitrile, and formyl groups, leading to yields between 54 and 77% (**9b**–**9j**; Scheme 3). We observed excessive deiodination of the starting material **7** in the case of the 3-acrylic amide, 4-OH, and 4-NH_2_ derivatives, leading to low yields (**9k**–**9m**). In the presence of *ortho*-CF_3_, 2,4-OMe, and bis-*ortho*-substituted boronic acids, the starting materials were mostly deiodinated (**9n**–**9q**). The trimethoxy and pentafluoro phenyl boronic acids inhibited the reaction, only enabling moderate conversions and no generation of the expected products **9r** and **9s**.

**Scheme 3:**
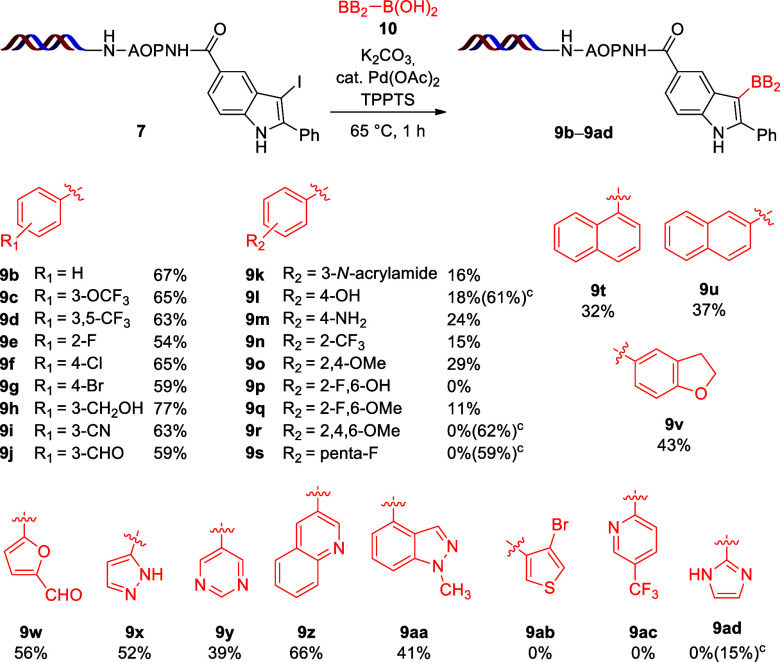
Scope of Boronic Acids in the Suzuki Coupling^,^ Reaction conditions: compound **7** (10 nmol, 500 μM in H_2_O), compound **10** (200 equiv, 100 mM in dioxane), K_2_CO_3_ (500 equiv, 500 mM in H_2_O), and precatalyst [0.6 equiv, 1 mM Pd(OAc)_2_ and 10 mM TPPTS in 9:1 H_2_O/DMA] for 1 h at 65 °C. Yields were determined by HPLC–MS. Conversions in parentheses.

Condensed cycles, such as α- and β-naphtalene or dihydrobenzofuran boronic acids, and the heterocyclic furan, pyrazole, pyrimidine, quinoline, and indazole boronic acids underwent the Suzuki coupling, leading to the corresponding products **9t**–**9aa** in moderate to good yields. α-Bromothiophene **9ab** and the pyridine derivative **9ac** suffered excessive deiodination, while 2-imidazole boronic acid blocked the reaction almost completely (**9ad**).

Next, we checked the HPLC–MS spectra for typical damage types and validated the feasibility of enzymatic ligation subsequent to the indole synthesis.^28^ Additional quantitative polymerase chain reaction (qPCR) experiments indicated insignificant damage to the DNA barcode compared to the control compound. Finally, we designed a 1 × 2 × 3 mock library to demonstrate the application of the developed method in DEL synthesis starting from compound **1a** using selected building blocks of the validation phase (see the Supporting Information).

In conclusion, we disclosed the first on-DNA indole synthesis using a Sonogashira coupling and intramolecular ring-closure cascade. Iodination at the third position, followed by Suzuki coupling, resulted in the derivatization of the heterocycle. This process enables the generation of indole-based DELs functionalized at the pharmacologically most relevant positions. We believe that bridging this method with known indole modifications could expose the chemical space of therapeutically relevant indoles toward DEL applications.

## Data Availability

The data underlying this study are available in the published article and its Supporting Information.

## Supporting Information Available

General methods, synthetic procedures, copies of HPLC–MS and deconvoluted MS spectra, DNA damage and qPCR evaluation, cell-based affinity screening and product characterization (PDF)

## References

[ref1] Shi Y.; Wu Y. R.; Yu J. Q.; Zhang W. N.; Zhuang C. L. DNA-Encoded Libraries (DELs): A Review of on-DNA Chemistries and Their Output. RSC Adv. 2021, 11 (4), 2359–2376. 10.1039/D0RA09889B. 35424149 PMC8693808

[ref2] Fitzgerald P. R.; Paegel B. M. DNA-Encoded Chemistry: Drug Discovery from a Few Good Reactions. Chem. Rev. 2021, 121, 7155–7177. 10.1021/acs.chemrev.0c00789. 33044817 PMC8345262

[ref3] Götte K.; Chines S.; Brunschweiger A. Reaction Development for DNA-Encoded Library Technology: From Evolution to Revolution?. Tetrahedron Lett. 2020, 61, 151889 10.1016/j.tetlet.2020.151889.

[ref4] Peterson A. A.; Liu D. R. Small-Molecule Discovery through DNA-Encoded Libraries. Nat. Rev. Drug Discovery 2023, 22, 699–722. 10.1038/s41573-023-00713-6. 37328653 PMC10924799

[ref5] Wan Y.; Li Y.; Yan C.; Yan M.; Tang Z. Indole: A Privileged Scaffold for the Design of Anti-Cancer Agents. Eur. J. Med. Chem. 2019, 183, 111691 10.1016/j.ejmech.2019.111691. 31536895

[ref6] Sravanthi T. V.; Manju S. L. Indoles—A Promising Scaffold for Drug Development. Eur. J. Pharm. Sci. 2016, 91, 1–10. 10.1016/j.ejps.2016.05.025. 27237590

[ref7] Vitaku E.; Smith D. T.; Njardarson J. T. Analysis of the Structural Diversity, Substitution Patterns, and Frequency of Nitrogen Heterocycles among U.S. FDA Approved Pharmaceuticals. J. Med. Chem. 2014, 57 (24), 10257–10274. 10.1021/jm501100b. 25255204

[ref8] Kumari A.; Singh R. K. Medicinal Chemistry of Indole Derivatives: Current to Future Therapeutic Prospectives. Bioorg. Chem. 2019, 89, 103021 10.1016/j.bioorg.2019.103021. 31176854

[ref9] Dadashpour S.; Emami S. Indole in the Target-Based Design of Anticancer Agents: A Versatile Scaffold with Diverse Mechanisms. Eur. J. Med. Chem. 2018, 150, 9–29. 10.1016/j.ejmech.2018.02.065. 29505935

[ref10] Xu H.; Wang Y.; Dong H.; Zhang Y.; Gu Y.; Zhang S.; Meng Y.; Li J.; Shi X. J.; Ji Q.; Liu L.; Ma P.; Ma F.; Yang G.; Hou W. Selenylation Chemistry Suitable for On-Plate Parallel and On-DNA Library Synthesis Enabling High-Throughput Medicinal Chemistry. Angew. Chem., Int. Ed. 2022, 61, e202206516 10.1002/anie.202206516. 35579067

[ref11] Lin B.; Lu W.; Chen Z. Y.; Zhang Y.; Duan Y. Z.; Lu X.; Yan M.; Zhang X. J. Enhancing the Potential of Miniature-Scale DNA-Compatible Radical Reactions via an Electron Donor-Acceptor Complex and a Reversible Adsorption to Solid Support Strategy. Org. Lett. 2021, 23 (19), 7381–7385. 10.1021/acs.orglett.1c02562. 34546064

[ref12] Xu H.; Gu Y.; Zhang S.; Xiong H.; Ma F.; Lu F.; Ji Q.; Liu L.; Ma P.; Hou W.; Yang G.; Lerner R. A. A Chemistry for Incorporation of Selenium into DNA-Encoded Libraries. Angew. Chem., Int. Ed. 2020, 59 (32), 13273–13280. 10.1002/anie.202003595. 32282979

[ref13] Yang S.; Zhao G.; Gao Y.; Sun Y.; Zhang G.; Fan X.; Li Y.; Li Y. In-Solution Direct Oxidative Coupling for the Integration of Sulfur/Selenium into DNA-Encoded Chemical Libraries. Chem. Sci. 2022, 13 (9), 2604–2613. 10.1039/D1SC06268A. 35340849 PMC8890091

[ref14] Cai P.; Yang G.; Zhao L.; Wan J.; Li J.; Liu G. Synthesis of C3-Alkylated Indoles on DNA via Indolyl Alcohol Formation Followed by Metal-Free Transfer Hydrogenation. Org. Lett. 2019, 21 (17), 6633–6637. 10.1021/acs.orglett.9b02132. 31411480

[ref15] Rozenman M. M.; Kanan M. W.; Liu D. R. Development and Initial Application of a Hybridization-Independent, DNA-Encoded Reaction Discovery System Compatible with Organic Solvents. J. Am. Chem. Soc. 2007, 129 (48), 14933–14938. 10.1021/ja074155j. 17994738 PMC2538361

[ref16] Wen X.; Duan Z.; Liu J.; Lu W.; Lu X. On-DNA Cross-Dehydrogenative Coupling Reaction toward the Synthesis of Focused DNA-Encoded Tetrahydroisoquinoline Libraries. Org. Lett. 2020, 22 (15), 5721–5725. 10.1021/acs.orglett.0c01565. 32644810

[ref17] Li K.; Liu X.; Liu S.; An Y.; Shen Y.; Sun Q.; Shi X.; Su W.; Cui W.; Duan Z.; Kuai L.; Yang H.; Satz A. L.; Chen K.; Jiang H.; Zheng M.; Peng X.; Lu X. Solution-Phase DNA-Compatible Pictet-Spengler Reaction Aided by Machine Learning Building Block Filtering. iScience 2020, 23 (6), 101142 10.1016/j.isci.2020.101142. 32446221 PMC7243192

[ref18] Gironda-Martínez A.; Gorre É. M. D.; Prati L.; Gosalbes J. F.; Dakhel S.; Cazzamalli S.; Samain F.; Donckele E. J.; Neri D. Identification and Validation of New Interleukin-2 Ligands Using DNA-Encoded Libraries. J. Med. Chem. 2021, 64 (23), 17496–17510. 10.1021/acs.jmedchem.1c01693. 34821503

[ref19] Zhong S.; Fang X.; Wang Y.; Zhang G.; Li Y.; Li Y. DNA-Compatible Diversification of Indole π-Activated Alcohols via a Direct Dehydrative Coupling Strategy. Org. Lett. 2022, 24 (4), 1022–1026. 10.1021/acs.orglett.1c04169. 35050627

[ref20] Yang J.; Xia S.; Liu J.; Yu Z.; Mei L.; Lu W.; Chen Y.; Chen S.; Wang X.; Lu X. DNA-Encoded Focused Indazole Library Synthesis by a Palladium-Mediated C–N(sp^2^) Cross-Coupling Reaction between DNA-Linked (Hetero)Aryl Halides and Aromatic Nitrogen Heterocycles. Tetrahedron Lett. 2022, 96, 153732 10.1016/j.tetlet.2022.153732.

[ref21] A Handbook for DNA-Encoded Chemistry: Theory and Applications for Exploring Chemical Space and Drug Discovery; Goodnow R. A. Jr., Ed.; John Wiley & Sons, Inc.: Hoboken, NJ, 2014; 10.1002/9781118832738.

[ref22] Li J. J. Name Reactions: A Collection of Detailed Mechanisms and Synthetic Applications; Springer: Cham, Switzerland, 2021; 10.1007/978-3-030-50865-4.

[ref23] Kazmierski W. M.; Xia B.; Miller J.; De La Rosa M.; Favre D.; Dunham R. M.; Washio Y.; Zhu Z.; Wang F.; Mebrahtu M.; Deng H.; Basilla J.; Wang L.; Evindar G.; Fan L.; Olszewski A.; Prabhu N.; Davie C.; Messer J. A.; Samano V. DNA-Encoded Library Technology-Based Discovery, Lead Optimization, and Prodrug Strategy toward Structurally Unique Indoleamine 2,3-Dioxygenase-1 (IDO1) Inhibitors. J. Med. Chem. 2020, 63 (7), 3552–3562. 10.1021/acs.jmedchem.9b01799. 32073266

[ref24] Hiroya K.; Itoh S.; Sakamoto T. Mild and Efficient Cyclization Reaction of 2-Ethynylaniline Derivatives to Indoles in Aqueous Medium. Tetrahedron 2005, 61 (46), 10958–10964. 10.1016/j.tet.2005.08.098.

[ref25] Satz A. L.; Cai J.; Chen Y.; Goodnow R.; Gruber F.; Kowalczyk A.; Petersen A.; Naderi-Oboodi G.; Orzechowski L.; Strebel Q. DNA Compatible Multistep Synthesis and Applications to DNA Encoded Libraries. Bioconjugate Chem. 2015, 26 (8), 1623–1632. 10.1021/acs.bioconjchem.5b00239. 26024553

[ref26] Favalli N.; Bassi G.; Bianchi D.; Scheuermann J.; Neri D. Large Screening of DNA-Compatible Reaction Conditions for Suzuki and Sonogashira Cross-Coupling Reactions and for Reverse Amide Bond Formation. Bioorg. Med. Chem. 2021, 41, 116206 10.1016/j.bmc.2021.116206. 34038862

[ref27] Liu S.; Qi J.; Lu W.; Wang X.; Lu X. Synthetic Studies toward DNA-Encoded Heterocycles Based on the on-DNA Formation of α,β-Unsaturated Ketones. Org. Lett. 2021, 23 (3), 908–913. 10.1021/acs.orglett.0c04118. 33444029

[ref28] Wang H.; Zhao G.; Zhang T.; Li Y.; Zhang G.; Li Y. Comparative Study of DNA Barcode Integrity Evaluation Approaches in the Early-Stage Development of DNA-Compatible Chemical Transformation. ACS Pharmacol. Transl. Sci. 2023, 6, 1724–1733. 10.1021/acsptsci.3c00181. 37974618 PMC10644510

