# Supplementary material for: On-DNA Synthesis of Multisubstituted Indoles
Source: Org Lett. 2023 Dec 18;26(13):2517–22. doi: 10.1021/acs.orglett.3c03602 (PMC11002923; doi:10.1021/acs.orglett.3c03602)
Supplement: Supplementary file 1 — ol3c03602_si_001.pdf [file ol3c03602_si_001.pdf]

Supporting information

On-DNA synthesis of multisubstituted indoles

András Gy. Németh, Levente Kollár, Krisztina Németh, Gitta Schlosser, Annamária Minus, György M. Keserű

Table of contents

1. General information 2

a. Materials and general procedures 2

2. Preparation of building blocks and small molecules 4

3. Preparation of on-DNA starting materials 13

4. Optimization of reaction conditions 15

a. Optimization of the Sonogashira coupling and intramolecular ring-closure 15

b. General recipe for the Sonogashira coupling and intramolecular ring-closure 16

c. Optimization of the Suzuki coupling 16

d. General recipe for the Suzuki coupling 17

5. Ligation and qPCR experiments 17

a. Ligation validation 17

b. qPCR measurement 18

6. Affinity screening against Dopamin D_3 receptors 21

7. Synthesis of the mock library 24

8. Control reactions 30

a. HPLC-MS spectra of the control reactions 30

b. HPLC-MS co-injection 38

9. HPLC-MS spectra 40

10. NMR spectra 118

11. References 132

1. General information

a. Materials and general procedures

The chemically modified DNA oligonucleotide headpiece (HP, 5'-/5Phos/GAGTCA/iSp9/iUniAmM/iSp9/TGACTCCC-3', **Figure S1**) and all coding sequences were purchased from HitGen Inc. T4 DNA ligase, ligation buffer, TBE buffer, ultra-low range DNA ladder and TrackIt Cyan/Yellow Loading Dye was purchased from Thermo Fisher Scientific. Tris-HCl, EDTA, NaHEPES were obtained from Sigma-Aldrich, D3R agonist, 7-OH-DPAT was obtained from Tocris Bioscience. CHO-hD3 cell line, the radiolabeled [^{3}H]7-OH-DPAT (specific activity: 100 Ci/mmol), and the Ultima GoldTM MV harmless scintillation cocktail were acquired from PerkinElmer. Borate buffer (250 mM sodium borate/boric acid, pH 9.4) and palladium precatalyst solutions were prepared in-house. Chemical building blocks and reagents were either purchased from a variety of vendors or prepared in-house. Reagents were generally used from aliquots dissolved in organic solvents or water depending on solubility and optimized reaction conditions.

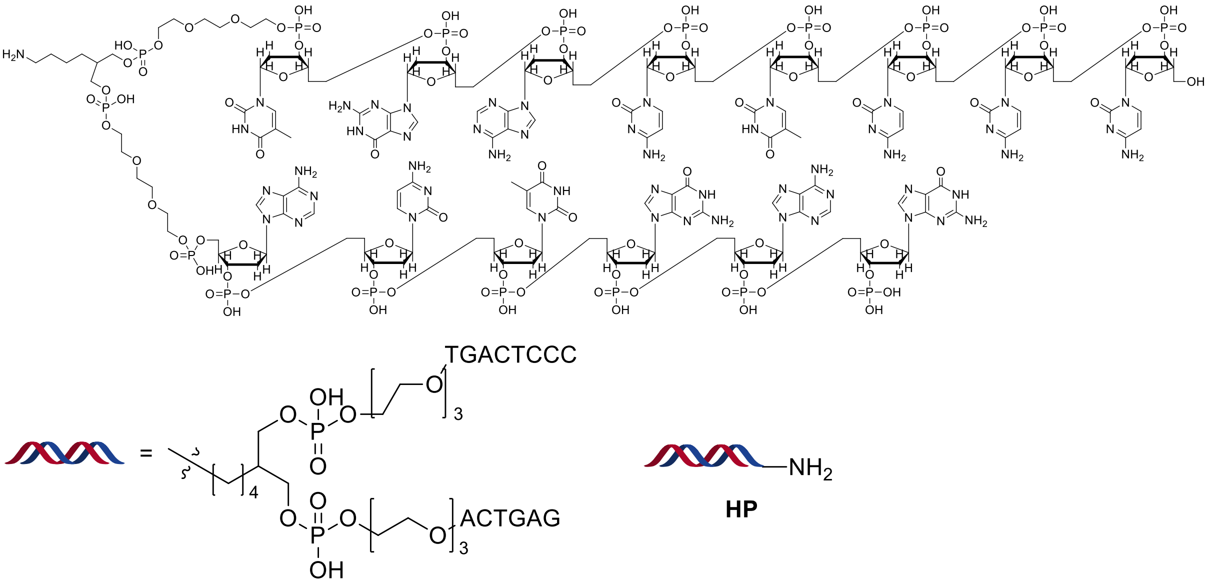

Figure S1. Structure of DNA-Headpiece

i. General procedure for the analysis of oligonucleotides and small molecules

HPLC-MS analyses were performed on a Thermo Scientific Q Exactive Focus, high resolution, and high mass accuracy, hybrid quadrupole-orbitrap mass spectrometer (Bremen, Germany) using on-line UHPLC coupling. UHPLC separation was performed on a Dionex 3000 UHPLC system using a Waters Acquity UPLC BEH300 C4 column (2.1 x 150 mm, 1.7 µm). Linear gradient elution (0 min 10% B, 1.0 min 10% B, 20.0 min 70% B, 20.5 min 100% B, 21.0 min

100% B. 21.5 min 10% B, 26.0 min 10% B) with eluent A (1% HFIP, 0.1% DIPEA, 10 µM EDTA in water) and eluent B (0.075% HFIP, 0.0375% DIPEA, 10 µM EDTA, in acetonitrile/water 65:35 V/V) was used at a flow rate of 0.400 mL/min at 65 ^{o}C column temperature. High-resolution mass spectra were acquired in the 550-2000 *m/z* range in negative ionization mode. Data were analyzed with FreeStyle™ 1.3 of Thermo Fisher Scientific Inc.

^{1}H- and ^{13}C NMR spectra were recorded in DMSO-d_6 or $CDCl_3$ solution at room temperature, on a Varian Unity Inova 500 spectrometer (500 and 125 MHz for ^{1}H- and ^{13}C NMR spectra, respectively), with the residual solvent signal as the lock and TMS as the internal standard. Chemical shifts (δ) and coupling constants (J) are given in ppm and Hz, respectively. High-resolution MS measurements of small molecules were performed on a Sciex TripleTOF 5600+ high-resolution tandem mass spectrometer equipped with a DuoSpray ion source (Sciex, MA, USA). Electrospray ionization was applied in negative or positive ion detection mode as indicated. Samples were dissolved in acetonitrile and flow injected into acetonitrile:water 50:50 flow. The flow rate was 0.2 mL/min. The resolution of the mass spectrometer was 35000. Data acquisition and processing were performed using Analyst TF Software 1.7.1 (Sciex, MA, USA).

ii. General procedure for ethanol precipitation

To a DNA reaction mixture was added 10 V/V% 5 M sodium chloride solution and 300 V/V% absolute ethanol. The solution was then incubated in dry ice for two hours or at -20 °C overnight. The precipitated material was then isolated as a pellet by centrifugation at 14000 rcf for 30 minutes and subsequent removal of the supernatant.

iii. General procedure for DNA ligation

To the aqueous solution of the DNA starting material (10 nmol, 10 µL in H_2O) ligation buffer (10X, 4 µL) was added and the resulting solution was cooled to 16 °C. After the addition of the coding sequences (13 nmol, 13 µL in H_2O) the sample was vortexed and centrifuged. Finally, T4 DNS ligase (30 U/µL, 0.4 µL) was added and the solution was incubated at 16 °C overnight. The crude material was purified by ethanol precipitation and taken on to the next step of synthesis without further purification. Ligation reactions were monitored by gel electrophoresis on 25% polyacrylamide gel in TBE buffer (40 mM Tris-Cl, 45 mM boric acid, 1 mM EDTA, pH 8.3). The system was referenced by an ultra-low range DNA ladder (10 bp to 300 bp). Before gel loading, the DNA samples were mixed with 20 V/V% TrackIt Cyan/Yellow Loading Dye. Then, 40 pmol of DNA samples were loaded on the gel and it was run at 100 V for 60–75 min.

The gel was stained with ethidium bromide (1 µg/mL) and the DNA fragments were visualized and analyzed by Bio-Rad ChemiDoc XRS+ Imaging system (Bio-Rad, CA, USA).

2. Preparation of building blocks and small molecules

a. General procedure for the preparation of S3–S7:

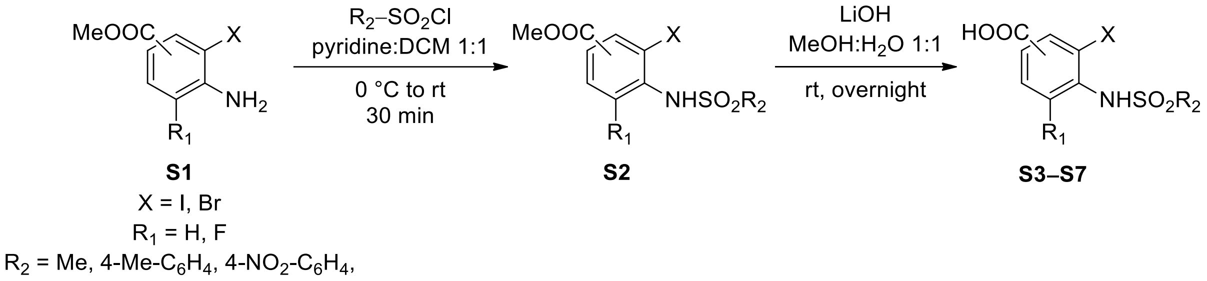

To the solution of the amine **S1** (1.0 mmol) in a 1:1 mixture of dichloromethane:pyridine (2 mL) under argon atmosphere, the corresponding sulfonyl chloride (1.1 mmol, 1.1 equivalents) was added at 0 °C dropwise. The reaction was stirred at room temperature for 30 minutes, then diluted with dichloromethane (10 mL) and extracted with 1.0 M aqueous hydrochloric acid (10 mL). The aqueous phase was extracted two times with dichloromethane (10–10 mL) and the merged organic phases were washed with brine (20 mL), dried over sodium sulfate, and evaporated. The crude product was purified by reverse-phased flash column chromatography using water and acetonitrile as eluents. The obtained NH-sulfonyl compounds **S2** were suspended in a 1:1 mixture of methanol:water (10 mL) and lithium hydroxide (0.24 g, 10 mmol, 10 equivalents) was added. The resulting mixture was stirred at room temperature overnight, acidified to pH~2 with 1.0 M aqueous hydrochloric acid, and extracted with ethyl acetate (15 mL) twice. The merged organic phases were washed with water and brine (15–15 mL), dried over sodium sulfate, and evaporation provided **S3–S7**.

3-Iodo-4-methanesulfonamidobenzoic acid (**S3**)

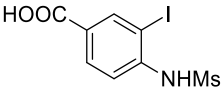

S3

Yield: 34%; white solid; 116 mg; ^{1}H NMR (500 MHz, DMSO-d_6) δ 8.35 (d, J = 1.7 Hz, 1H), 7.91 (dd, J = 8.4, 1.8 Hz, 1H), 7.49 (d, J = 8.4 Hz, 1H), 3.11 (s, 3H), resonances for COOH and NH are not visible; ^{13}C NMR (125 MHz, DMSO-d_6) δ 165.5, 143.4, 140.3, 130.0, 128.7, 124.3, 95.4, 41.4; HRMS (ESI$^-$) *m/z* [M–H]$^-$, calcd. for $C_8H_7NO_4SI$: 339.9140, found: 339.9131.

4-Iodo-3-methanesulfonamidobenzoic acid (**S4**)

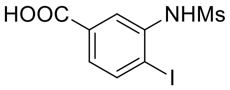

S4

Yield: 29%, 99 mg; white solid; ^{1}H NMR (500 MHz, DMSO-*d$_6$*) δ 13.24 (s, 1H), 9.41 (s, 1H), 8.05 (d, *J* = 8.1 Hz, 1H), 7.88 (d, *J* = 1.9 Hz, 1H), 7.53 (dd, *J* = 8.2, 2.0 Hz, 1H), 3.07 (s, 3H); ^{13}C NMR (125 MHz, DMSO-*d$_6$*) δ 166.3, 140.1, 139.1, 131.8, 128.3, 127.3, 104.4, 41.3; HRMS (ESI^{-}) *m/z* [M–H]$^{-}$, calcd. for $C_8H_7NO_4SI$: 339.9140, found: 339.9131.

3-Iodo-4-(4-methylbenzenesulfonamido)benzoic acid (**S5**)

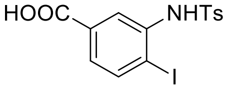

S5

Yield: 35%, 146 mg; white solid; ^{1}H NMR (500 MHz, DMSO-*d$_6$*) δ 13.10 (s, 1H), 9.84 (s, 1H), 8.28 (d, *J* = 1.9 Hz, 1H), 7.84 (dd, *J* = 8.4, 1.9 Hz, 1H), 7.66 (d, *J* = 8.3 Hz, 2H), 7.38 (d, *J* = 8.1 Hz, 2H), 7.20 (d, *J* = 8.3 Hz, 1H), 2.38 (s, 3H); ^{13}C NMR (125 MHz, DMSO-*d$_6$*) δ 165.4, 143.4, 142.4, 140.3, 137.5, 129.8, 129.7, 129.5, 126.8, 125.0, 96.0, 21.0; HRMS (ESI^{-}) *m/z* [M–H]$^{-}$, calcd. for $C_{14}H_{11}NO_4SI$: 415.9453, found: 415.9465.

3-Iodo-4-(4-nitrobenzenesulfonamido)benzoic acid (**S6**)

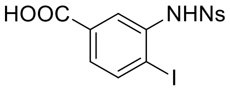

S6

Yield 23%, 103 mg; light yellow solid; ^{1}H NMR (500 MHz, DMSO-*d$_6$*) δ 10.52 (s, 1H), 8.41 – 8.37 (m, 2H), 8.27 (d, *J* = 2.0 Hz, 1H), 8.01 – 7.97 (m, 2H), 7.82 (dd, *J* = 8.4, 1.9 Hz, 1H), 7.20 – 7.16 (m, 1H), resonance for NH is not visible. ^{13}C NMR (126 MHz, DMSO-*d$_6$*) δ 165.4, 149.6, 146.8, 140.3, 129.9, 128.3, 125.8, 124.6, 97.2; HRMS (ESI^{-}) *m/z* [M–H]$^{-}$, calcd. for $C_{13}H_8N_2O_6SI$: 446.9147, found: 446.9162.

3-Bromo-5-fluoro-4-methanesulfonamidobenzoic acid (**S7**)

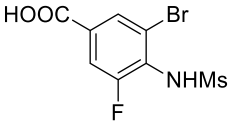

S7

Yield: 22%, 69 mg; white solid; ^{1}H NMR (500 MHz, DMSO-d_6) δ 8.00 (d, J = 1.3 Hz, 1H), 7.78 (dd, J = 9.7, 1.7 Hz, 1H), 3.16 (s, 3H), resonances for COOH and NH are not visible. ^{13}C NMR (126 MHz, DMSO-d_6) δ 164.7 (d, *J* = 2.3 Hz), 158.7 (d, *J* = 252.0 Hz), 132.2 (d, *J* = 7.7 Hz), 129.2 (d, *J* = 3.1 Hz), 128.3 (d, *J* = 15.6 Hz), 124.7 (s), 116.3 (d, *J* = 23.2 Hz), 42.8 (d, *J* = 3.3 Hz). HRMS (ESI$^-$) *m/z* [M–H]$^-$, calcd. for $C_8H_6NO_4FSBr$: 309.9184, found: 309.9175.

b. Preparation of S10

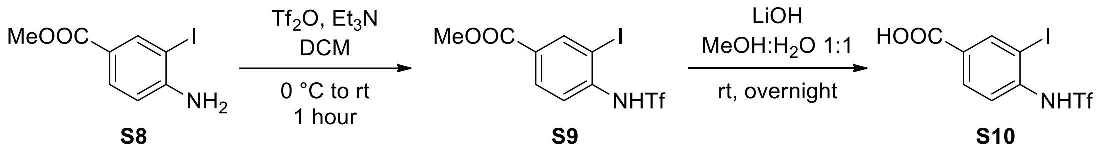

To the solution of **S8** (277 mg, 1.0 mmol) in dichloromethane (2 mL) triethylamine (348 µL, 2.5 mmol, 2.5 equivalents) was added and the resulting solution was cooled to 0 °C. Then, the solution of trifluoromethane sulfonic anhydride (252 µL, 1.5 mmol, 1.5 equivalents) in dichloromethane (1 mL) was added dropwise and the reaction proceeded at room temperature for 1 hour. The reaction was quenched with a 1:2 mixture of 1.0 M NaOH and methanol (15 mL), stirred for 1 hour, and then acidified to pH~2 with 1.0 M aqueous hydrochloric acid and extracted with ethyl acetate (15 mL) twice. The merged organic phases were dried over sodium sulfate and evaporated. The crude product was purified by reverse-phased flash column chromatography using water and acetonitrile as eluents. The obtained *N*-triflate compound **S9** was suspended in a 1:1 mixture of methanol:water (10 mL) and lithium hydroxide (0.24 g, 10 mmol, 10 equivalents) was added. The resulting mixture was stirred at room temperature overnight, acidified to pH~2 with 1.0 M aqueous hydrochloric acid, and extracted with ethyl acetate (15 mL) twice. The merged organic phases were washed with water and brine (15–15 mL), dried over sodium sulfate and evaporation provided the carboxylic acid **S10**.

3-Iodo-4-(trifluoromethanesulfonamido)benzoic acid (**S10**)
Yield: 36%, 143 mg; pale brown solid; ^{1}H NMR (500 MHz, DMSO-d_6) δ 8.26 (d, *J* = 2.0 Hz, 1H), 7.76 (dd, *J* = 8.5, 2.0 Hz, 1H), 7.39 (d, *J* = 8.5 Hz, 1H), resonances for COOH and NH are not visible; ^{13}C NMR (125 MHz, DMSO-d_6) δ 166.1, 150.2, 139.9, 129.8, 125.1, 122.4, 120.0, 96.6; HRMS (ESI$^-$) *m/z* [M–H]$^-$, calcd. for $C_8H_4NO_4F_3SI$: 393.8857, found: 393.8852.

c. Preparation of S12

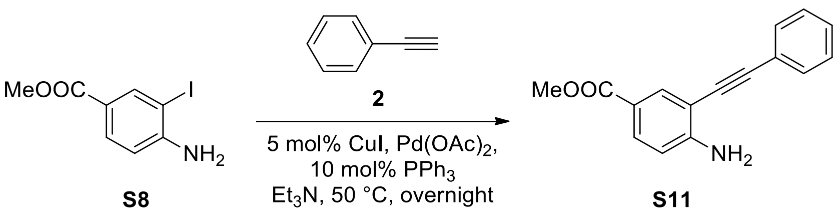

To the solution of **S8** (277 mg, 1.00 mmol) in triethylamine (2 mL) under argon atmosphere, copper(I) iodide (10 mg, 0.05 mmol, 0.05 equivalents), palladium(II) acetate (9 mg, 0.05 mmol, 0.05 equivalents), triphenylphosphine (26 mg, 0.10 mmol, 0.10 equivalents) and phenylacetylene (**2**, 220 µL, 2.00 mmol, 2.00 equivalents) was added and the resulting mixture was stirred at 50 °C overnight. The reaction mixture was filtered through celite, washed with ethyl acetate (30 mL), and purified with normal-phase flash column chromatography in hexane–ethyl acetate to provide **S11**.

Methyl 4-amino-3-(2-phenylethynyl)benzoate (**S11**)[1]
Yield: 99%, 249 mg; pale brown solid; ^{1}H NMR (300 MHz, $CDCl_3$) δ 8.09 (d, *J* = 2.0 Hz, 1H), 7.82 (dd, *J* = 8.5, 2.0 Hz, 1H), 7.53 (ddd, *J* = 8.7, 4.0, 2.4 Hz, 2H), 7.39 – 7.33 (m, 3H), 6.70 (d, *J* = 8.5 Hz, 1H), 4.69 (s, 2H), 3.87 (s, 3H).

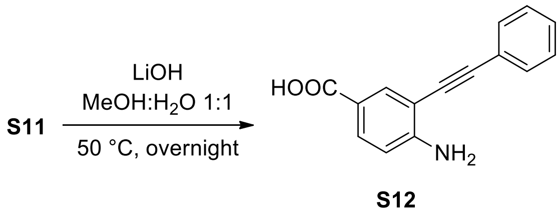

The ester **S11** (249 mg, 0.99 mmol) was suspended in a 1:1 mixture of methanol:water (10 mL), and lithium hydroxide (0.24 g, 10 mmol, 10.1 equivalents) was added. The resulting mixture was stirred at 50 °C overnight, acidified to pH~2 with 1.0 M aqueous hydrochloric acid, and extracted with ethyl acetate (15 mL) twice. The merged organic phases were washed with water (15 mL) and brine (15 mL), dried over sodium sulfate, and evaporation provided **S12**.

4-Amino-3-(2-phenylethynyl)benzoic acid (**S12**)
Yield: 89%, 214 mg; pale brown solid; ^{1}H NMR (500 MHz, DMSO-d_6) δ 12.26 (s, 1H), 7.83 (s, *J* = 1.9 Hz, 1H), 7.68 – 7.62 (m, 3H), 7.45 – 7.38 (m, 3H), 6.76 (d, *J* = 8.6 Hz, 1H), 6.25 (s, 2H); ^{13}C NMR (125 MHz, DMSO-d_6) δ 166.7, 153.2, 134.0, 131.3, 131.3, 128.5, 128.4, 122.7, 117.5, 113.1, 104.7, 94.0, 85.7; HRMS (ESI$^-$) *m/z* [M–H]$^-$, calcd. for $C_{15}H_{10}NO_2$: 236.0711, found: 236.0700.

d. Preparation of S14

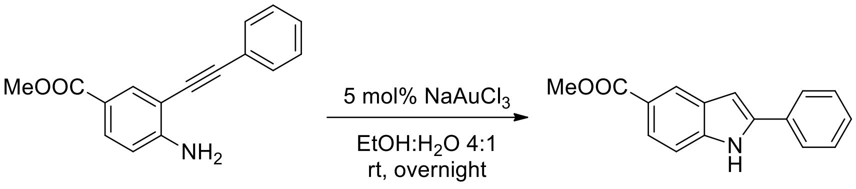

To the suspension of the ester **S11** (251 mg, 1.00 mmol) in a 4:1 mixture of ethanol:water (7.5 mL) under argon atmosphere sodium tetrachloroaurate trihydrate (4 mg, 0.05 mmol, 0.05 equivalents) was added and the resulting mixture was stirred at room temperature overnight. The crude reaction mixture was purified by normal phased flash column chromatography in hexane–ethyl acetate to provide **S13**.

Methyl 2-phenyl-1*H*-indole-5-carboxylate (**S13**)[2]
Yield 74%, 186 mg; pale brown solid; ^{1}H NMR (500 MHz, $CDCl_3$) δ δ 8.61 (s, 1H), 8.40 – 8.39 (m, 1H), 7.91 (dd, *J* = 8.5, 1.6 Hz, 1H), 7.70 – 7.66 (m, 2H), 7.46 (dd, *J* = 10.6, 4.8 Hz, 2H), 7.41 (d, *J* = 8.5 Hz, 1H), 7.36 (dd, *J* = 9.3, 5.6 Hz, 1H), 6.90 (dd, *J* = 1.9, 0.6 Hz, 1H), 3.94 (s, 3H).

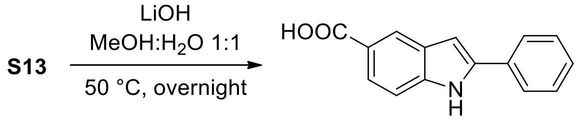

The ester **S13** (126 mg, 0.5 mmol) was suspended in a 1:1 mixture of methanol:water (5 mL) and lithium hydroxide (0.24 g, 10 mmol, 20 equivalents) was added. The resulting mixture was stirred at 50 °C overnight, acidified to pH~2 with 1.0 M aqueous hydrochloric acid, and extracted with ethyl acetate (10 mL) twice. The merged organic phases were washed with water (10 mL) and brine (10 mL), dried over sodium sulfate, and evaporation provided **S14**.

2-Phenyl-1*H*-indole-5-carboxylic acid (**S14**)[3]
Yield: quant., 119 mg; white solid; ^{1}H NMR (500 MHz, DMSO-d_6) δ 12.40 (s, 1H), 11.86 (s, 1H), 8.22 (s, 1H), 7.88 (d, *J* = 7.7 Hz, 2H), 7.73 (dd, *J* = 8.5, 1.5 Hz, 1H), 7.47 (dd, *J* = 17.1, 8.3 Hz, 3H), 7.35 (t, *J* = 7.4 Hz, 1H), 7.05 (d, *J* = 1.4 Hz, 1H).

e. Preparation of S16

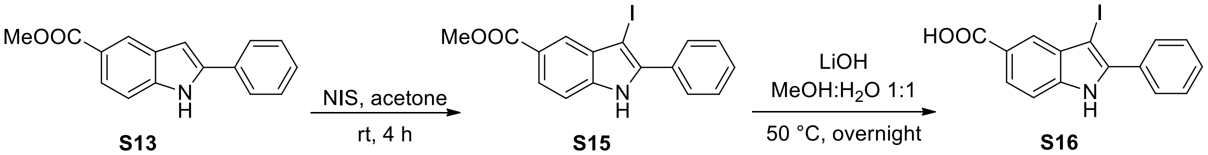

To a solution of **S13** (251 mg, 1.00 mmol) in acetone (15 mL) *N*-iodo succinimide (236 mg, 1.05 mmol, 1.05 equivalents) was added at 0 °C and the resulting mixture was stirred for 4 hours at room temperature. The reaction was quenched with saturated sodium thiosulfate (15 mL), then it was partitioned between water (20 mL) and ethyl acetate (20 mL). The aqueous phase was extracted with ethyl acetate (15 mL) two times and the merged organic phases were washed with brine (20 mL), dried over sodium sulfate, and evaporated. The crude reaction mixture was purified by normal phased flash column chromatography in hexane–ethyl acetate to provide **S15**. The ester **S15** was suspended in a 1:1 mixture of methanol:water (10 mL) and lithium hydroxide (0.24 g, 10 mmol, 10 equivalents) was added. The resulting mixture was stirred at 50 °C overnight, acidified to pH~2 with 1.0 M aqueous hydrochloric acid, and extracted with ethyl acetate (15 mL) twice. The merged organic phases were washed with water (15 mL) and brine (15 mL), dried over sodium sulfate, and evaporation provided **S16**.

3-Iodo-2-phenyl-1*H*-indole-5-carboxylic acid (**S16**)
Yield: 52%, 189 mg; light yellow solid; ^{1}H NMR (500 MHz, DMSO-d_6) δ 12.63 (s, 1H), 12.23 (s, 1H), 8.02 (s, 1H), 7.89 – 7.85 (m, 2H), 7.82 (dd, *J* = 8.5, 1.4 Hz, 1H), 7.57 (t, *J* = 7.7 Hz, 2H), 7.48 (t, *J* = 7.5 Hz, 2H); ^{13}C NMR (126 MHz, DMSO-d_6) δ 168.0, 139.6, 139.4, 131.6, 131.0, 128.7, 128.5, 125.2, 124.0, 123.2, 122.9, 111.8, 58.4; HRMS (ESI$^-$) *m/z* [M–H]$^-$, calcd. for $C_{15}H_9NO_2I$: 361.9678, found: 361.9682.

f. Off-DNA synthesis of top 3 hits from Dopamine D_3 receptor affinity selection

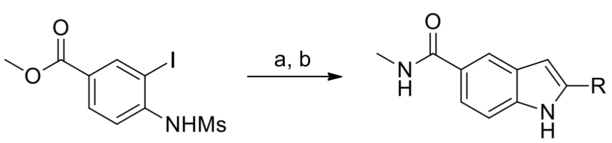

Reaction conditions ‘**a**’: corresponding acetylene, Na-ascorbate, K_2CO_3, sSPhosPd G2, $CuSO_4$, DMSO; ‘**b**’: DMF, 40 % methylamine in water.

The mesylated starting material (**S2Ms**, 0.59 mmol) was dissolved in DMSO (1 mL). The corresponding acetylene (0.59 mmol, 1.0 equivalents), potassium carbonate (1.77 mmol, 3.0 equivalents), sodium ascorbate (1.48 mmol, 2.5 equivalents), sSPhosPd G2 (0.06 mmol, 0.1 equivalents), $CuSO_4$ (0.18 mmol, 0.3 equivalents) were added to the solution. Argon inert

atmosphere was used in the reaction. The suspension was stirred at 70 °C for 1 hour, then the mixture was filtered. The filtrate was evaporated and dissolved in DMF (1 mL). Afterward, 1 mL of methylamine solution (40 % in water) was added and the resulting mixture was stirred at 50 °C for 24 hours. The solution was concentrated and purified on preparative HPLC.

tert-Butyl 3-(5-(methylcarbamoyl)-1*H*-indol-2-yl)pyrrolidine-1-carboxylate (**PUB-16**)

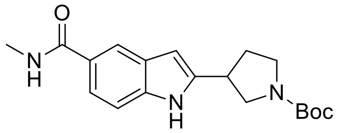

PUB-16

0.210 mg (0.59 mmol) Mesylated compound (**S2Ms**), 0.127 mg (0.65 mmol) tert-butyl 3-ethynylpyrrolidine-1-carboxylate, 0.245 mg (1.77 mmol) K_2CO_3, 0.292 mg (1.475 mmol) Na-ascorbate, 49 mg (0.059 sSPhosPd G2, 28 mg (0.177 mmol) $CuSO_4$. After purification on preparative HPLC, we obtained 32 mg (16 %) white powder. Purity: 94.7 % HRMS (ESI$^+$) *m/z* [M+H]$^+$, calcd. for $C_{19}H_{26}N_3O_3$: 344.1974, found: 344.1982 ^{1}H NMR (300 MHz, CD_3OD) δ 8.25 (s, 0H), 7.98 (s, 1H), 7.55 (d, *J* = 8.4 Hz, 1H), 7.33 (d, *J* = 8.8 Hz, 1H), 6.34 (s, 0H), 3.82 (s, 1H), 3.57 (s, 2H), 3.46 (d, *J* = 8.7 Hz, 1H), 2.93 (d, *J* = 3.8 Hz, 3H), 2.35 (s, 1H), 2.17 (s, 1H), 1.52 – 1.44 (m, 9H). ^{13}C NMR (125 MHz, DMSO-*d*$_6$) δ 168.7, 154.9, 142.0, 135.0, 132.7, 132.5, 125.1, 120.5, 111.6, 100. 98, 78.8, 53.1, 48.5, 31.7, 29.3, 27.7.

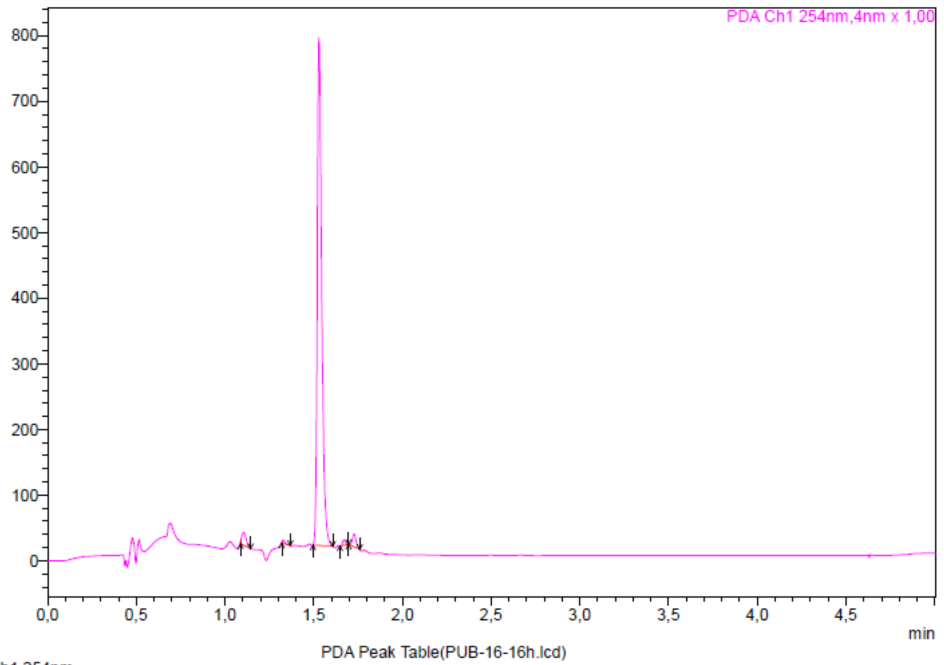

PDA Peak Table(PUB-16-16h.lcd)

PDA Ch1 254nm

Peak#	Ret. Time	Height	Area	Area%
1	1,105	17901	32335	2,270
2	1,328	4732	5698	0,400
3	1,531	742014	1350361	94,781
4	1,671	7931	9374	0,658
5	1,727	18285	26949	1,892
Total		790863	1424718	100,000

N-methyl-2-phenethyl-1*H*-indole-5-carboxamide (**PUB-17**)

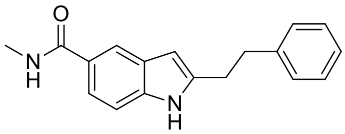

PUB-17

0.210 mg (0.59 mmol) Mesylated compound (**S2Ms**), 0.84 mg (0.091 ml, 0.65 mmol) 4-phenyl-1-butyne, 0.245 mg (1.77 mmol) K_2CO_3, 0.292 mg (1.475 mmol) Na-ascorbate, 49 mg (0.059 sSPhosPd G2, 28 mg (0.177 mmol) $CuSO_4$. After the purification on preparative HPLC we obtained 25 mg (15 %) yellowish white powder. Purity: 98.6 % HRMS (ESI$^+$) *m/z* [M+H]$^+$, calcd. for $C_{18}H_{19}N_2O$: 279.1497, found: 279.1498 ^{1}H NMR (500 MHz, $CDCl_3$) δ 8.00 (d, *J* = 14.6 Hz, 2H), 7.91 – 7.80 (m, 1H), 7.61 – 7.54 (m, 1H), 7.49 (d, *J* = 4.8 Hz, 1H), 7.32 (d, *J* = 7.6 Hz, 2H), 7.25 – 7.20 (m, 3H), 6.35 (s, 1H), 6.12 (s, 1H), 4.44 – 4.36 (m, 1H), 3.12 (d, *J* = 6.7 Hz, 2H), 3.10 – 3.03 (m, 6H). ^{13}C NMR (125 MHz, DMSO-d_6) δ 168.5, 141.0, 139.7, 135.7, 135.1, 130.4, 129.0, 128.7, 127.3, 125.5, 123.5, 121.5, 109.9, 101.33, 35.05, 29.7, 25.6.

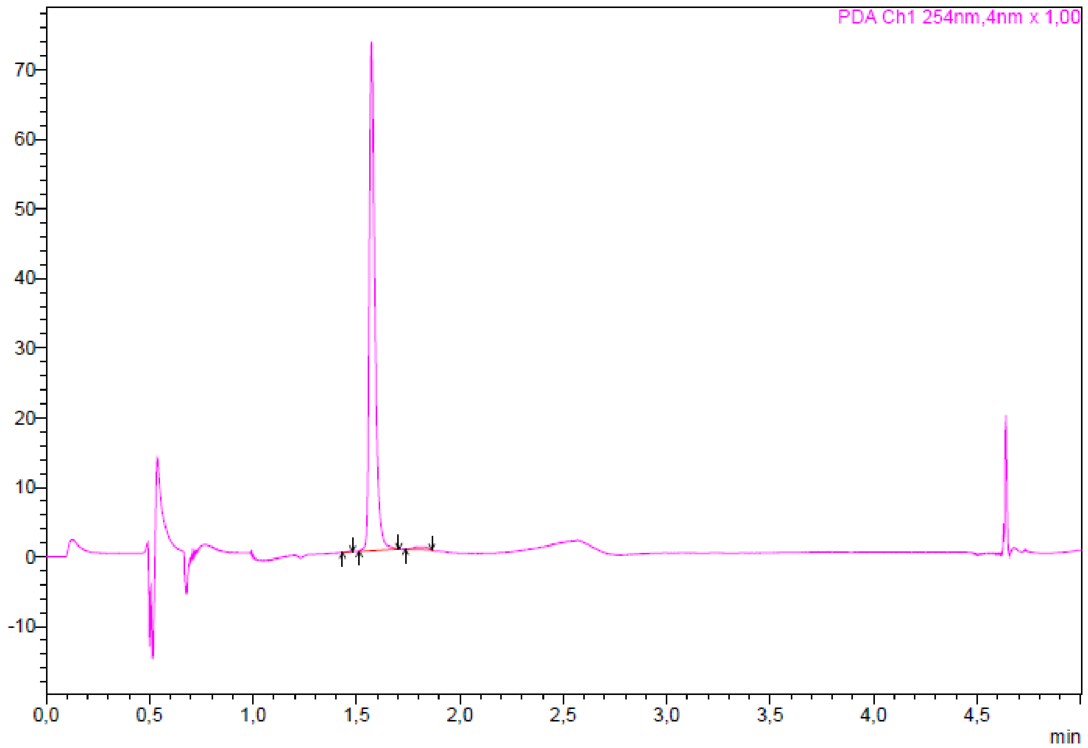

PDA Peak Table(pub-17-re-fr41.lcd)

PDA Ch1 254nm

Peak#	Ret. Time	Height	Area	Area%
1	1.472	32	75	0.055
2	1.573	71643	135243	98.697
3	1.828	383	1711	1.249
Total		72058	137029	100.000

N-methyl-2-(quinolin-3-yl)-1*H*-indole-5-carboxamide (**PUB-37**)

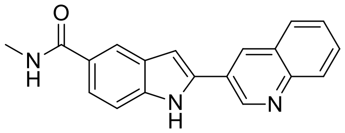

PUB-37

0.210 mg (0.59 mmol) Mesylated compound (**S2Ms**), 99 mg (0.65 mmol) 3-ethynylquinoline, 0.245 mg (1.77 mmol) K_2CO_3, 0.292 mg (1.475 mmol) Na-ascorbate, 49 mg (0.059 sSPhosPd G2, 28 mg (0.177 mmol) $CuSO_4$. After the purification on preparative HPLC we obtained 17 mg (10 %) white powder. Purity: 99.3 % HRMS (ESI^+) *m/z* $[M+H]^+$, calcd. for $C_{19}H_{16}N_3O$: 302.1293, found: 302.1303 ^{1}H NMR (500 MHz, CD_3OD) δ 9.36 (d, *J* = 2.2 Hz, 1H), 8.68 (d, *J* = 2.3 Hz, 1H), 8.16 (d, *J* = 1.6 Hz, 1H), 8.04 (dd, *J* = 21.0, 8.3 Hz, 2H), 7.81 – 7.74 (m, 1H), 7.71 – 7.63 (m, 2H), 7.24 – 7.21 (m, 1H), 2.96 (d, *J* = 1.1 Hz, 3H). ^{13}C NMR (125 MHz, $CDCl_3$) δ 120.75, 119.46, 100.1, 110.50, 28.59.

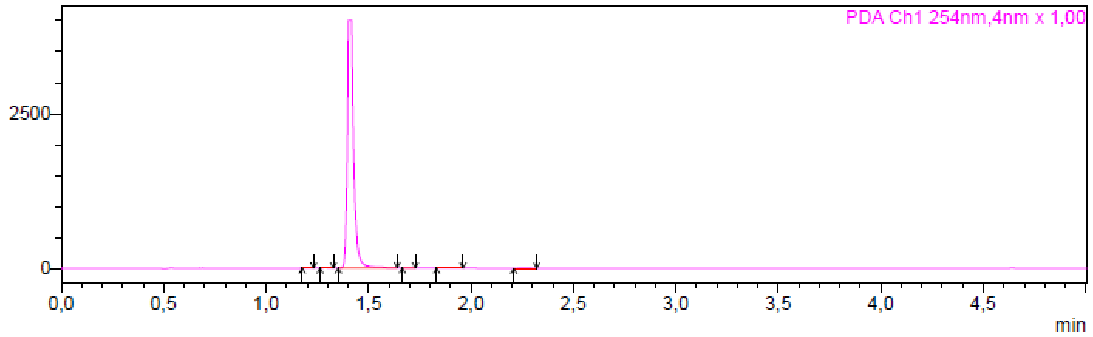

PDA Peak Table(PUB-37-ctrl.lcd)

PDA Ch1 254nm

Peak#	Ret. Time	Height	Area	Area%
1	1,196	5805	9129	0,103
2	1,290	9581	16887	0,191
3	1,409	3998052	8785096	99,398
4	1,690	2506	3032	0,034
5	1,853	2623	8537	0,097
6	2,253	6269	15662	0,177
Total		4024837	8838342	100,000

3. Preparation of on-DNA starting materials

a. Preparation of the HP-AOP-NH2 conjugate

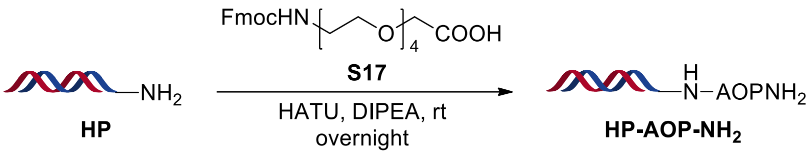

In a 50 mL Falcon tube, to a solution of **HP** (1 µmol, 1 mL in H_2O) was added borate buffer (1 mL, 250 mM, pH 9.4). In a separate vessel, in ice cooling, a solution of 1-(9H-fluoren-9-yl)-3-oxo-2,7,10,13,16- pentaoxa-4-azanonadecan-19-oic acid (**S17**, 40 eq., 200 mM in DMA) was mixed with diisopropyl ethylamine (DIPEA, 80 eq., 200 mM in DMA) and hexafluorophosphate azabenzotriazole tetramethyl uranium (HATU, 40 eq., 200 mM in DMA) and was incubated for 10 minutes. Then, this mixture was added to the solution of the **HP**, vortexed, centrifuged, and then incubated at room temperature overnight. The product was obtained by ethanol precipitation. The DNA pellet was dissolved in 1 mL milli-Q water and piperidine (100 µL) was added. The resulting mixture was incubated at room temperature for 2 hours. The product **HP-AOP-NH2** was obtained by ethanol precipitation.

b. The preparation of 1a–1f, 3a, 7 and S19 by acylation

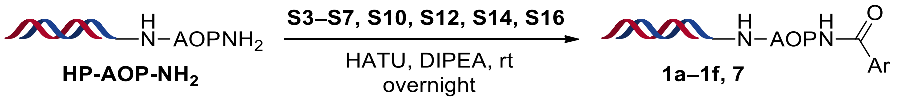

In a 0.5 mL Eppendorf tube, to a solution of **HP-AOP-NH2** (100 nmol, 100 µL in H_2O) was added borate buffer (250 mM, 100 µL, pH 9.4). In a separate vessel, in ice cooling, a solution of the corresponding acid (100 eq., 200 mM in DMA) was mixed with DIPEA (200 eq., 200

mM in DMA) and (HATU, 100 eq., 200 mM in DMA) and was incubated for 10 minutes. Then, this mixture was added to the solution of the **HP-AOP-NH$_2$**, vortexed, centrifuged, and then incubated at room temperature overnight. The products **1a–1f, 3a, 7, and S19** were obtained by ethanol precipitation.

c. General recipe for the iodination of indoles

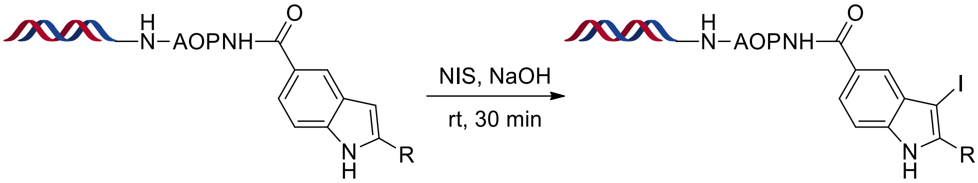

In a 0.5 mL Eppendorf tube, to a solution of on-DNA indole compound (10 nmol, 10 µL in H_2O) was added sodium hydroxide (100 eq., 200 mM in H_2O) and *N*-iodo succinimide (4 eq., 10 mM in DMA) and was incubated at room temperature for 30 minutes. The product was obtained by ethanol precipitation.

4. Optimization of reaction conditions

a. Optimization of the Sonogashira coupling and intramolecular ring-closure

Table S1: Optimization of the Sonogashira coupling sequential ring-closure

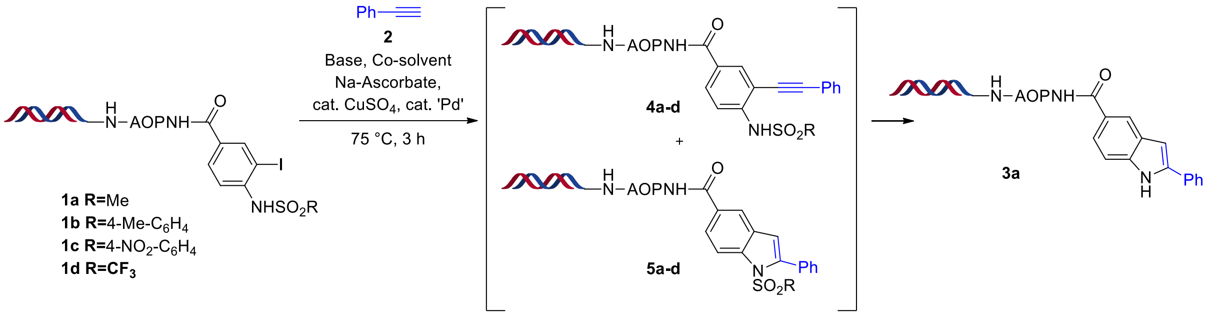

Entry	Co-solvent	Base	Temp. [°C]; time [h]	Catalyst	Yield (3a) [%][d]	Yield (4+5) [%][d]
1	DMAc	K_2CO_3	75; 3	$Pd(OAc)_2$, TPPTS	9	78
2	DMSO				73	0
3	MeCN				9	81
4	DMF				3	93
5	NMP				1	11
6	EtOH				4	37
7	Dioxane				5	61
8	DMSO	Cs_2CO_3			71	0
9		K_3PO_4			74	0
10		DIPEA			0	>99
11		NaOH			73	0
12		K_2CO_3	65; 3		38	37
13			85; 2		73	0
14				$Pd(dppf)Cl_2$	24	2
15				$Pd(PPh_3)_4$	40	0
16				^{t}BuXPhos Pd G3	66	1
17				BrettPhos Pd G3	55	8
18				ssPhos Pd G2	**78**	**0**
19 (**1b**)[b]					22	78
20 (**1c**, 98)[b,c]					52	16
21 (**1d**, 97)[b,c]					2	88

[a]Reaction conditions: **1** (10 nmol, 500 µM in H_2O), **2** (300 eq., 100 mM in co-solvent), base (500 eq., 500 mM in H_2O or co-solvent for DIPEA), Na-ascorbate (25 eq., 50 mM in H_2O), precatalyst (0.6 eq., 1 mM $Pd(OAc)_2$, 10 mM TPPTS, 2 mM $CuSO_4 \cdot 5H_2O$ in H_2O:DMA 9:1) for 3 hours at 75 °C. [b]sSPhos Pd G2 (0.6 eq., 10 mM in H_2O), $CuSO_4 \cdot 5H_2O$ (1.2 eq., 20 mM in H_2O) for 2 hours at 85°C. [c]Conversion in parentheses. [d]Yields were determined by HPLC-MS.

b. General recipe for the Sonogashira coupling and intramolecular ring-closure

In a 0.5 mL Eppendorf tube, to a solution of **1a–f** (10 nmol, 20 µL in H_2O) was added potassium carbonate (500 eq., 500 mM in H_2O), sodium ascorbate (25 eq., 50 mM in H_2O), copper(II) sulfate pentahydrate (1.2 eq., 20 mM in H_2O) and sSPhos Pd G2 (0.6 eq., 10 mM in H_2O) and the corresponding acetylene (300 eq., 100 mM in DMSO). Then, this mixture was vortexed, centrifuged, and incubated at 85 °C for 2 hours. The reaction was quenched with sodium diethyldithiocarbamate (90 eq., 100 mM in H_2O) and incubated at 75 °C for 20 minutes. The product was obtained by ethanol precipitation.

The purity of the starting material was taken into consideration when the yield was calculated based on the following formula:

$$Yield = \frac{HPLC\ UV\ purity\ of\ the\ product\ [\%]}{HPLC\ UV\ purity\ of\ the\ starting\ material\ [\%]}$$

c. Optimization of the Suzuki coupling

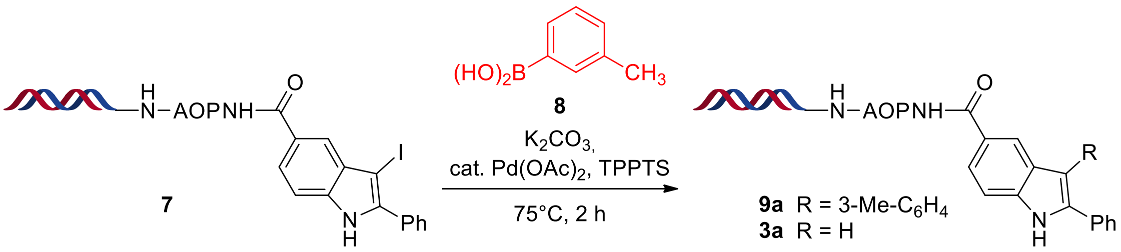

Table S2: Optimization of the Suzuki coupling

<table>
<tr><th>Entry</th><th>Co-solvent</th><th>Base</th><th>Temp. [°C]; time [h]</th><th>Catalyst</th><th>Yield (9a) [%][d]</th><th>Yield (3a) [%][d]</th></tr>
<tr><td>1</td><td>DMSO</td><td rowspan="5">K_2CO_3</td><td rowspan="11">75, 2</td><td rowspan="13">$Pd(OAc)_2$, TPPTS</td><td>17</td><td>63</td></tr>
<tr><td>2</td><td>Dioxane</td><td>60</td><td>25</td></tr>
<tr><td>3</td><td>NMP</td><td>48</td><td>32</td></tr>
<tr><td>4</td><td>DMF</td><td>53</td><td>30</td></tr>
<tr><td>5</td><td>DMA</td><td>55</td><td>26</td></tr>
<tr><td>6</td><td rowspan="12">Dioxane</td><td>KOAc</td><td>59</td><td>24</td></tr>
<tr><td>7</td><td>KF</td><td>60</td><td>24</td></tr>
<tr><td>8</td><td>K_3PO_4</td><td>45</td><td>40</td></tr>
<tr><td>9</td><td>Et_3N</td><td>30</td><td>49</td></tr>
<tr><td>10</td><td>Cs_2CO_3</td><td>49</td><td>36</td></tr>
<tr><td>11</td><td>Na_2CO_3</td><td>57</td><td>27</td></tr>
<tr><td>12</td><td rowspan="6">K_2CO_3</td><td>65, 1</td><td>63</td><td>29</td></tr>
<tr><td>13</td><td>55, 1</td><td>43</td><td>41</td></tr>
<tr><td>14</td><td rowspan="4">65, 1</td><td>$Pd(dppf)Cl_2$</td><td>49</td><td>32</td></tr>
<tr><td>15</td><td>$Pd(PPh_3)_4$</td><td>29</td><td>47</td></tr>
<tr><td>16[b]</td><td rowspan="2">$Pd(OAc)_2$, TPPTS</td><td>58</td><td>35</td></tr>
<tr><td>17[c]</td><td>63</td><td>32</td></tr>
</table>

[a]Reaction conditions: **7** (10 nmol, 500 µM in H_2O), **8** (200 eq., 100 mM in co-solvent), base (500 eq., 500 mM in H_2O or co-solvent for Et_3N), precatalyst (0.6 eq., 1 mM $Pd(OAc)_2$, 10 mM TPPTS in H_2O:DMA 9:1) for 2 hours at 75 °C. [b]0.4 eq. Pd catalyst. [c]0.8 eq. Pd catalyst. [d]Yields were determined by HPLC-MS.

d. General recipe for the Suzuki coupling

In a 0.5 mL Eppendorf tube, to a solution of **7** (10 nmol, 20 µL in H_2O) was added potassium carbonate (500 eq., 500 mM in H_2O), the precatalyst (0.6 eq., 1 mM $Pd(OAc)_2$ and 10 mM TPPTS in H_2O:DMA 9:1) and the corresponding boronic acid (200 eq., 100 mM in dioxane). Then, this mixture was vortexed, centrifuged, and incubated at 65 °C for 1 hour. The reaction was quenched with sodium diethyldithiocarbamate (30 eq., 100 mM in H_2O) and incubated at 75 °C for 20 minutes. The product was obtained by ethanol precipitation.
The purity of the starting material was taken into consideration when the yield was calculated (see page 11, **4/b**).

5. Ligation and qPCR experiments

a. Ligation validation

Based on the recent paper of *Li*, *Zhang*, and *Li*, we performed ligation experiments and qPCR measurements to check that the DNA did not suffer deamination, depurination, or backbone hydrolysis.[4] The **HP-AOP-NH$_2$** underwent ligation with the Primer followed by the Cycle1Tag and Cycle2Tag using the general ligation procedure (see page 3, **1/a/iii**). Compound **S18** was then acylated with **S3** to **S19** according to general procedure **3/b** (see page 10) followed by the Sonogashira coupling and intramolecular ring closure under the optimized conditions resulting in the indole **S20** (see page 11, **4/b**). The crude product was then filtered with a 10kDa Amicon Ultra-0.5 Centrifugal Filter Unit at 14000 rcf for 20 minutes and underwent ligation with the Cycle3Tag leading to **S21**. Compounds **S20** and **S21** were then analyzed by gel electrophoresis, which did not show any apparent damage to the DNA.

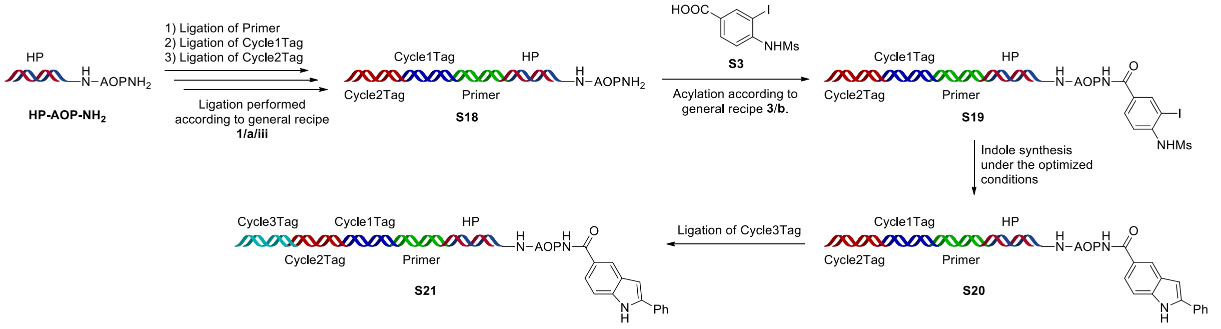

Sequence of the DNA tags:

- Primer: fwd 5'-AAATCGATGTG-3' rev 3'-GGTTTAGCTAC-5'
- Cycle1Tag fwd 5'-AAAGGGAGTAG-3' rev 3'-ACTTTCCCTCA-5'
- Cycle2Tag fwd 5'-CGTTAGTTCGT-3' rev 3'-TCGCAATCAAG-5'
- Cycle3Tag fwd 5'-CGTTAGTTCGT-3' rev 3'-CATGACCAGCT-5'

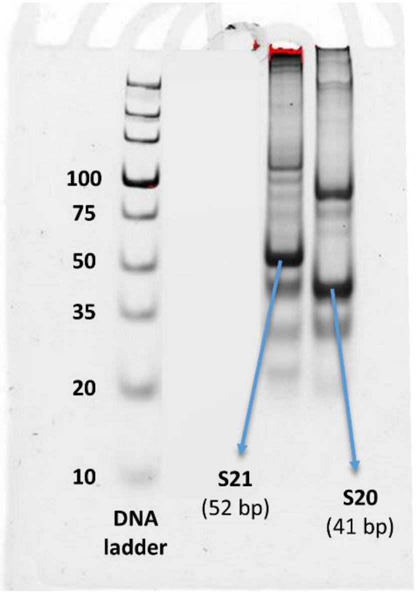

b. qPCR measurement

The DNA tag of compound **S19** was elongated by the Cycle3Tag and the Closing Primer (**S22**) and underwent the Sonogashira coupling and intramolecular ring closure according to the optimized conditions (see page 11, **4/b**) resulting in the indole **S23**. Compound **S22** and **S23** were then analyzed by TapeStation and **S22** was used as reference in the qPCR experiment.

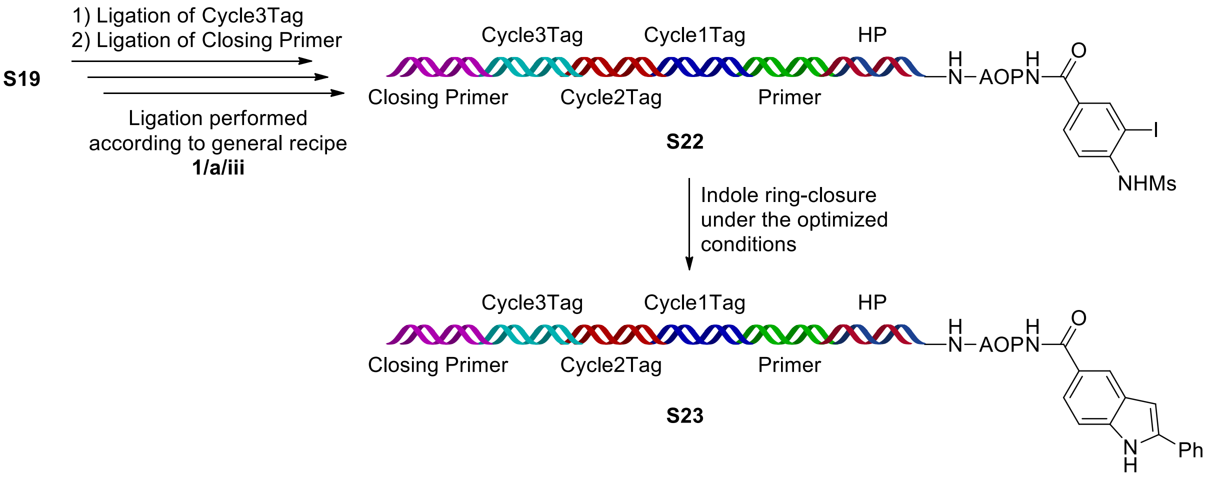

Sequence of Closing Primer:

fwd 5'-CATAGCTAATGAGCAGC-3'

rev 3’-CAGTATCGATTACTCGTCG-5’

i. Total DNA quantification by NanoDrop 1000

The final DNA products of the reference (**S22**) and the target compound (**S23**) were quantified using NanoDrop 1000 (Thermo Fisher Scientific, Waltham, MA, **Table S3**).

ii. Amplifiable DNA quantification with qPCR

The qPCR standard curve was prepared using the amplifiable 79-bp DNA fragment of the reference compound (**S22**) which is structurally similar to the target compound (**S23**). The 79-bp DNA concentration in the reference sample was quantified using Agilent D1000 ScreenTape Assay (Agilent, CA), Agilent D1000 reagents (5067-5583), Agilent D1000 ScreenTape device (5067-5582) in 4200 TapeStation system (Agilent). The sample was diluted to a range of 0.1-50 ng/μL with nuclease-free water according to the manufacturer’s instructions. The reference sample (**S22**) was prepared by 5-fold gradient dilution to five points for the standard curve with the highest concentration of 0.640 μM (**Table S3**). The dilution was obtained with the same nuclease-free water which was used as negative control in qPCR. The diluted samples were quantified by qPCR using SYPBR Green Master Mix (Thermo Fisher Scientific) in Bio-Rad CFX96 (Bio-Rad Laboratories, Hercules, CA) qPCR system according to the following thermal protocol: 50 ^{o}C for 2 min, 95 ^{o}C for 10 min, and 40 cycles of 95 ^{o}C for 15 sec, 60 ^{o}C for 30 sec and 72 ^{o}C for 30 sec. All reactions were performed in three technical parallels. The oligonucleotide sequences of the primers are summarized in **Table S4**. The standard curve was used to calculate the PCR efficiency (E, **Figure S2**). The amplifiable DNA of the target compound was calculated by the ratio of total DNA quantity and DNA quantity by qPCR. The qPCR measurement does not indicate any apparent damage to the DNA.

Table S3. qPCR data for compound S22 and S23

Sample Name	Total DNA quantification by NanoDrop (µM)	79 bp fragment DNA quantification by TapeStation (µM)	CT value	DNA quantification by PCR (µM)	Amplifiable DNA ratio (%)
**qPCR standard S22 (dilution 1)**	0.9200	0.6400	3.81 ± 0.18	-	-
**qPCR standard S22 (dilution 2)**	0.1840	0.1280	6.13 ± 0.21	-	-
**qPCR standard S22 (dilution 3)**	0.0368	0.0256	8.44 ± 0.10	-	-
**qPCR standard S22 (dilution 4)**	0.0074	0.0051	10.92 ± 0.16	-	-
**qPCR standard S22 (dilution 5)**	0.0015	0.0010	13.97 ± 0.14	-	-
**S23**	0.8022	-	8.57 ± 0.29 (25x dilution)	0.6731	83.90

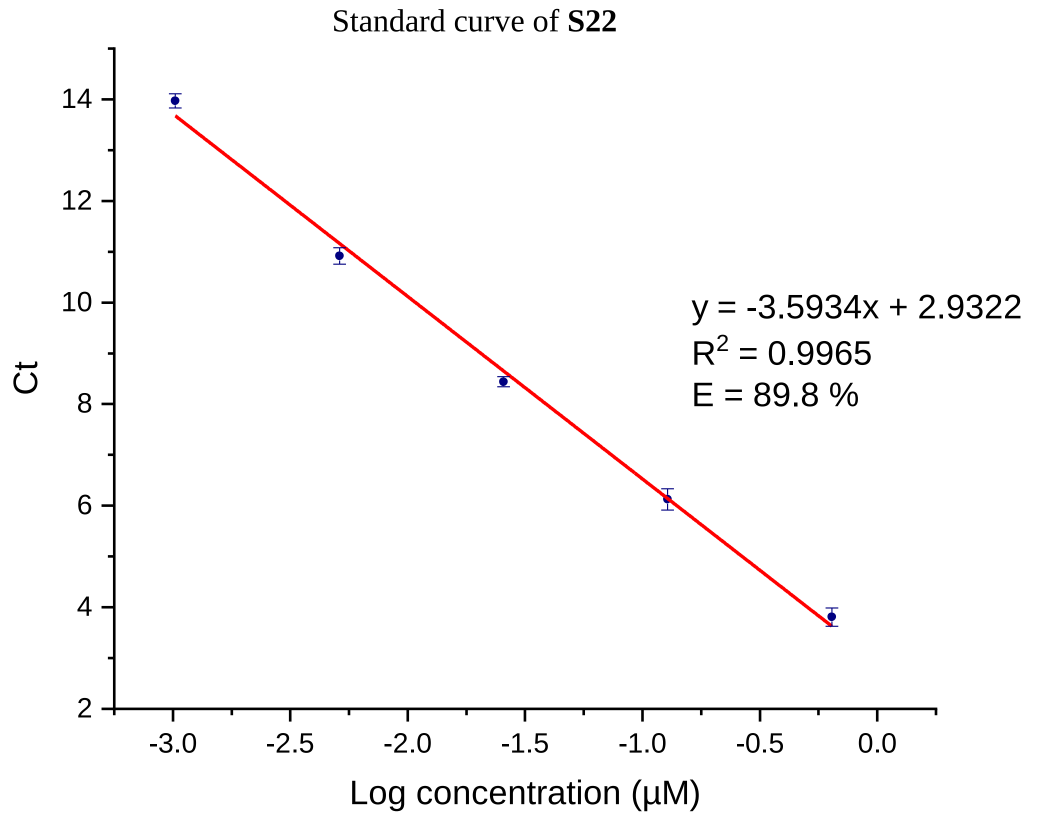

Figure S2. qPCR standard curve of reference compound S22.

Table S4. DNA quantification by NanoDrop and qPCR.

Name	Sequence (5'-->3')
Forward primer	TGACTCCCAAATCGATG
Reverse primer	GCTGCTCATTAGCTATG

6. Affinity screening against Dopamin D_3 receptors

All products of the building block validation of the indole synthesis (**3a, 3c–i, 3k, 3l, 3v, 3w, 3y–aa, 3ad–ag, 3ak**) and the Suzuki coupling (**9a–j, 9w, 9x, 9z**) that resulted in a yield above 50% were selected and their DNA headpiece was elongated with unique PCR amplifiable DNA tags using the general ligation procedure (see page 3, **1/a/iii**). These compounds were mixed manually and used in an affinity-based selection experiment on intact, Dopamine receptor D_3 overexpressed cells according to the method of *Wu and Israel et al*.[4] The best binders were selected for off-DNA synthesis and underwent a competitive binding assay. The main goal of this experiment was to validate the ligation and PCR amplification of compounds that underwent the indole synthesis and Suzuki coupling in a practical biological affinity screening.

a. Preparation of cell line for binding assays

Cells were collected by a cell scraper in ice-cold PBS and centrifuged at 4°C for 15 min, at 2000 g. The pellet was resuspended in a buffer containing 10 mM Tris-HCl and 1 mM EDTA (pH 7.5) and homogenized with a Teflon-glass Braun homogenizer operating at 1500 rpm. The membrane homogenate was centrifuged at 40000 g, at 4°C for 15 min. The final pellet was resuspended in a buffer containing 50 mM NaHepes, 1 mM EDTA, 50 μM 8-hydroxy-quinoline, 0.005% ascorbic acid, and 0.1% bovine serum albumin (pH 7.5, incubation buffer). The membrane preparation was aliquoted and stored at -80°C until use. The protein concentration of the membrane preparations was determined by Bradford method.[5]

b. Affinity selection experiment

The DNA-encoded library solution (10^{10} copies of each library member) was incubated with the cells ($7*10^6$ cells in 700 μL PBS containing 0.1 w/w% NaN_3 and 1 mg/mL sheared salmon sperm DNA), at 37 °C for 1 hour. Then, the cells were washed with 700 μL ice-cold PBS 5 times by gentle centrifugation and resuspension to remove unbound library molecules. The cells were then resuspended in PBS and were heated to 95 °C for 10 min, followed by centrifugation at 14 000 rpm to remove cells. The recovered supernatant was used two more times in the same

protocol for the enrichment of binders. Then, the recovered final supernatant underwent PCR amplification and subsequent Next Generation Sequencing at Delta Bio 2000 Ltd (HU-6726, Szeged, Temesvári krt. 62). The three most enriched compounds were identified as **3y**, **3af**, and **3ak** (Table S5) and they have been synthesized off-DNA as described at ESI pages 9-13 and underwent competitive binding assay to measure their affinity towards Dopamine D_3 receptors.

Table S5. Affinity screening on Dopamin D_3 overexpressed intact cells

Compound	DNA tag	E.F.
3a	GGAGTAATGAGAGTTGAGCTGTACTGGTCGAGA	<1
3c	GGAGTAATGAGGCTTACAGAGTACTGGTCGAGA	<1
3d	GGAGTAATGAGGTCCTAAGTGTACTGGTCGAGA	2.0
3e	GGAGTAATGAGGTACAGTAGGTACTGGTCGAGA	<1
3f	GGAGTAATGAGTTCAAGGACGTACTGGTCGAGA	<1
3g	GGAGTAATGAGCCTTCATACGTACTGGTCGAGA	<1
3h	GGAGTAATGAGGTTGCTCATGTACTGGTCGAGA	<1
3i	GGAGTAATGAGCTCAGCTTAGTACTGGTCGAGA	<1
3k	GGAGTAATGAGCACCATCAAGTACTGGTCGAGA	1.1
3l	GGAGTAATGAGCATCTCCAAGTACTGGTCGAGA	2.4
3v	GGAGTAATGAGAGTTTCCGCGTACTGGTCGAGA	<1
3w	GGAGTAATGAGTGTGCATTGGTACTGGTCGAGA	<1
**3y**	**GGAGTAATGAGAGGGAAATGGTACTGGTCGAGA**	**5.5**
3z	GGAGTAATGAGAACGACTGAGTACTGGTCGAGA	<1
3aa	GGAGTAATGAGTTCCTGTGTGTACTGGTCGAGA	<1
3ad	GGAGTAATGAGCATCCCAAGGTACTGGTCGAGA	<1
3ae	GGAGTAATGAGCGTTAGTTCGTACTGGTCGAGA	<1
**3af**	**GGAGTAATGAGAATCATCCGGTACTGGTCGAGA**	**7.1**
3ag	GGAGTAATGAGGAGGATTCCGTACTGGTCGAGA	<1
**3ak**	**GGAGTAATGAGTCAGGCATTGTACTGGTCGAGA**	**5.1**
9a	GGAGTAATGAGAGTTGAGCTGTACTGGTCGAGA	<1
9b	CGGAATGATAGGAGGAGTATGTACTGGTCGAGA	<1
9c	CGGAATGATAGCTCTACTGCGTACTGGTCGAGA	<1
9d	CGGAATGATAGGAATACCCAGTACTGGTCGAGA	<1
9e	CGGAATGATAGTCGTCTGTAGTACTGGTCGAGA	1.8
9f	CGGAATGATAGCGCAGCTATGTACTGGTCGAGA	<1
9g	CGGAATGATAGTGTCCCAAAGTACTGGTCGAGA	2.3
9h	CGGAATGATAGAATCCCTTGGTACTGGTCGAGA	3.5
9i	CGGAATGATAGACTACCTGTGTACTGGTCGAGA	<1
9j	CGGAATGATAGAGATGCGAAGTACTGGTCGAGA	<1
9w	CGGAATGATAGTGTGCATTGGTACTGGTCGAGA	<1
9x	CGGAATGATAGAACGACTGAGTACTGGTCGAGA	<1
9z	CGGAATGATAGAGGGAAATGGTACTGGTCGAGA	<1

E.F. : Enrichment factor; E.F. = (copy number of the molecule/total reads)/(real number of the molecule/library size)

c. Competitive binding experiment

In D3R displacement aliquots of frozen CHO-hD3 cell line membrane homogenates (10 μg of protein) were thawed and suspended in the incubation buffer (described above). Membranes were incubated in the presence of the unlabelled ligands in increasing concentrations (10^{-10} – 10^{-3} M) at 25˚C for 1 h with [^{3}H]7-OH-DPAT according to Levesque et al.[6] The non-specific and total binding was determined in the presence and absence of 10 μM unlabelled 7-OH-DPAT. The reaction was terminated by rapid filtration under vacuum (Brandel M24R Cell Harvester), and washed three times with 5 mL ice-cold buffer through Whatman GF/C glass fibers coated with 0.1% bovine serum albumin. The radioactivity of the dried filters was detected using UltimaGold™ MV aqueous scintillation cocktail with Packard Tricarb 2300TR liquid scintillation counter. The competitive binding assays were performed in duplicate and repeated at least three times.

d. Data analysis

Experimental data were presented as means ± S.E.M. Points were fitted with the professional curve fitting program, GraphPad Prism 5.0 (GraphPad Prism Software Inc., San Diego, CA), using non-linear regression analysis. In the competition binding assay, the 'One site competition' fitting was used to establish the equilibrium binding affinity (K_i value).

e. Results

Dopamine receptor binding affinities of the analogues were examined in [^{3}H]7-OH-DPAT homologous displacement experiments for D3R in CHO-hD3 cell line membrane homogenates. All derivatives exhibited lower binding affinity in the dopamine receptor system than the selective ligand 7-OH-DPAT (Fig. 1), analogues displayed micromolar affinities.

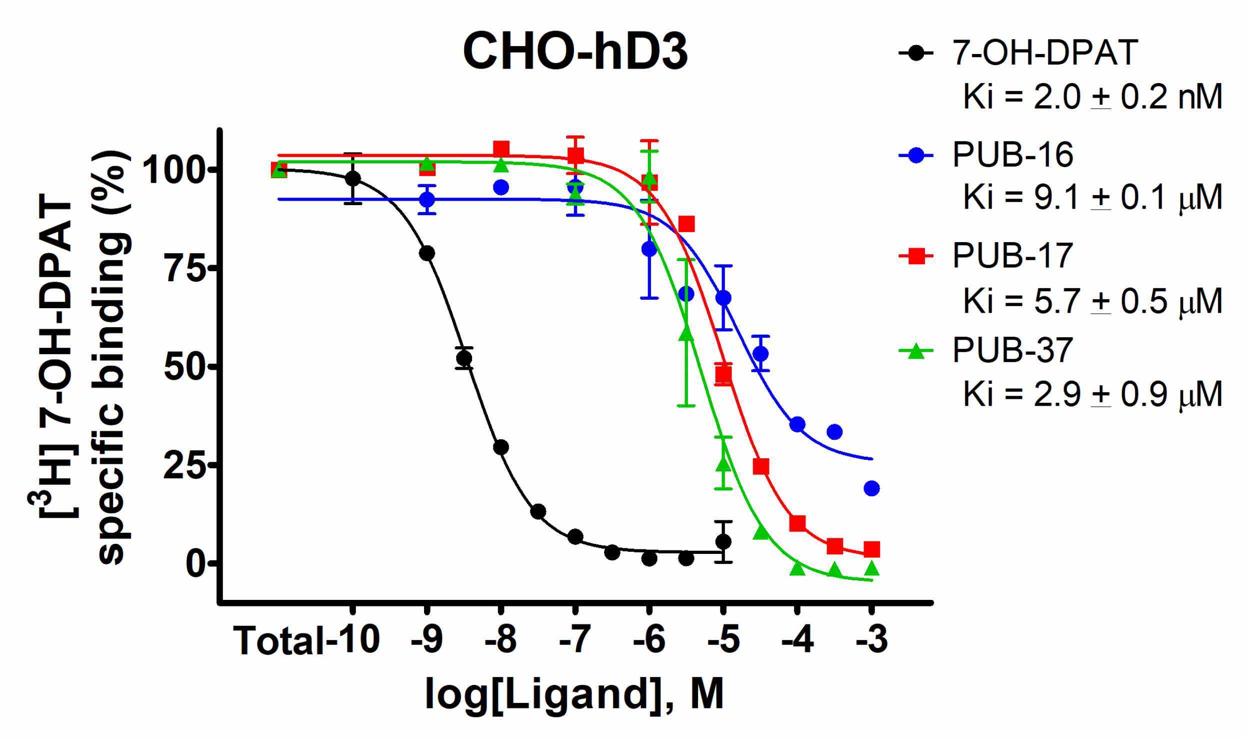

Figure S3. D3R binding affinity of PUB analogues.

The binding affinity of ligands was compared to 7-OH-DPAT in $[^3H]$7-OH-DPAT competition binding assays in CHO cells expressing hD3 membrane homogenates. Values represent mean values ± S.E.M. for at least three experiments performed in duplicate. The IC_{50} values for the D3R according to the competition binding curves were converted into equilibrium inhibitory constant (K_i) values, using the Cheng-Prusoff equation.[7]

7. Synthesis of the mock library

Compound **1a** was reacted with acetylene building blocks **BB1a** and **BB1b** (according to the procedure described on page 11, **4/b**). Subsequent mixing and iodination at the 3rd position (according to the procedure described on page 10, **3/c**) yielded compounds **S24** and **S25**. Suzuki coupling with three different boronic acids **BB2a-c** was performed (according to the procedure described on page 13, **4/d**) and all expected products (**S26–31**) were detected by HPLC-MS.

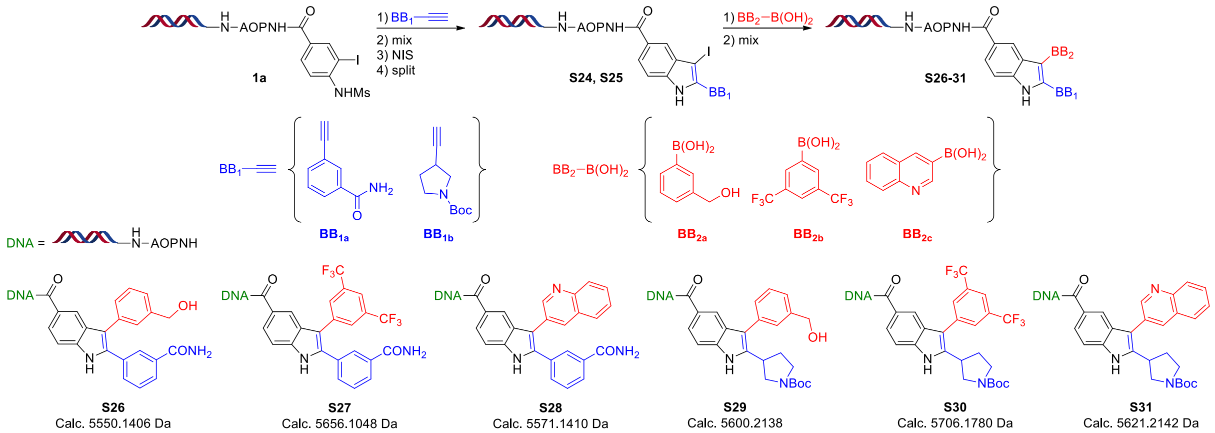

The synthesis steps were followed by HPLC-MS:

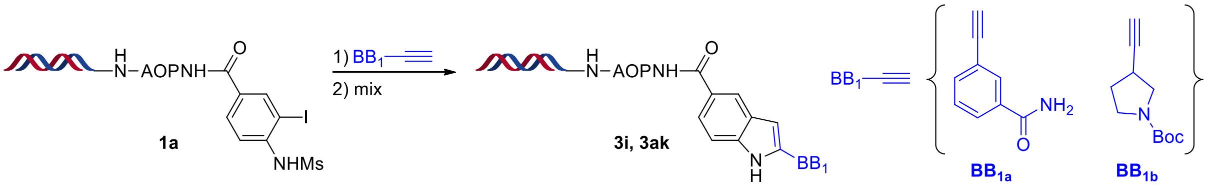

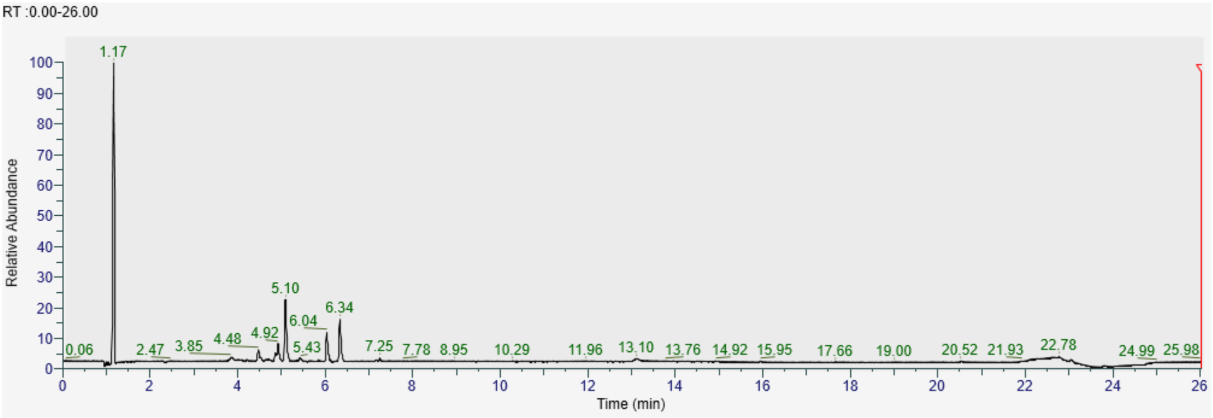

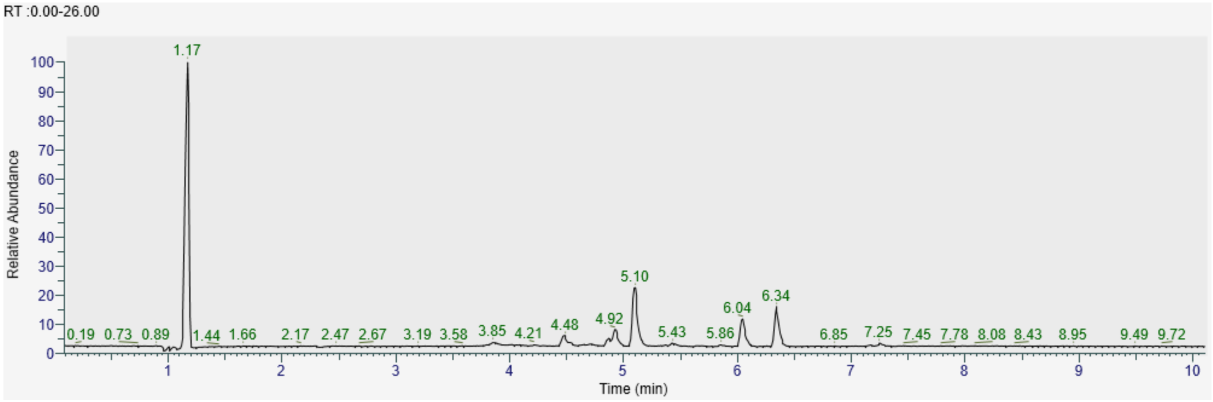

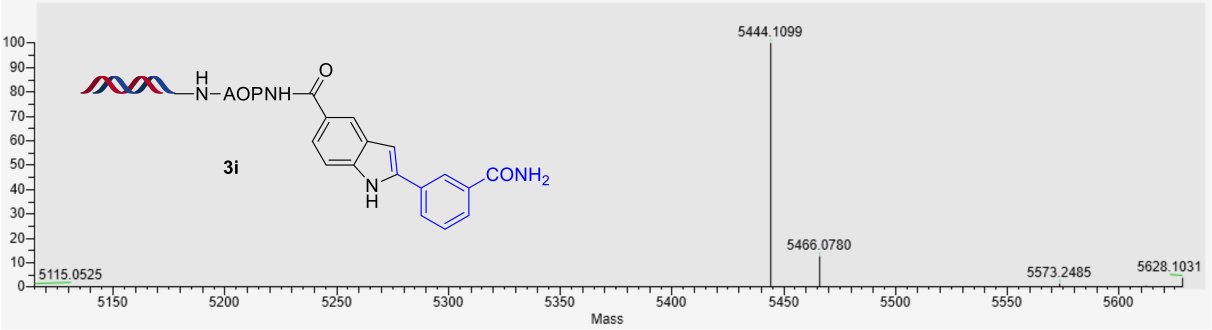

Retention time of **3i**: 5.10 min

Calcd. for $C_{181}H_{246}N_{55}O_{108}P_{17}$ 5444.0988; found 5444.1099

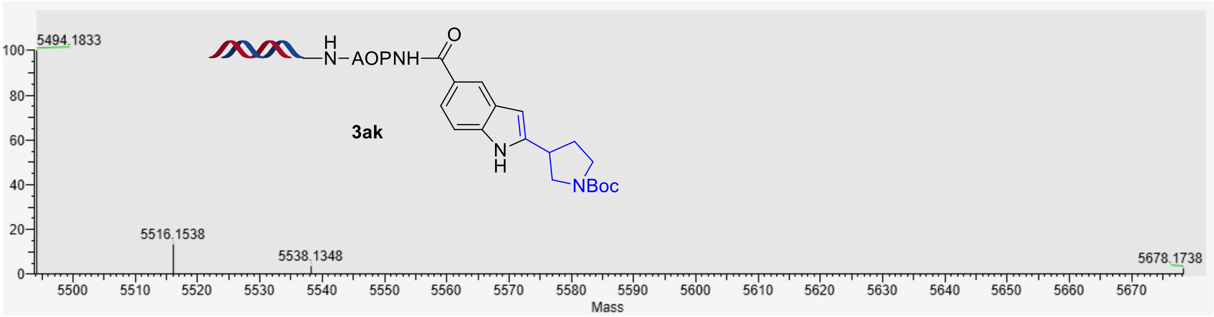

Retention time of **3ak**: 6.04 min

Calcd. for $C_{183}H_{256}N_{55}O_{109}P_{17}$ 5494.1720; found 5494.1833

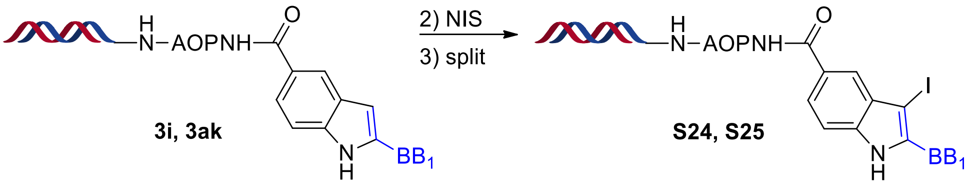

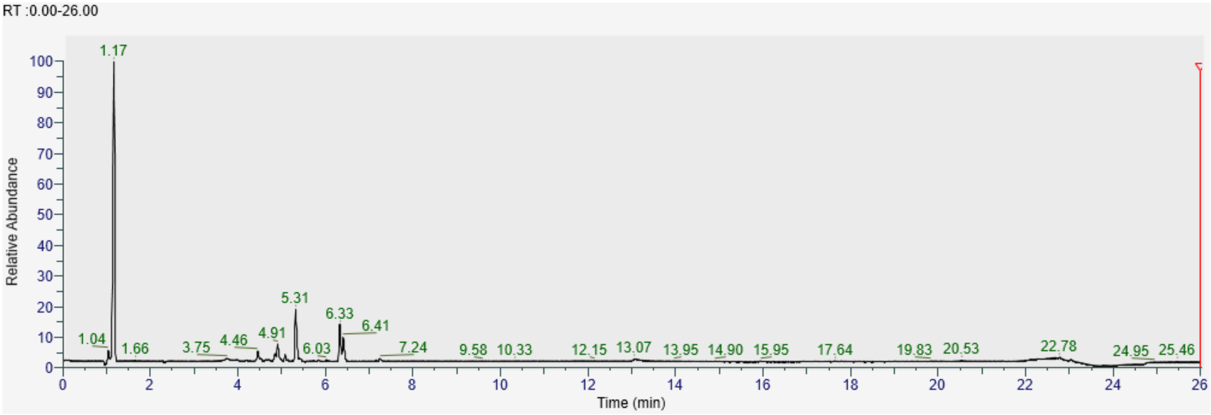

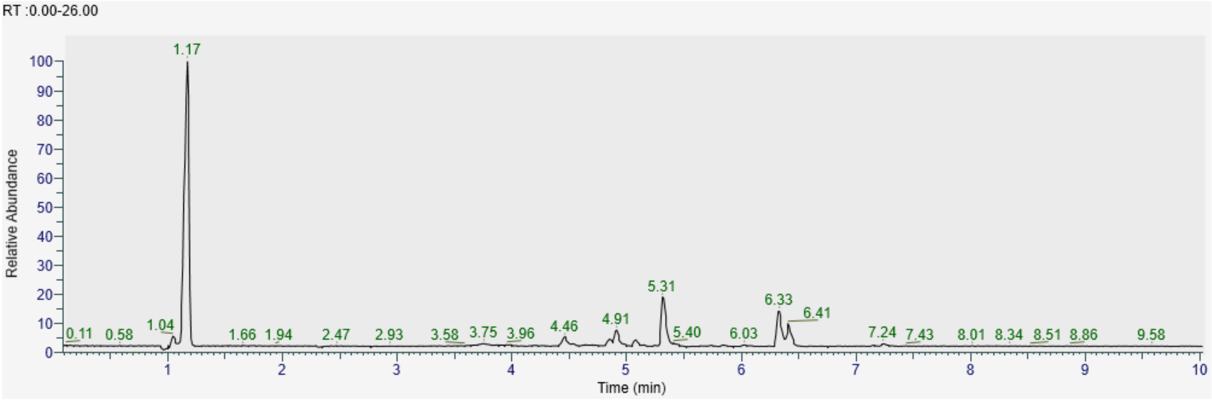

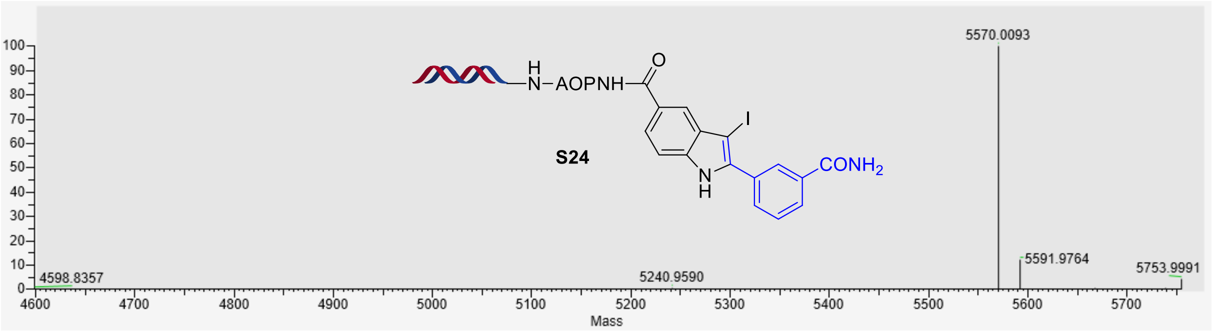

Retention time of **S24**: 5.31 min

Calcd. for $C_{181}H_{245}N_{55}O_{108}P_{17}I$ 5569.9955; found 5570.0093

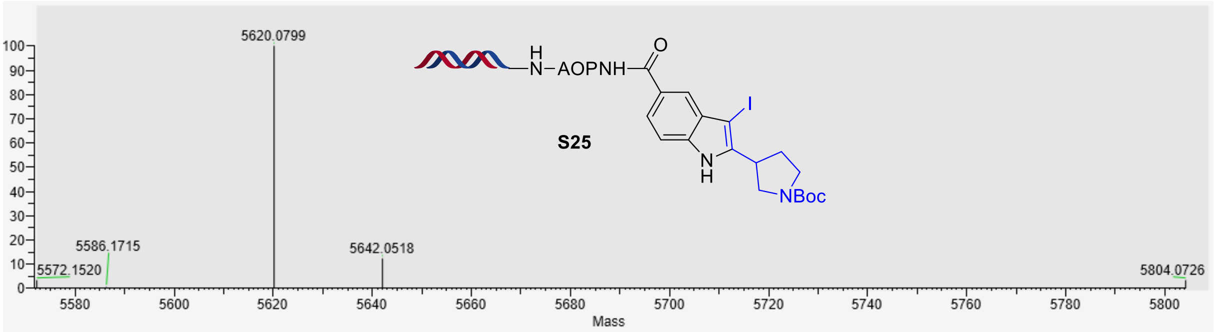

Retention time of **S25**: 6.41 min

Calcd. for $C_{183}H_{255}N_{55}O_{109}P_{17}I$ 5620.0686; found 5620.0799

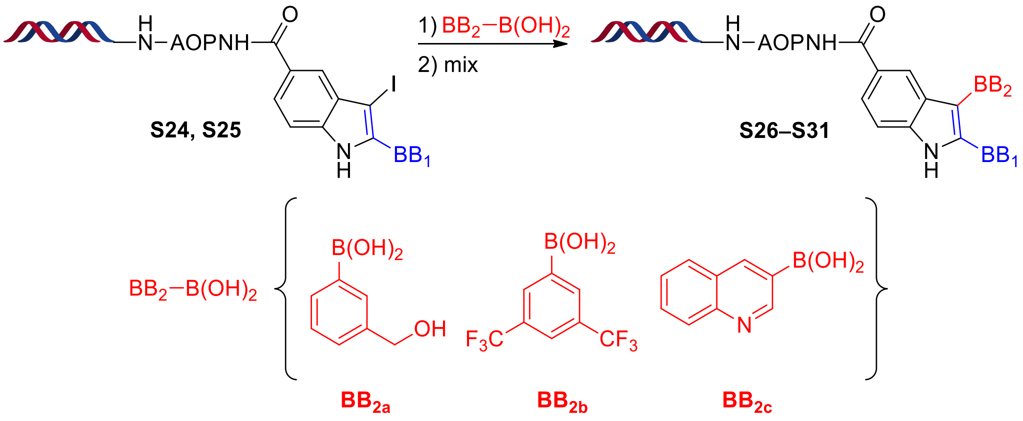

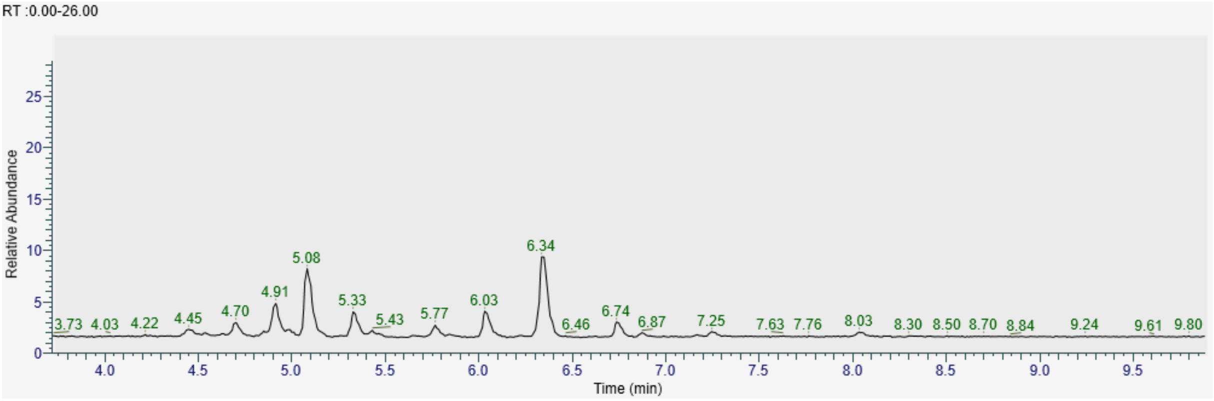

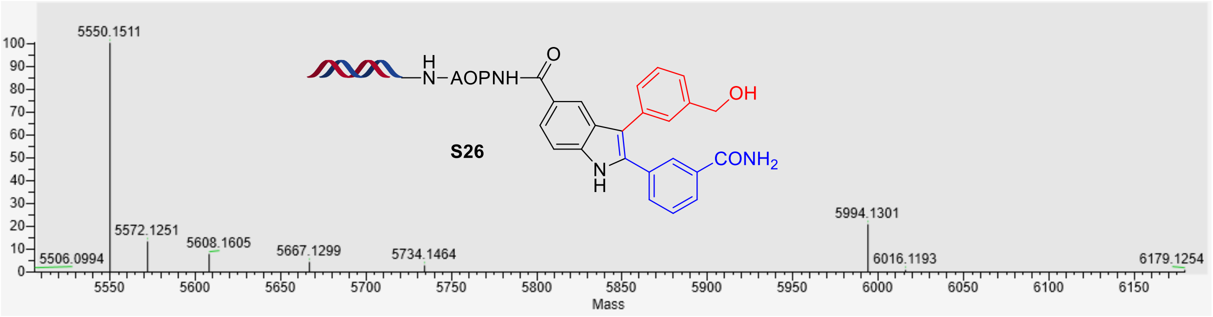

Retention time of **S26**: 5.33 min

Calcd. for $C_{188}H_{252}N_{55}O_{109}P_{17}$ 5550.1406; found 5550.1511

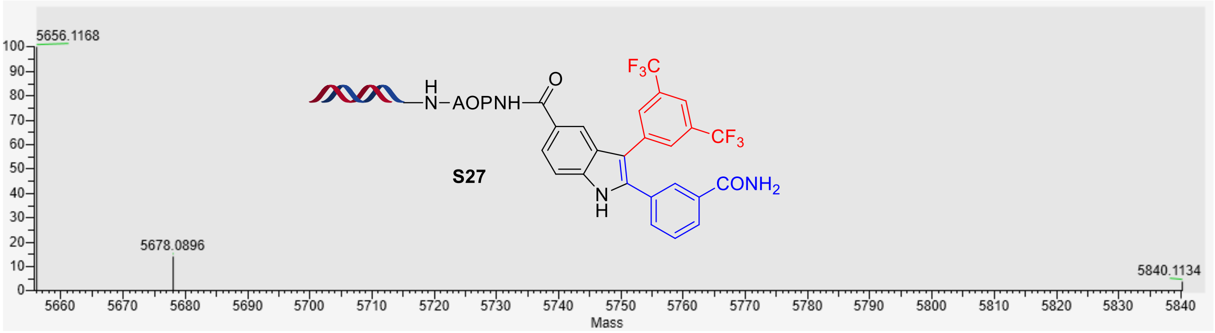

Retention time of **S27**: 6.74 min

Calcd. for $C_{189}H_{248}N_{55}O_{108}P_{17}F_6$ 5656.1049; found 5656.1168

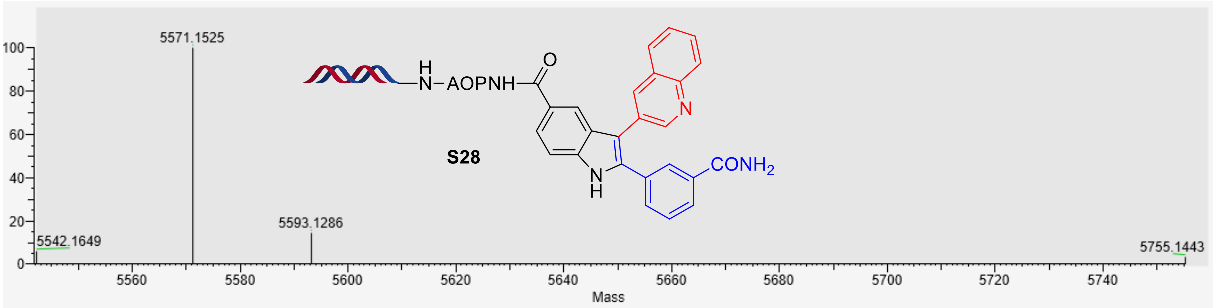

Retention time of **S28**: 5.77 min

Calcd. for $C_{190}H_{251}N_{56}O_{108}P_{17}$ 5571.1410; found 5571.1525

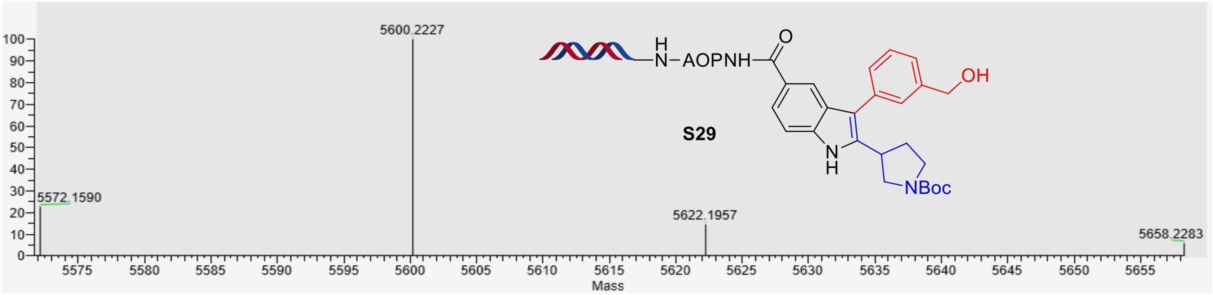

Retention time of **S29**: 6.30 min

Calcd. for $C_{190}H_{262}N_{55}O_{110}P_{17}$ 5600.2138; found 5600.2227

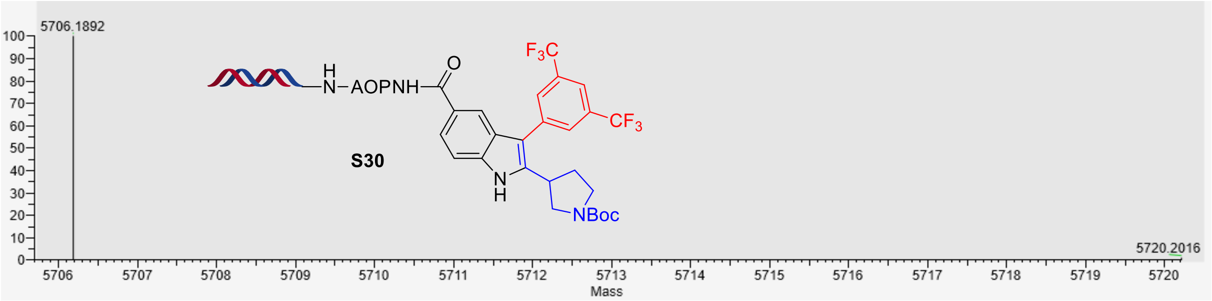

Retention time of **S30**: 8.03 min

Calcd. for $C_{191}H_{258}N_{55}O_{109}P_{17}F_6$ 5706.1780; found 5706.1892

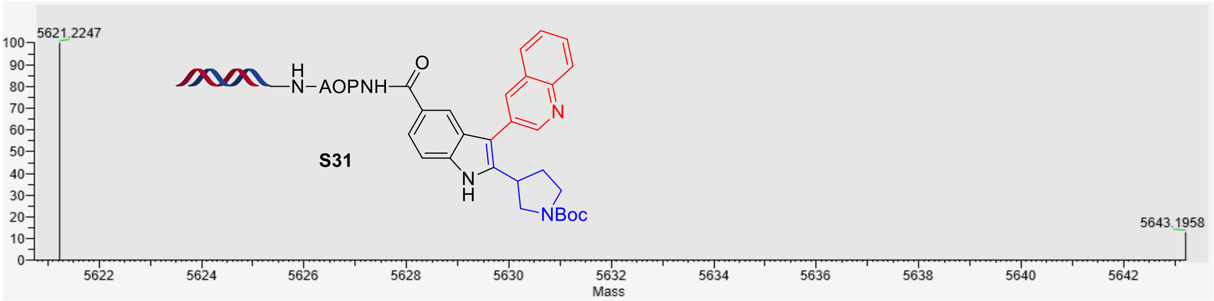

Retention time of **S31**: 6.87 min

Calcd. for $C_{192}H_{261}N_{56}O_{109}P_{17}$ 5621.2141; found 5621.2247

8. Control reactions

We performed control reactions to validate the structure of the indole prepared through the Sonogashira and intramolecular ring closure cascade.

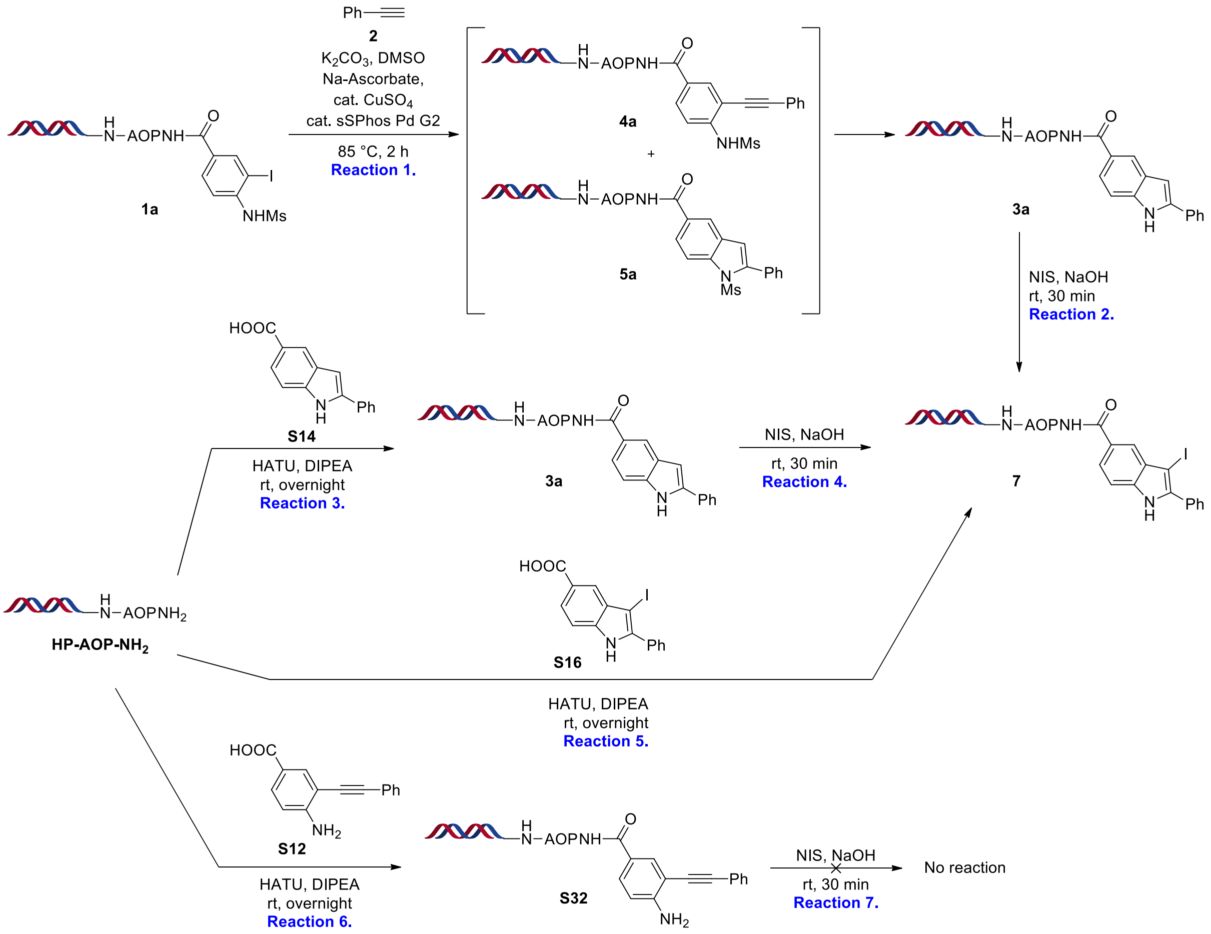

a. HPLC-MS spectra of the control reactions

Reaction 1.

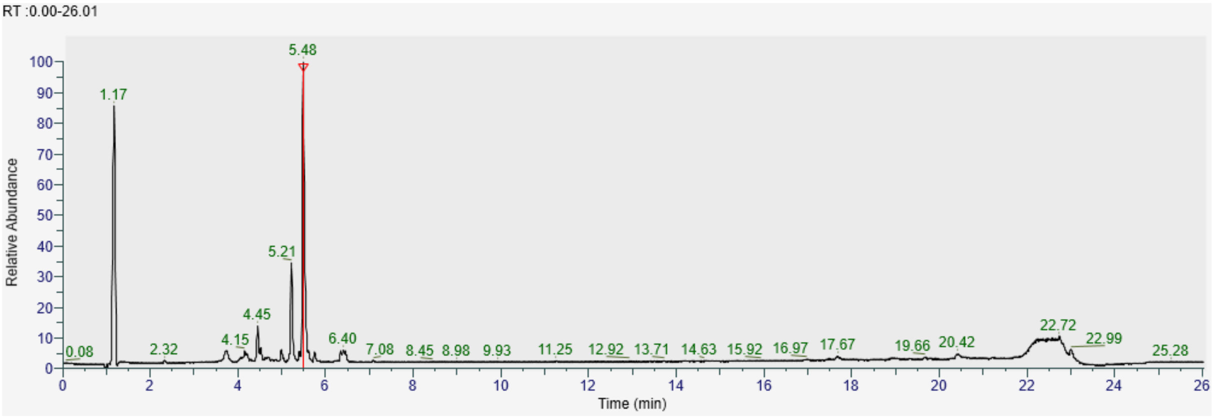

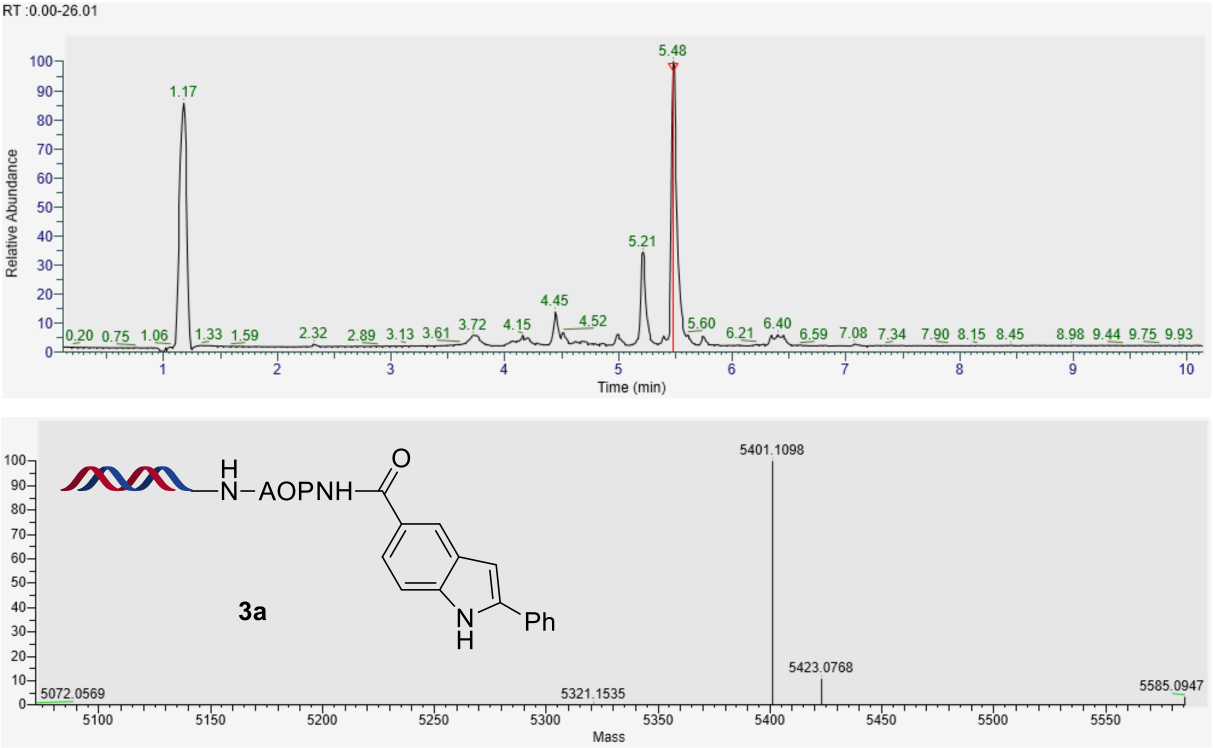

Retention time of **3a**: 5.48 min

Calcd. for $C_{180}H_{245}N_{54}O_{107}P_{17}$ 5401.0930; found 5401.1098

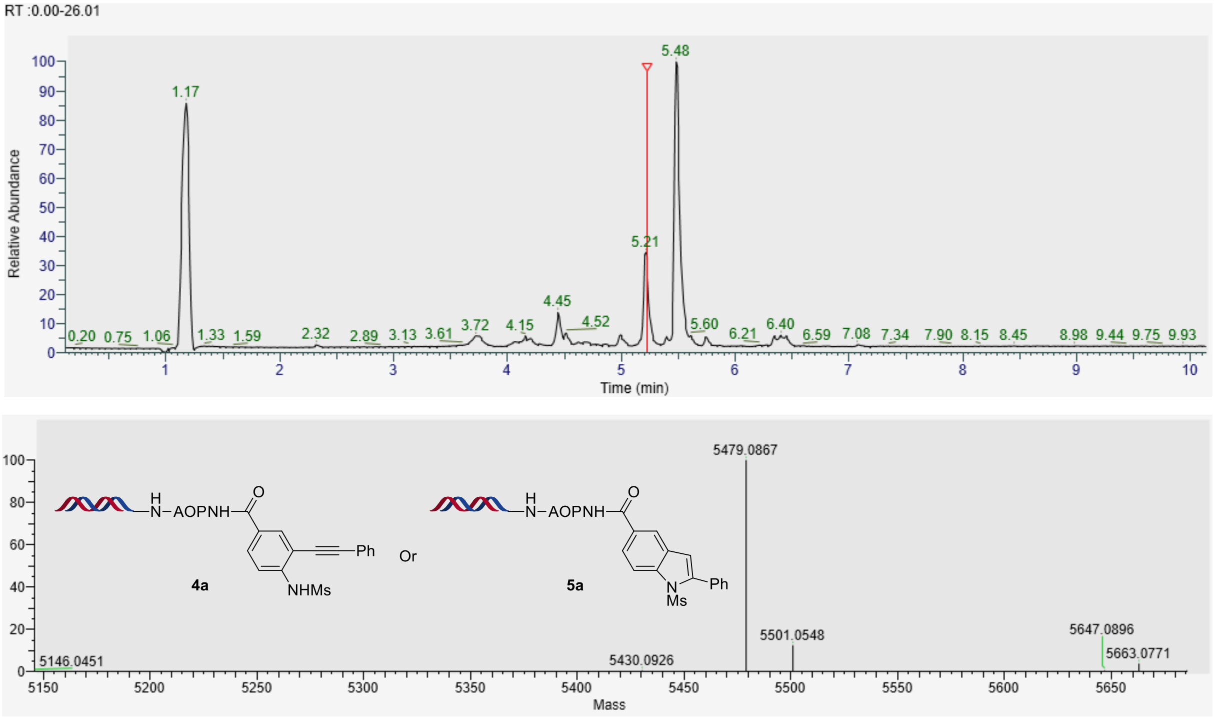

Retention time of **4a** or **5a**: 5.21 min

Calcd. for $C_{181}H_{247}N_{54}O_{109}P_{17}S$ 5479.0705; found 5479.0867

Based on the recent paper of *Li*, *Zhang*, and *Li*, we checked the HPLC-MS spectra of the synthesis of **3a** looking for typical DNA damage types of deamination, depurination, and backbone hydrolysis.[8] Besides the expected product **3a** and the known intermediate **4a** or **5a**, the other components remain unidentified side-products, not being correspondent with the known DNA damage types. The list of components found in the crude HPLC-MS spectra is as follows:

Retention time: 3.72 min; found mass: 4976.8993; unidentified side-product

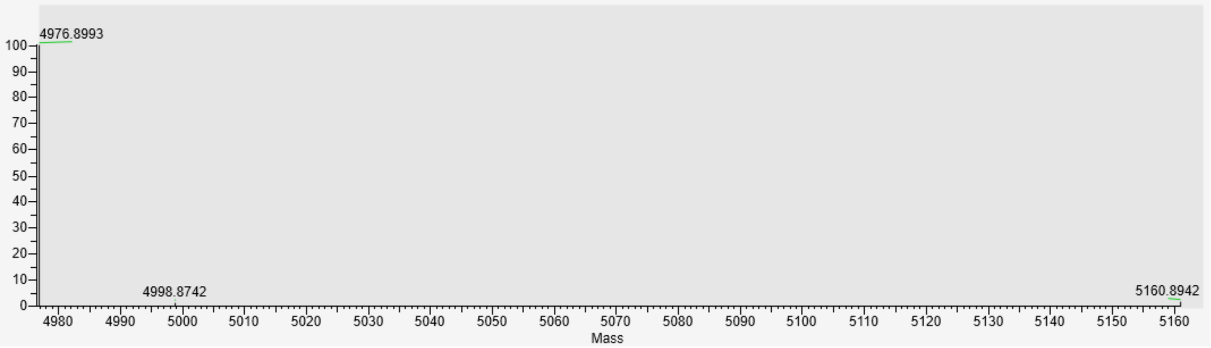

Retention time: 4.15 min; found mass: 4988.8991; unidentified side-product

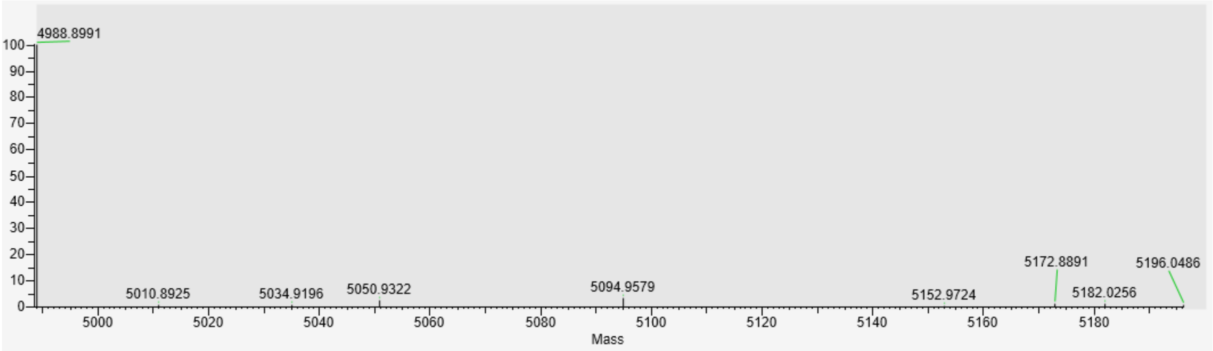

Retention time: 4.45 min; found mass: 5224.0506; unidentified side-product

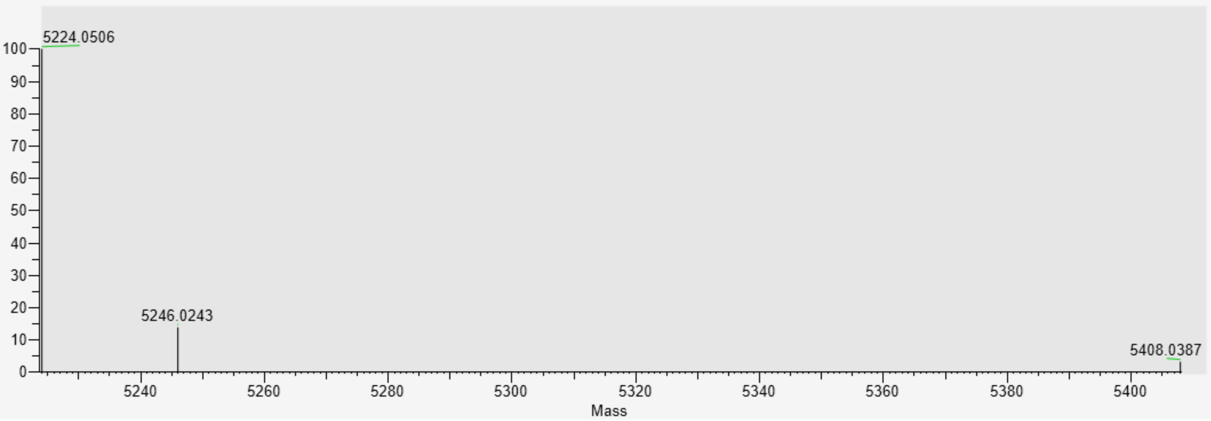

Retention time: 4.52 min; found mass: 5224.0420; unidentified side-product

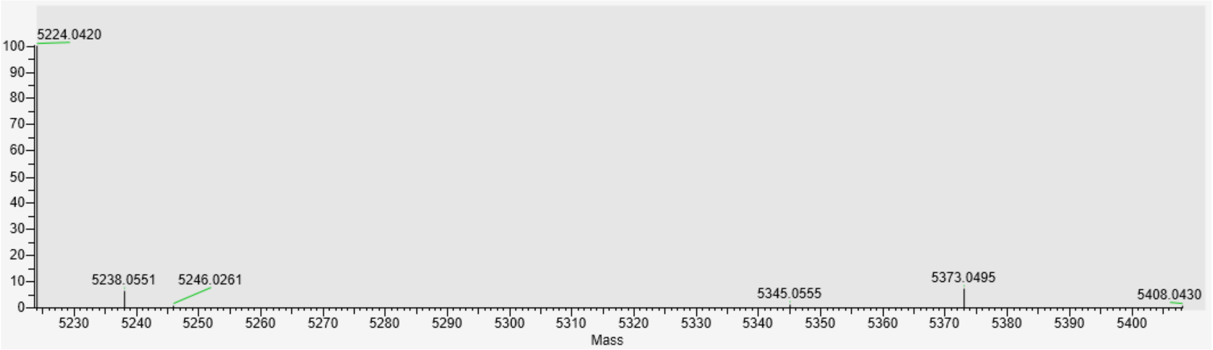

Retention time: 4.99 min; found mass: 5458.1581, 5433.0915, 5480.1277; unidentified side-products

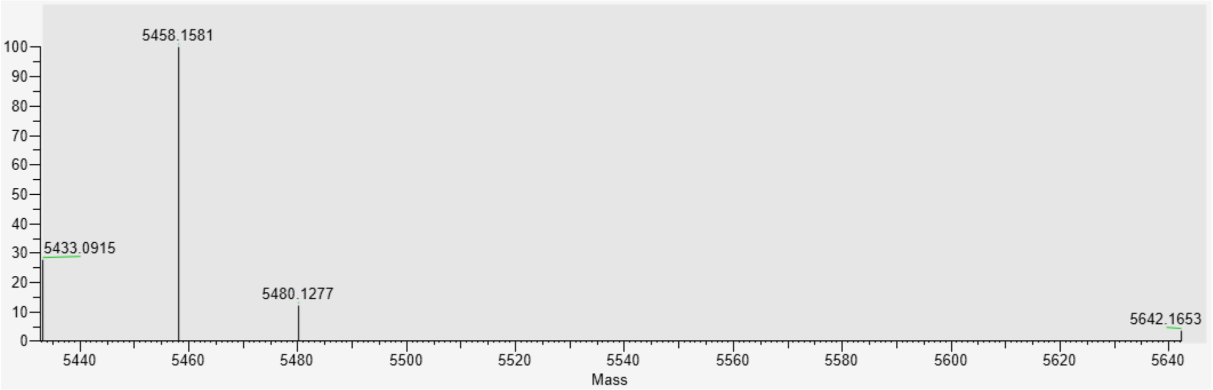

Retention time: 5.74 min; found mass: 5479.0802; unidentified side-product

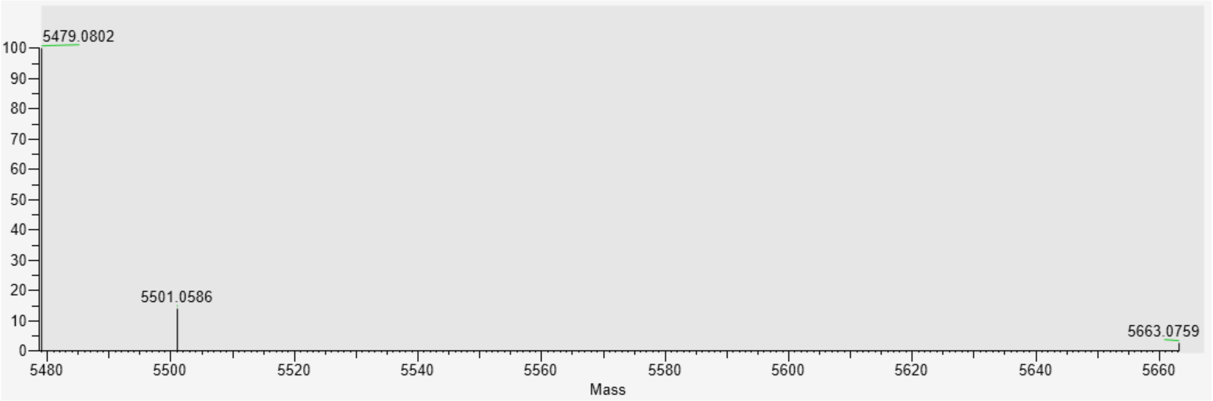

Retention time: 6.40 min; found mass: 5581.1278; unidentified side-product

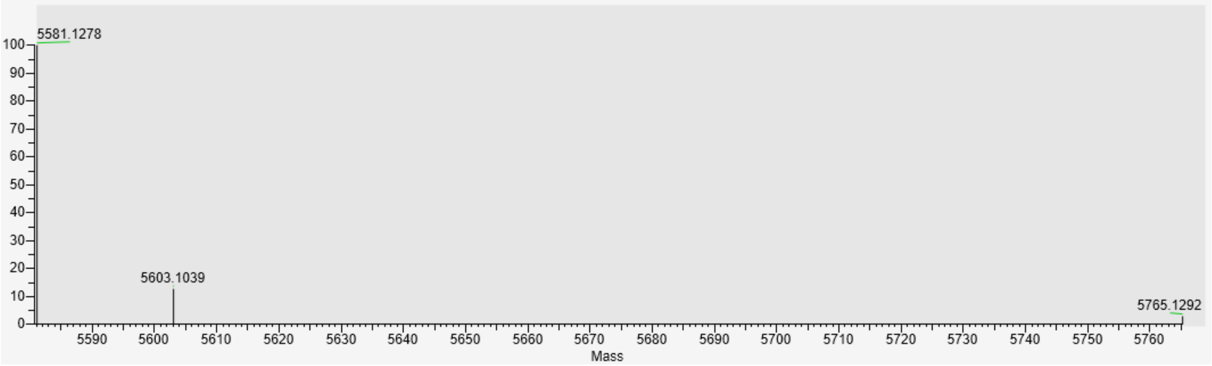

Reaction 2.

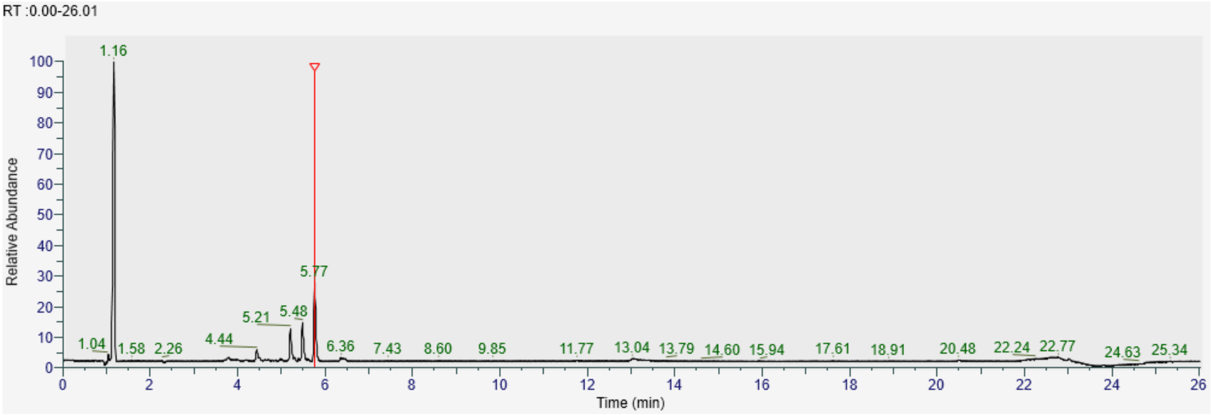

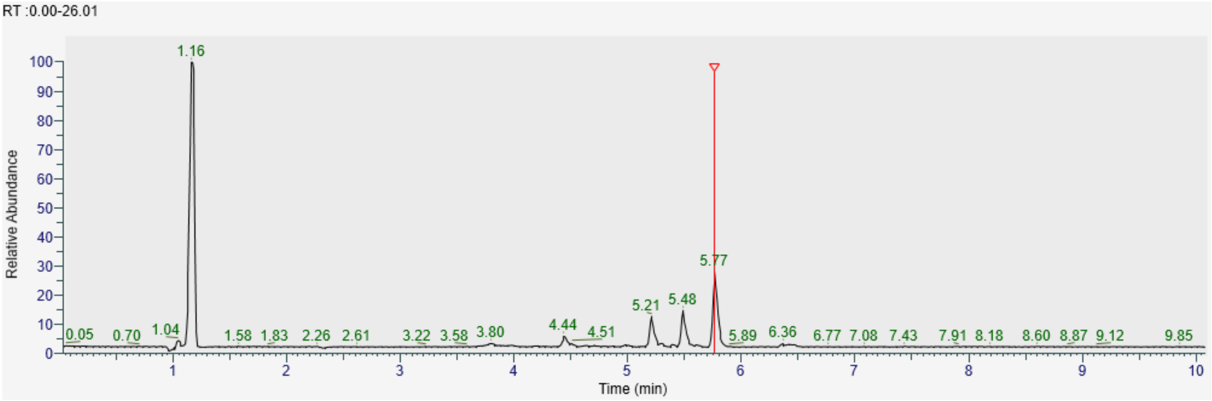

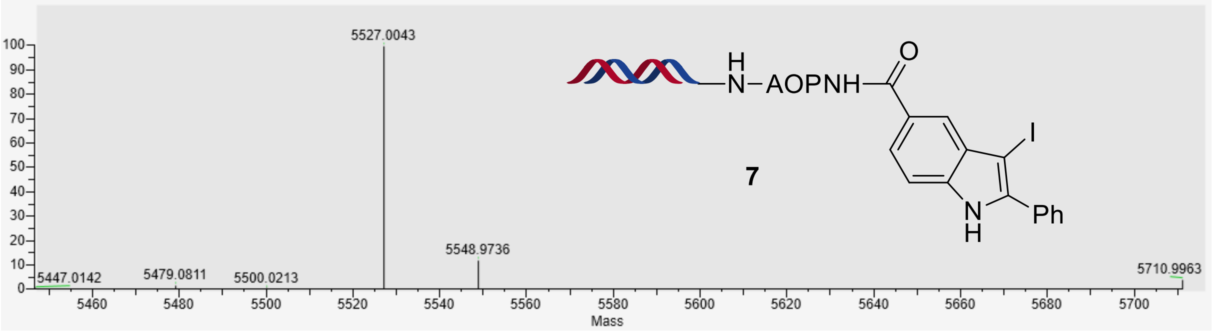

Retention time of **7**: 5.77 min

Calcd. for $C_{180}H_{244}N_{54}O_{107}P_{17}I$ 5526.9896; found 5527.0043

Reaction 3.

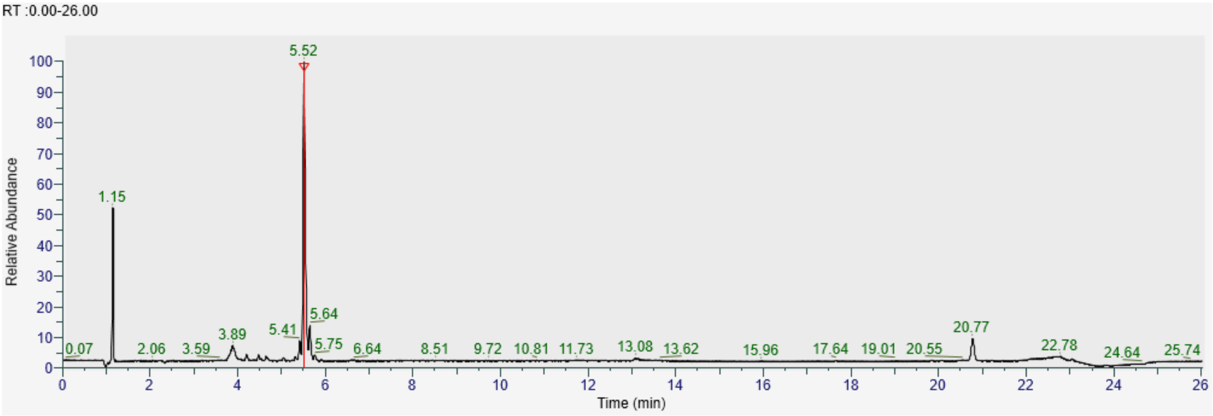

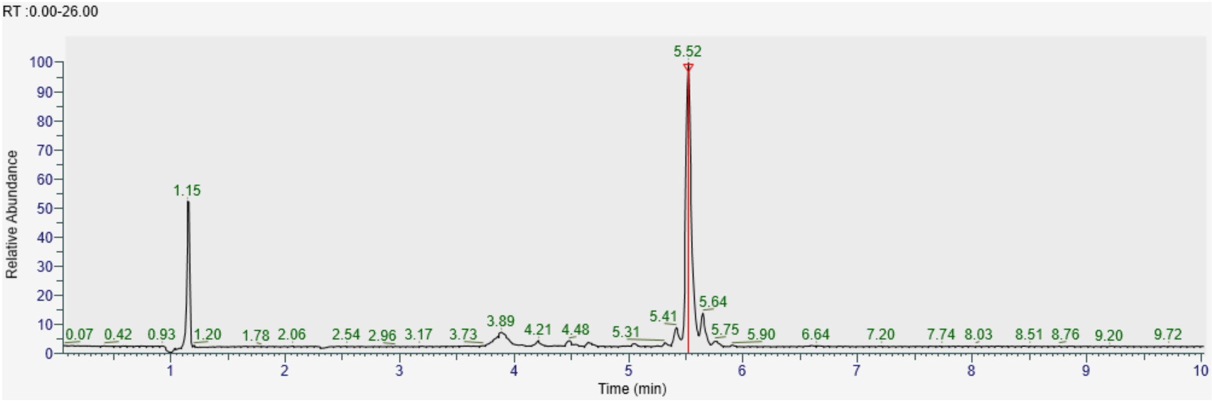

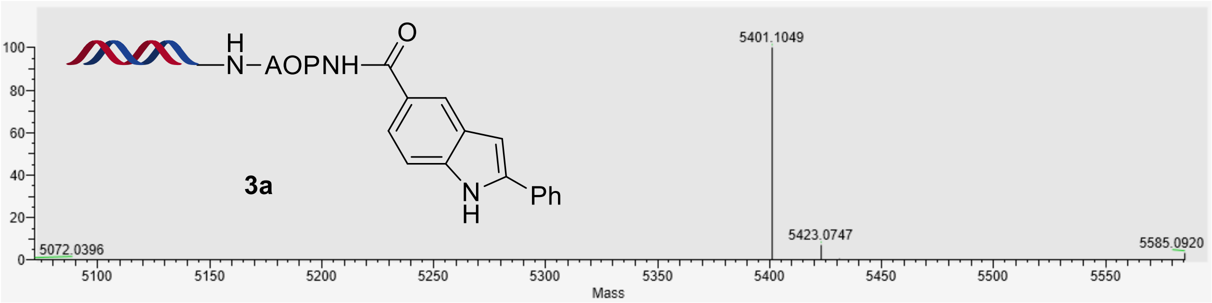

Retention time: 5.52 min

Calcd. for $C_{180}H_{245}N_{54}O_{107}P_{17}$ 5401.0930; found 5401.1049

Reaction 4.

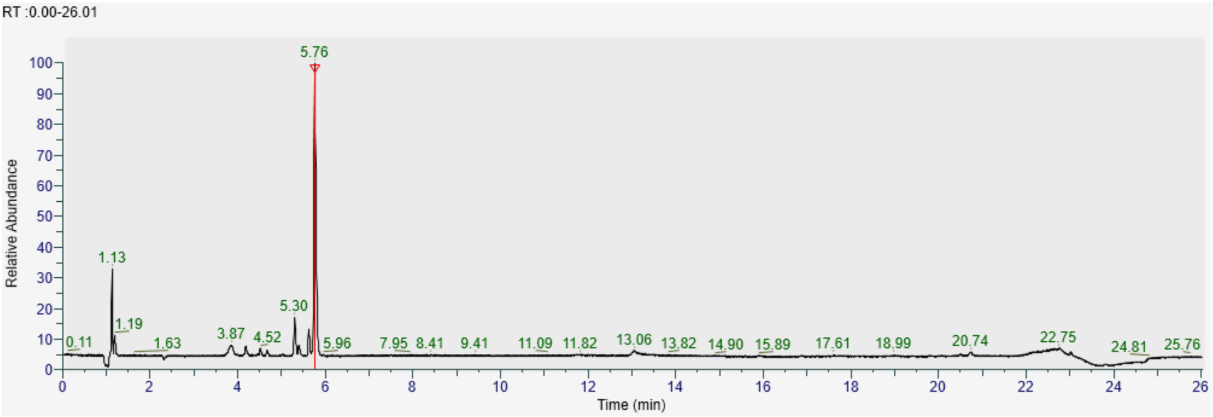

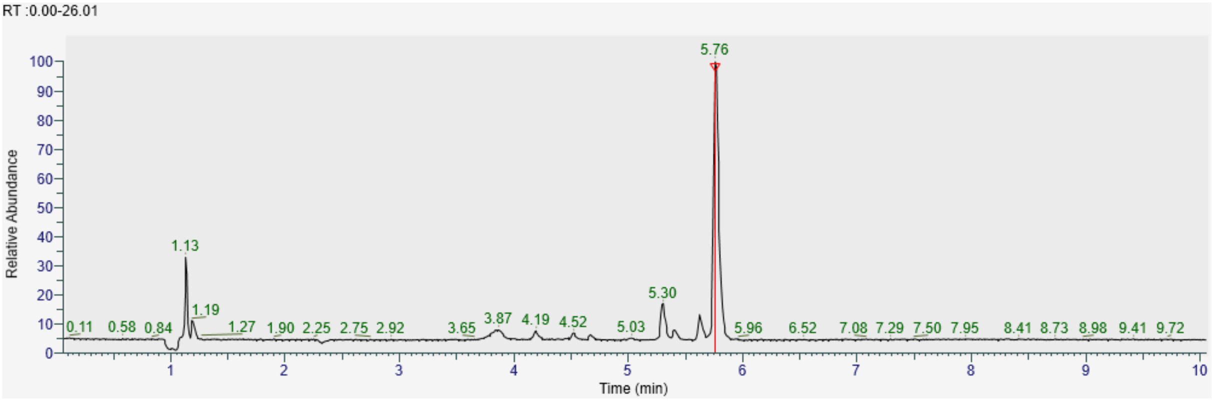

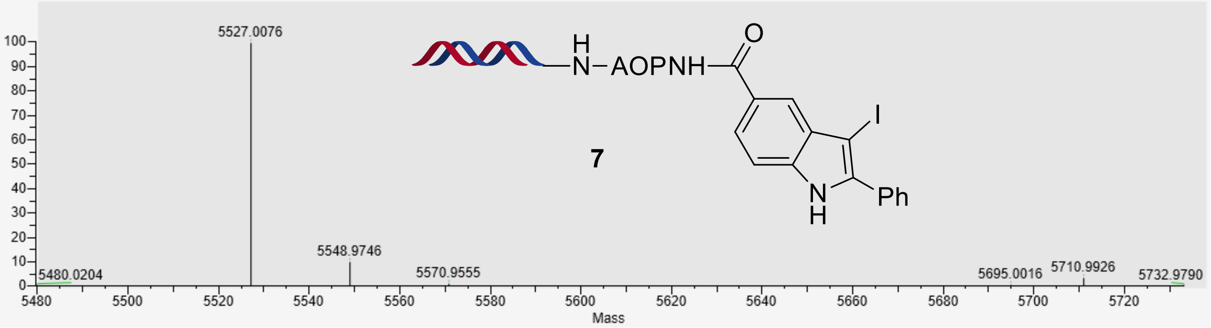

Retention time: 5.76 min

Calcd. for $C_{180}H_{244}N_{54}O_{107}P_{17}I$ 5526.9896; found 5527.0076

Reaction 5.

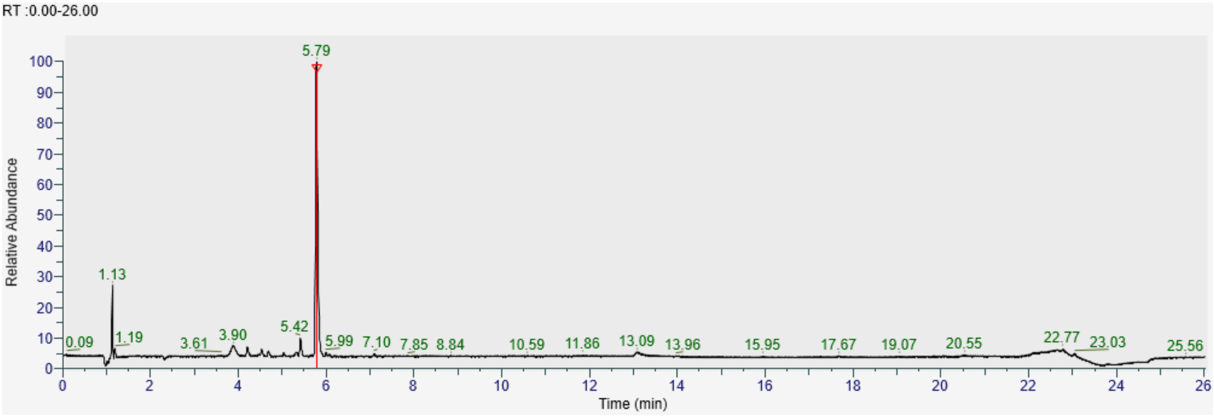

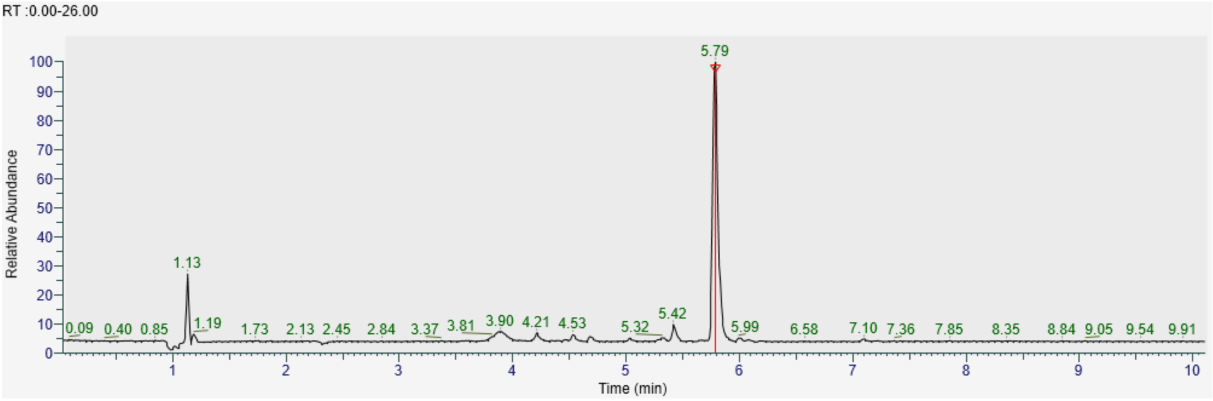

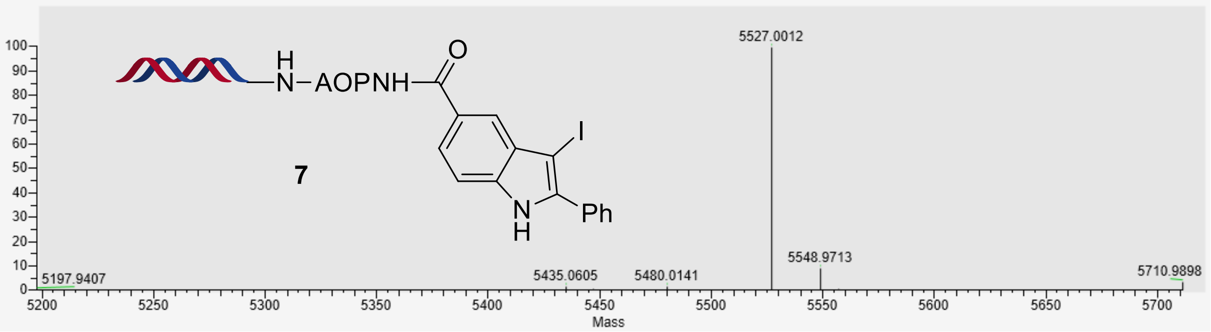

Retention time: 5.79 min

Calcd. for $C_{180}H_{244}N_{54}O_{107}P_{17}I$ 5526.9896; found 5527.0012

Reaction 6.

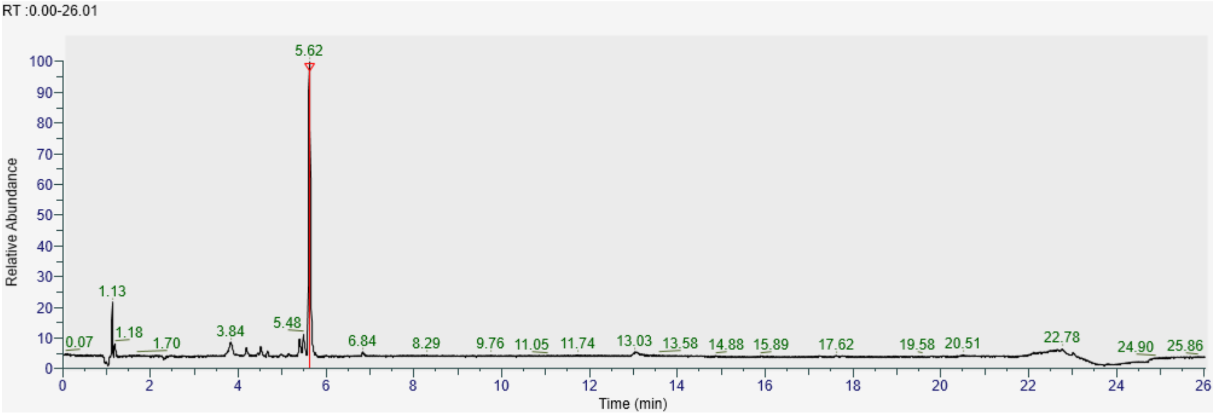

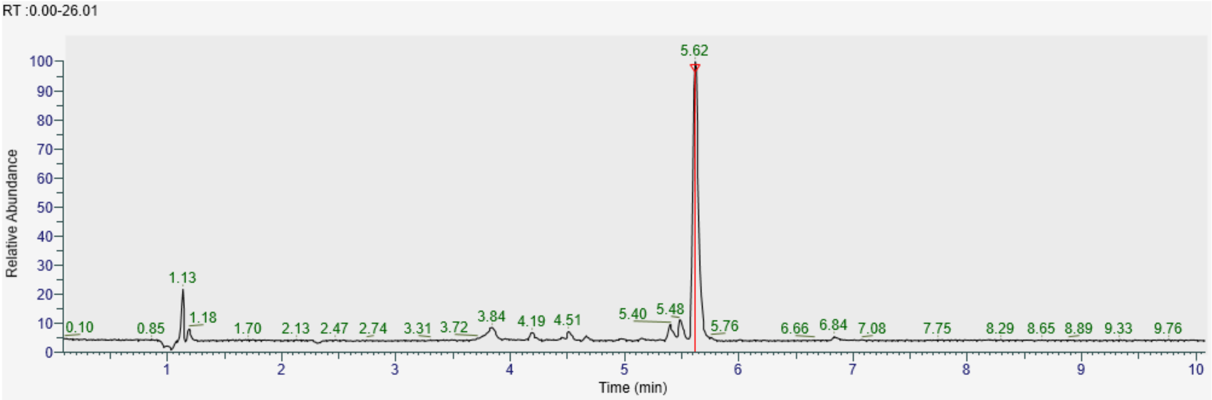

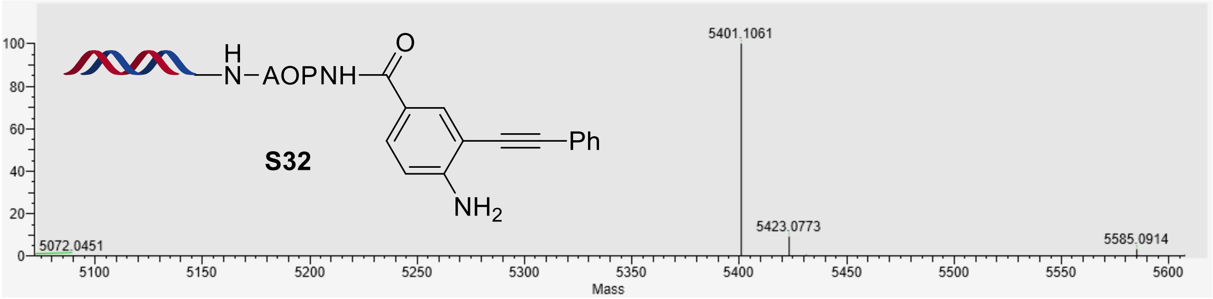

Retention time of **S32** 5.62 min

Calcd. for $C_{180}H_{245}N_{54}O_{107}P_{17}$ 5401.0930; found 5401.1061

Also contains 5% of **3a** (retention time: 5.48 min)

Reaction 7.

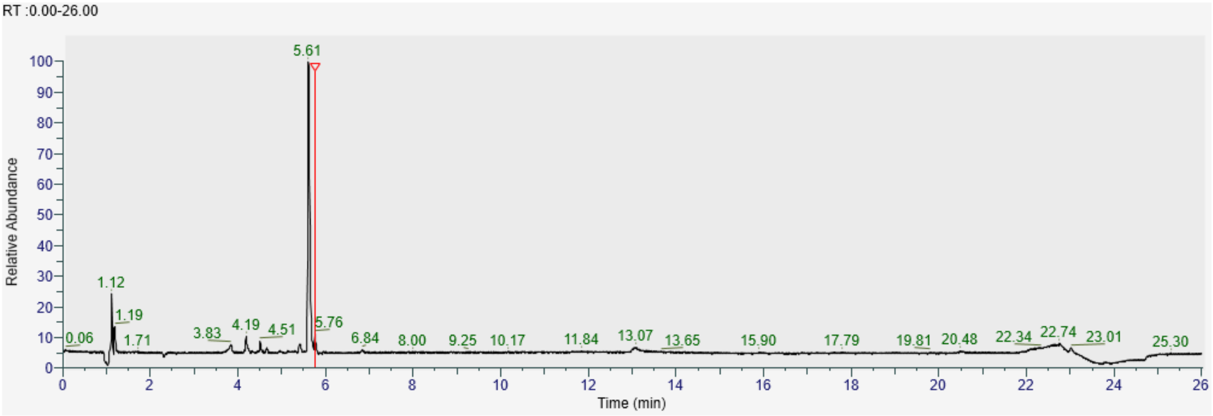

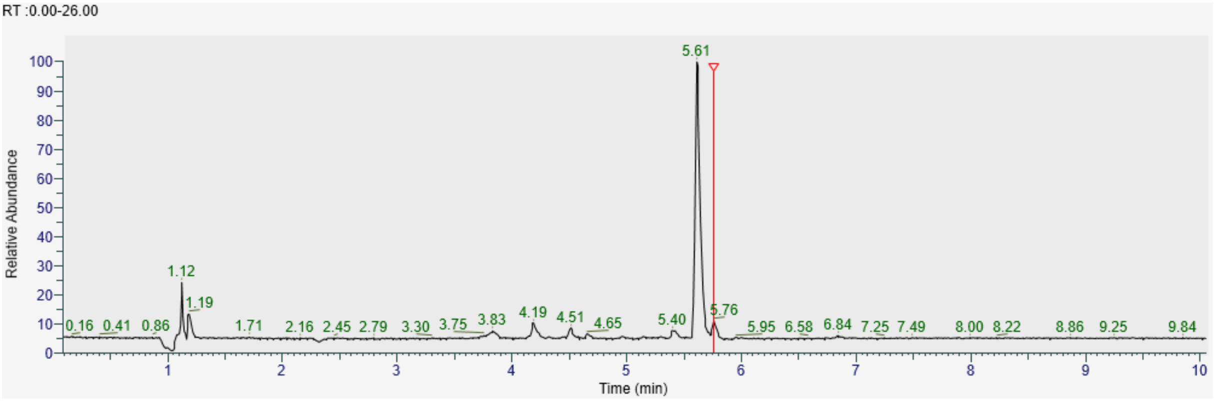

Retention time 5.61 min: **S32**

Retention time 5.76 min: **7** (from 5% of **3a**)

b. HPLC-MS co-injection

We have co-injected the samples of Reaction 1. and 3. to ensure that the proposed structure of **3a** synthesized by the developed method (Reaction 1.) is identical to that of the reference compound (Reaction 3.). The retention time of the expected indole is 5.48 min, the deconvoluted mass is 5401.0999.

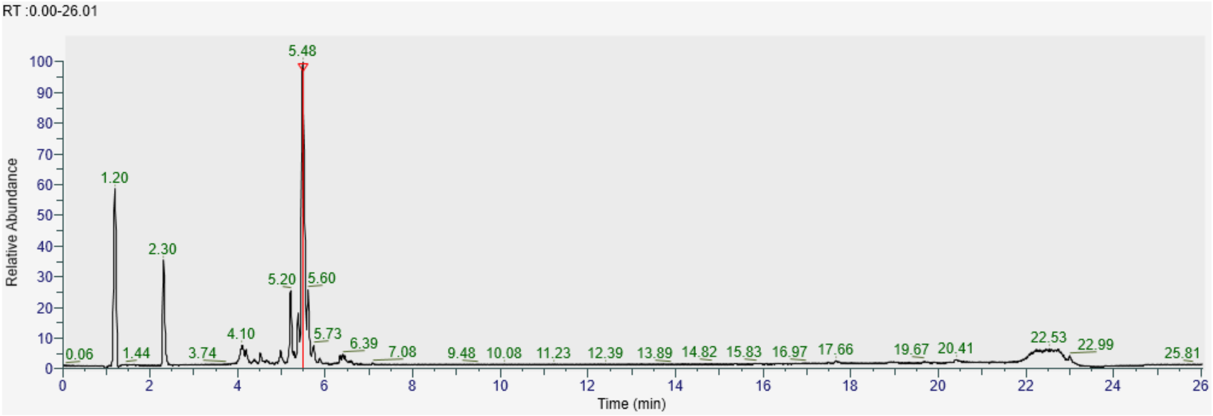
RT :0.00-26.01
Relative Abundance
Time (min)
5.48
1.20
2.30
5.20
5.60
4.10
5.73
6.39
0.06
1.44
3.74
7.08
9.48
10.08
11.23
12.39
13.89
14.82
15.83
16.97
17.66
19.67
20.41
22.53
22.99
25.81

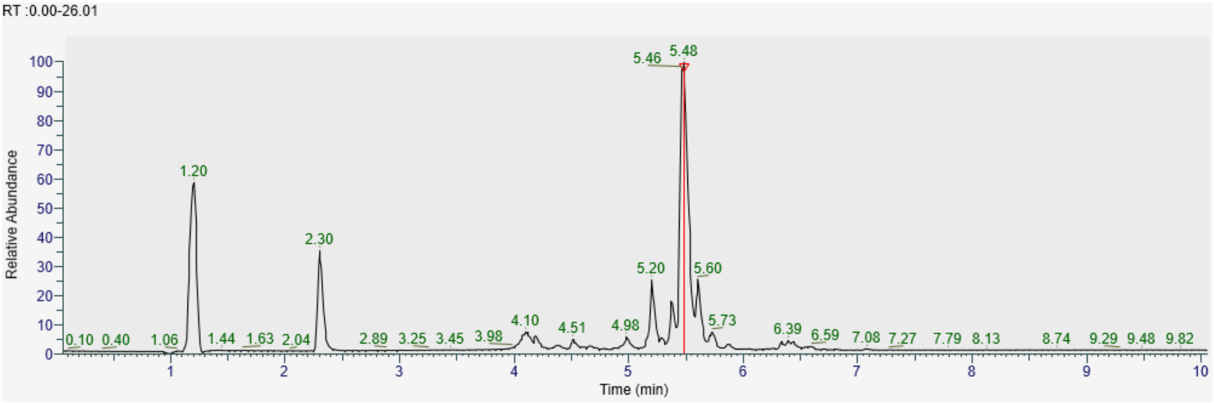
RT :0.00-26.01
Relative Abundance
Time (min)
5.46
5.48
1.20
2.30
5.20
5.60
4.10
4.51
4.98
5.73
6.39
6.59
0.10
0.40
1.06
1.44
1.63
2.04
2.89
3.25
3.45
3.98
7.08
7.27
7.79
8.13
8.74
9.29
9.48
9.82

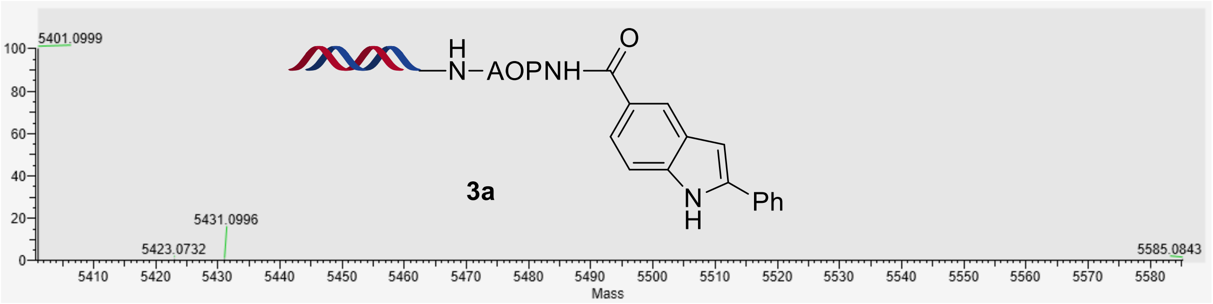
5401.0999
5431.0996
5423.0732
5585.0843
Mass
H
N–AOPNH
O
N
H
Ph
3a

9. HPLC-MS spectra

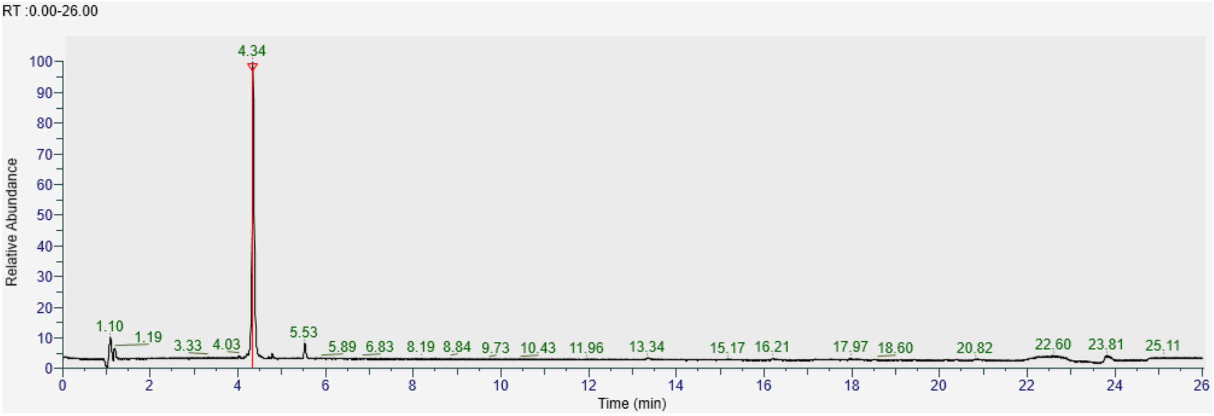

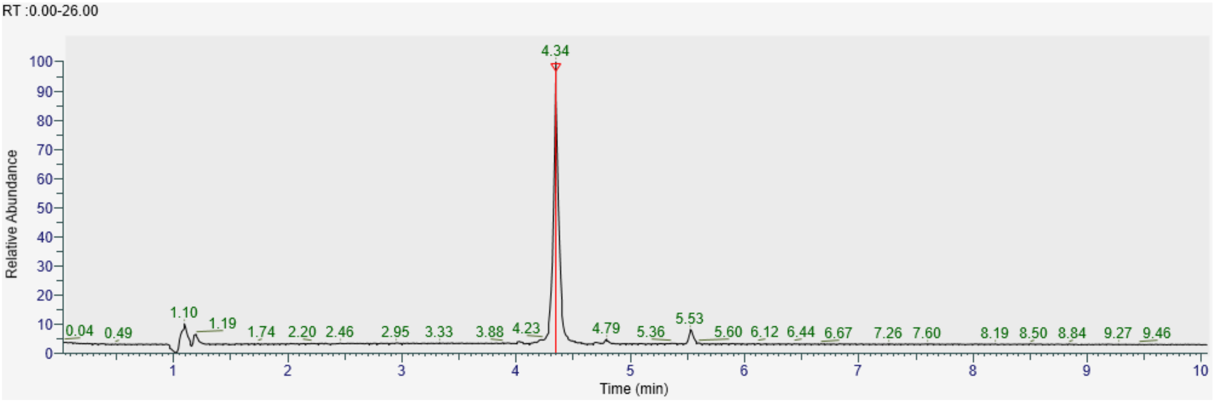

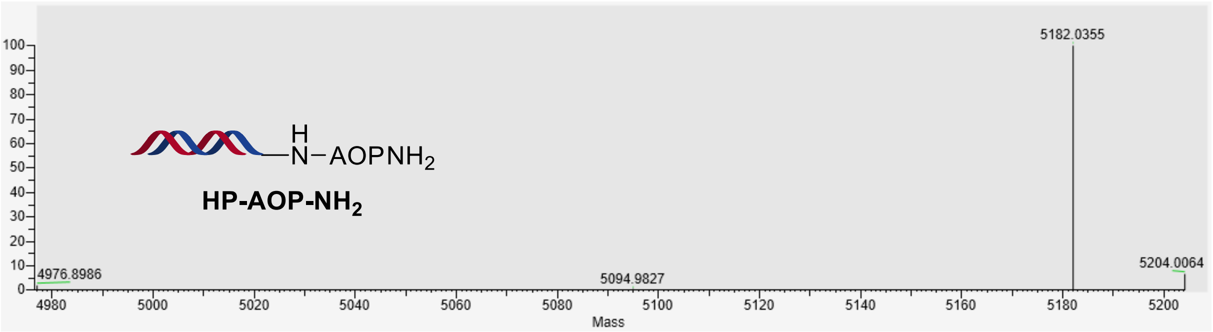

Retention time of **HP-AOP-NH2**: 4.34 min

Yield: 93%

Calcd. for $C_{165}H_{236}N_{53}O_{106}P_{17}$ 5182.0246; found 5182.0355

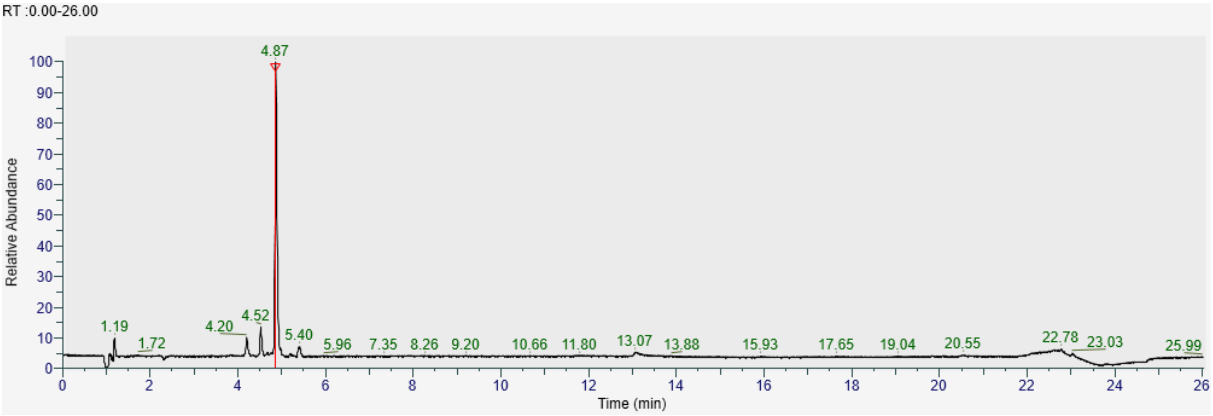

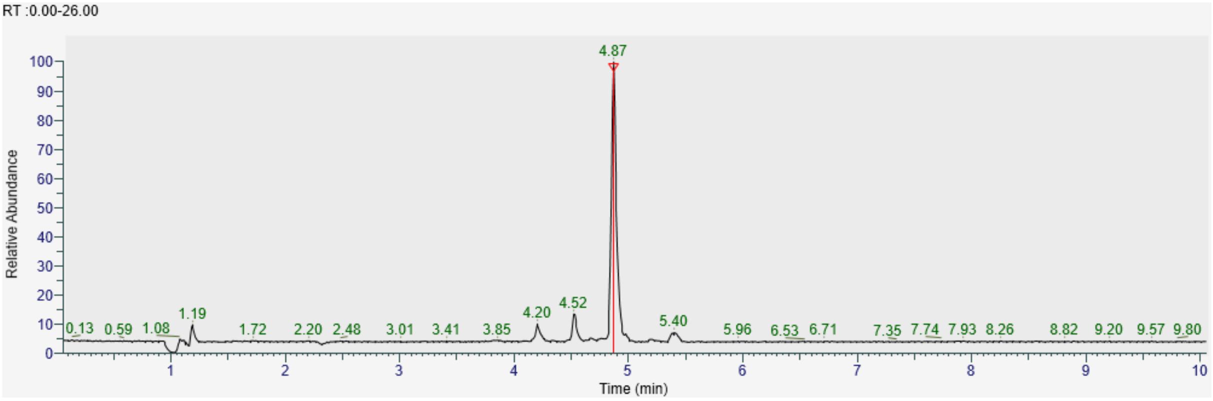

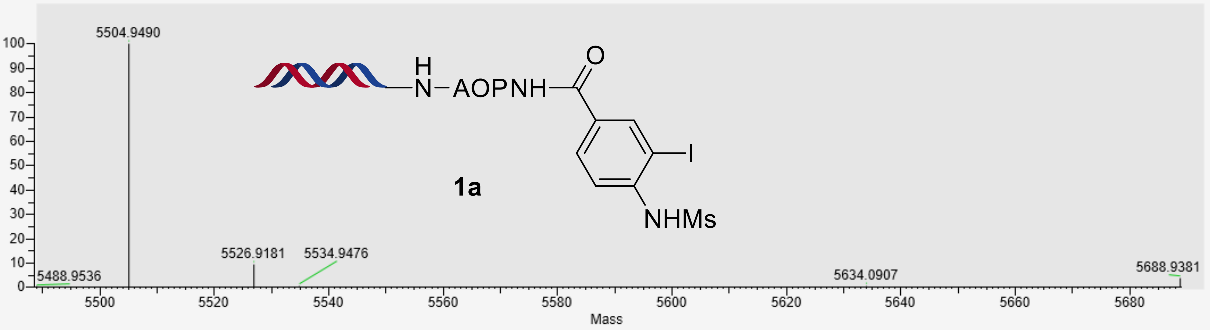

Retention time of **1a**: 4.87 min

Yield: 88%

Calcd. for $C_{173}H_{242}N_{54}O_{109}P_{17}SI$ 5504.9359; found 5504.9490

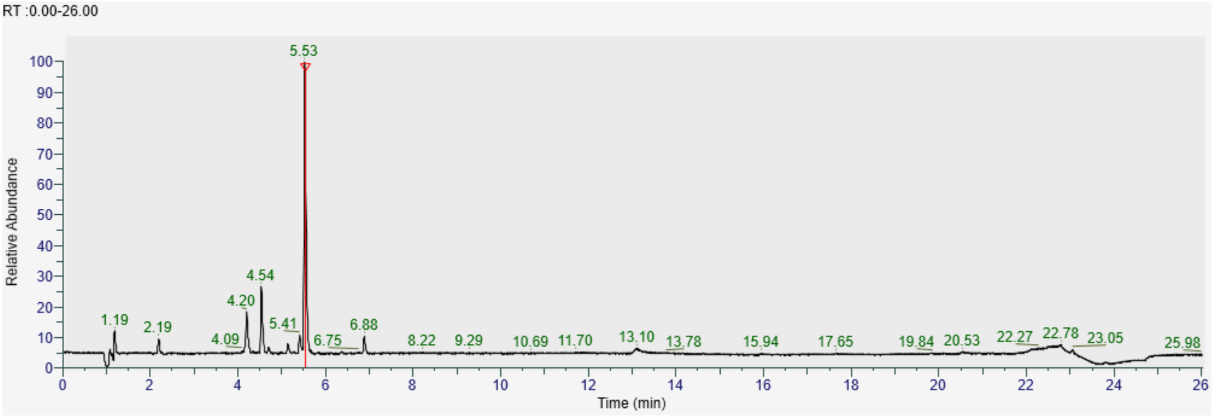

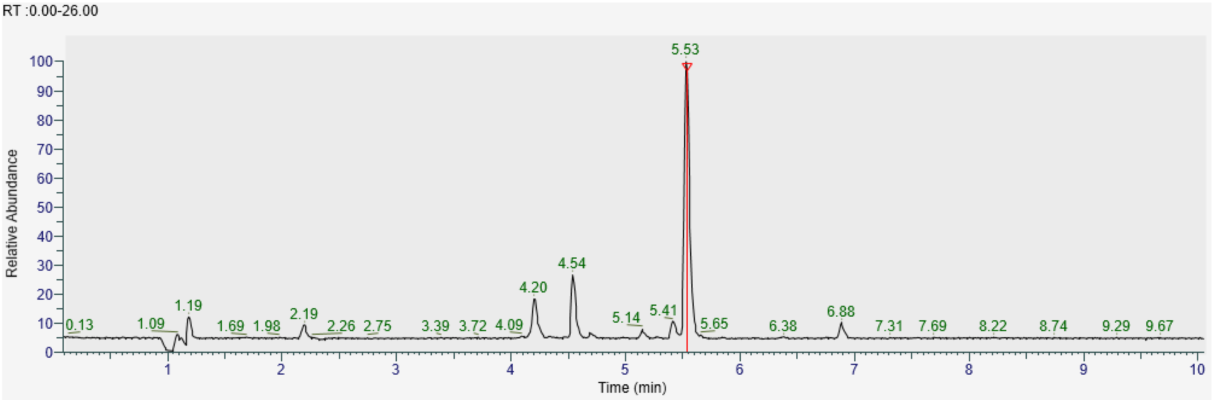

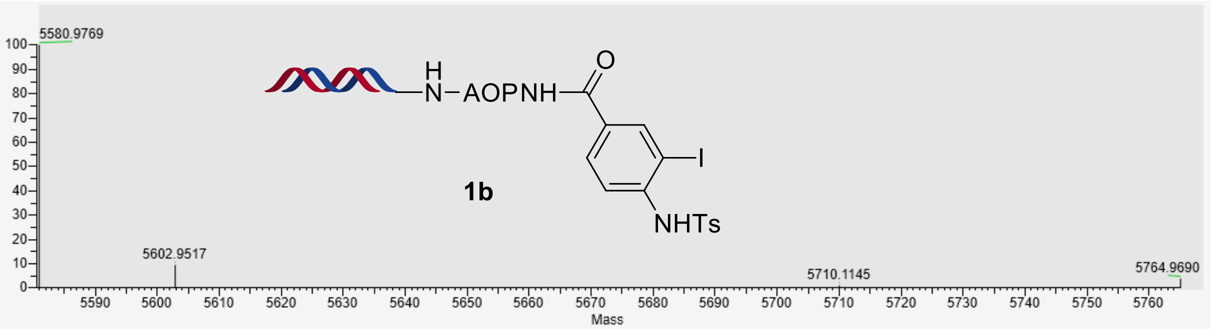

Retention time of **1b**: 5.53 min

Yield: 73% (68%)

Calcd. for $C_{179}H_{246}N_{54}O_{109}P_{17}SI$ 5580.9672; found 5580.9769

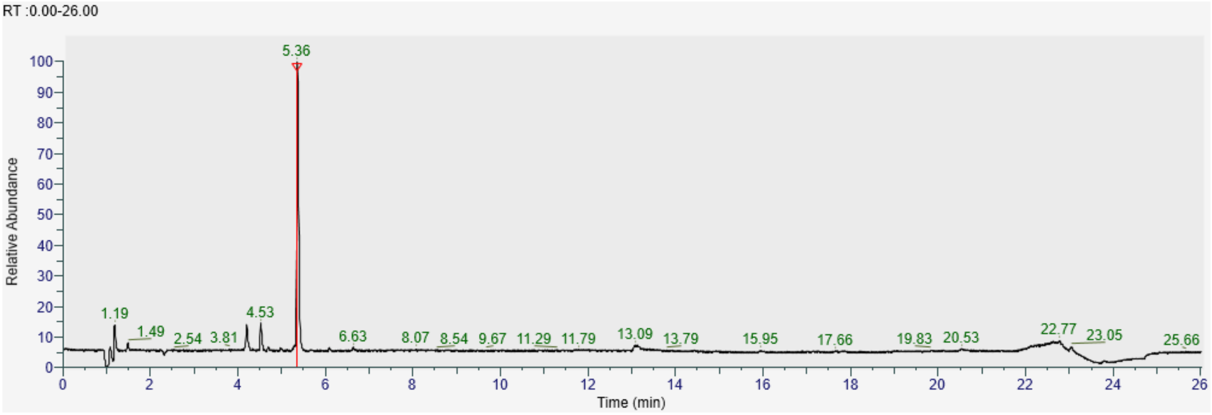

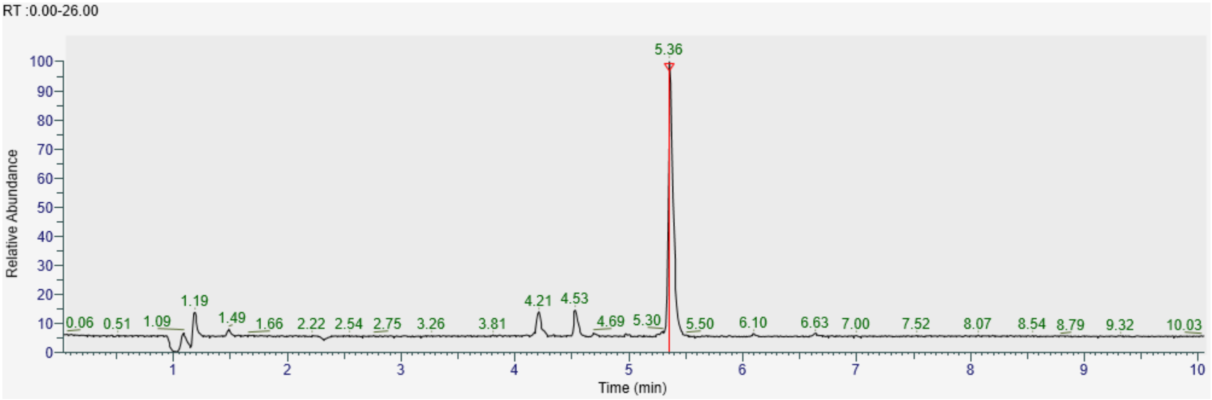

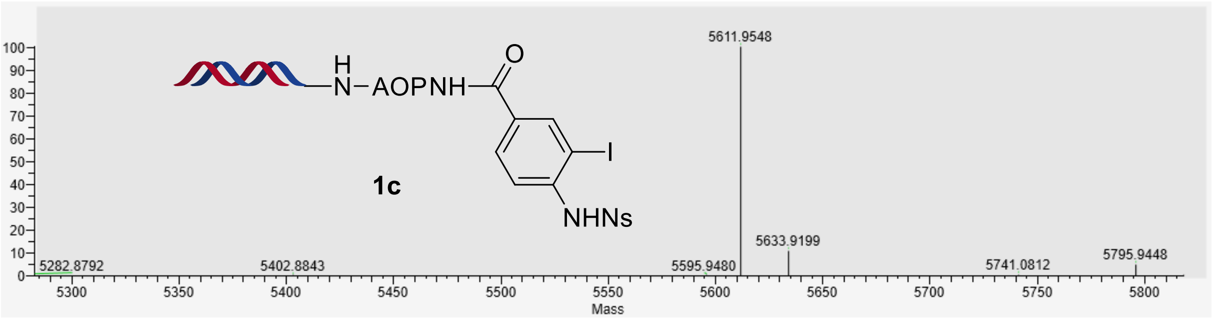

Retention time of **1c**: 5.36 min

Yield: 88% (82%)

Calcd. for $C_{178}H_{243}N_{55}O_{111}P_{17}SI$ 5611.9366; found 5611.9548

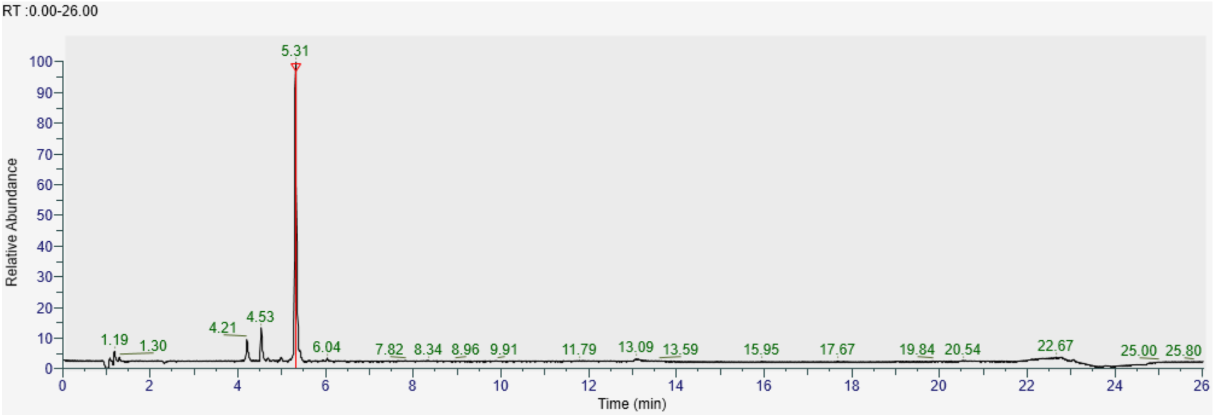

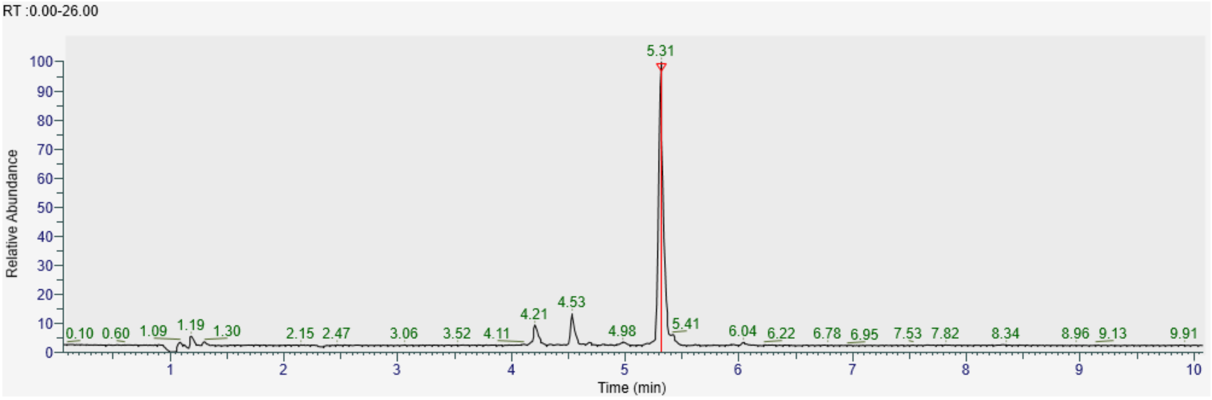

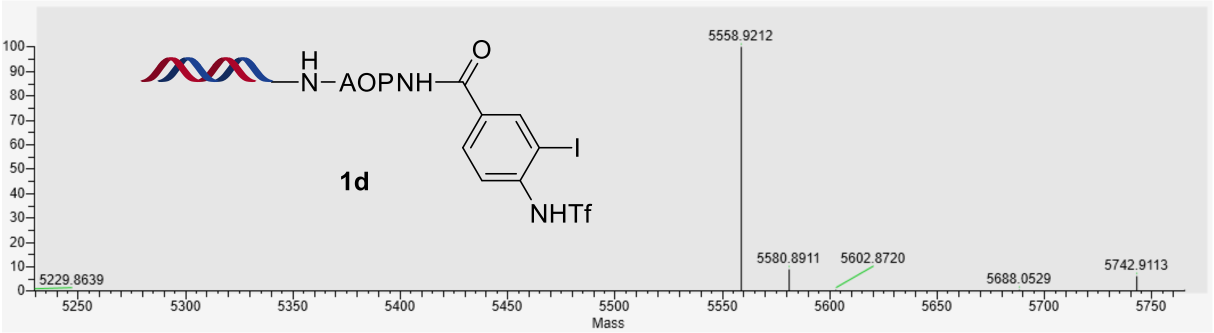

Retention time of **1d**: 5.31 min

Yield: 92% (86%)

Calcd. for $C_{173}H_{239}N_{54}O_{109}P_{17}SF_3I$ 5558.9076; found 5558.9212

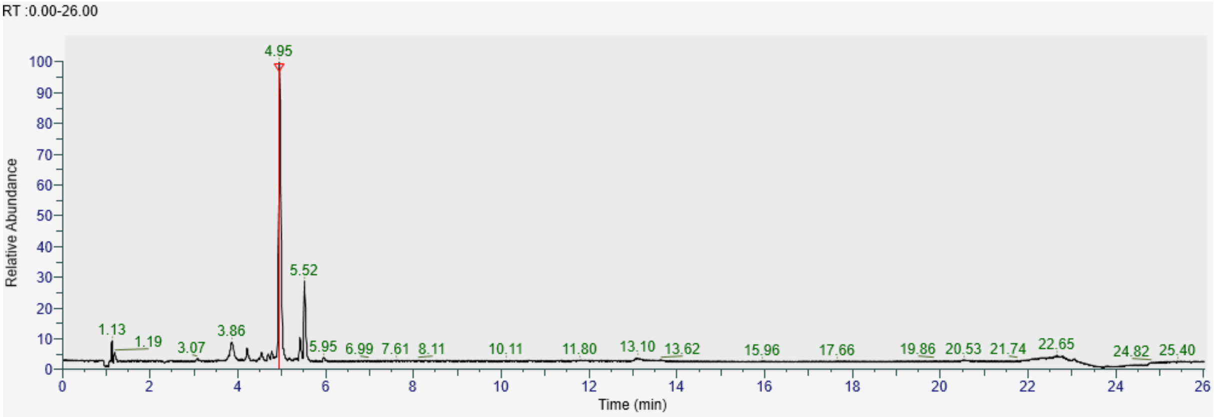

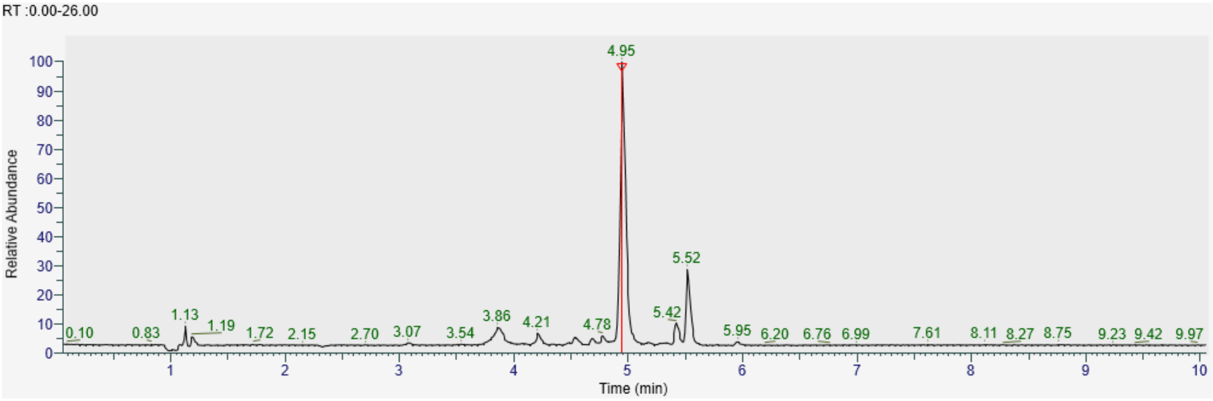

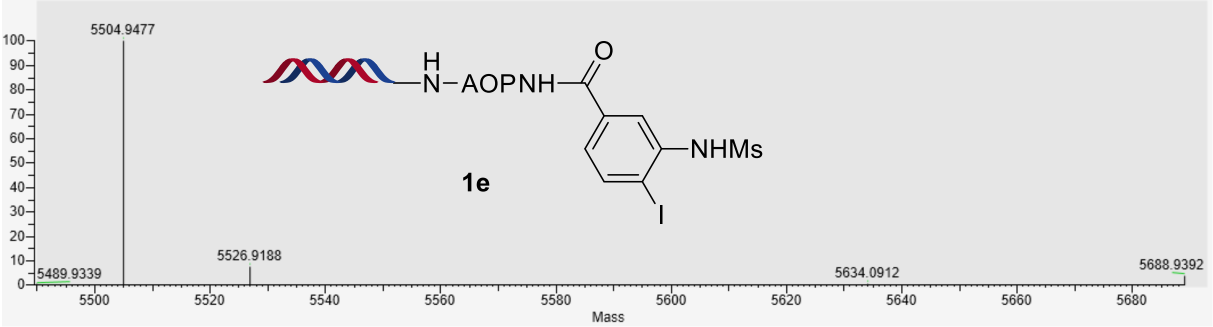

Retention time of **1e**: 4.95 min

Yield: 72% (67%)

Calcd. for $C_{173}H_{242}N_{54}O_{109}P_{17}SI$ 5504.9359; found 5504.9490

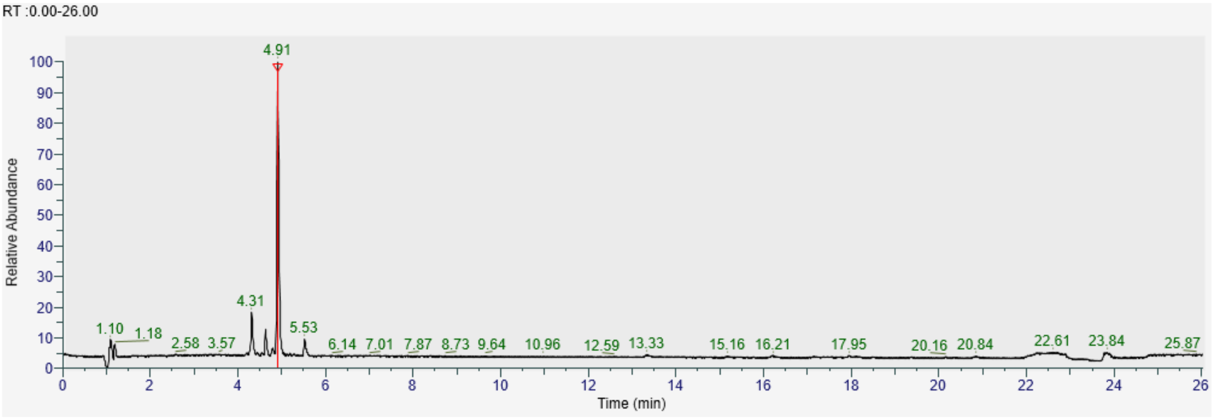

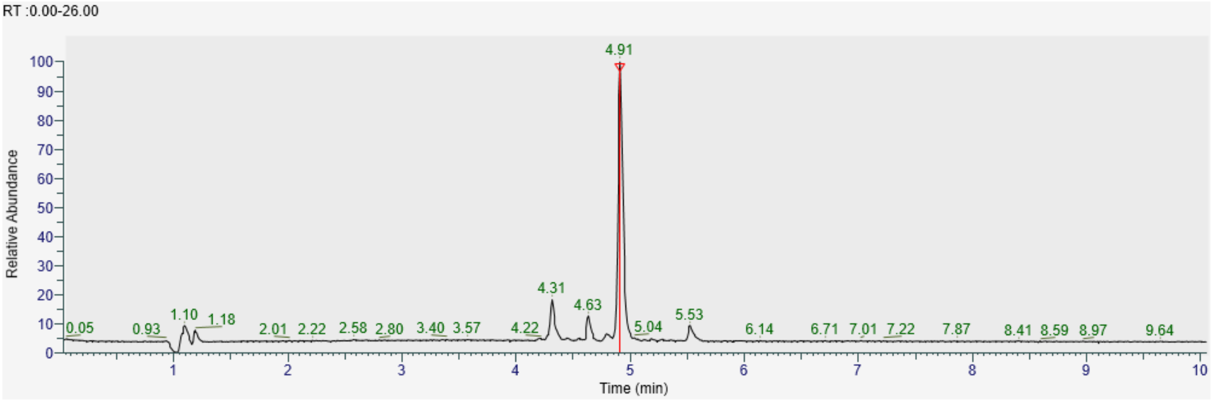

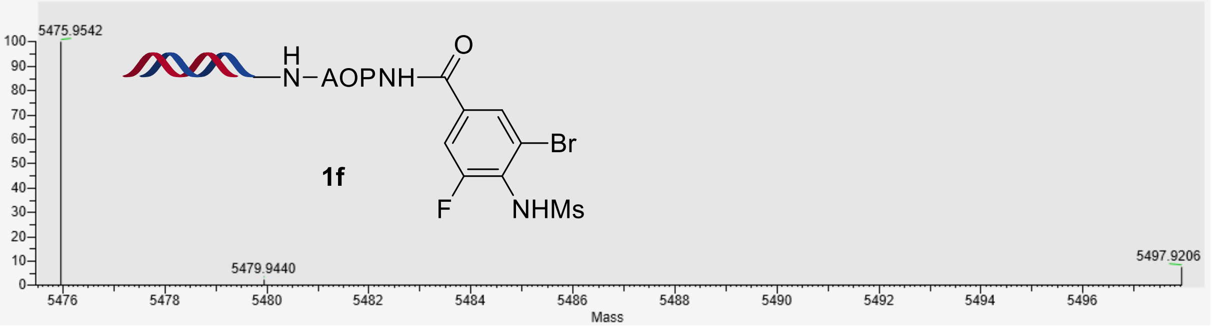

Retention time of **1f**: 4.91 min

Yield: 81% (75%)

Calcd. for $C_{173}H_{241}N_{54}O_{109}P_{17}SBrF$ 5474.9403; found 5475.9542

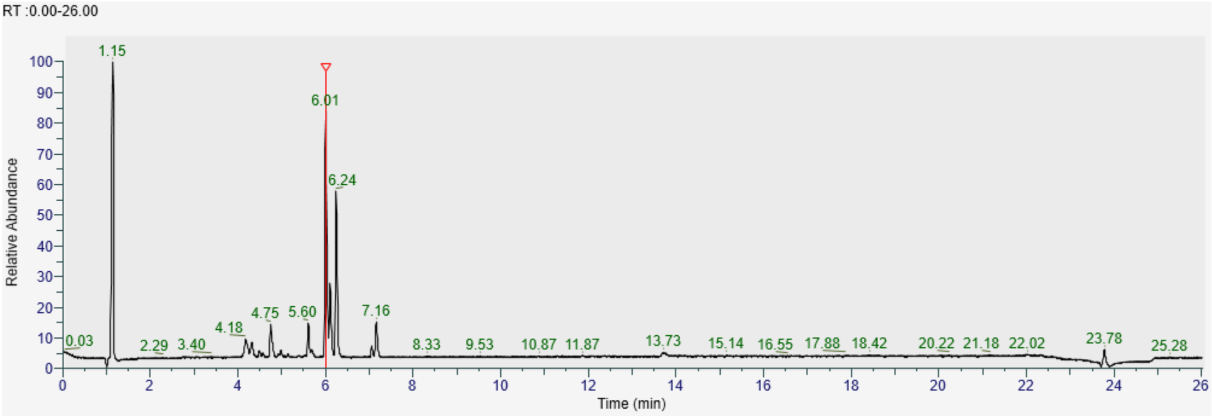

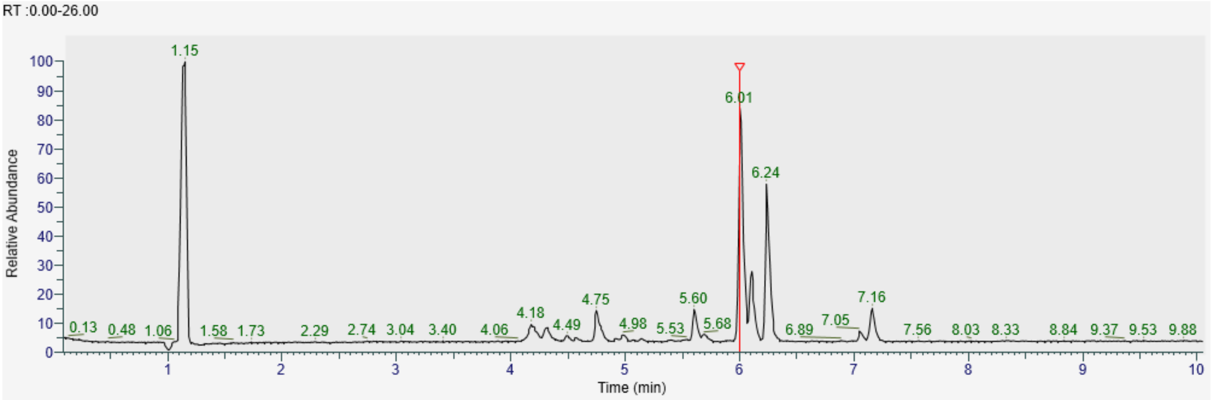

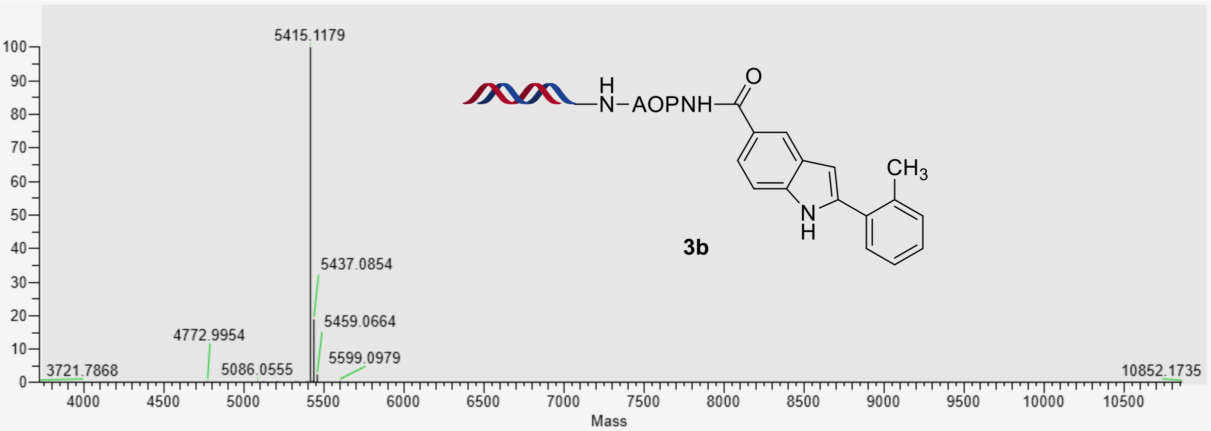

Calcd. for $C_{181}H_{247}N_{54}O_{107}P_{17}$ 5415.1086; found 5415.1179

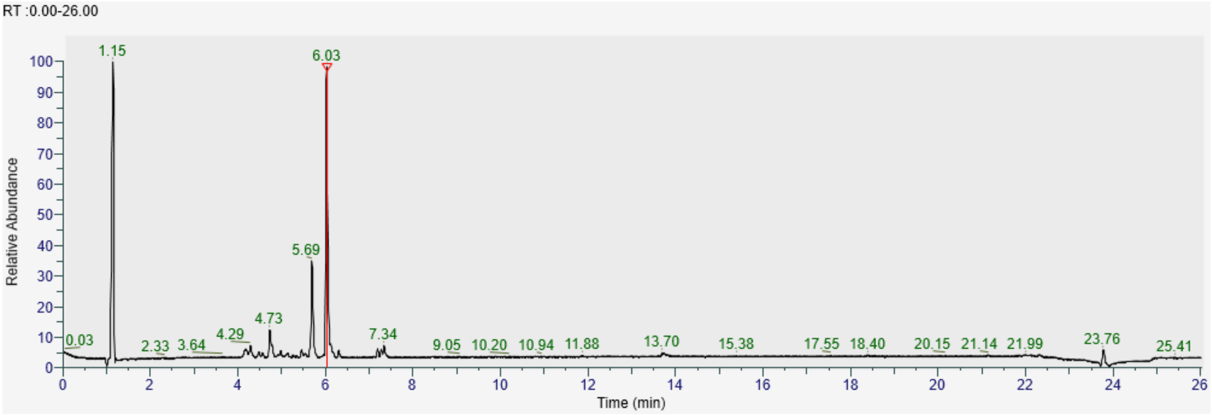

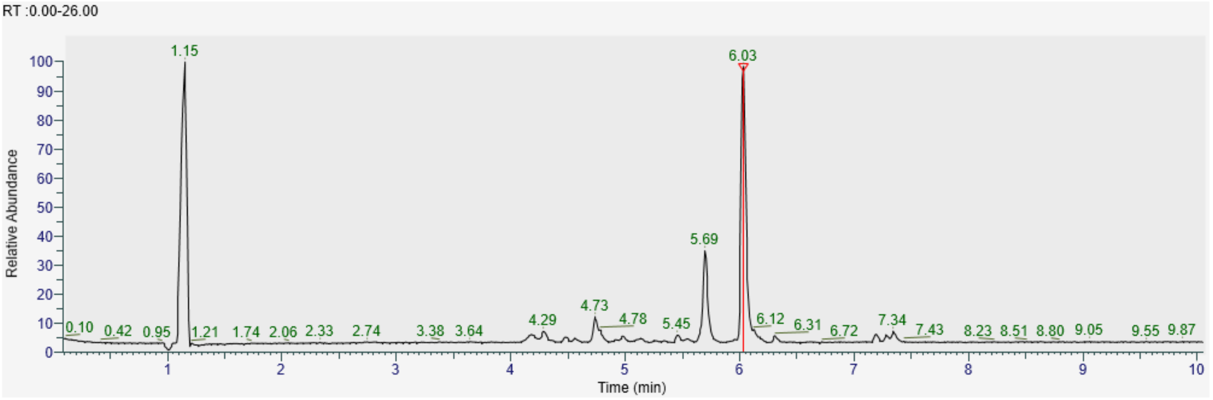

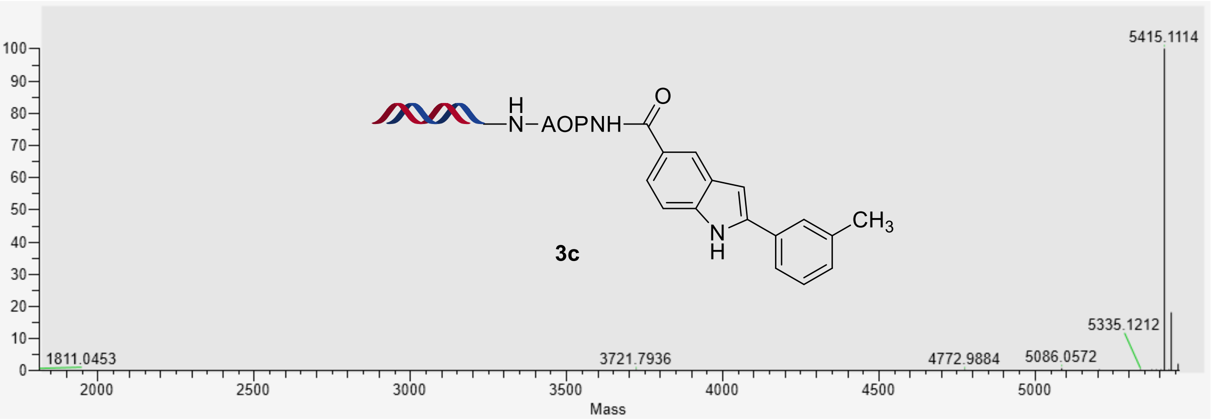

Calcd. for $C_{181}H_{247}N_{54}O_{107}P_{17}$ 5415.1086; found 5415.1114

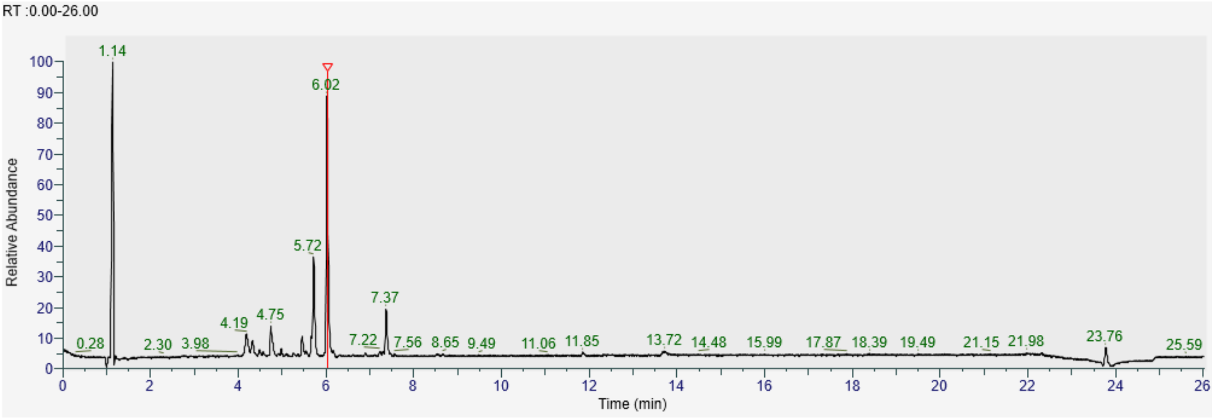

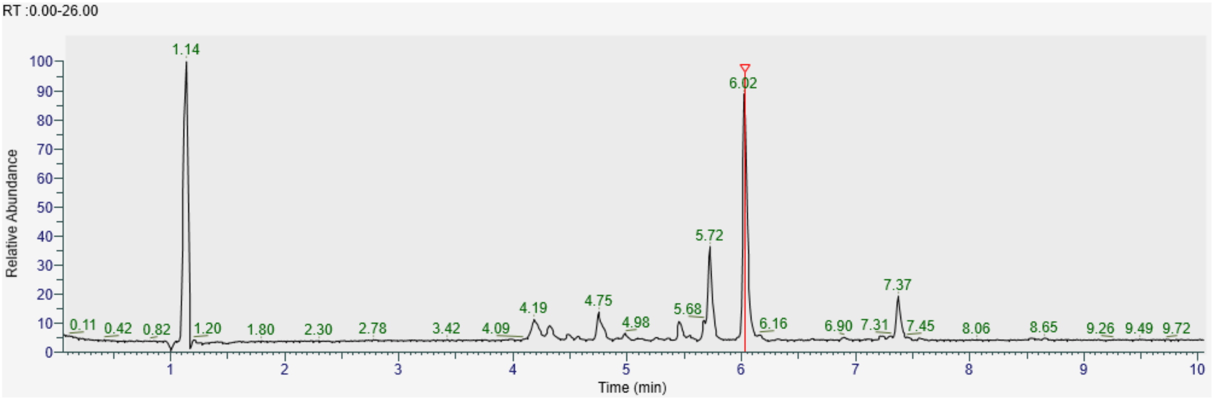

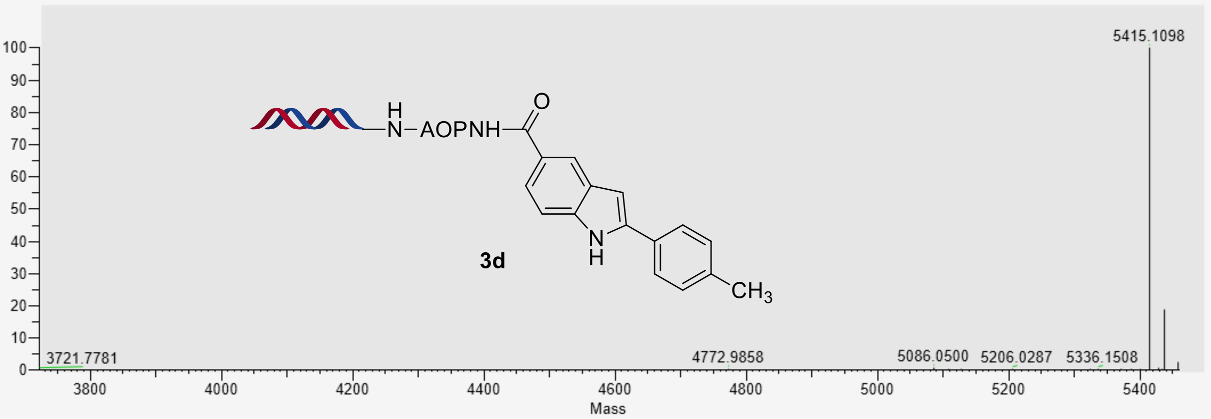

Calcd. for $C_{181}H_{247}N_{54}O_{107}P_{17}$ 5415.1086; found 5415.1098

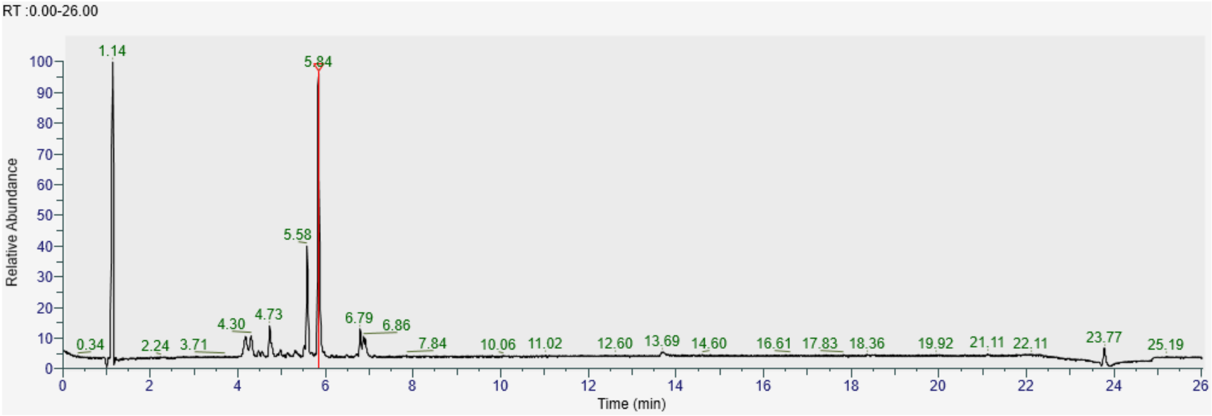

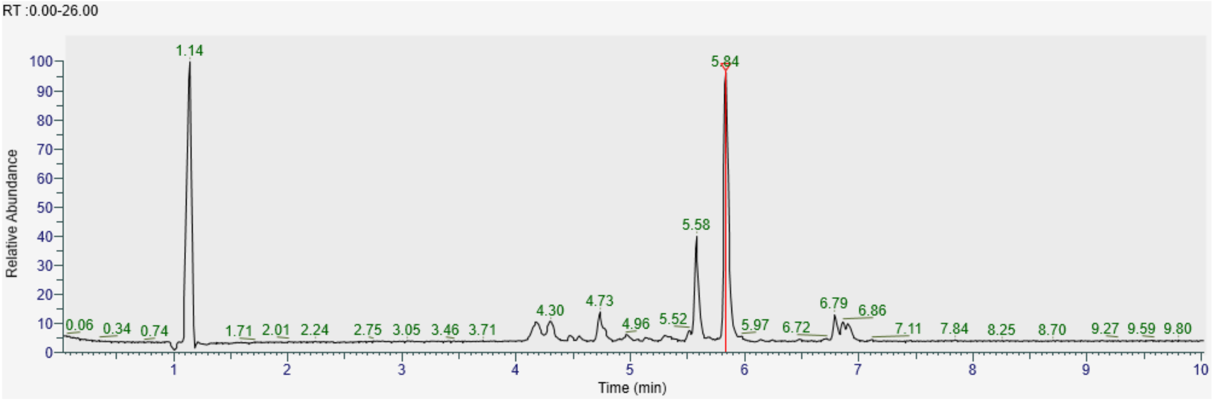

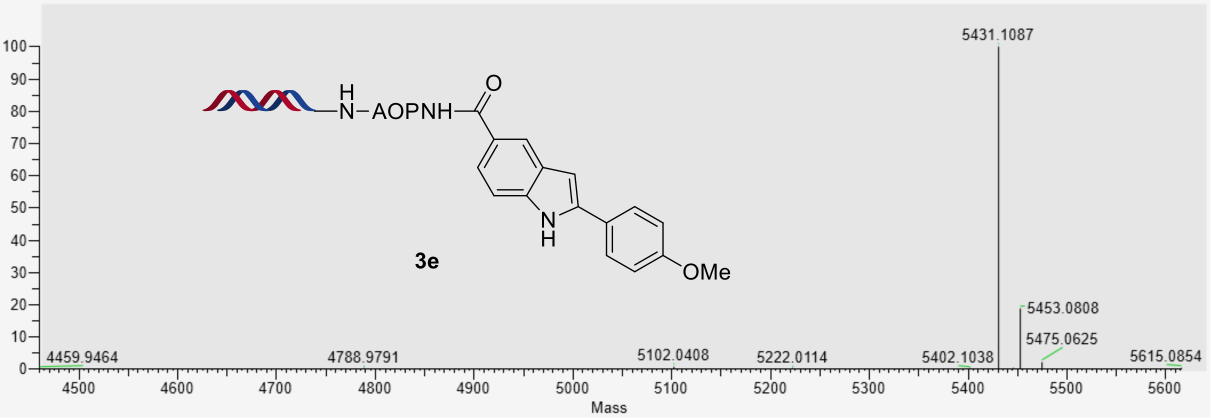

Calcd. for $C_{181}H_{247}N_{54}O_{108}P_{17}$ 5431.1036; found 5431.1087

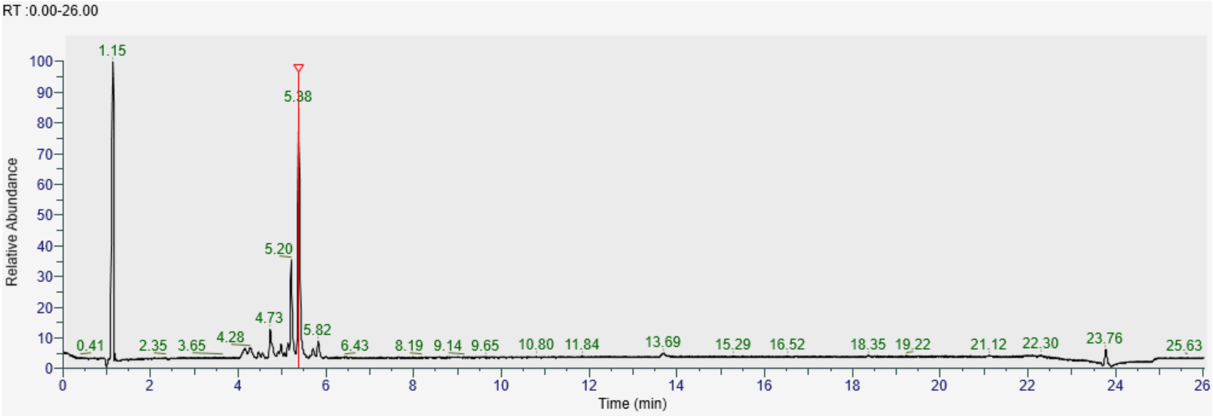

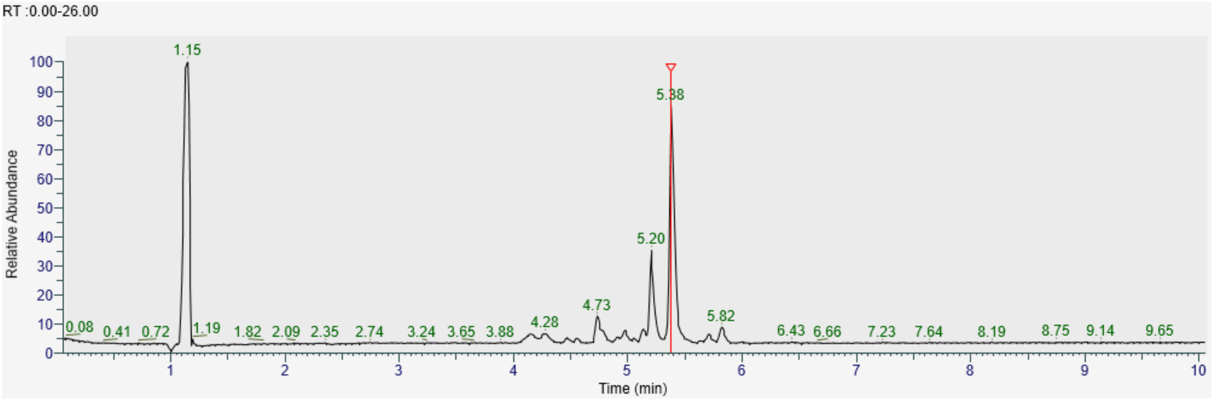

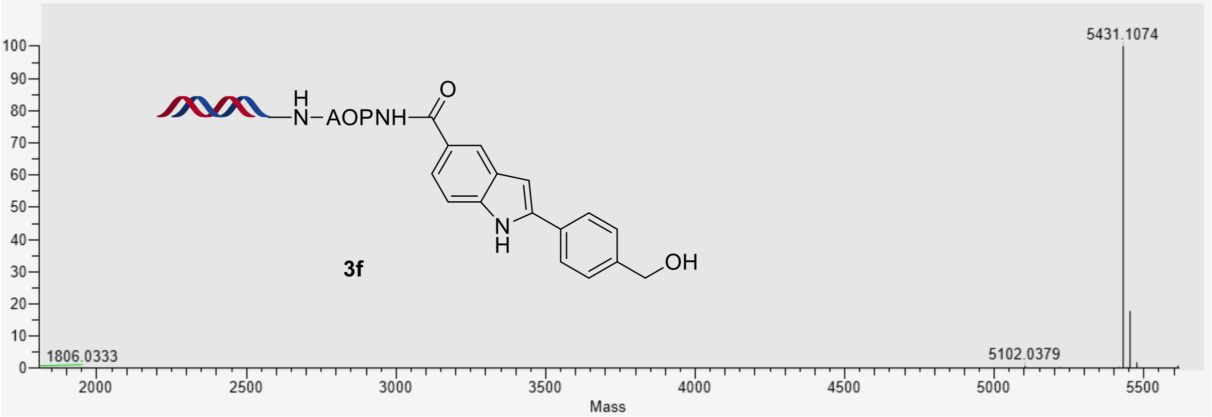

Calcd. for $C_{181}H_{247}N_{54}O_{108}P_{17}$ 5431.1036; found 5431.1074

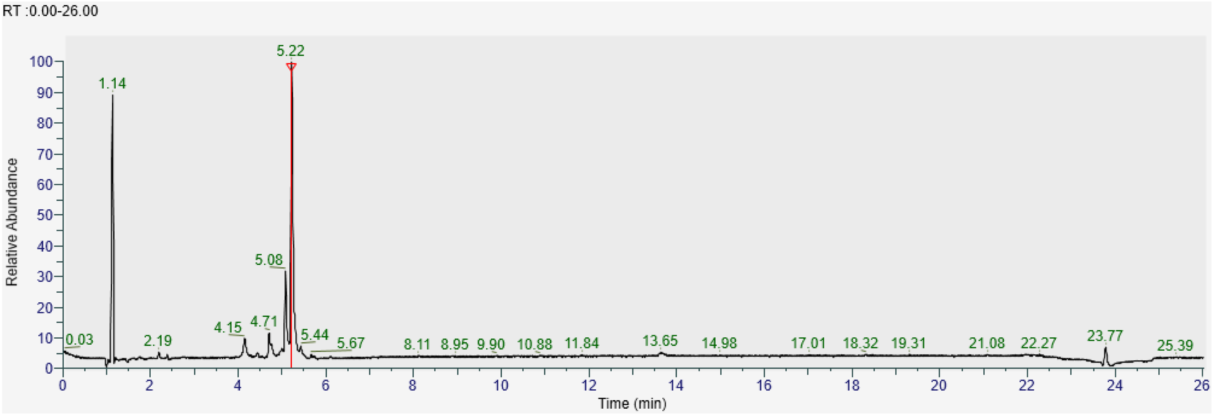

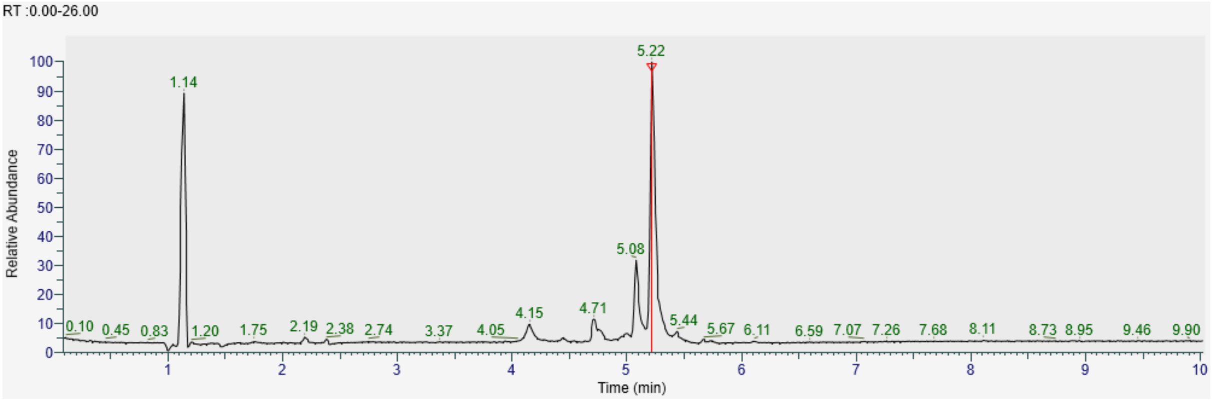

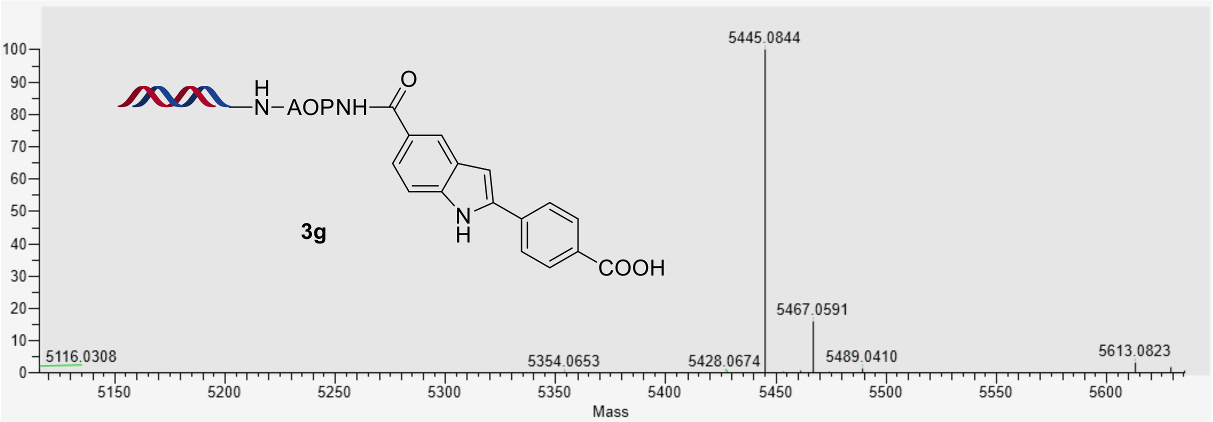

Calcd. for $C_{181}H_{245}N_{54}O_{109}P_{17}$ 5445.0828; found 5445.0844

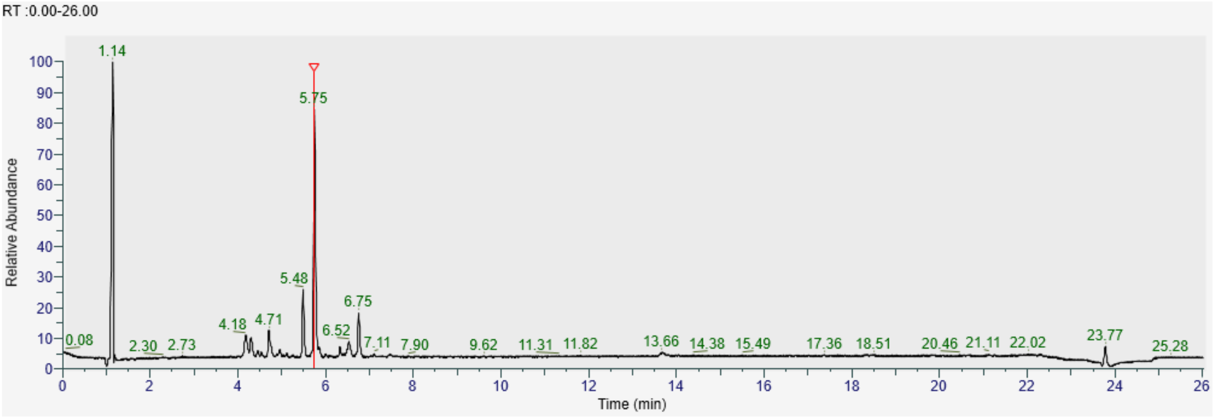

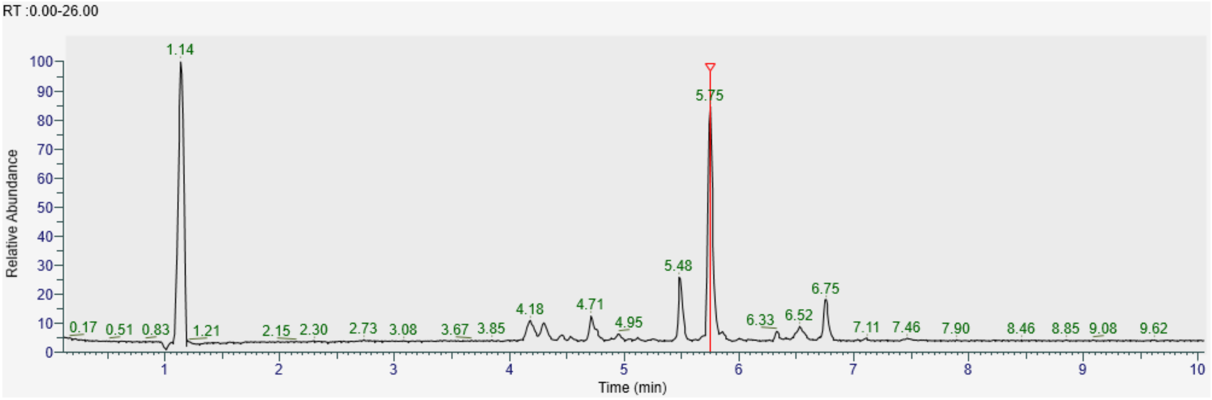

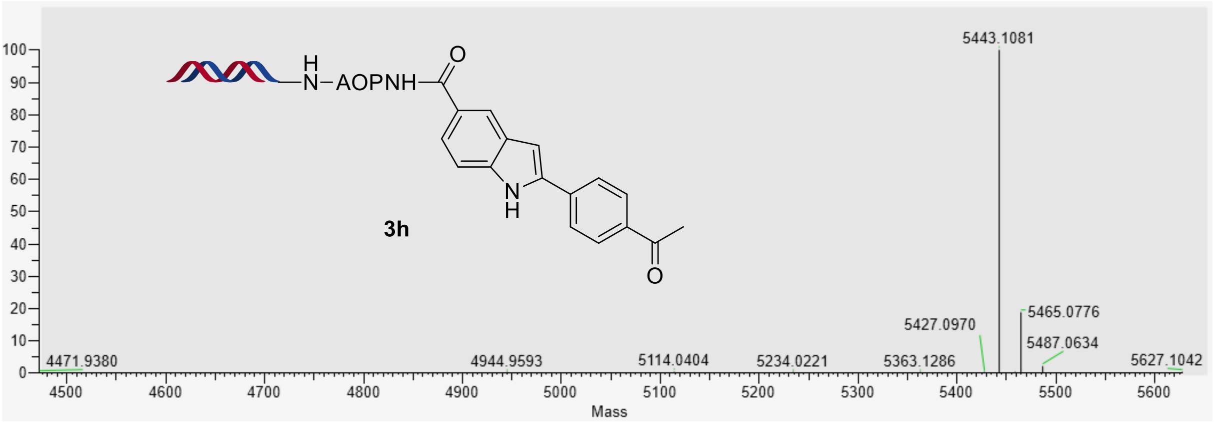

Calcd. for $C_{182}H_{247}N_{54}O_{108}P_{17}$ 5443.1036; found 5443.1081

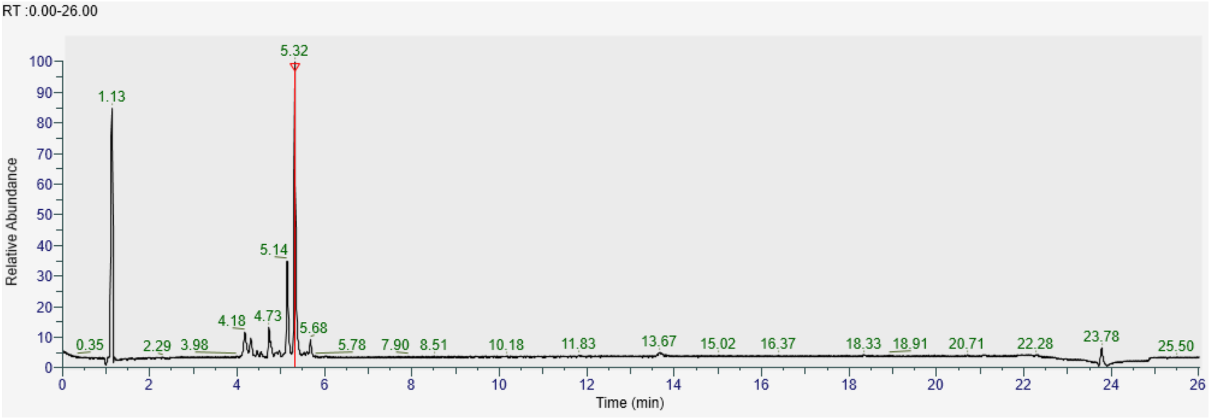

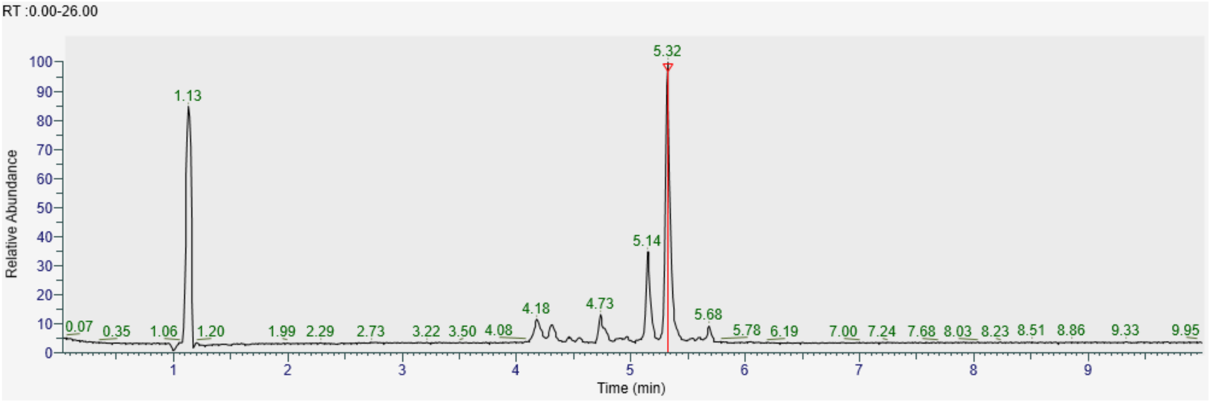

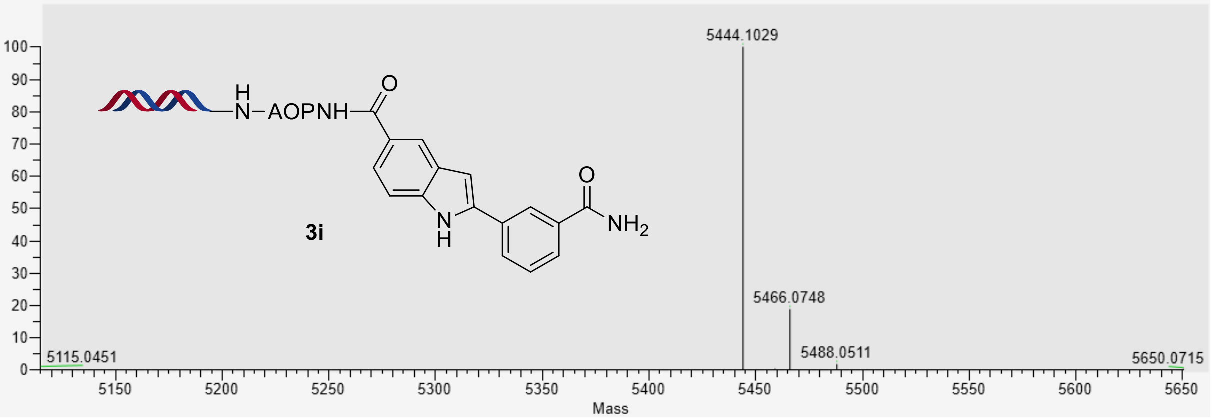

Calcd. for $C_{181}H_{246}N_{55}O_{108}P_{17}$ 5444.0988; found 5444.1029

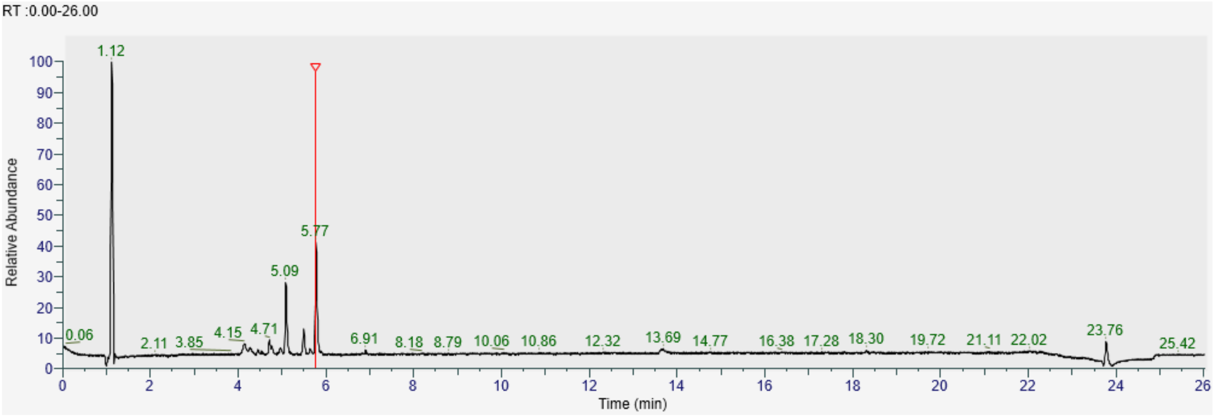

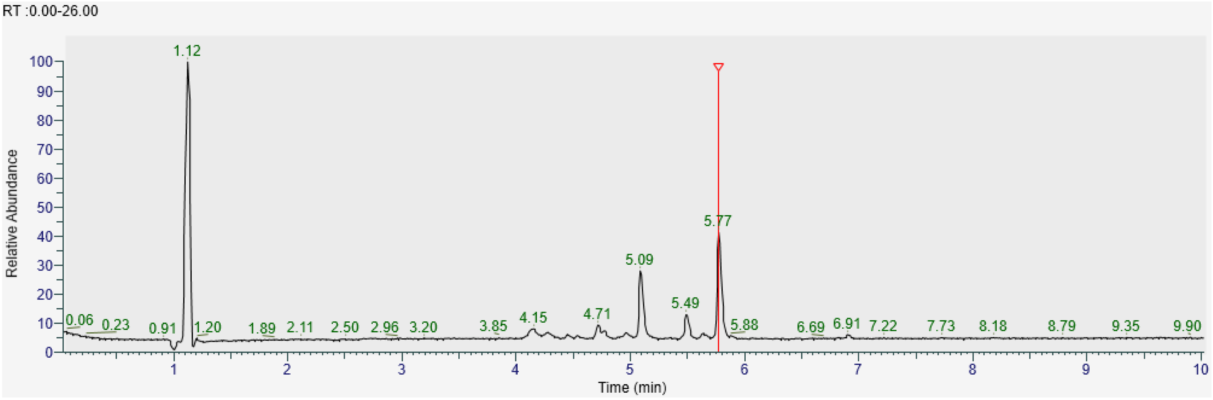

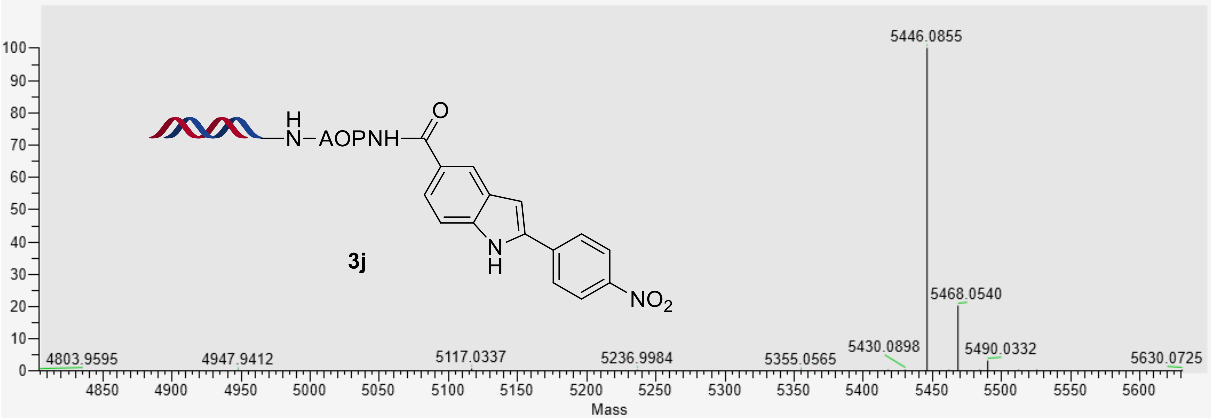

Calcd. for $C_{180}H_{244}N_{55}O_{109}P_{17}$ 5446.0781; found 5446.0855

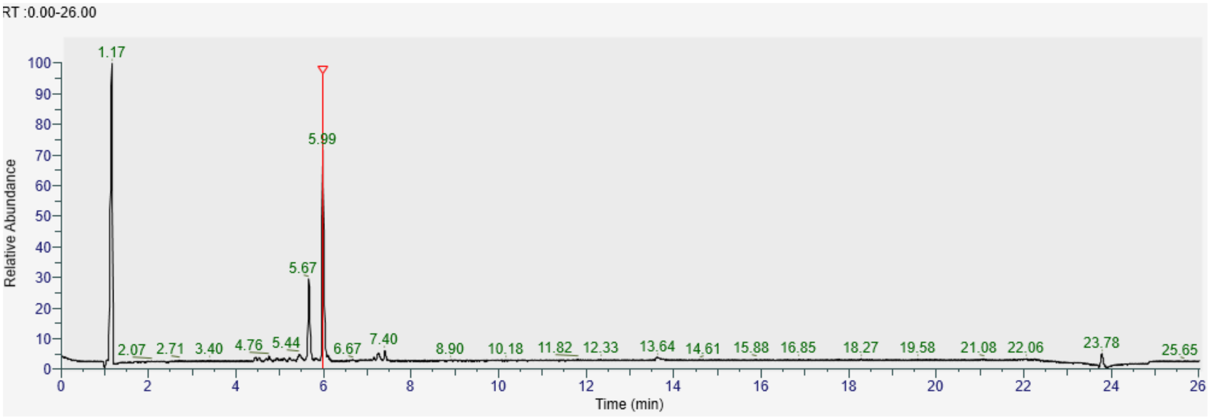

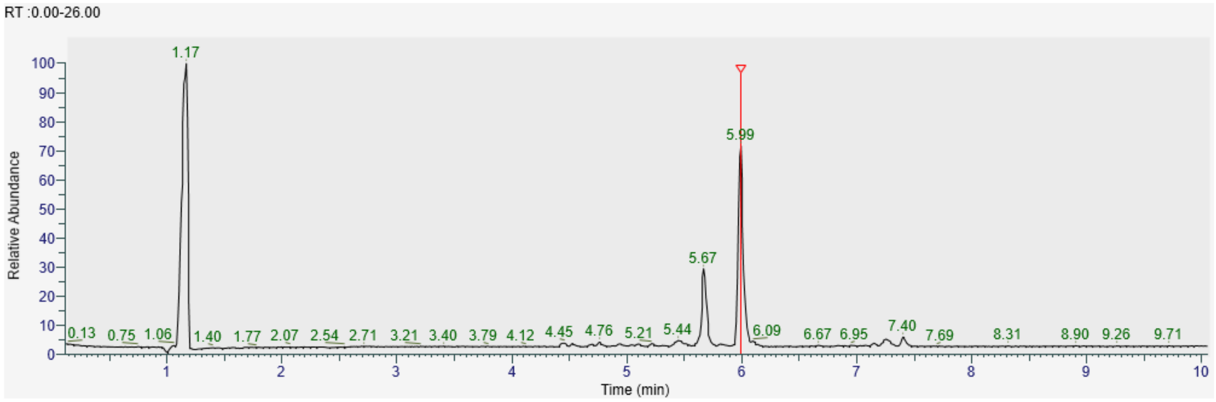

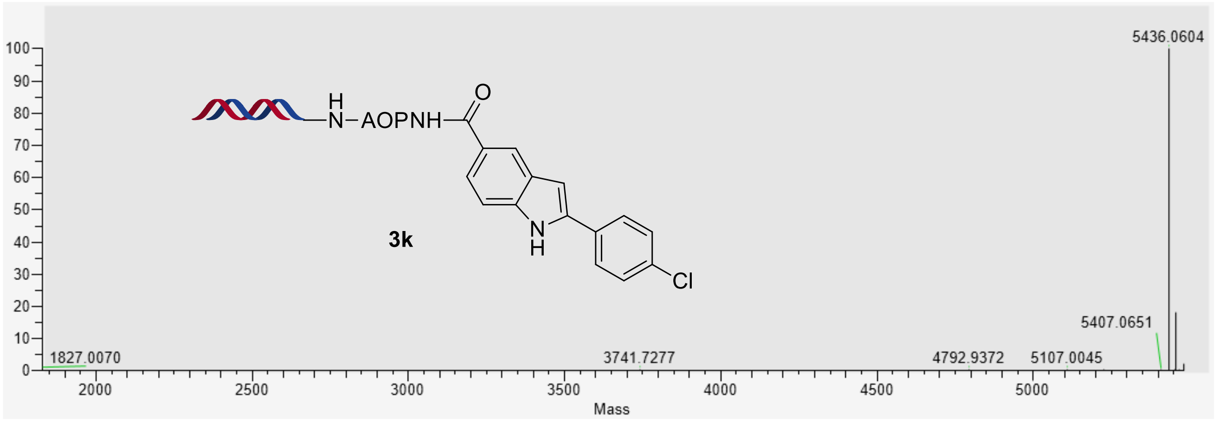

Calcd. for $C_{180}H_{244}ClN_{54}O_{107}P_{17}$ 5435.0540; found 5436.0604

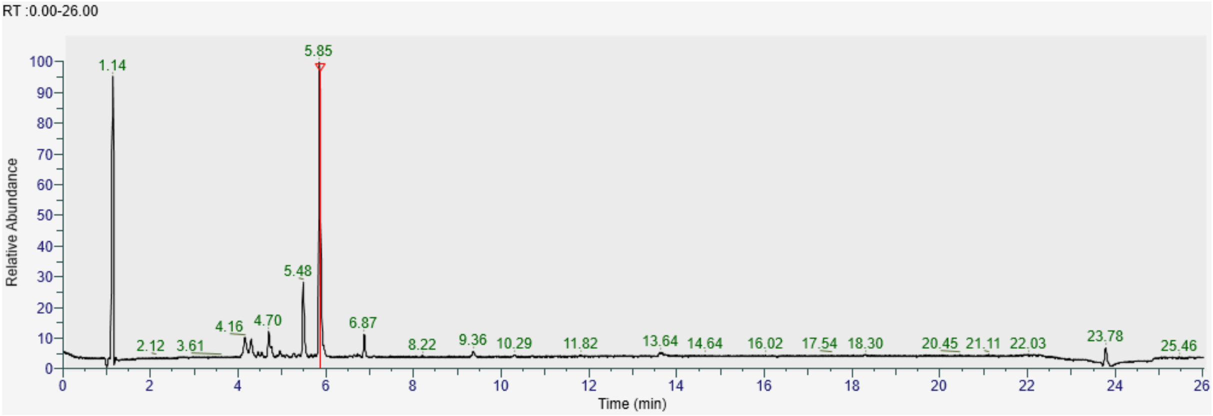

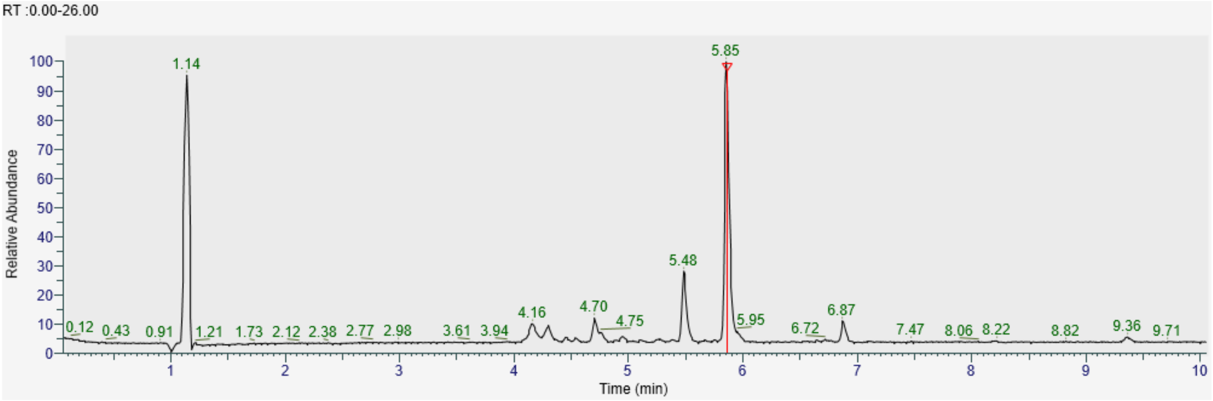

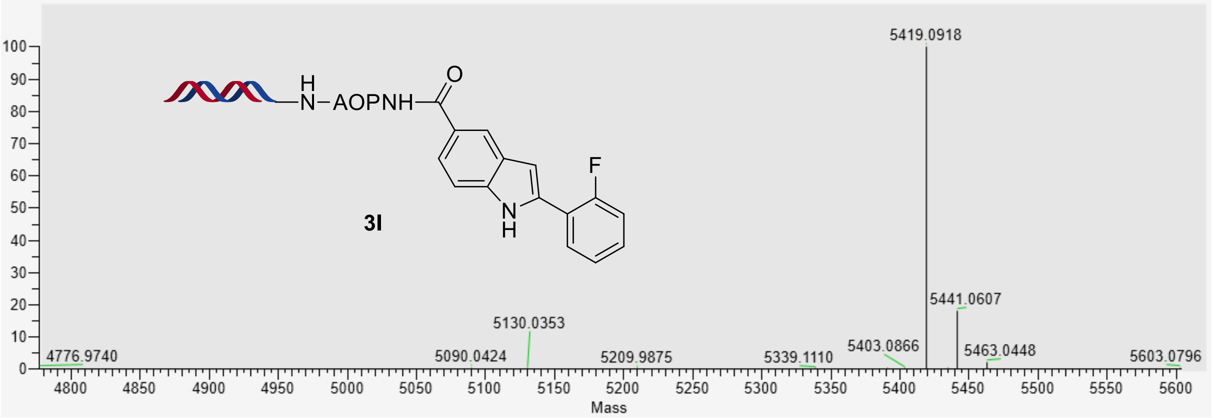

Calcd. for $C_{180}H_{244}FN_{54}O_{107}P_{17}$ 5419.0836; found 5419.0918

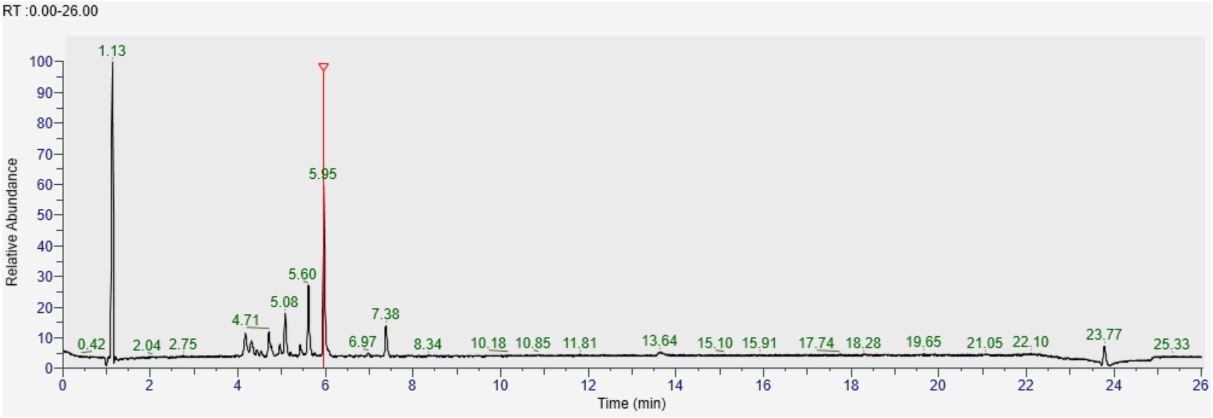

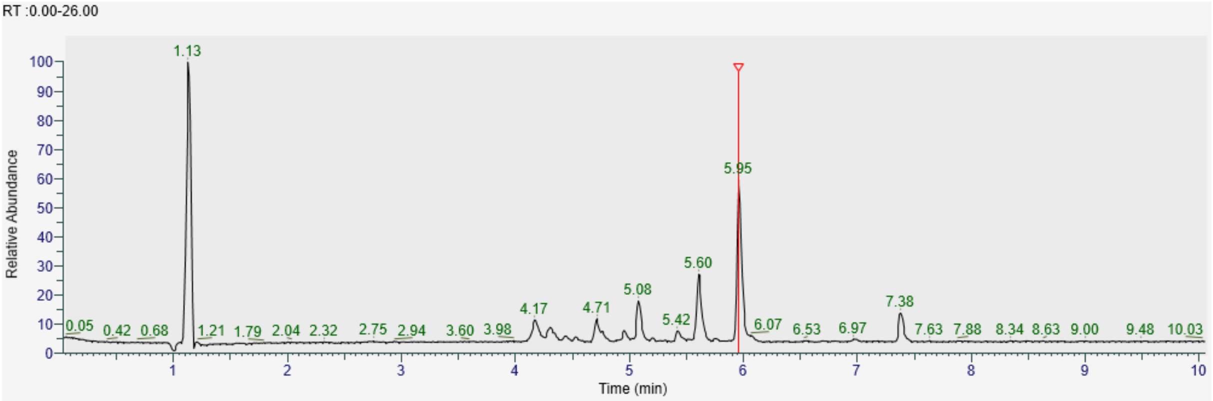

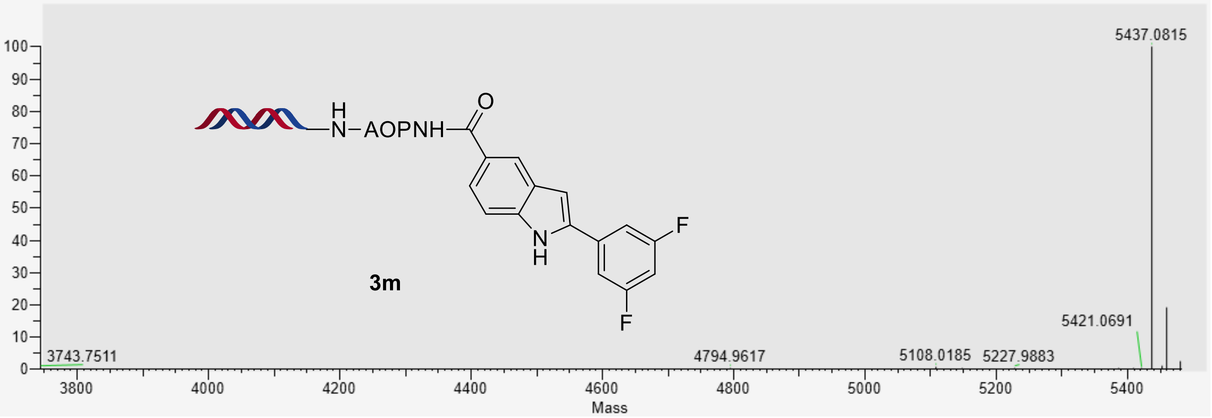

Calcd. for $C_{180}H_{243}F_2N_{54}O_{107}P_{17}$ 5437.0742; found 5437.0815

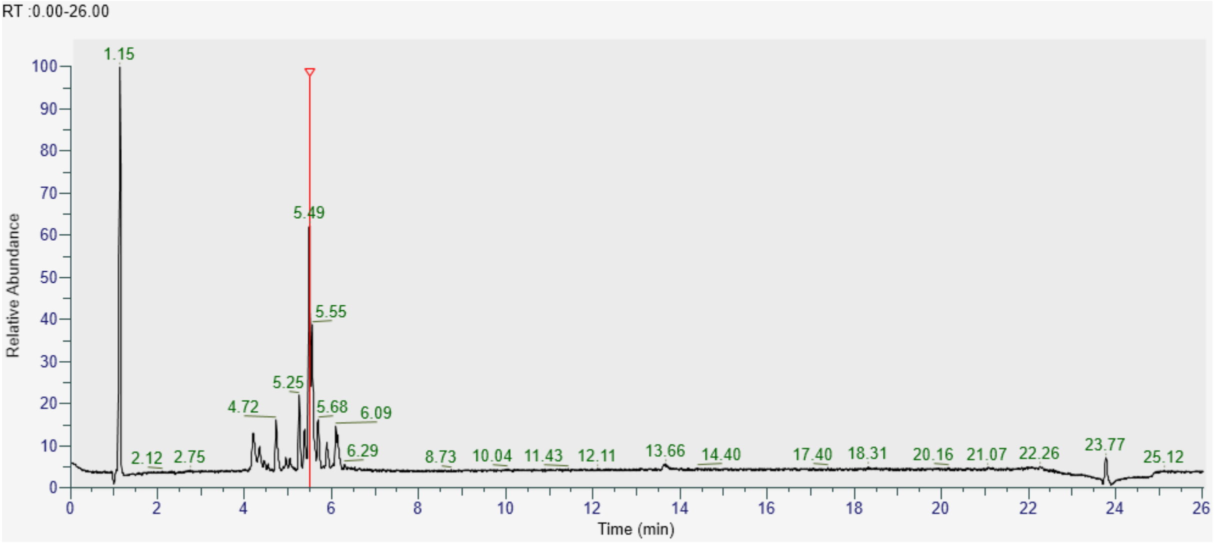

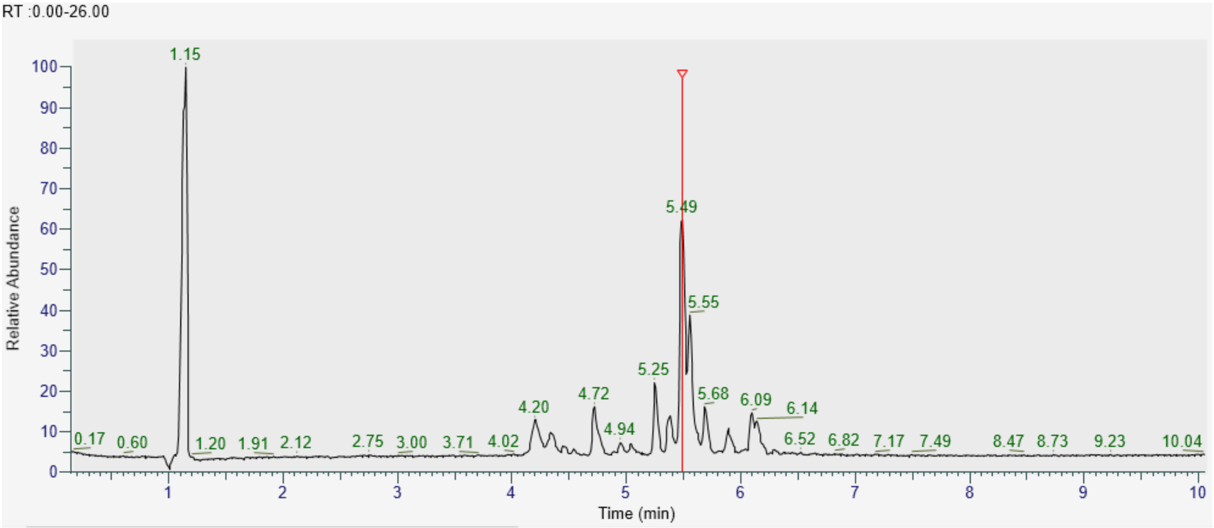

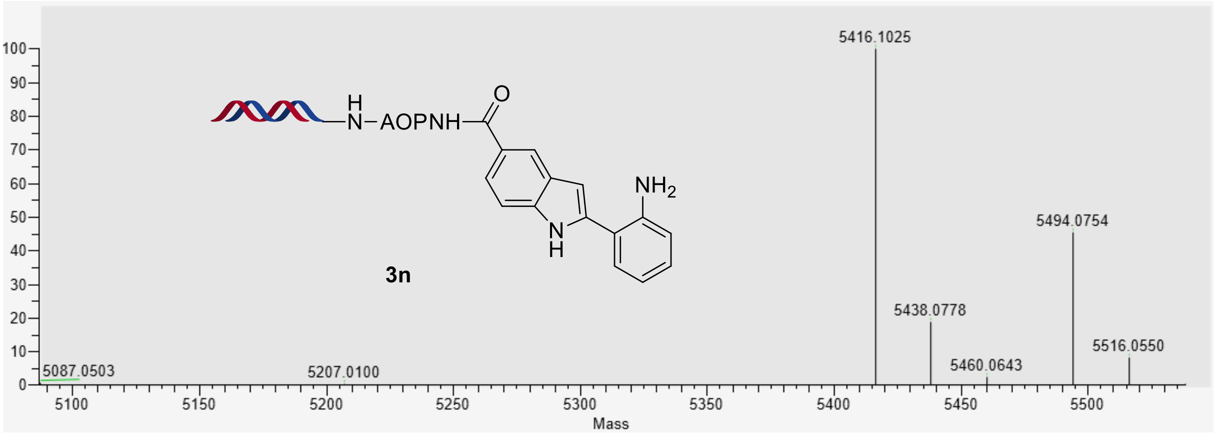

Calcd. for $C_{180}H_{246}N_{55}O_{107}P_{17}$ 5416.1039; found 5416.1009

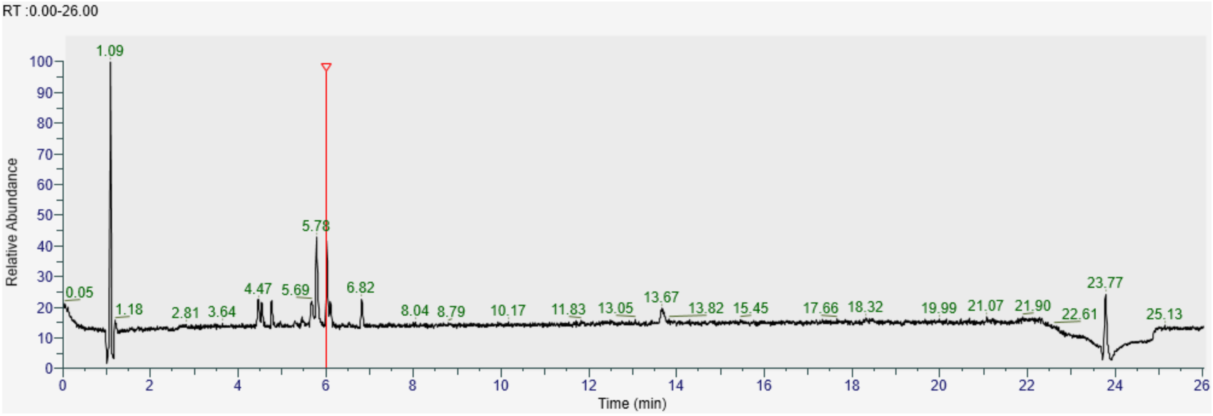

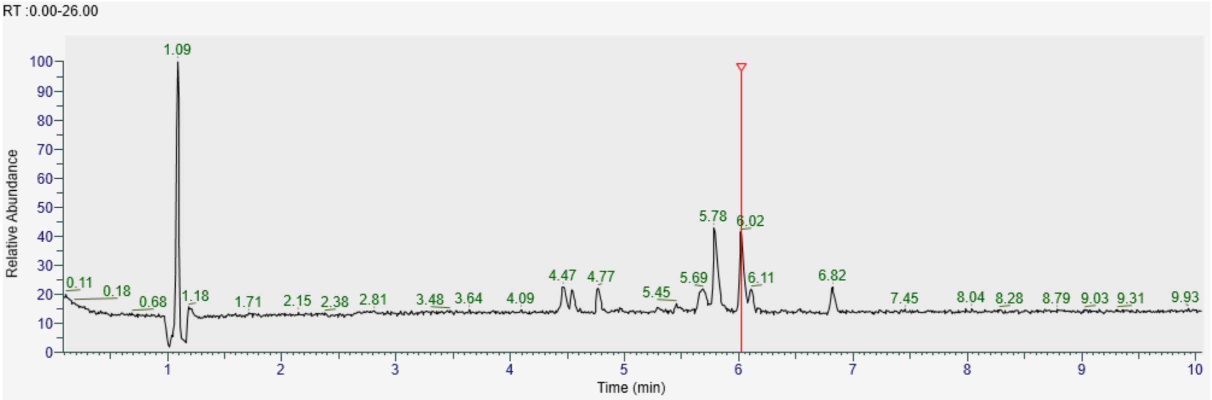

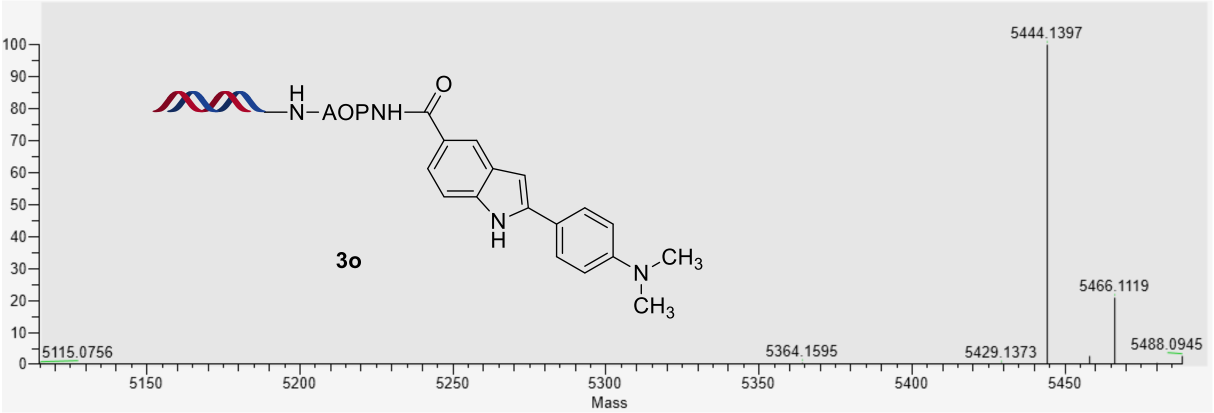

Calcd. for $C_{182}H_{250}N_{55}O_{107}P_{17}$ 5444.1352; found 5444.1397

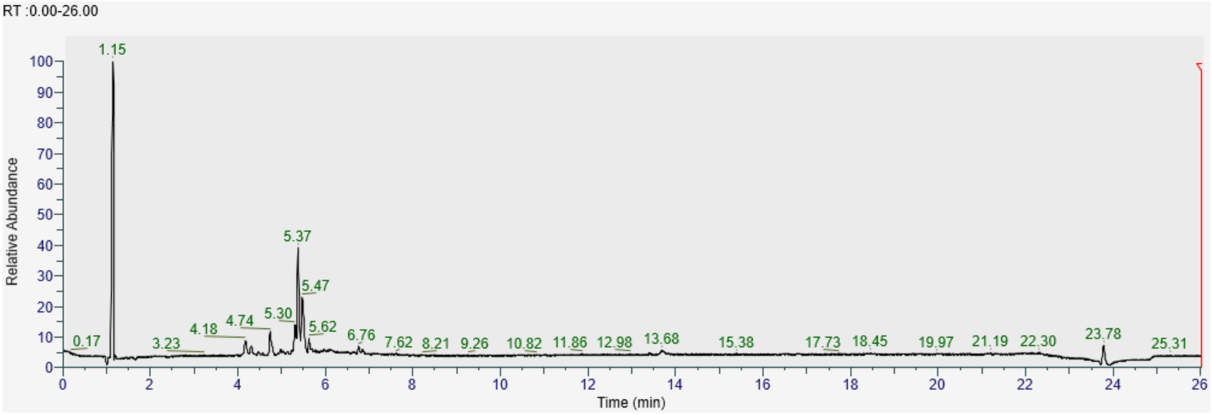

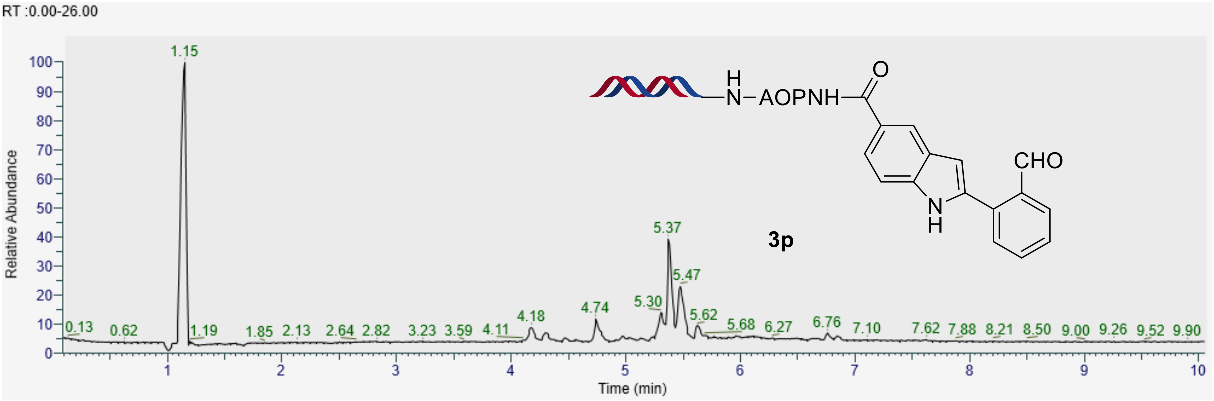

Calcd. for $C_{181}H_{245}N_{54}O_{108}P_{17}$ 5429.0879; No product detected.

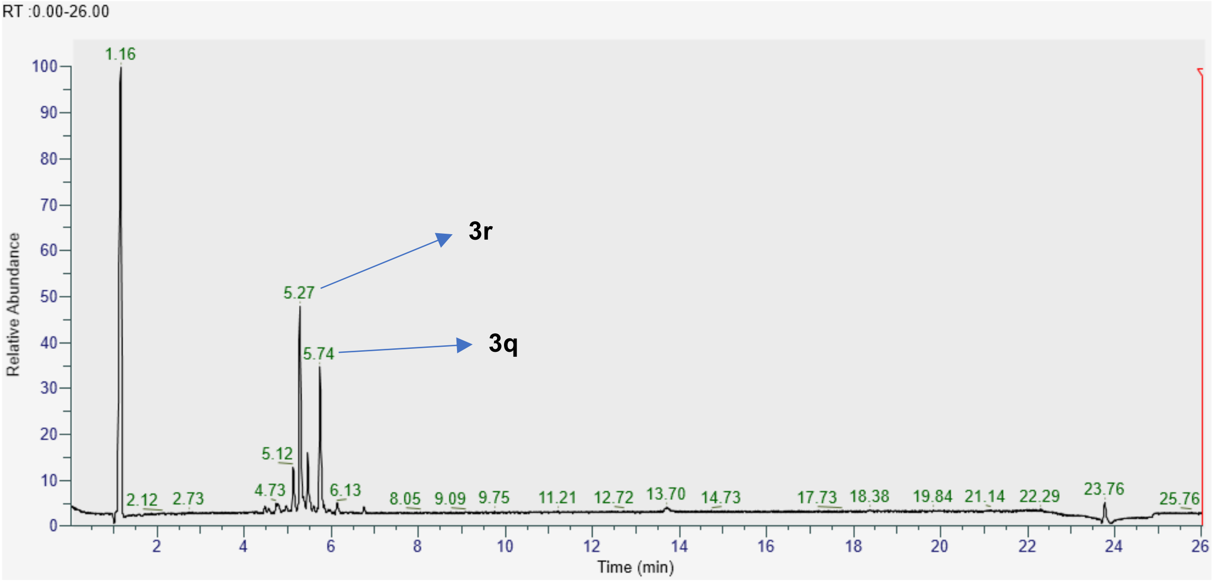
RT :0.00-26.00
Relative Abundance
Time (min)
1.16
5.27
5.74
5.12
4.73
6.13
2.12
2.73
8.05
9.09
9.75
11.21
12.72
13.70
14.73
17.73
18.38
19.84
21.14
22.29
23.76
25.76
3r
3q

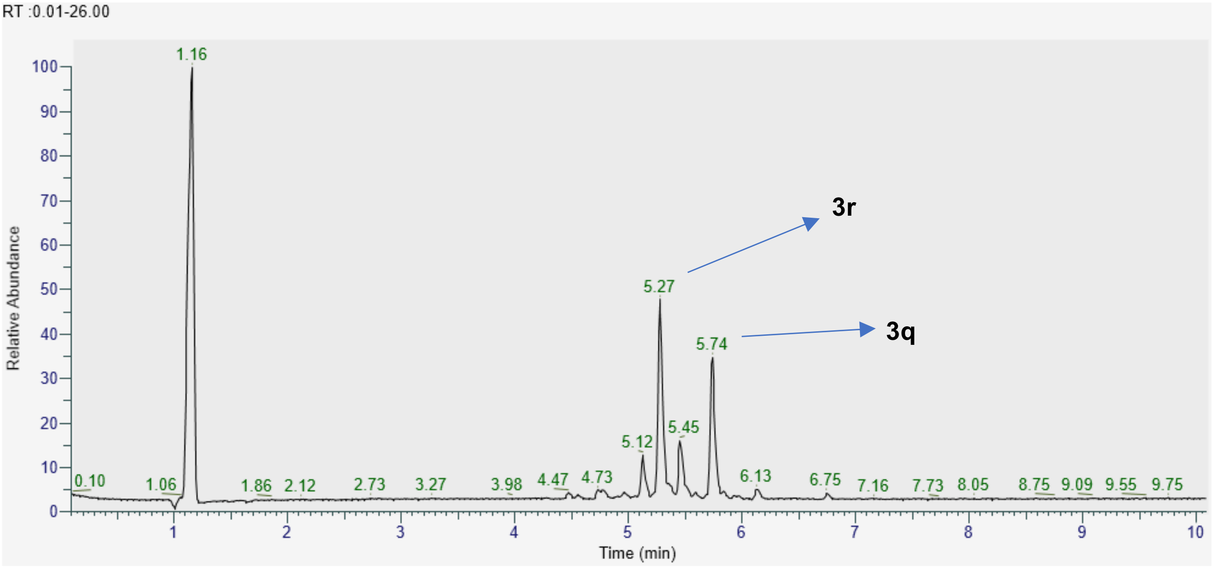
RT :0.01-26.00
Relative Abundance
Time (min)
1.16
5.27
5.74
5.45
5.12
0.10
1.06
1.86
2.12
2.73
3.27
3.98
4.47
4.73
6.13
6.75
7.16
7.73
8.05
8.75
9.09
9.55
9.75
3r
3q

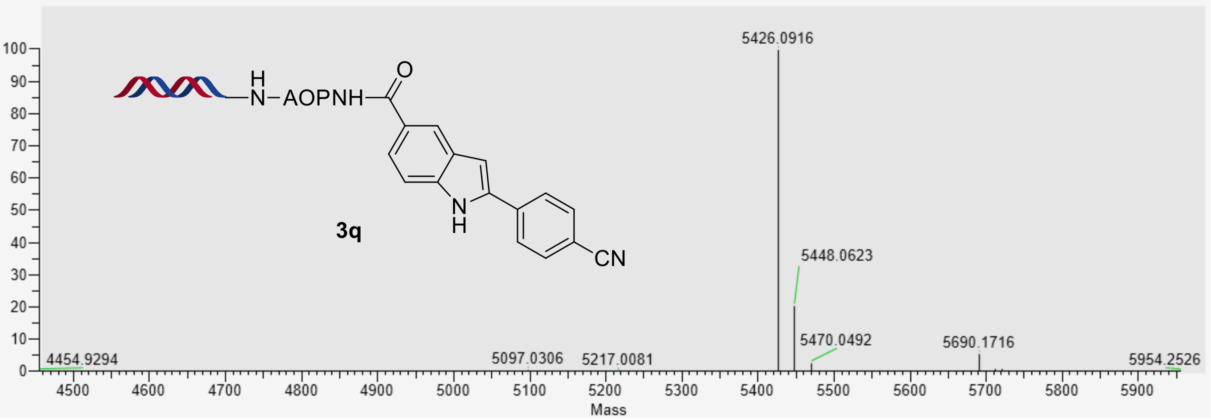

For **3q**: Calcd. for $C_{181}H_{244}N_{55}O_{107}P_{17}$ 5426.0882; found 5426.0916

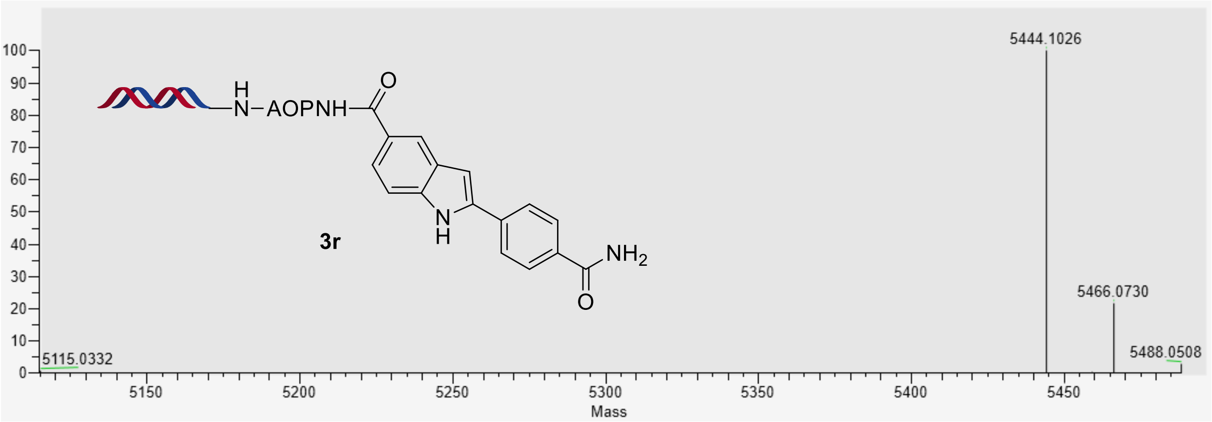

For **3r**: Calcd. for $C_{181}H_{246}N_{55}O_{108}P_{17}$ 5444.0988; found 5444.1026

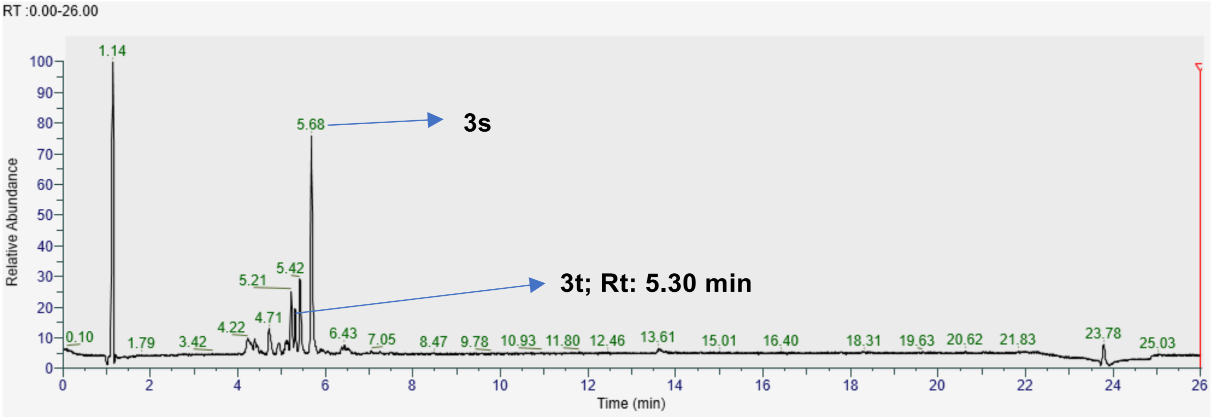
RT :0.00-26.00
Relative Abundance
Time (min)
3s
3t; Rt: 5.30 min

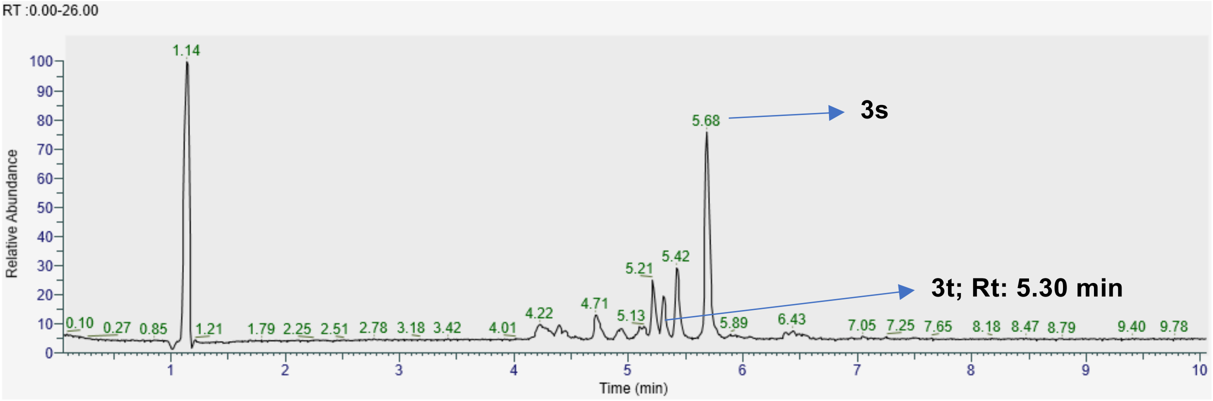
RT :0.00-26.00
Relative Abundance
Time (min)
3s
3t; Rt: 5.30 min

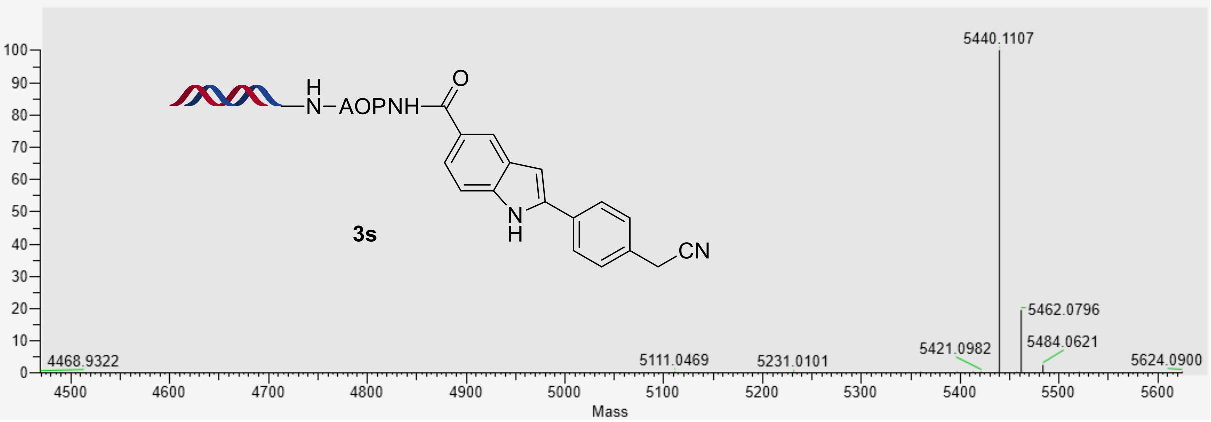

For **3s**: Calcd. for $C_{182}H_{246}N_{55}O_{107}P_{17}$ 5440.1039; found 5440.1107

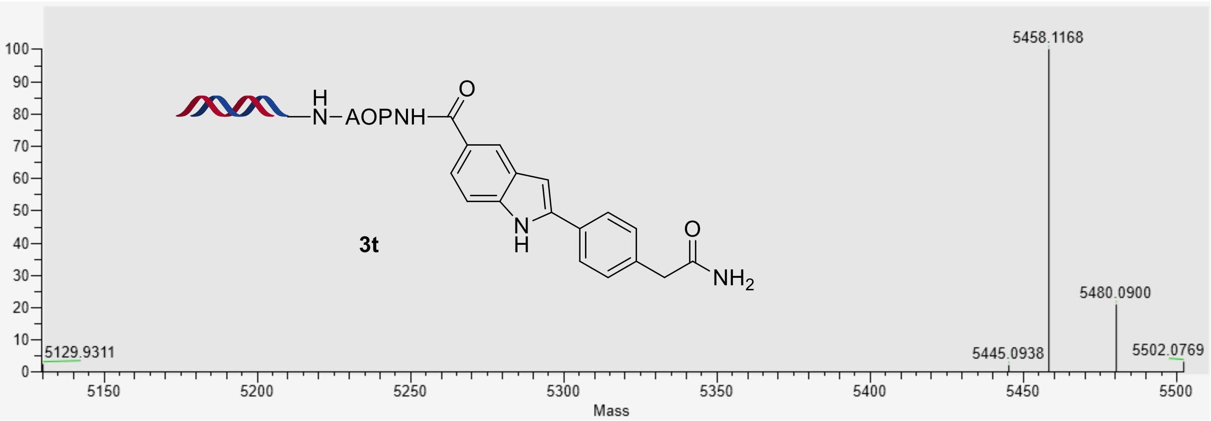

For **3t**: Calcd. for $C_{182}H_{248}N_{55}O_{108}P_{17}$ 5458.1145; found 5458.1168

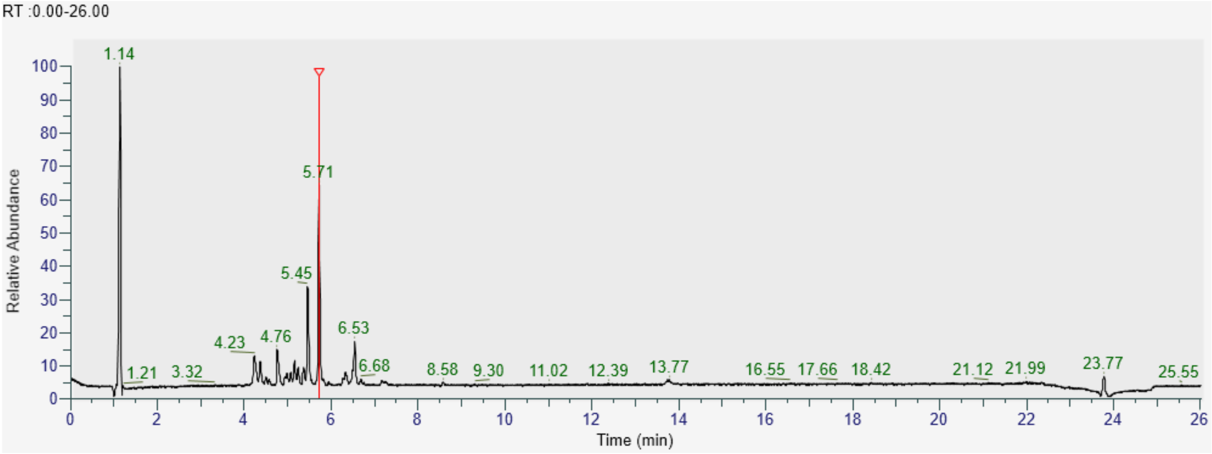

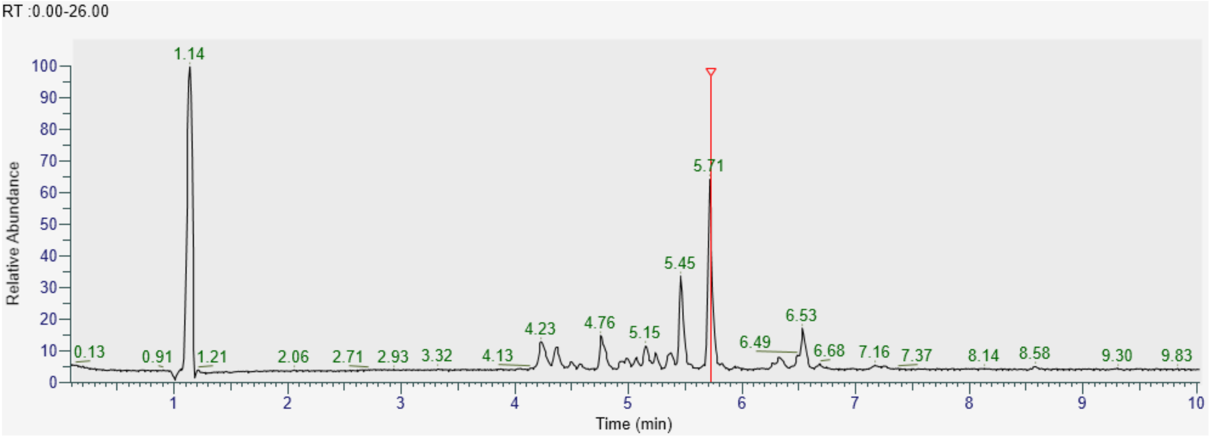

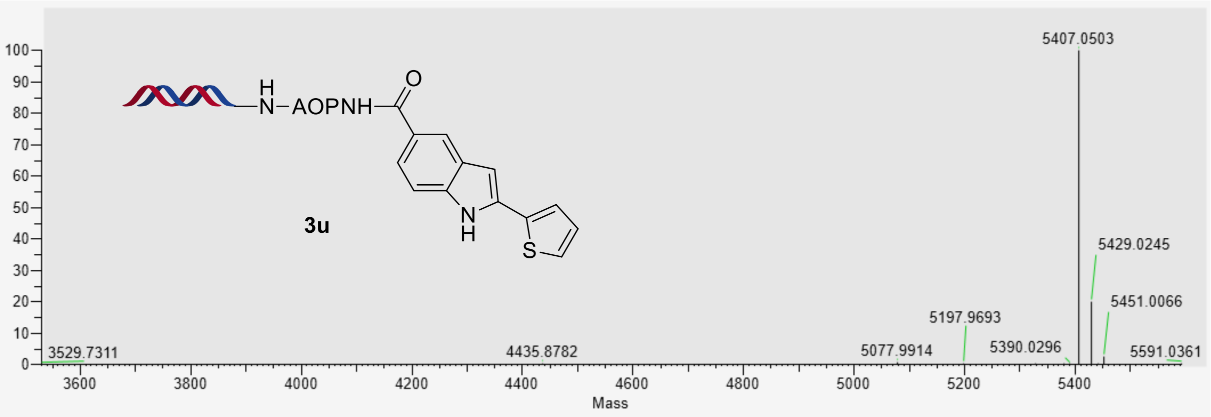

Calcd. for: $C_{178}H_{243}N_{54}O_{107}P_{17}S$ 5407.0494; found 5407.0503

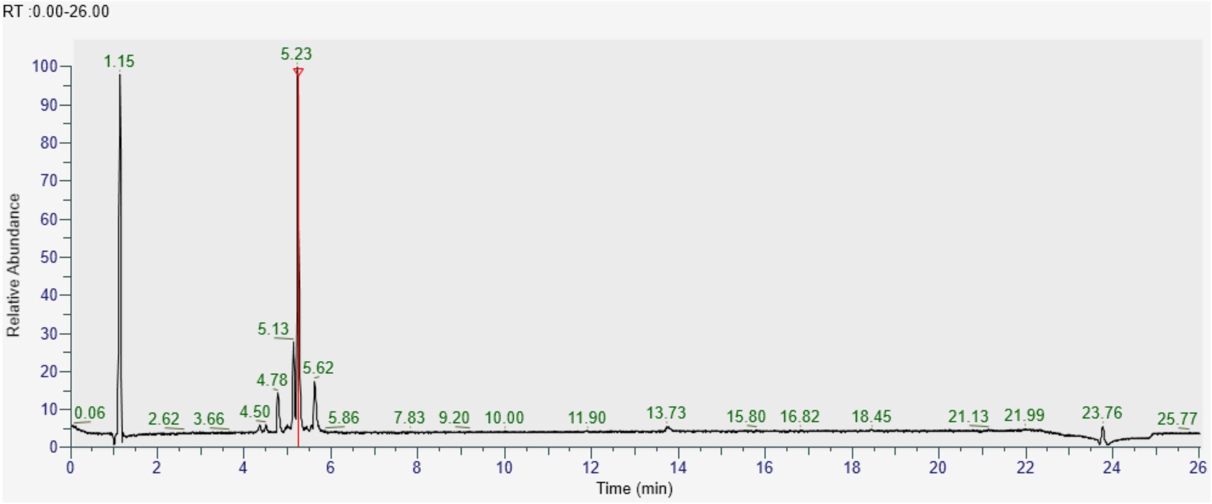

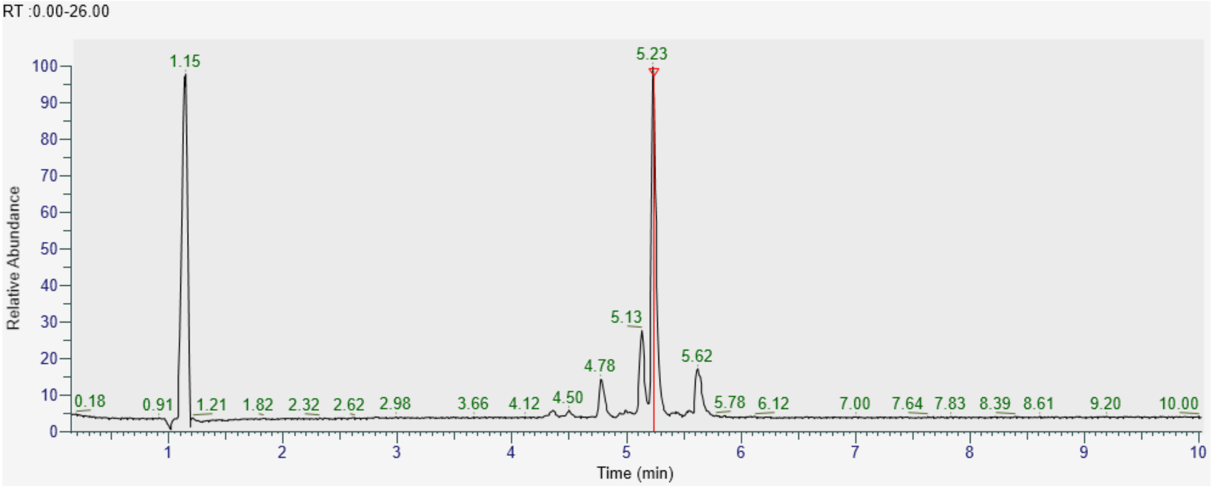

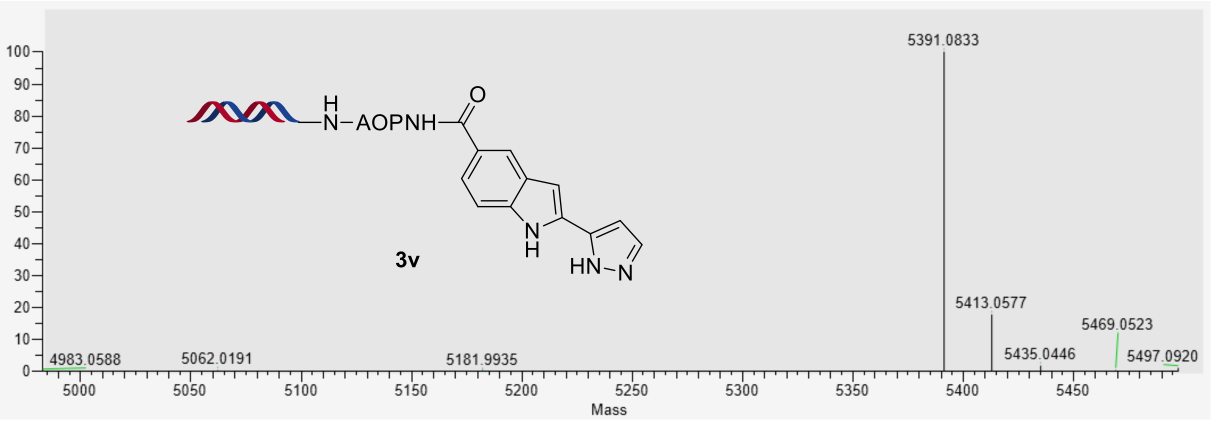

Calcd. for $C_{177}H_{243}N_{56}O_{107}P_{17}$ 5391.0835; found 5391.0833

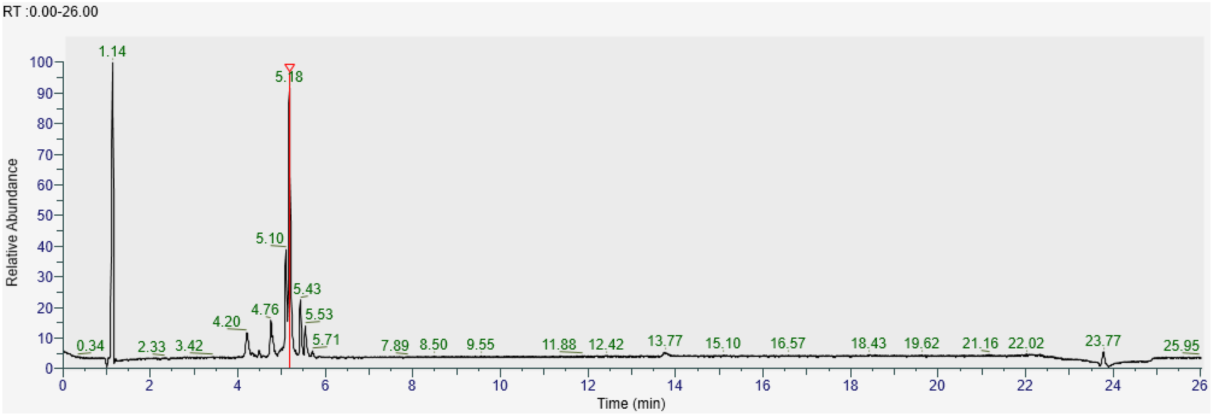

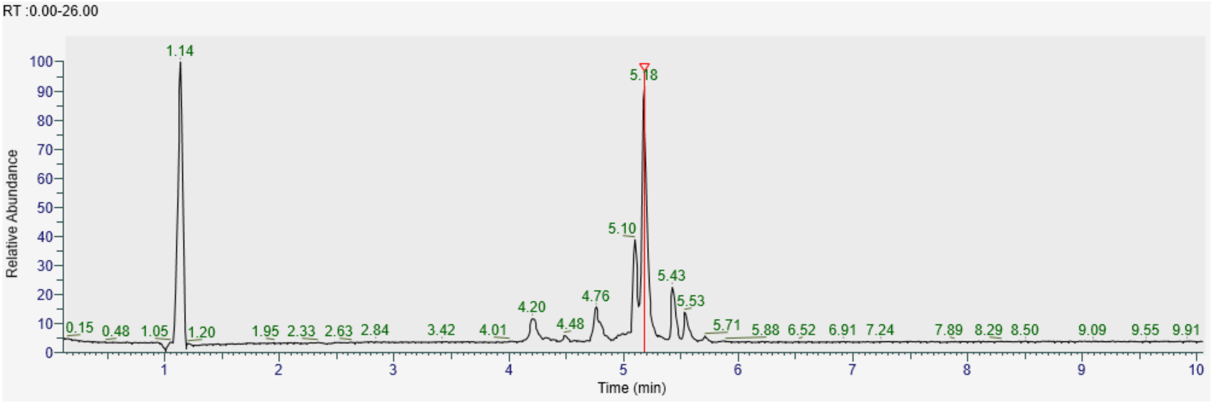

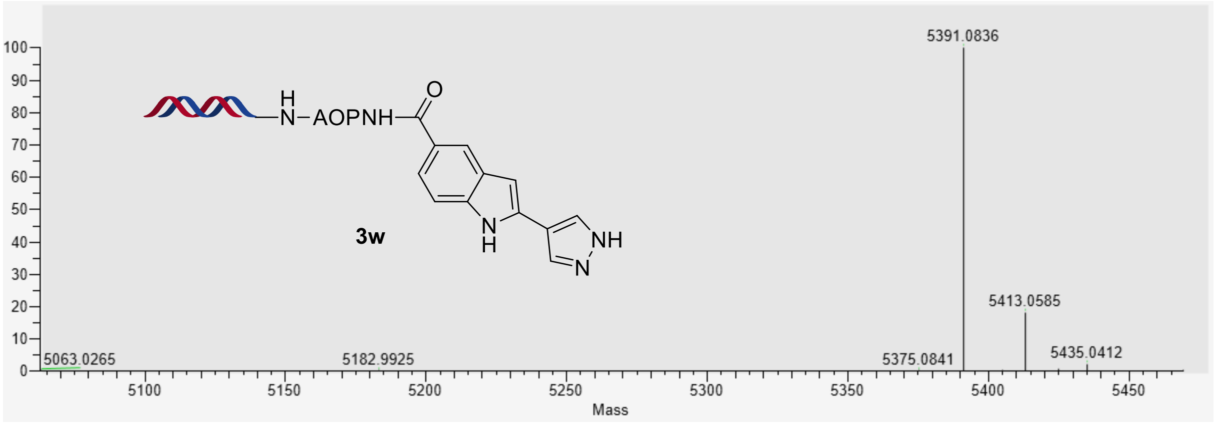

Calcd. for $C_{177}H_{243}N_{56}O_{107}P_{17}$ 5391.0835; found 5391.0836

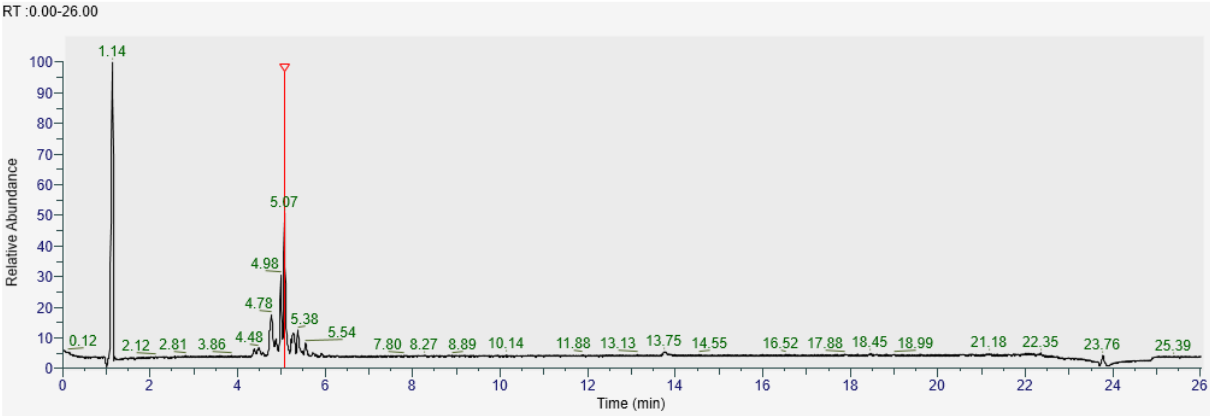

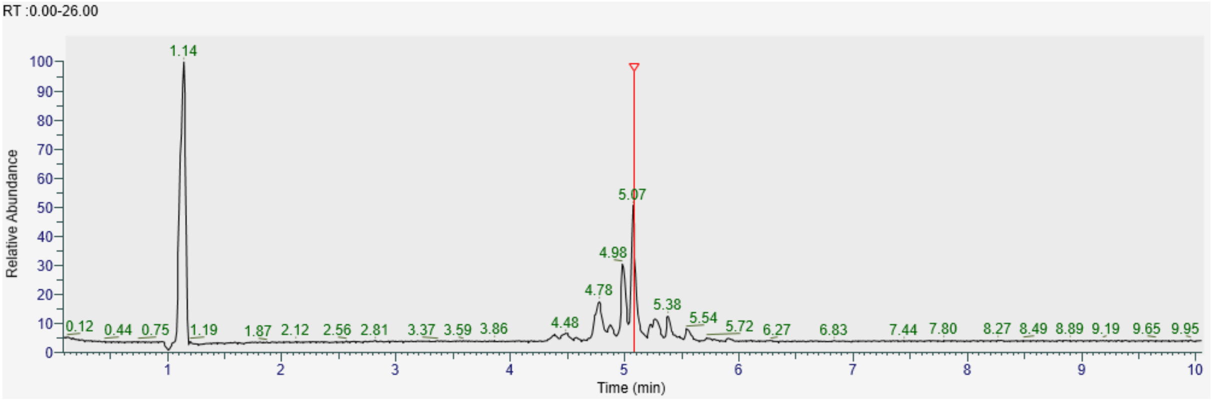

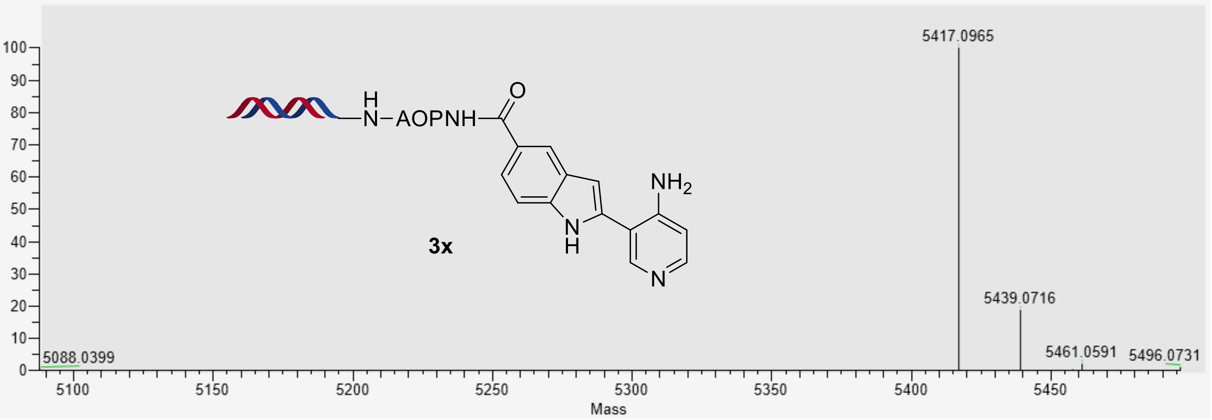

Calcd. for $C_{179}H_{245}N_{56}O_{107}P_{17}$ 5417.0991; found 5417.0965

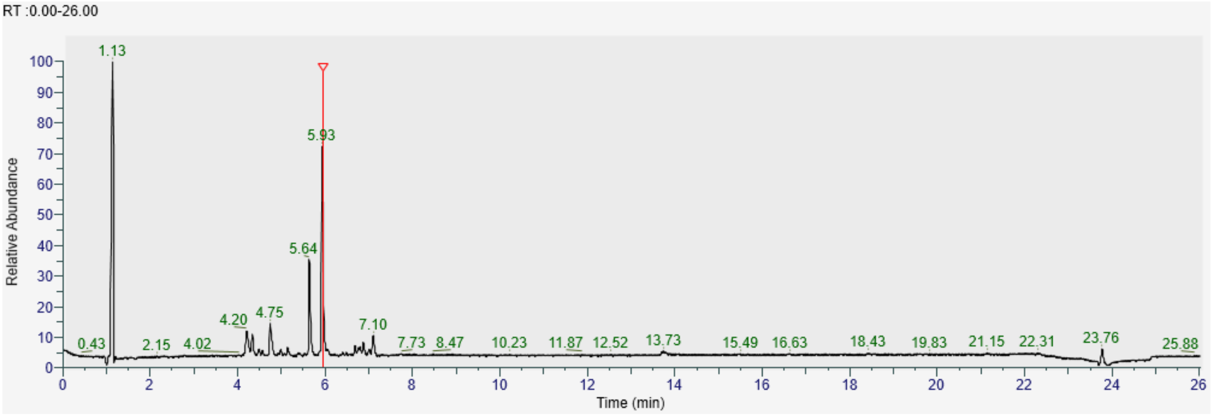

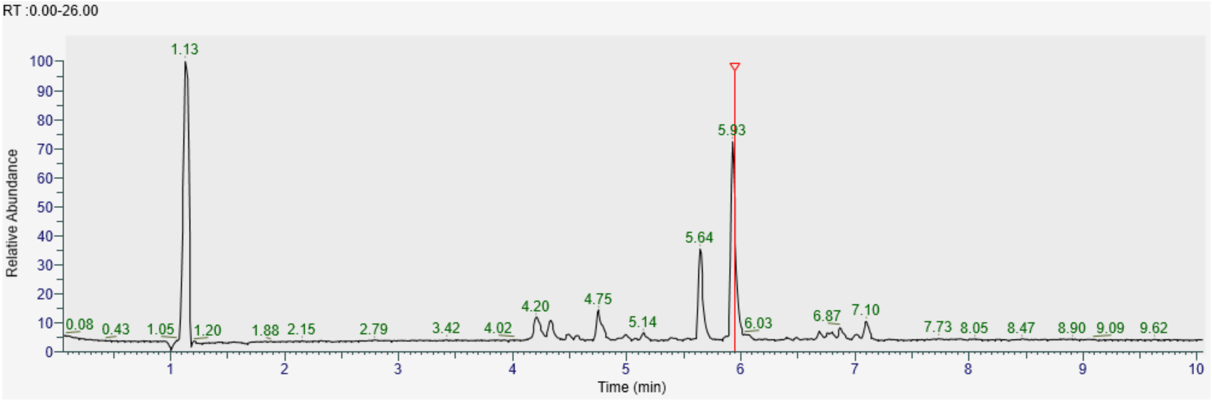

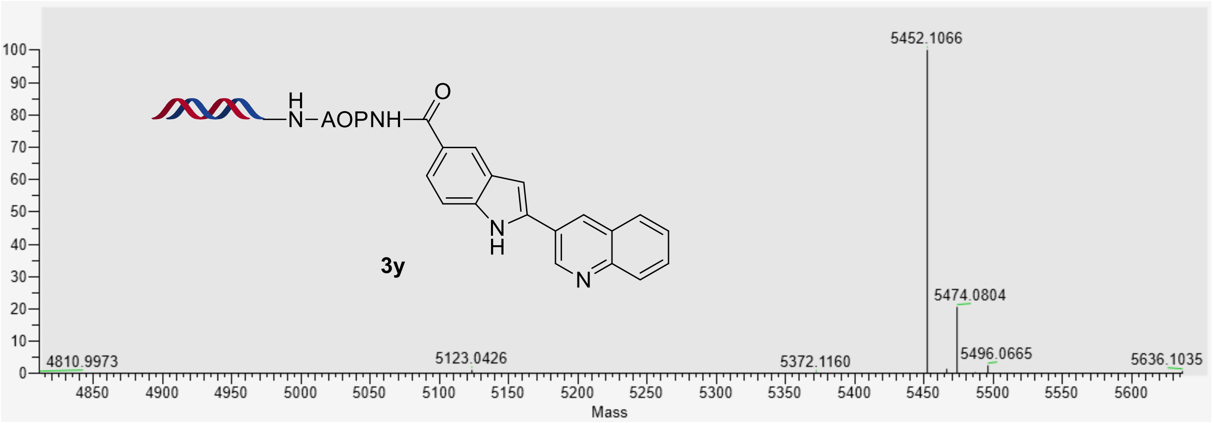

Calcd. for $C_{183}H_{246}N_{55}O_{107}P_{17}$ 5452.1039; found 5452.1066

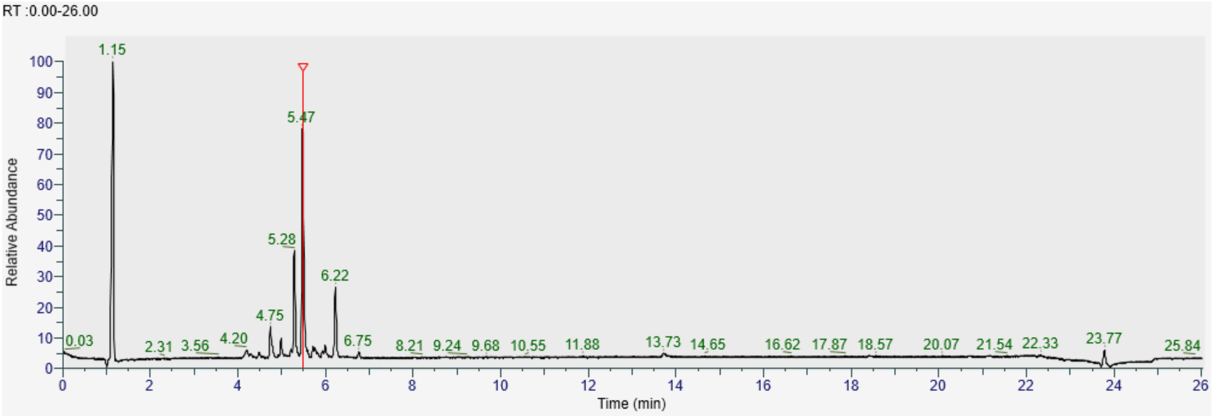

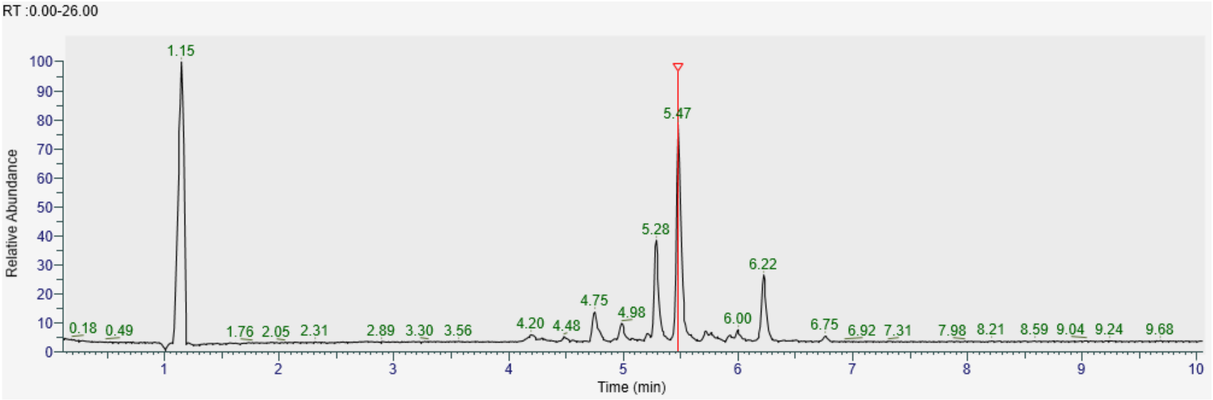

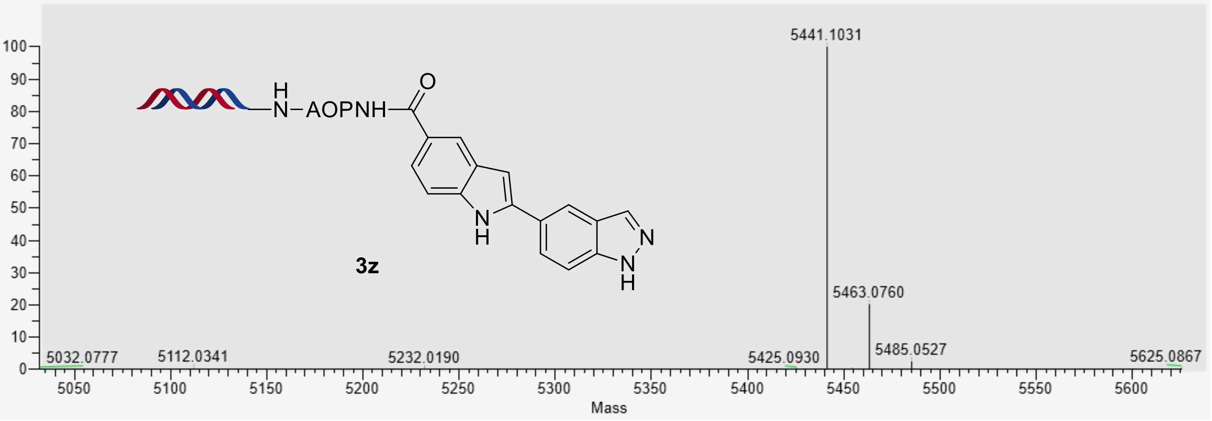

Calcd. for $C_{181}H_{245}N_{56}O_{107}P_{17}$ 5441.0991; found 5441.1031

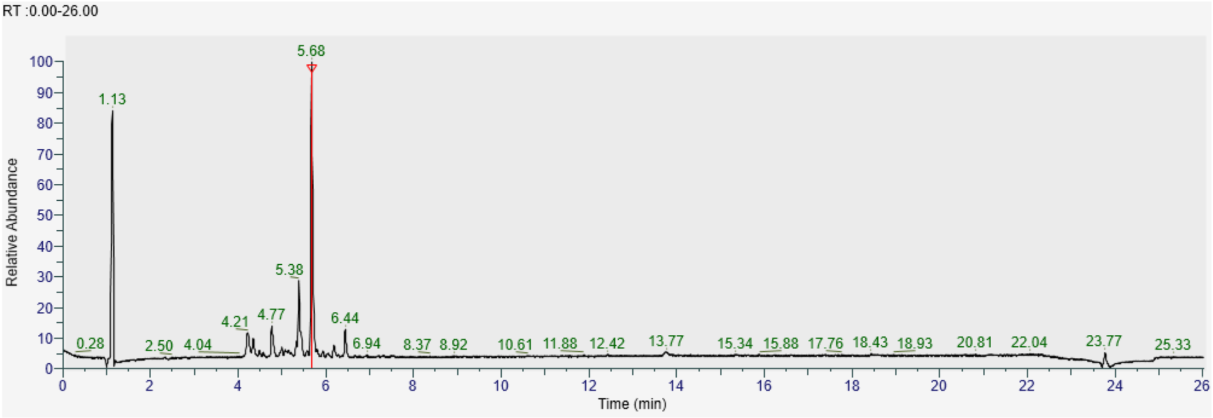

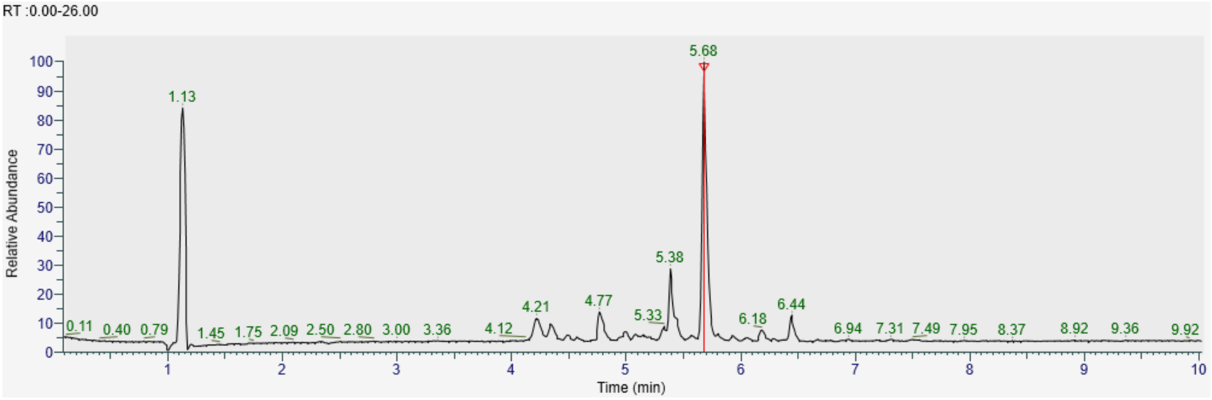

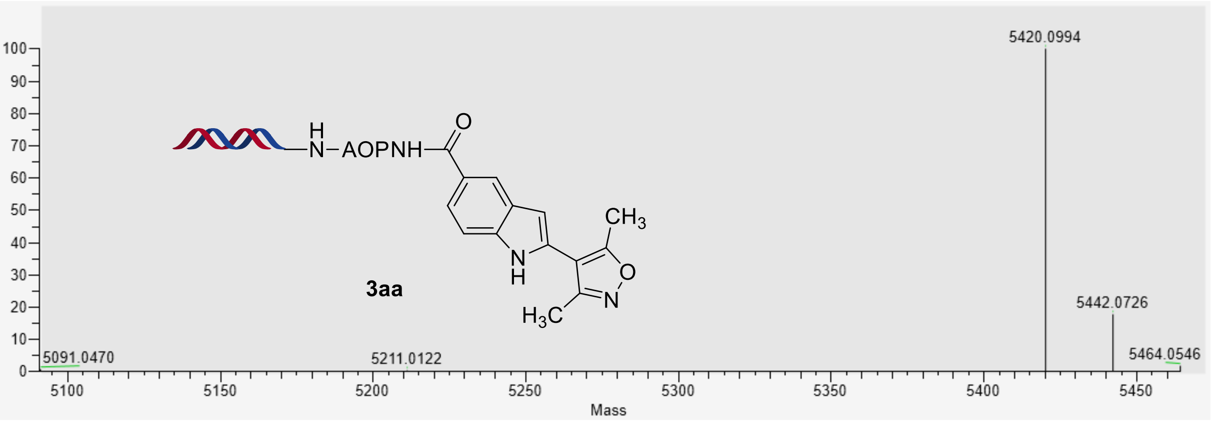

Calcd. for $C_{179}H_{246}N_{55}O_{108}P_{17}$ 5420.0988; found 5420.0994

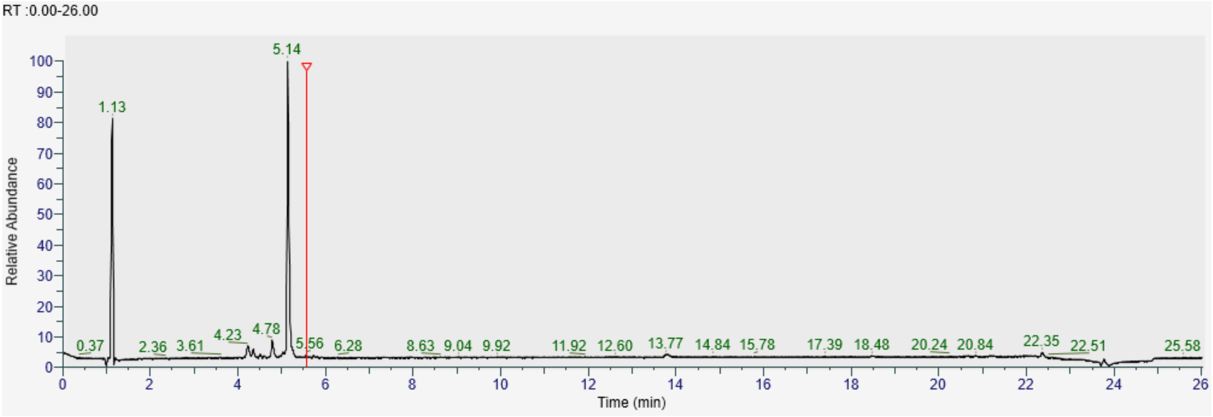

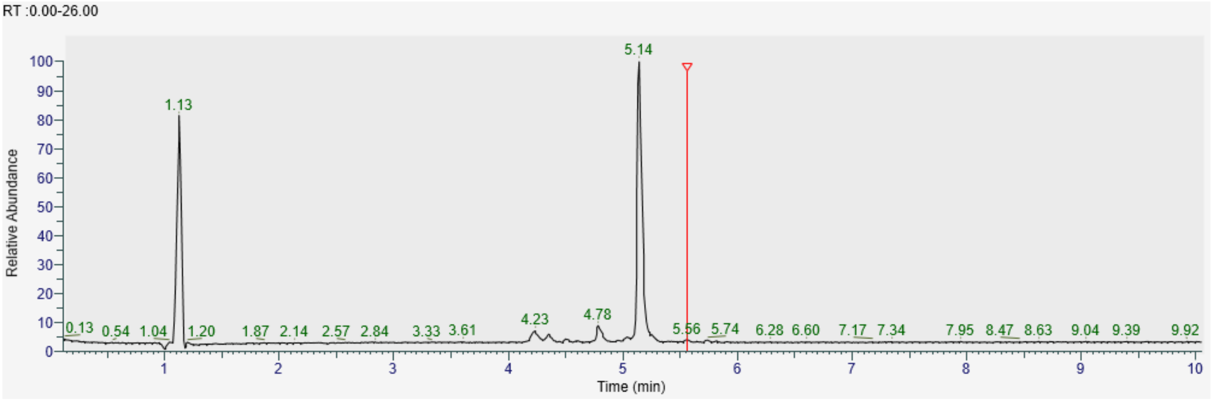

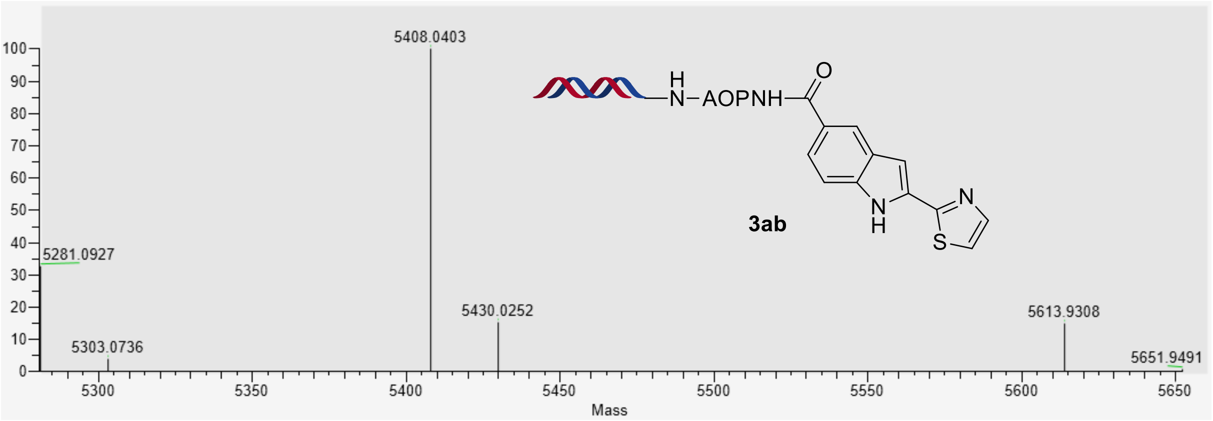

Calcd. for $C_{177}H_{242}N_{55}O_{107}P_{17}S$ 5408.0447; found 5408.0403

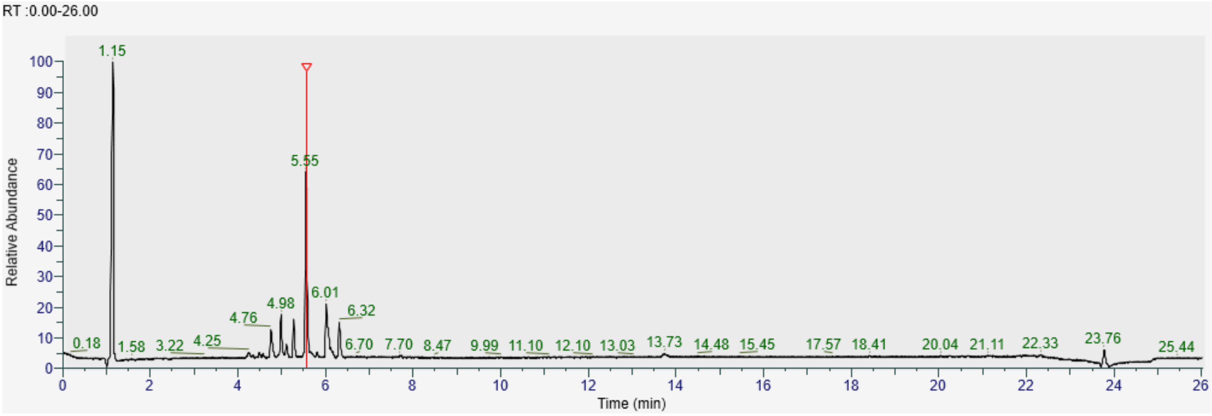

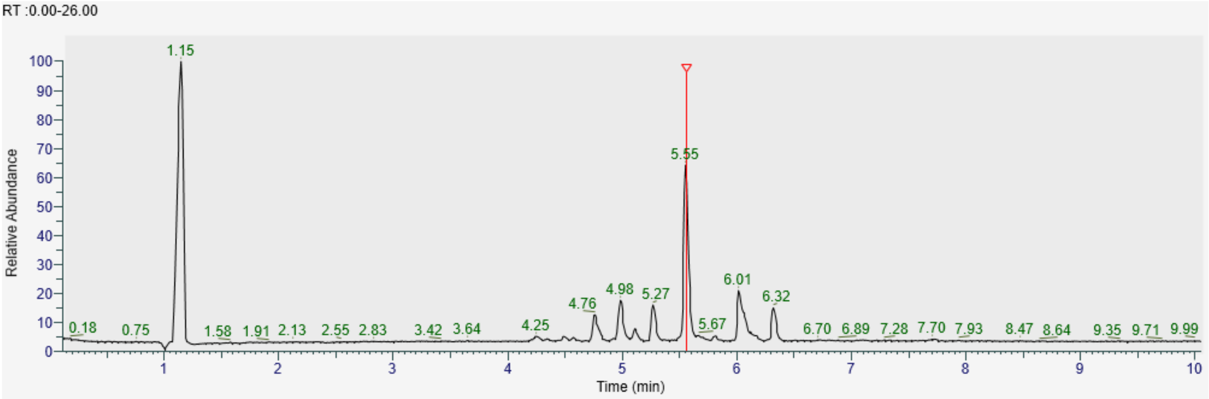

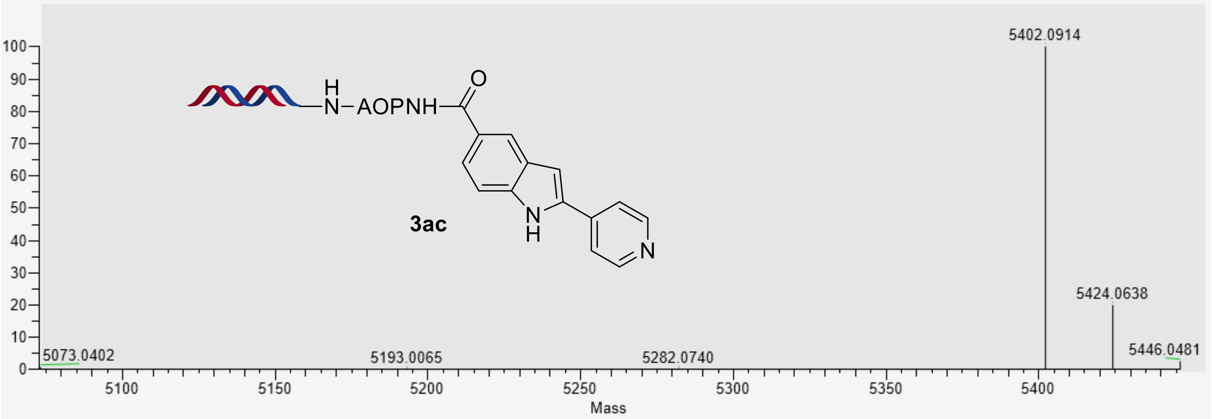

Calcd. for $C_{179}H_{244}N_{55}O_{107}P_{17}$ 5402.0882; found 5402.0914

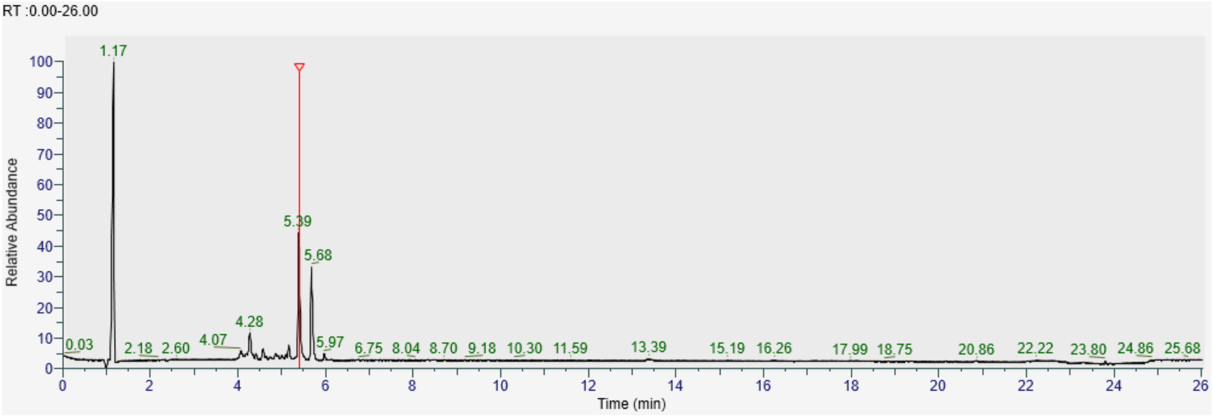

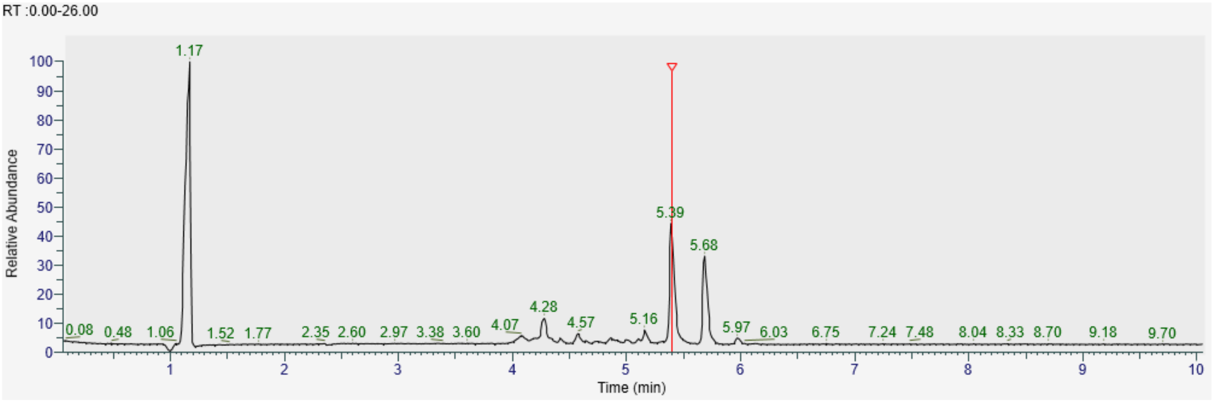

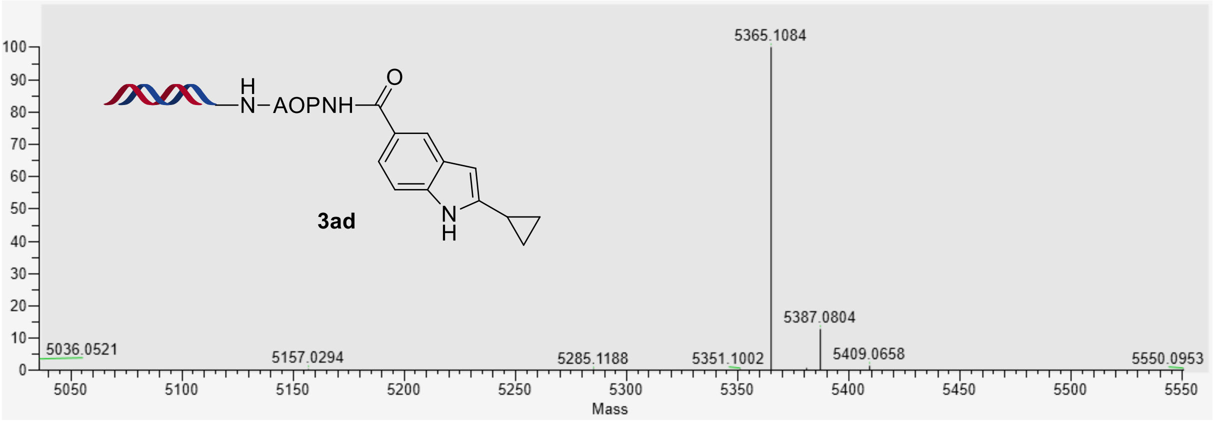

Calcd. for $C_{177}H_{245}N_{54}O_{107}P_{17}$ 5365.0930; found 5365.1084

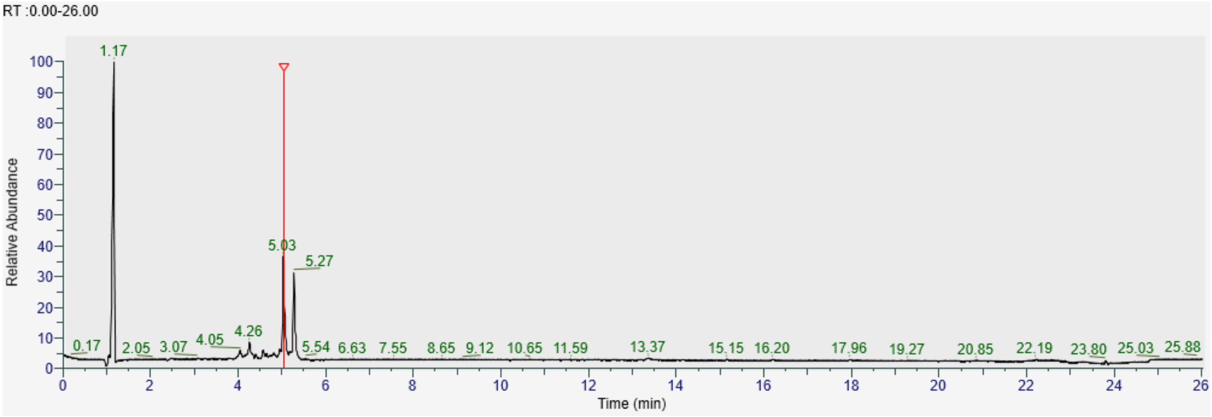

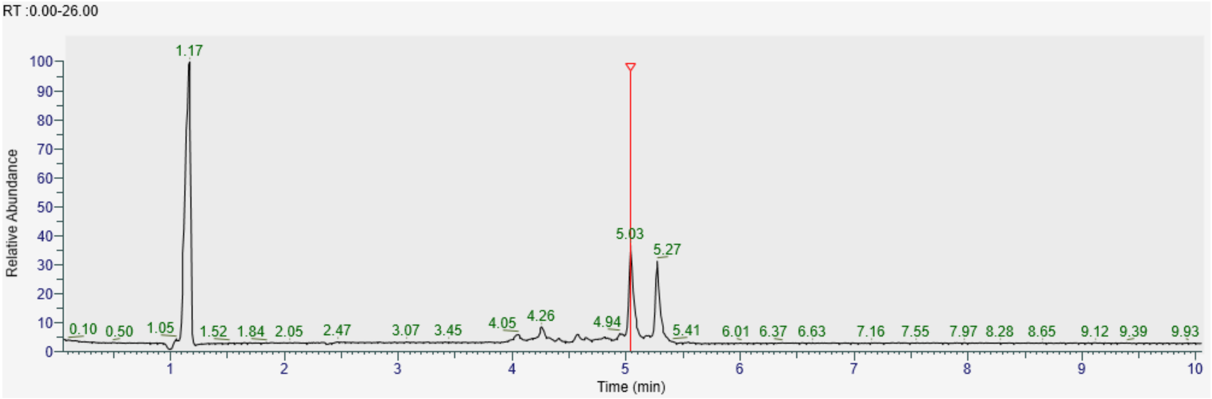

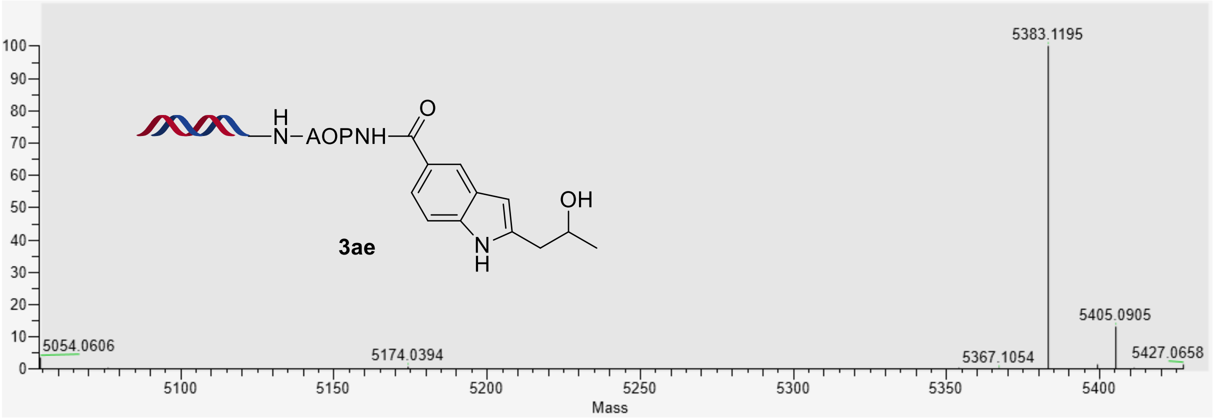

Calcd. for $C_{177}H_{247}N_{54}O_{108}P_{17}$ 5383.1036; found 5383.1195

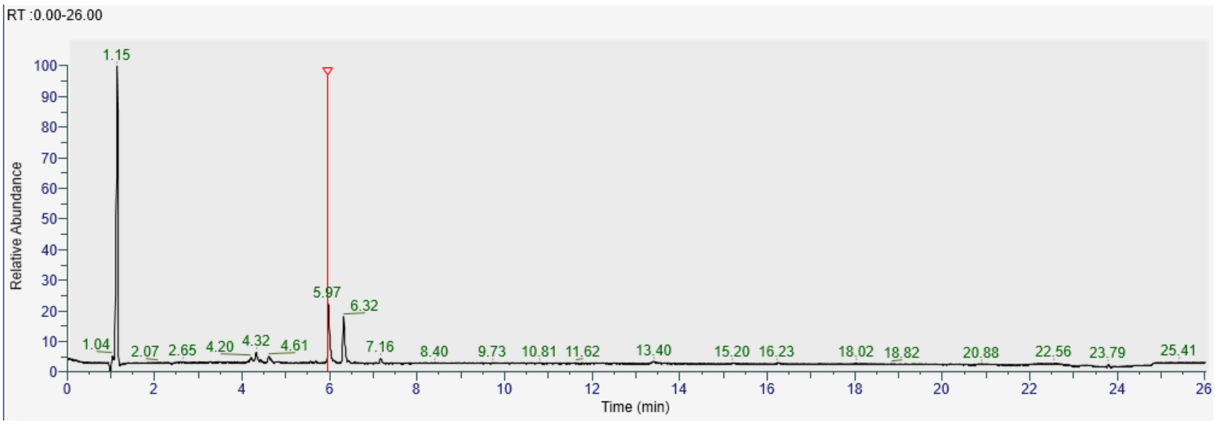

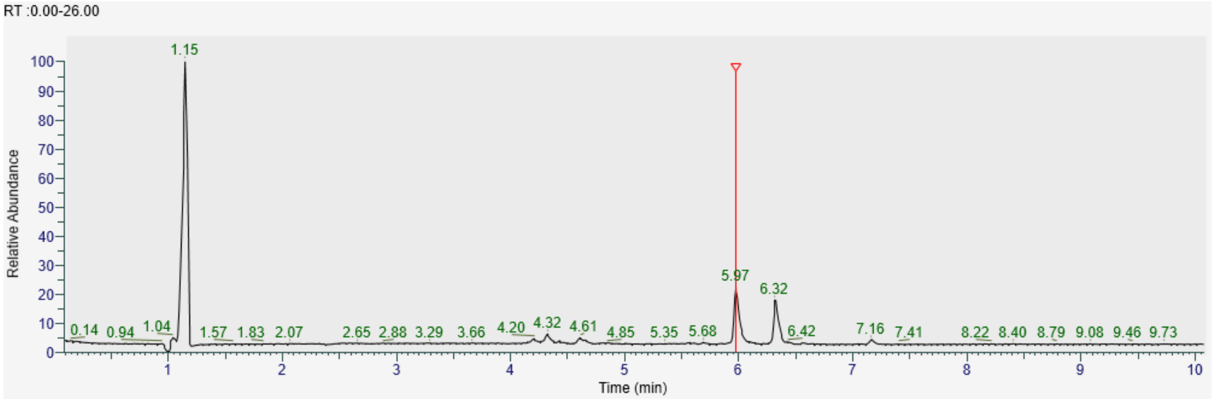

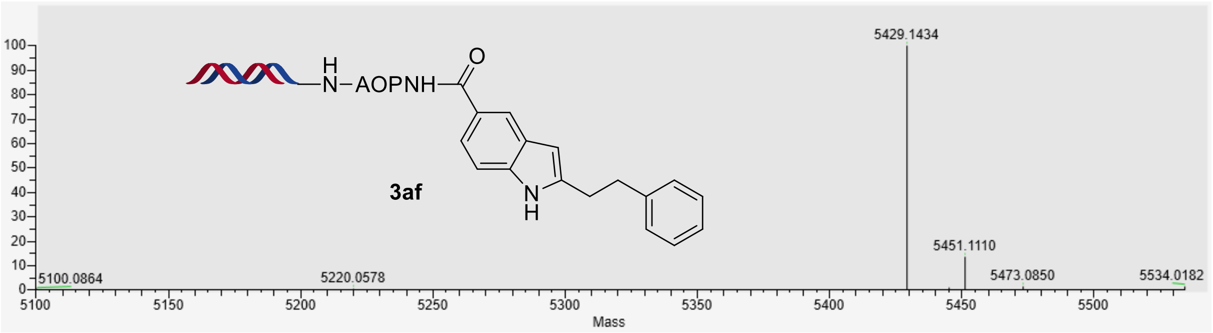

Calcd. for $C_{182}H_{249}N_{54}O_{107}P_{17}$ 5429.1242; found 5429.1434

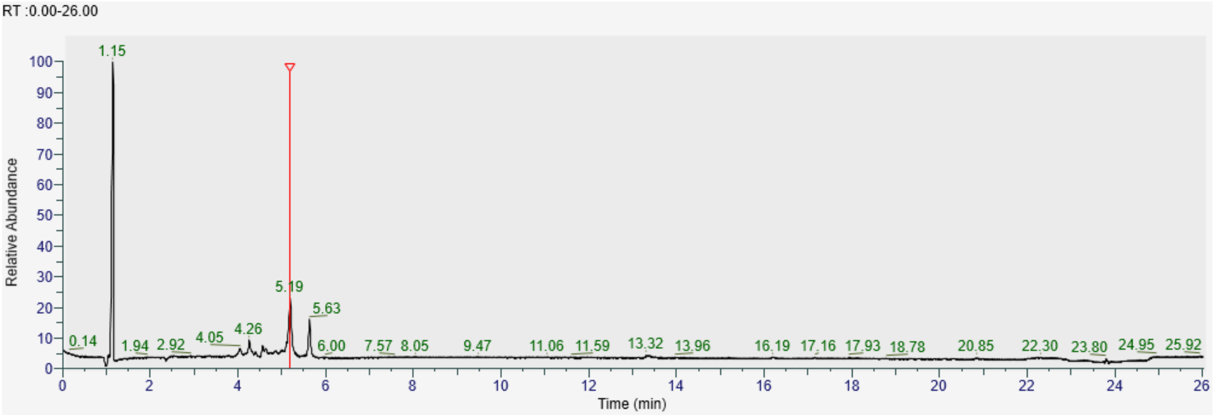

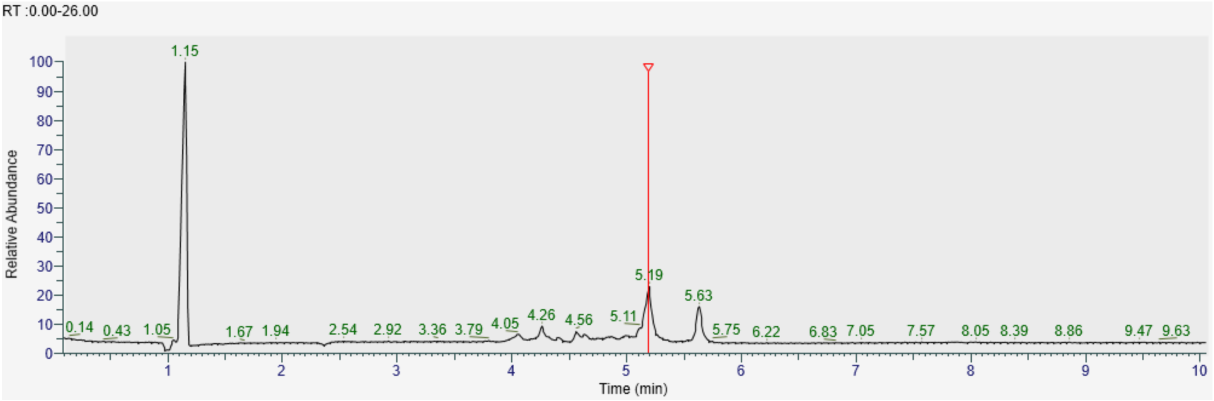

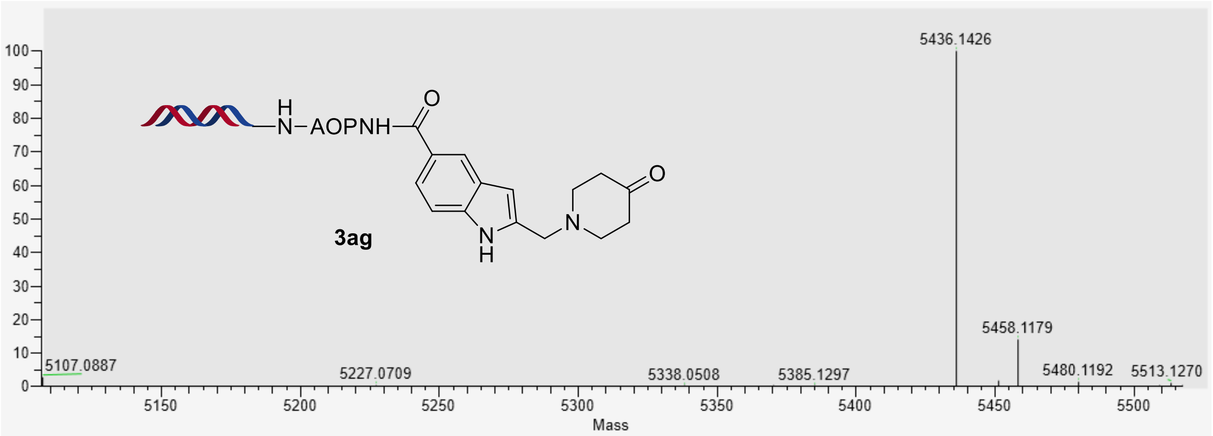

Calcd. for $C_{180}H_{250}N_{55}O_{108}P_{17}$ 5436.1301; found 5436.1426

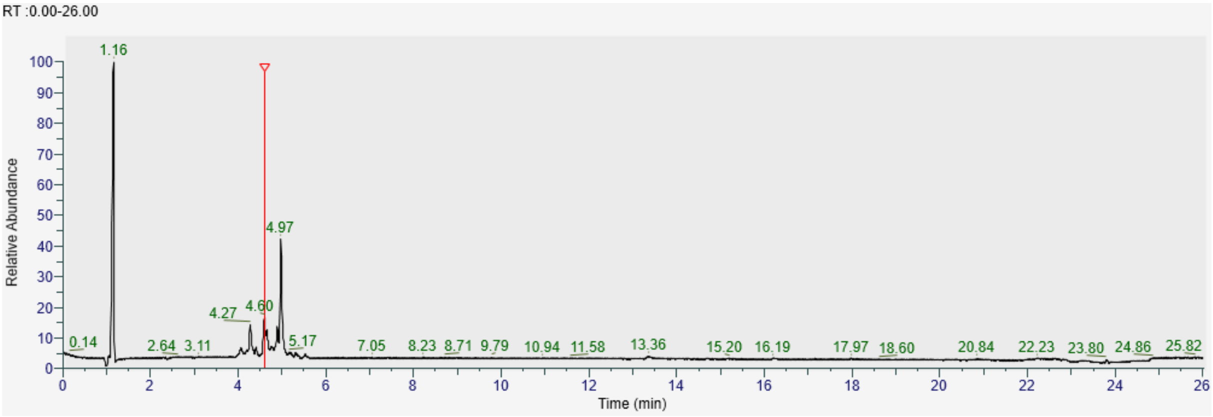

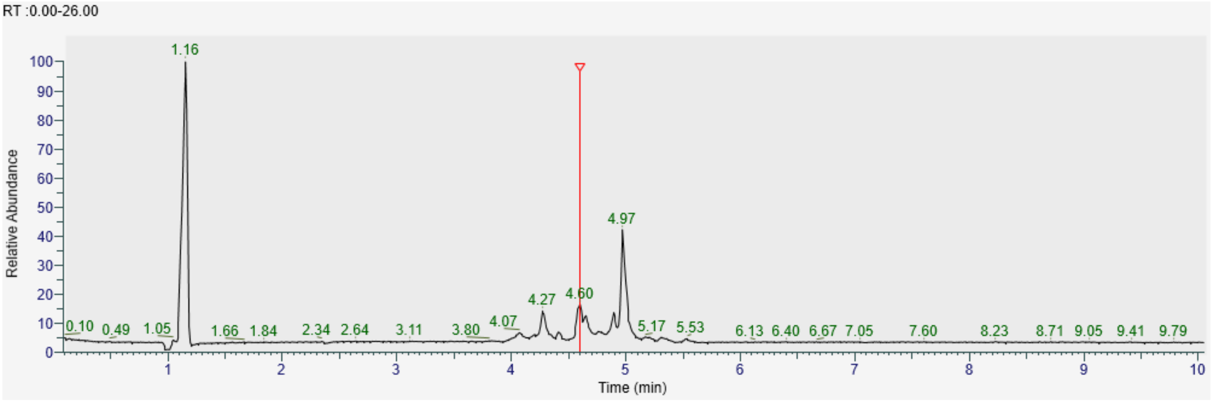

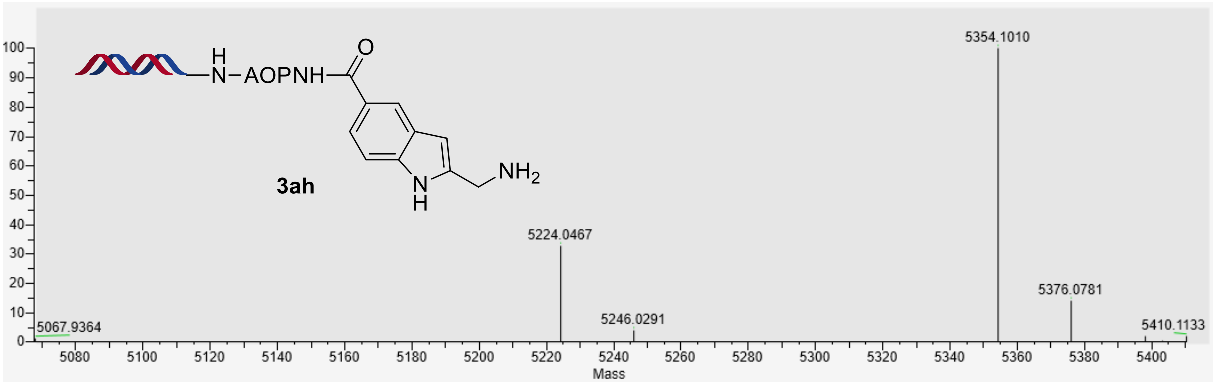

Calcd. for $C_{175}H_{244}N_{55}O_{107}P_{17}$ 5354.0882; found 5354.1010

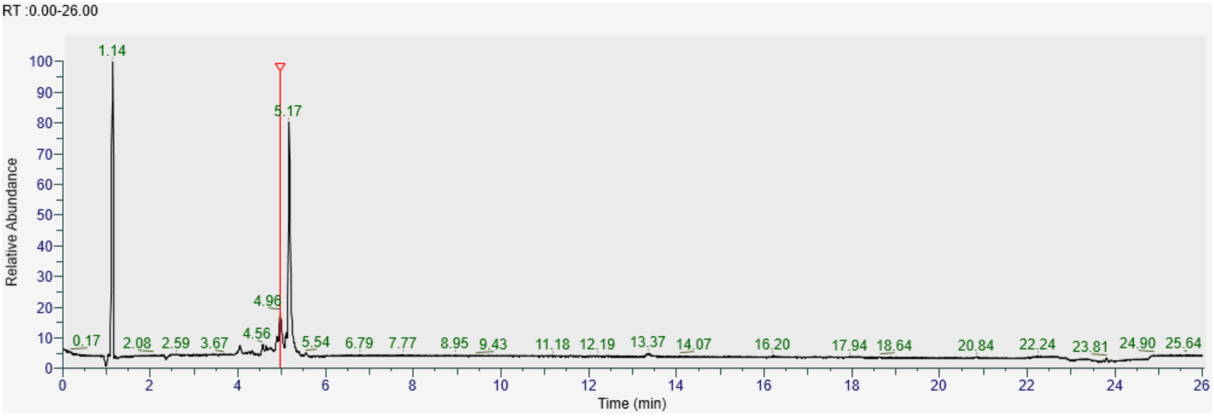

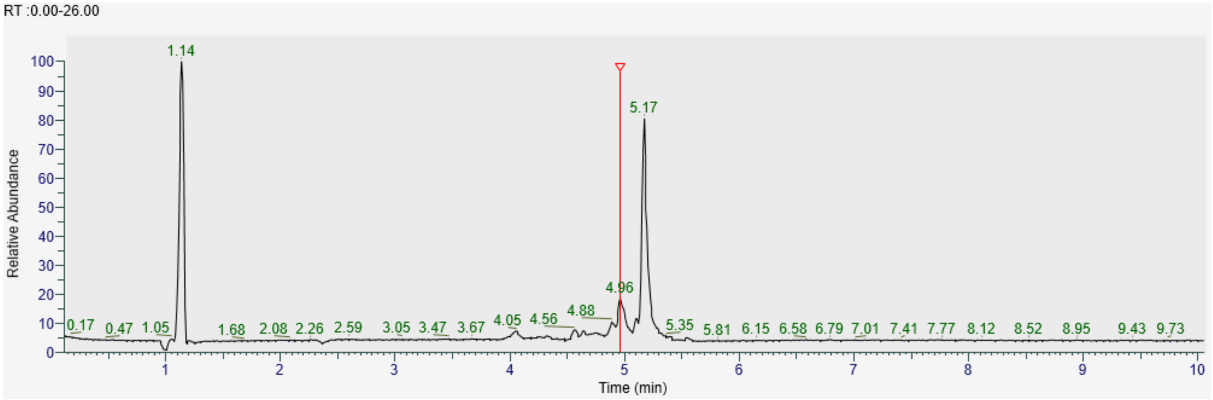

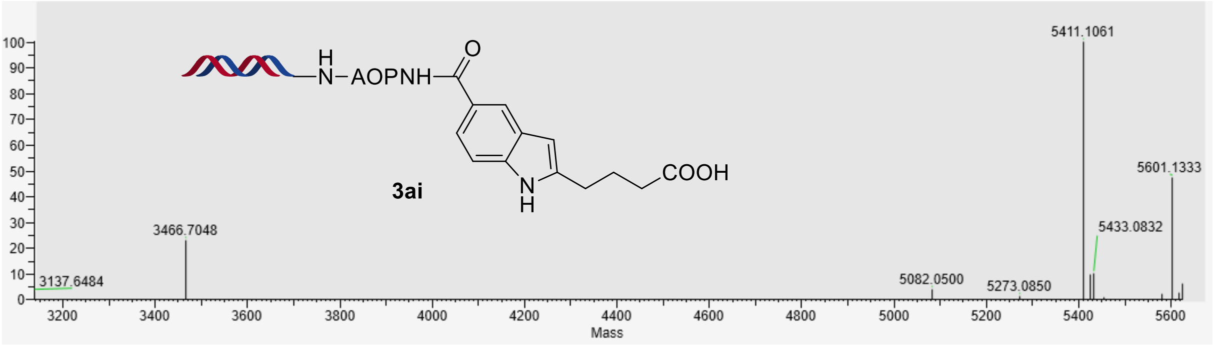

Calcd. for $C_{178}H_{247}N_{54}O_{109}P_{17}$ 5411.0985; found 5411.1072

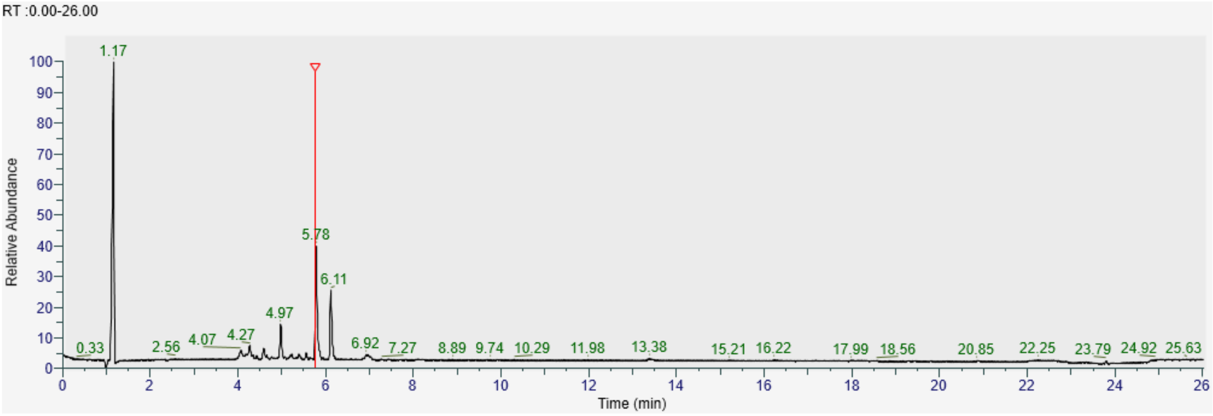

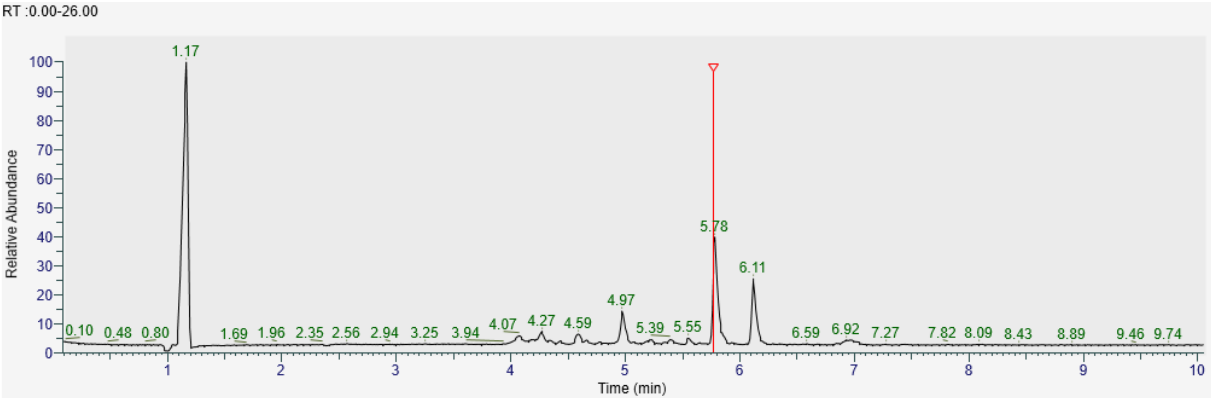

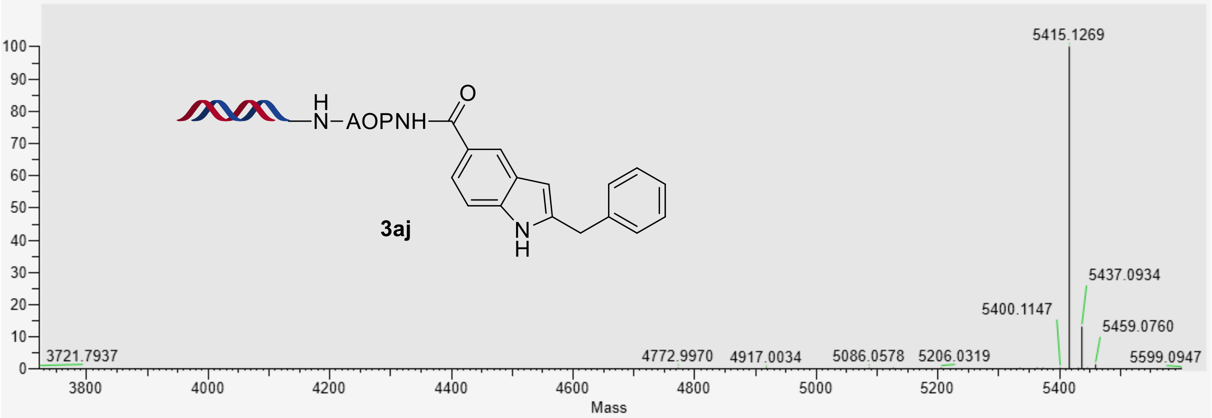

Calcd. for $C_{181}H_{247}N_{54}O_{107}P_{17}$ 5415.1086; found 5415.1269

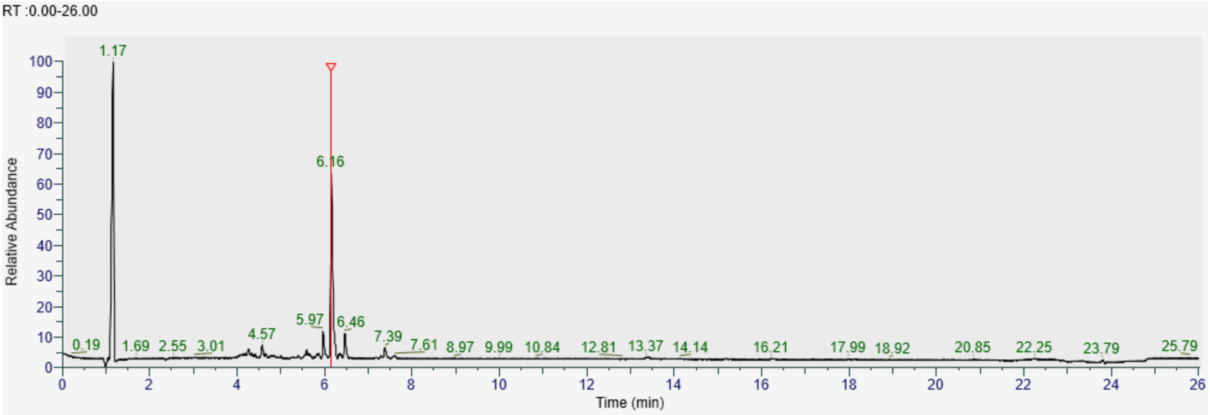

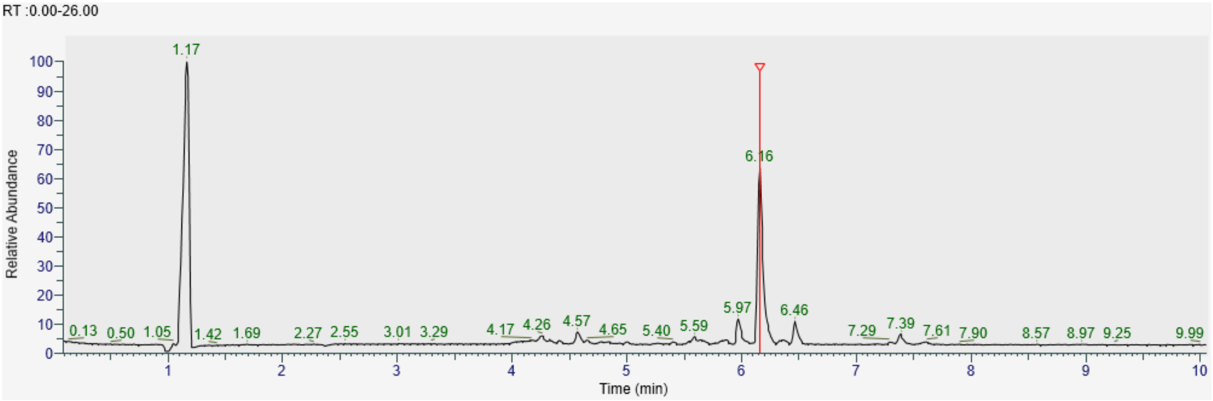

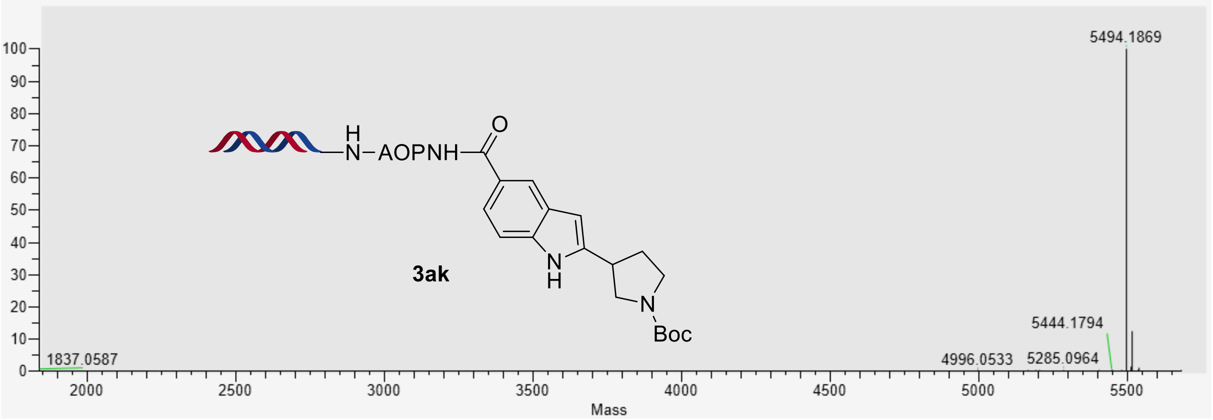

Calcd. for $C_{183}H_{256}N_{55}O_{109}P_{17}$ 5494.1720; found 5494.1869

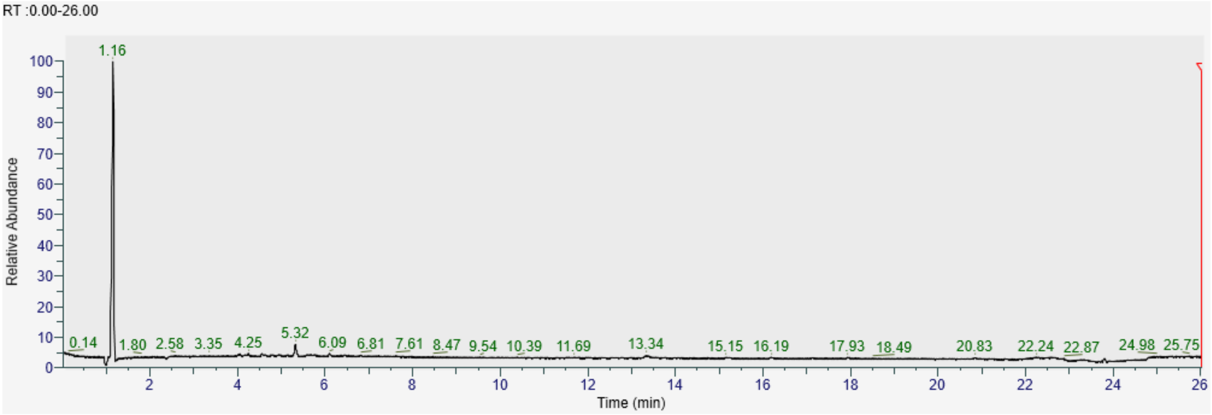

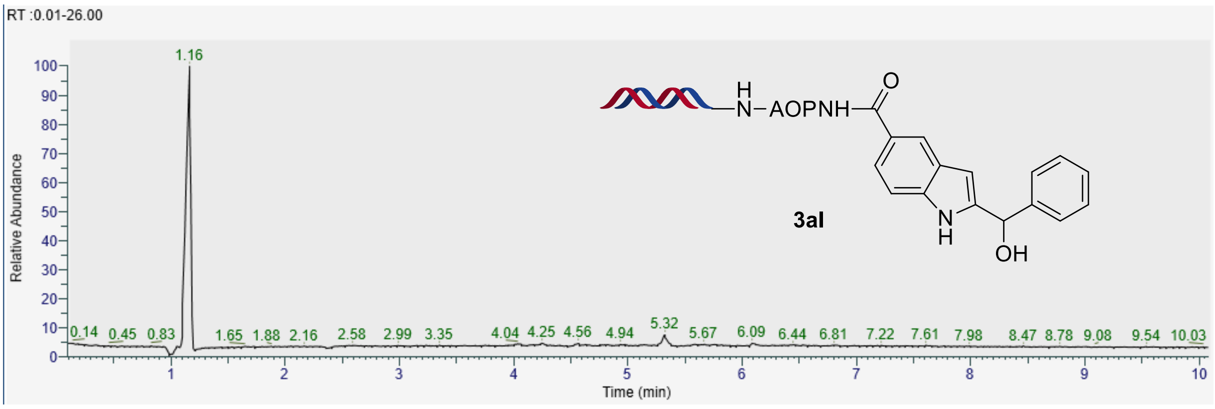

Calcd. for $C_{181}H_{247}N_{54}O_{108}P_{17}$ 5431.1036; No product detected.

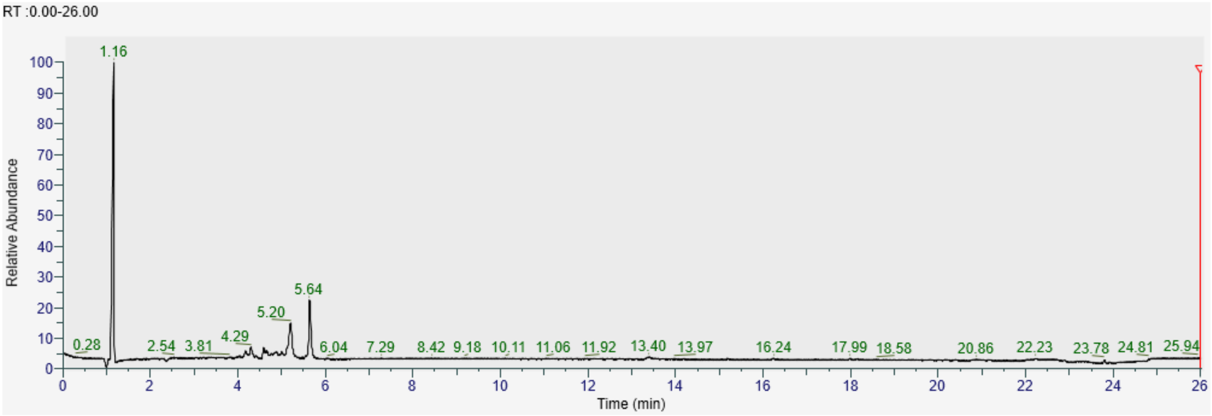

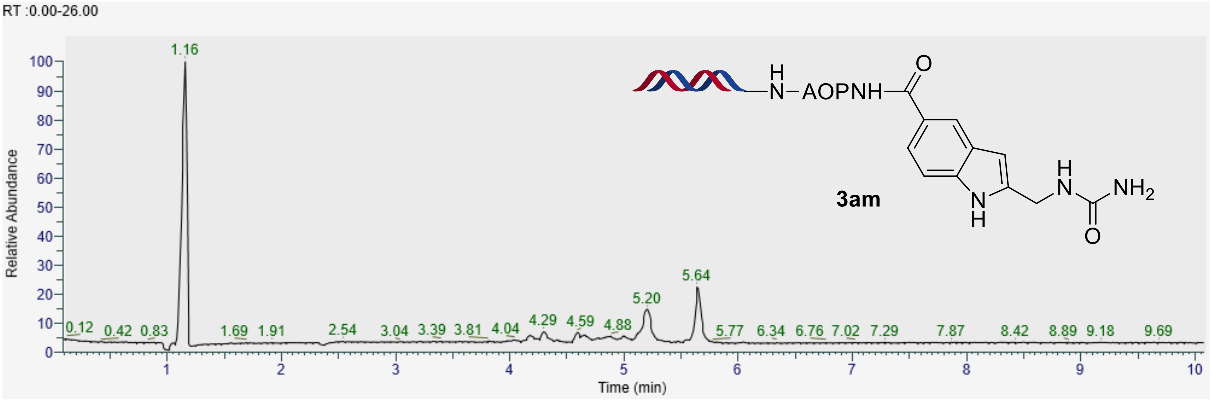

Calcd. for $C_{176}H_{245}N_{56}O_{108}P_{17}$ 5397.0941; No product detected.

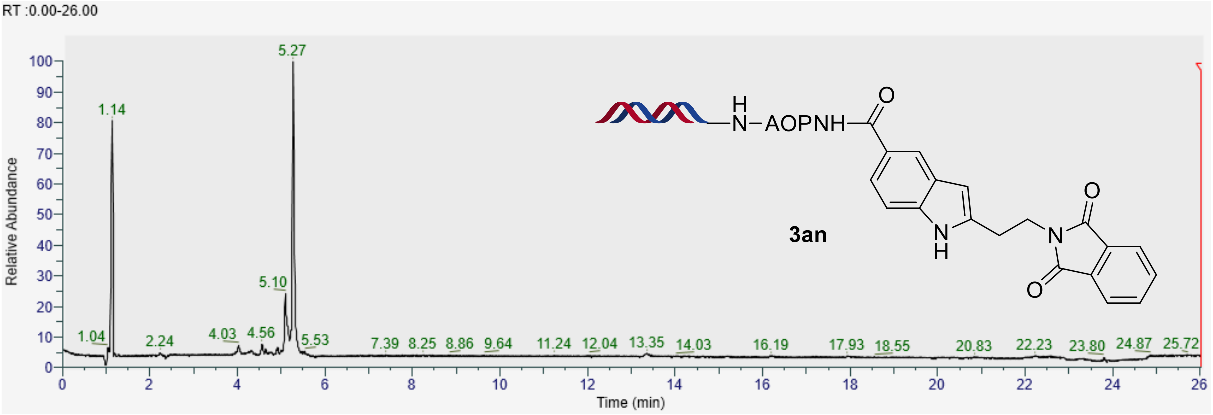

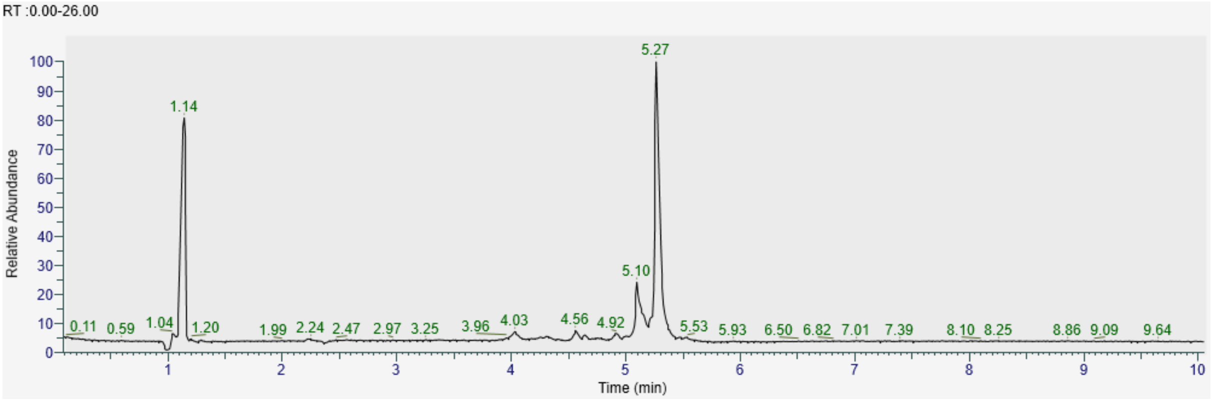

Calcd. for $C_{184}H_{248}N_{55}O_{109}P_{17}$ 5498.1094; No product detected

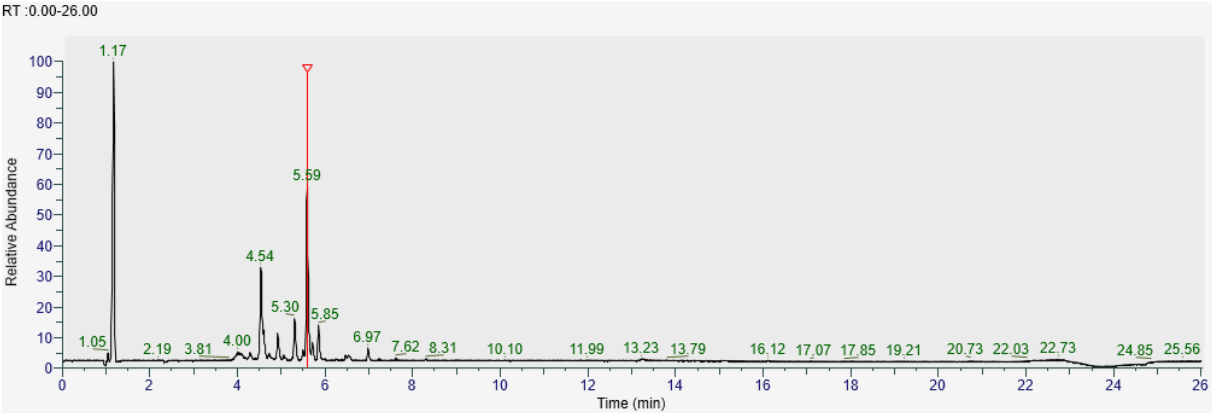

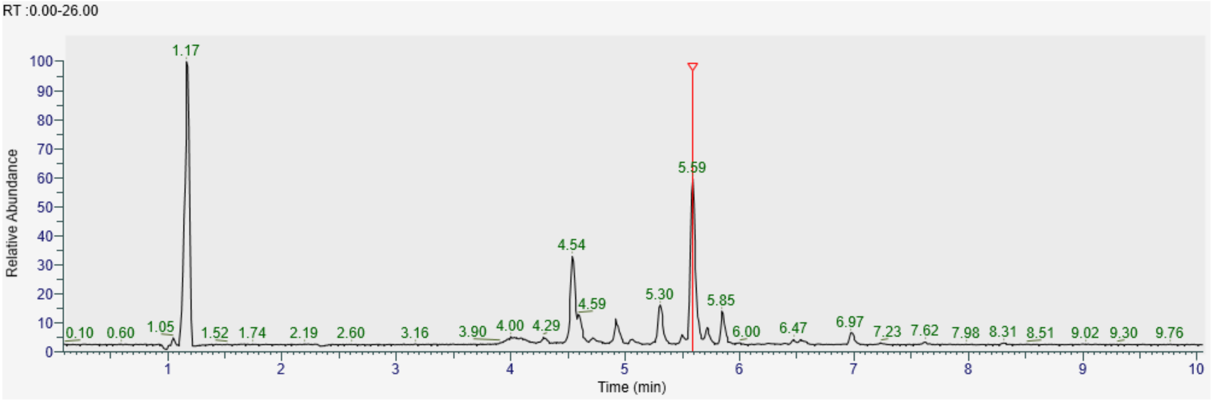

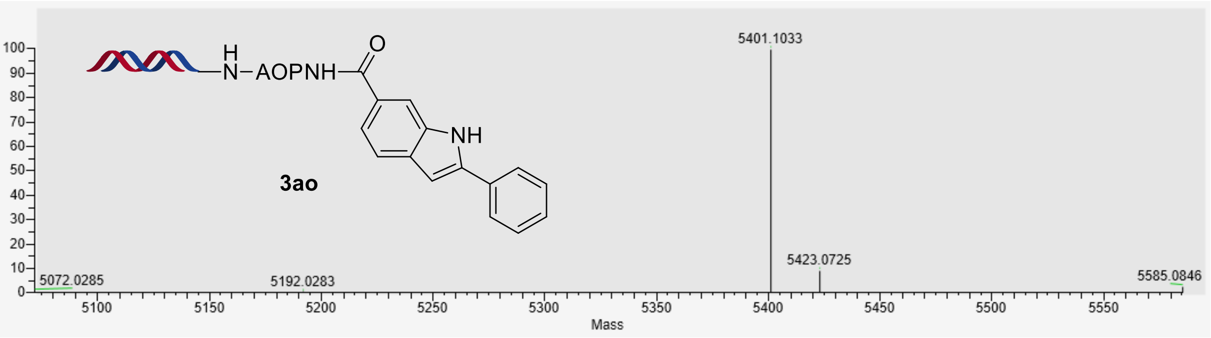

Calcd. for $C_{180}H_{245}N_{54}O_{107}P_{17}$ 5401.0930; found 5401.1033

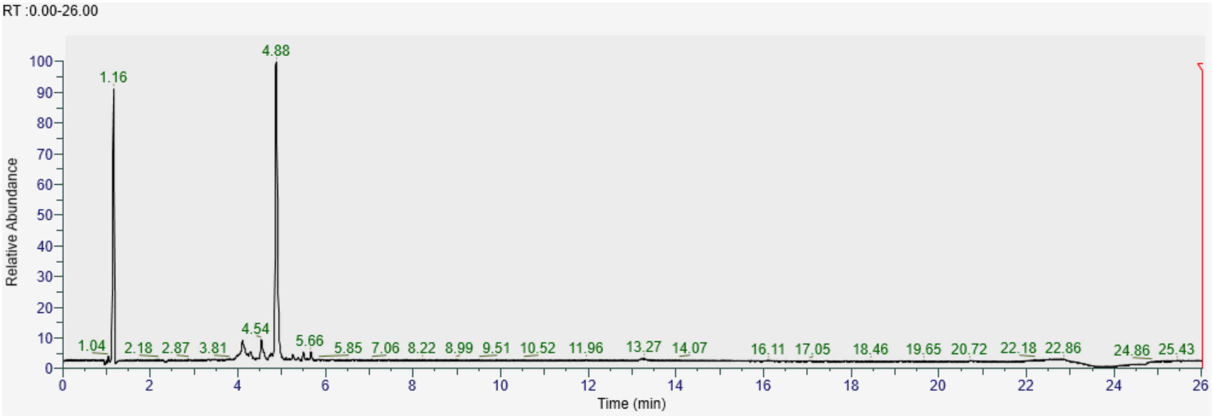

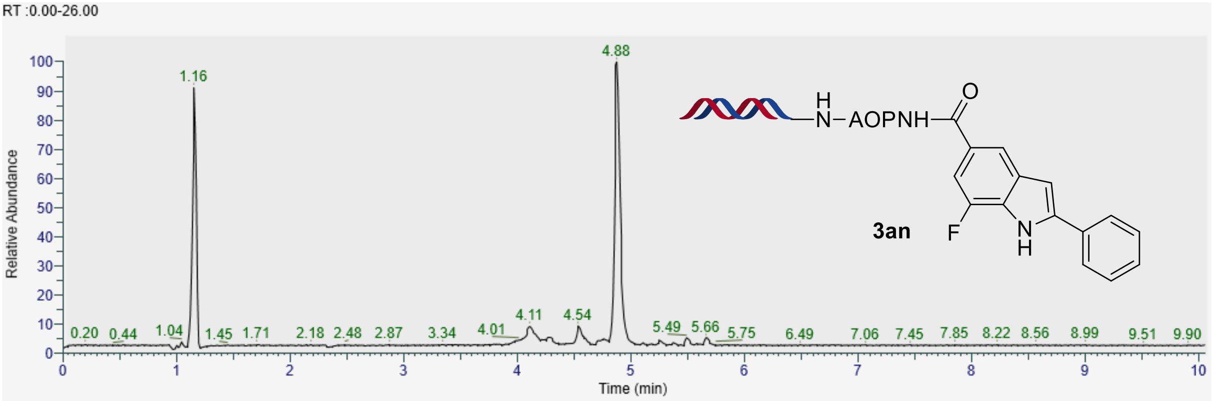

Calcd. for $C_{180}H_{246}N_{54}O_{107}P_{17}F$ 5421.0992; No product detected.

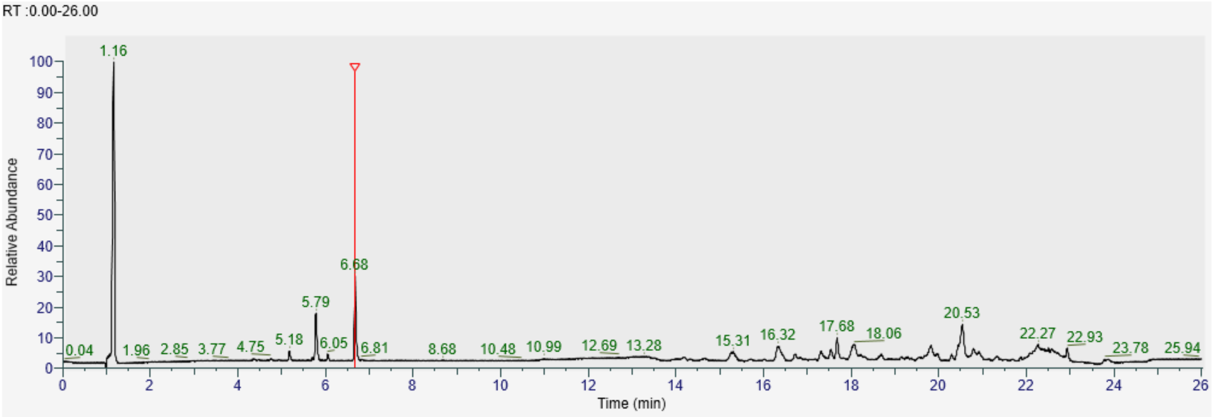

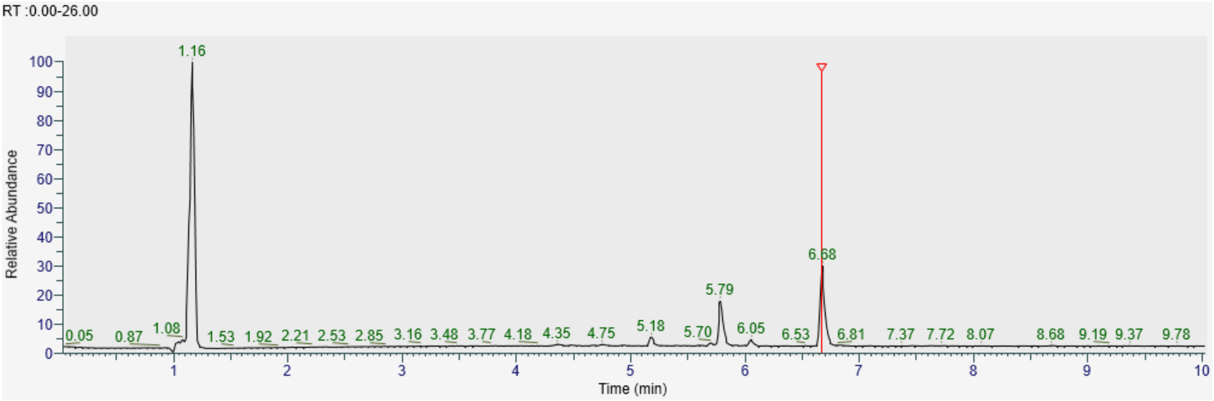

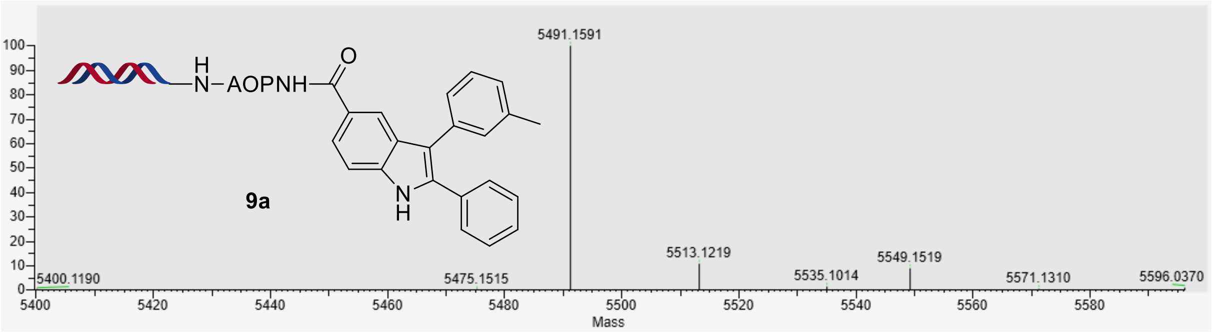

Calcd. for $C_{187}H_{251}N_{54}O_{107}P_{17}$ 5491.1399; found 5491.1591

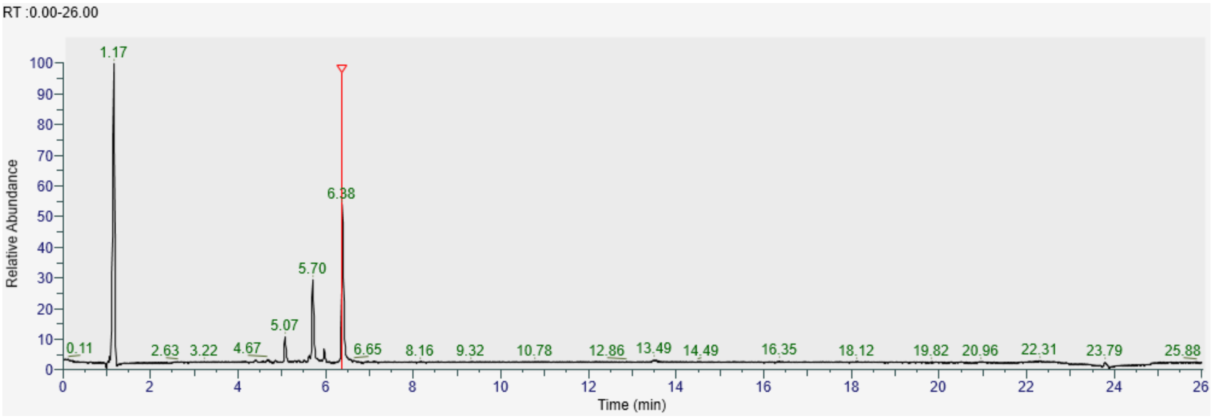

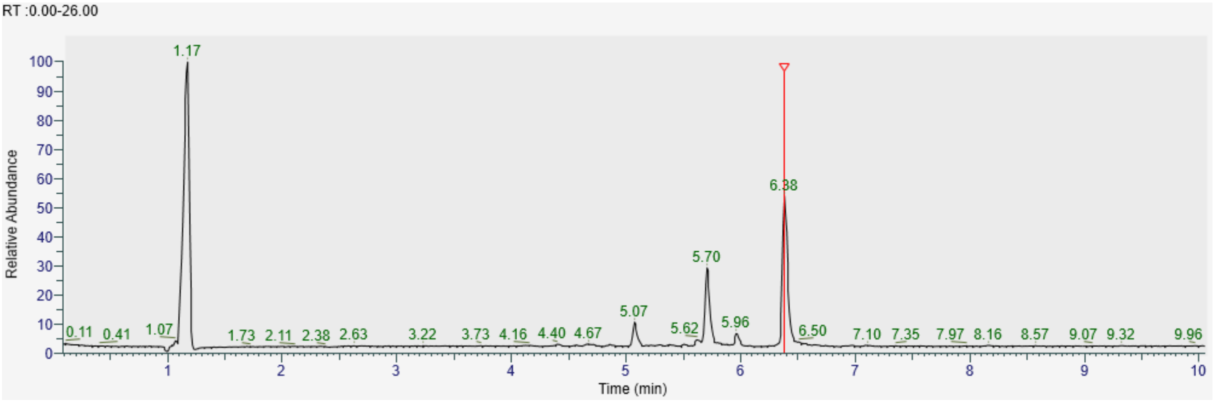

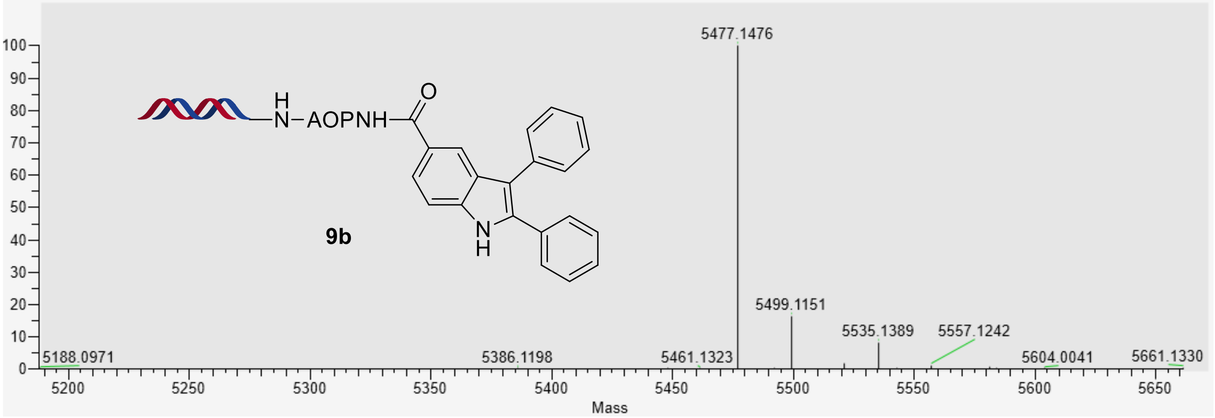

Calcd. for $C_{186}H_{249}N_{54}O_{107}P_{17}$ 5477.1243; found 5477.1476

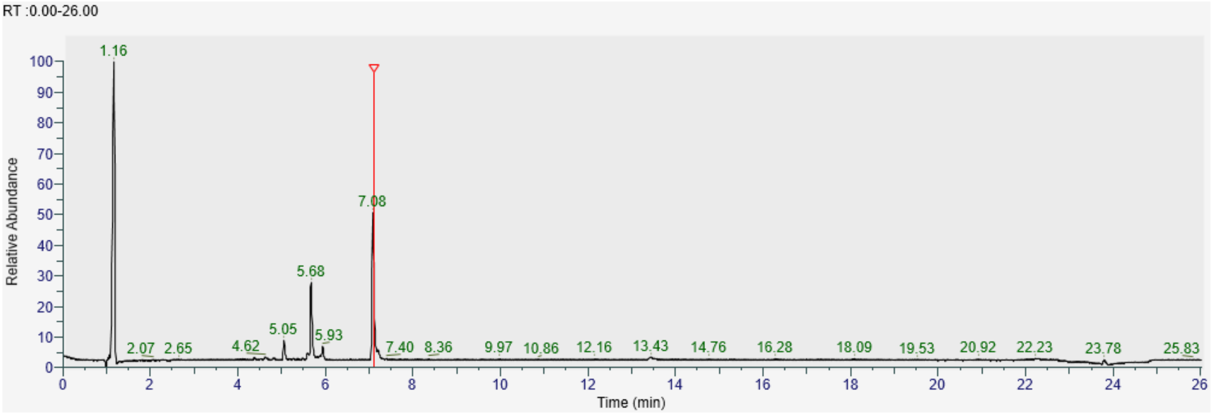

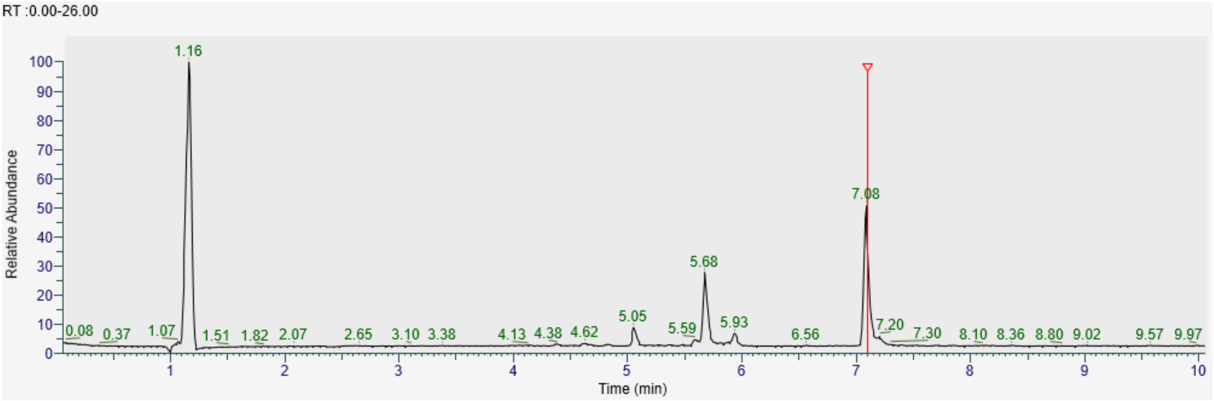

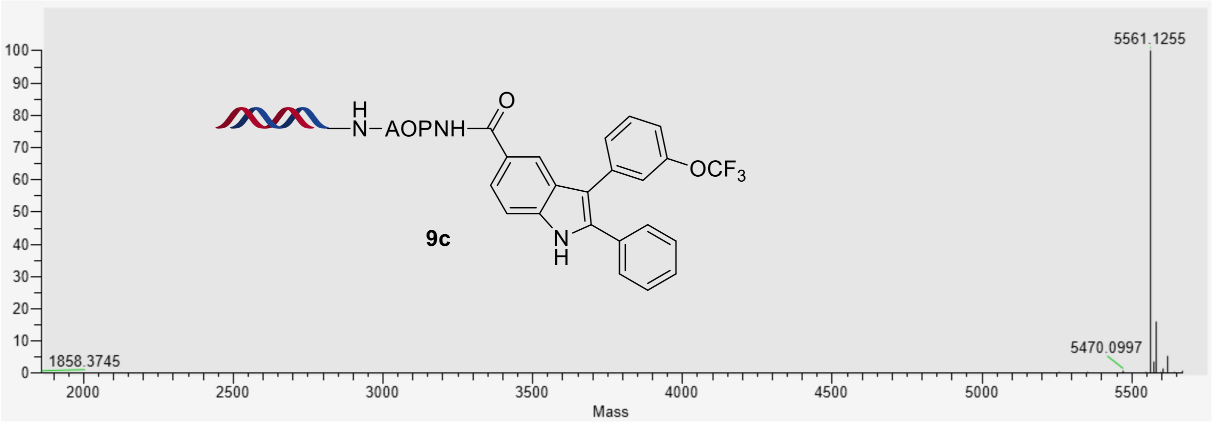

Calcd. for $C_{187}H_{248}F_3N_{54}O_{108}P_{17}$ 5561.1065; found 5561.1255

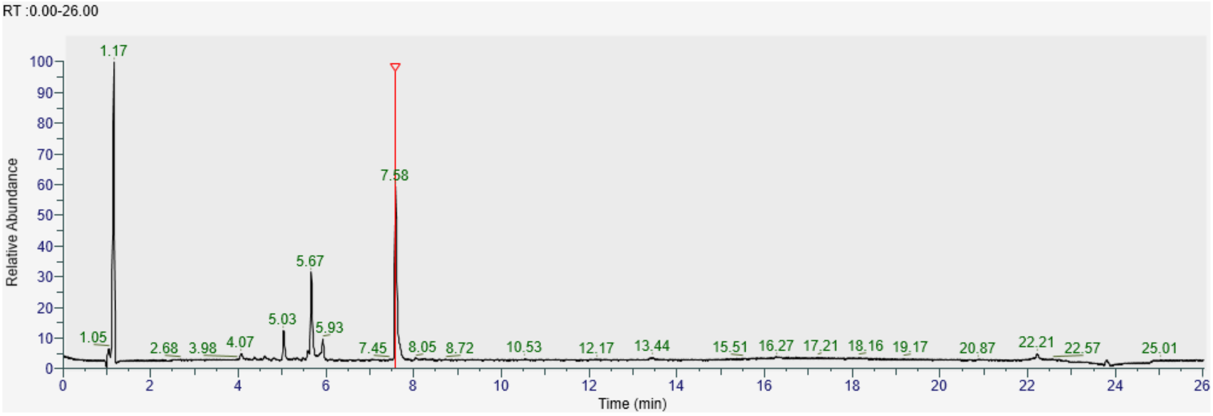

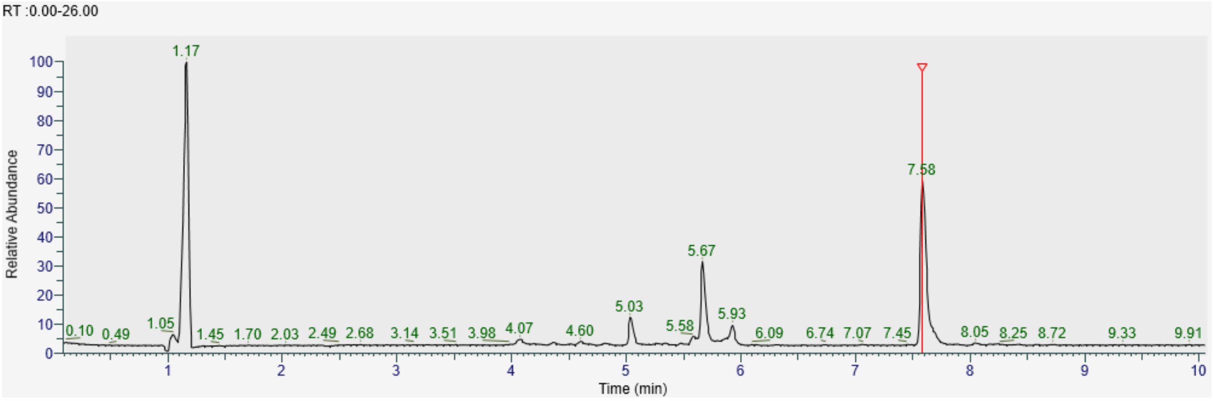

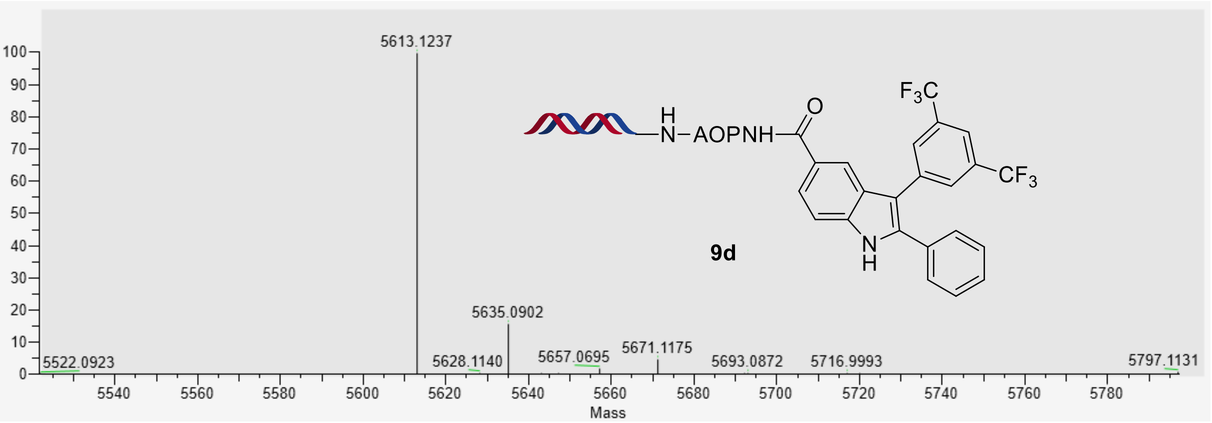

Calcd. for $C_{188}H_{247}F_6N_{54}O_{107}P_{17}$ 5613.0991; found 5613.1237

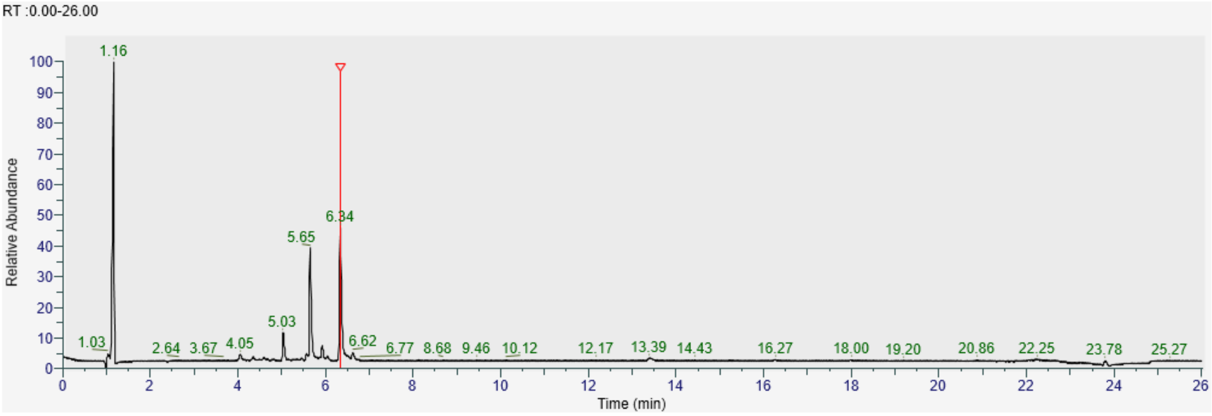

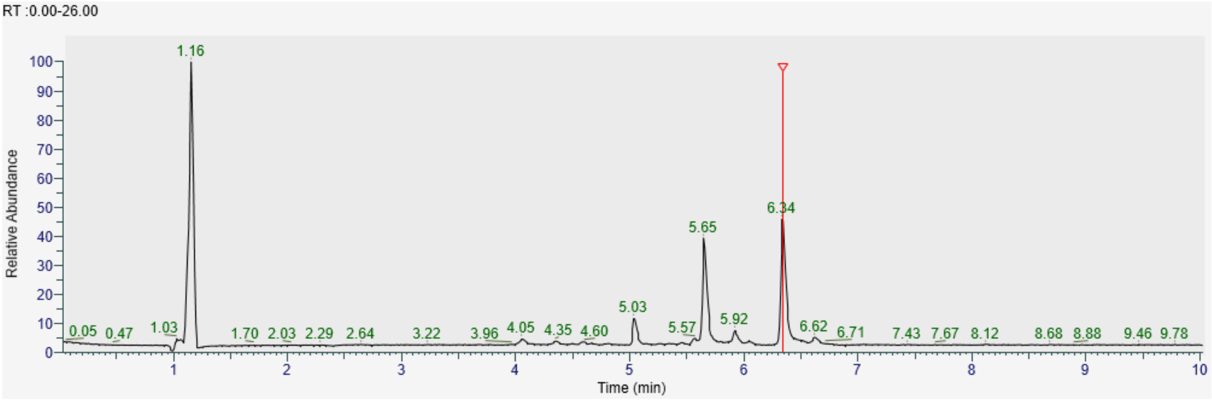

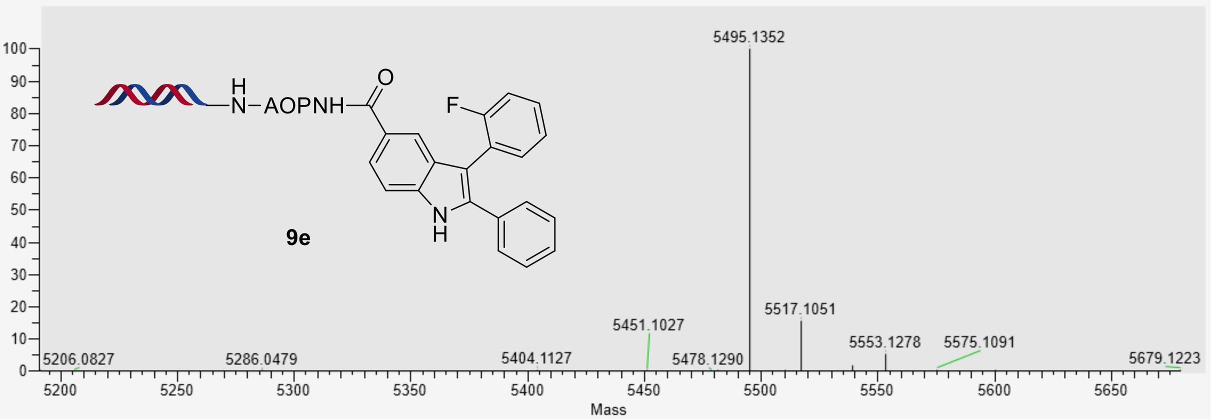

Calcd. for $C_{186}H_{248}FN_{54}O_{107}P_{17}$ 5495.1149; found 5495.1352

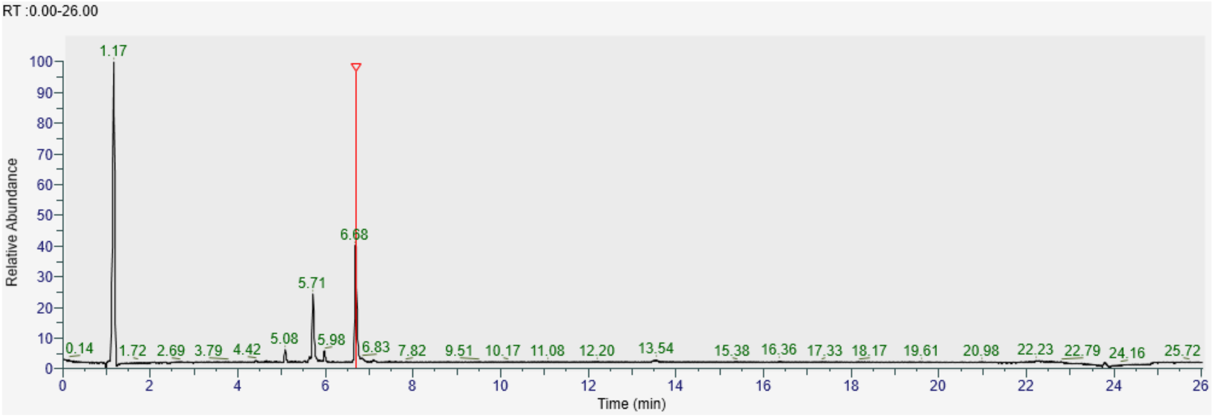

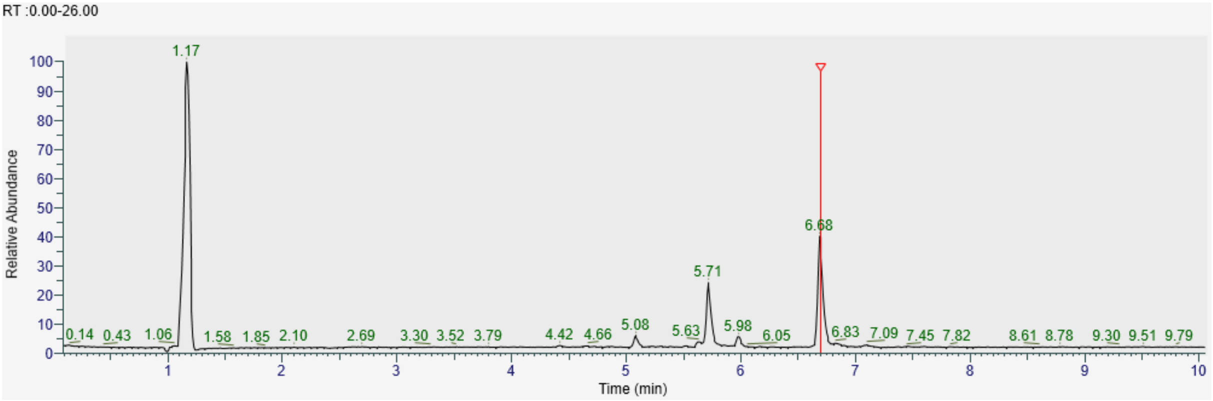

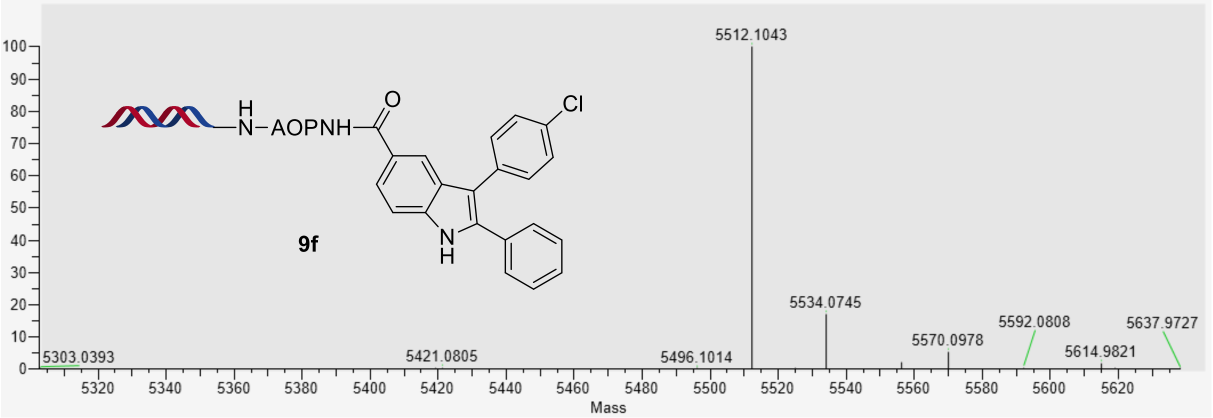

Calcd. for $C_{186}H_{248}ClN_{54}O_{107}P_{17}$ 5511.0853; found 5512.1043

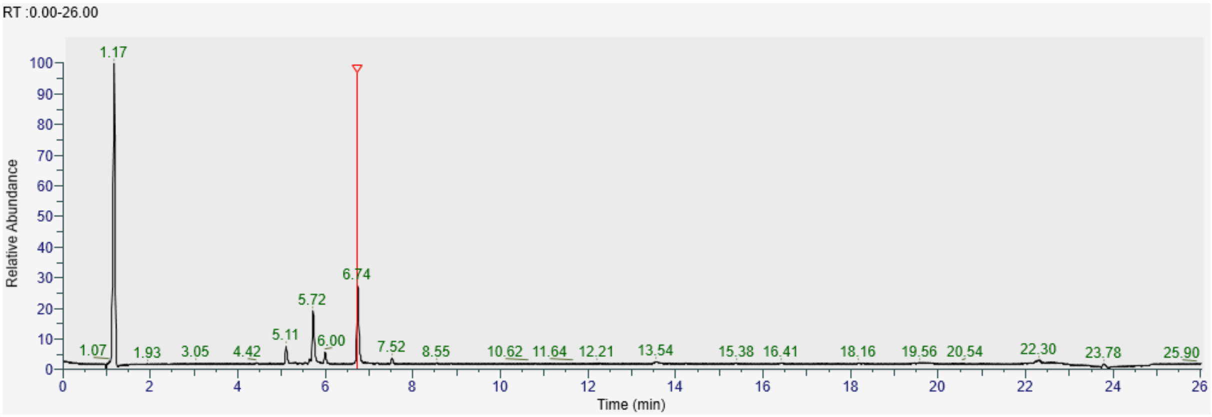

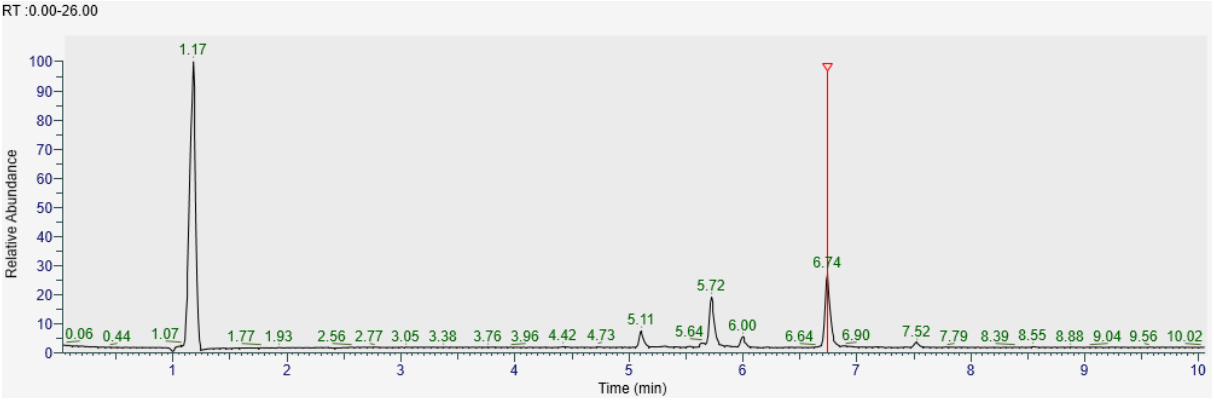

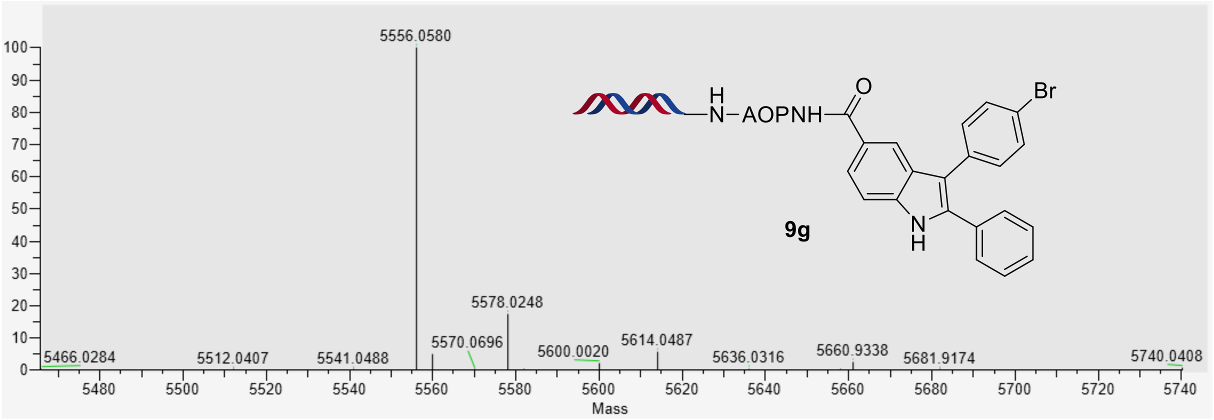

Calcd. for $C_{186}H_{248}BrN_{54}O_{107}P_{17}$ 5555.0348; found 5555.0580

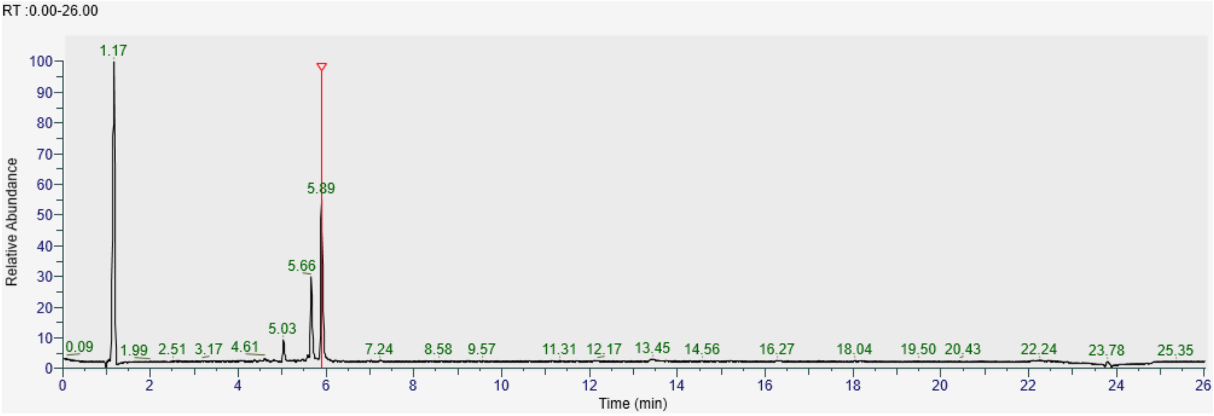

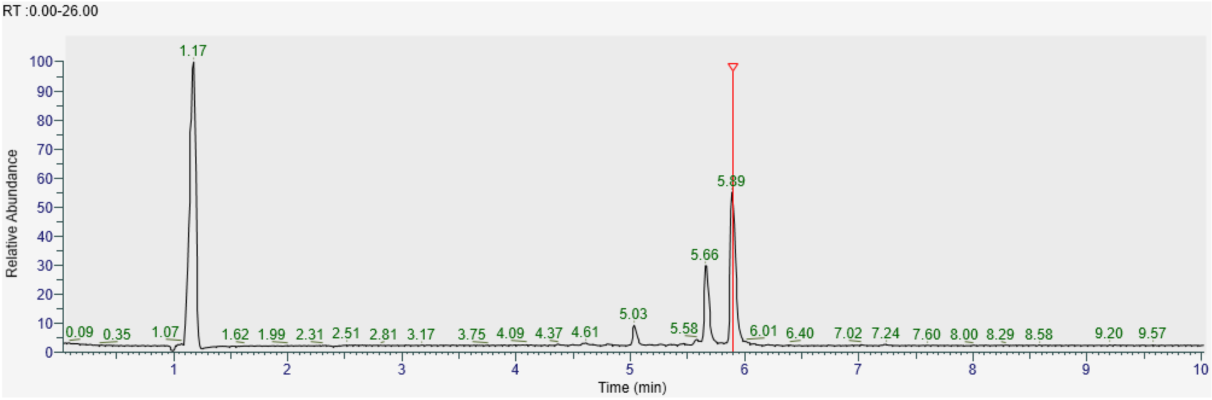

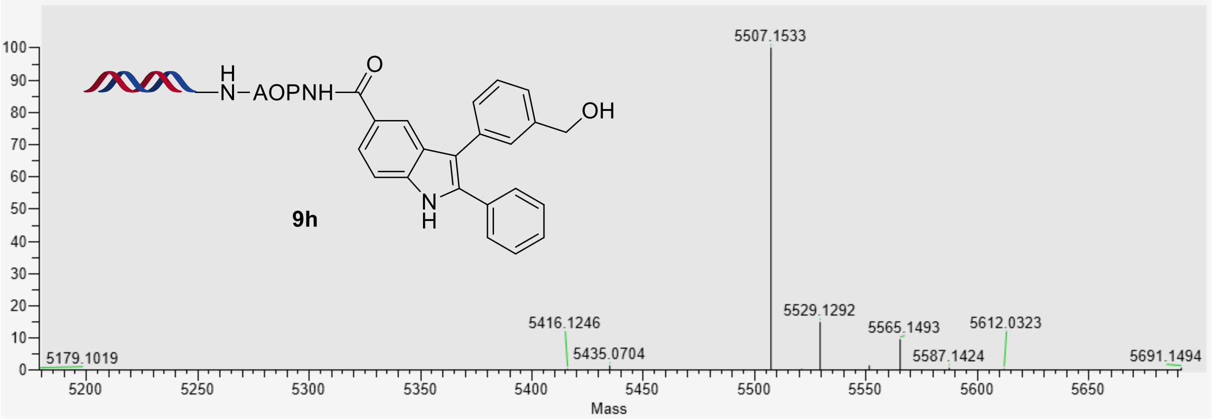

Calcd. for $C_{187}H_{251}N_{54}O_{108}P_{17}$ 5507.1349; found 5507.1533

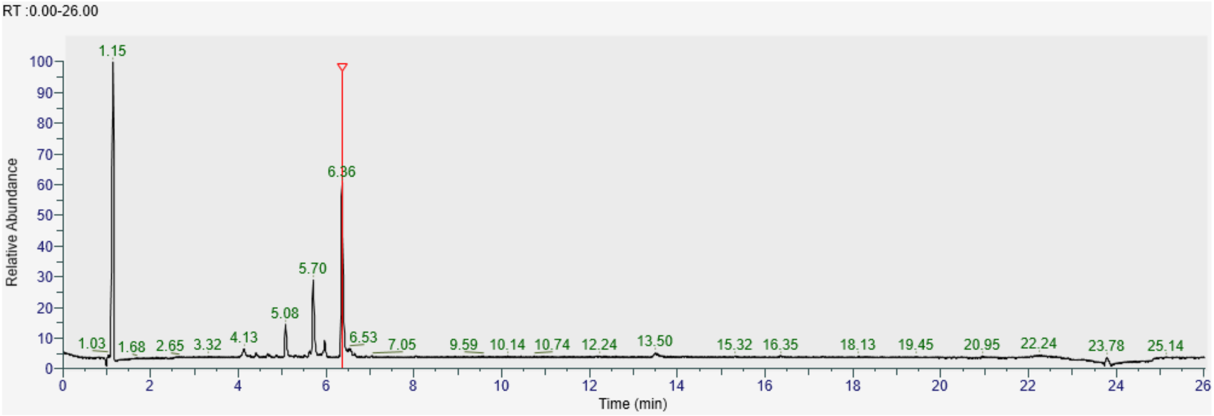

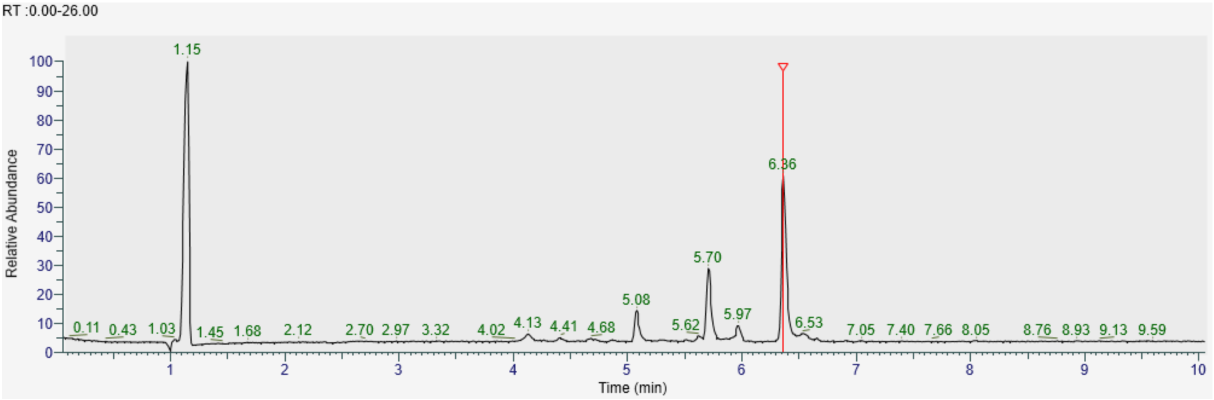

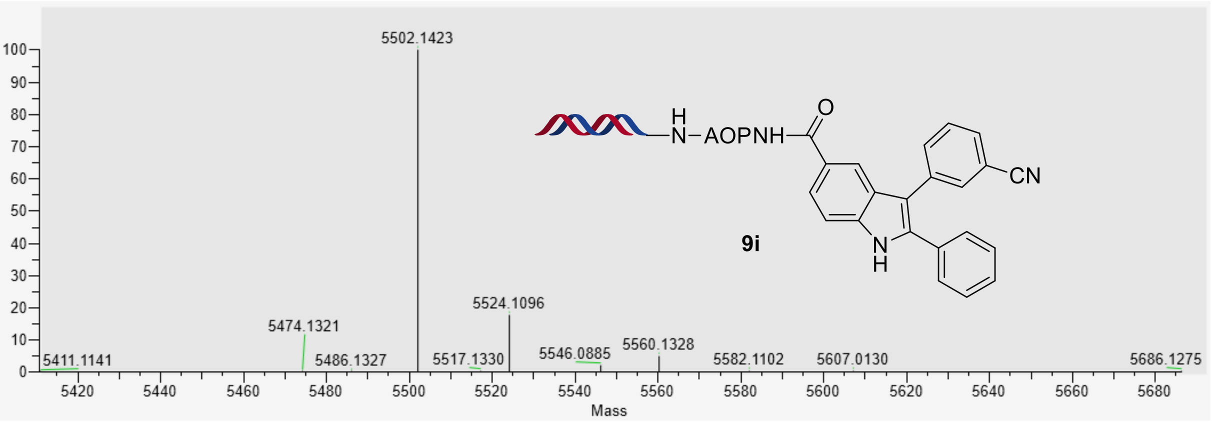

Calcd. for $C_{187}H_{248}N_{55}O_{107}P_{17}$ 5502.1195; found 5502.1423

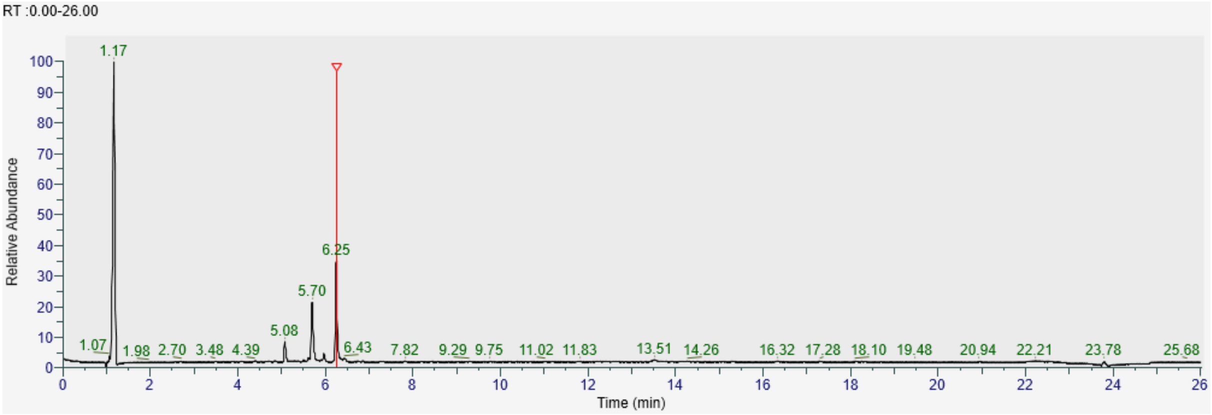

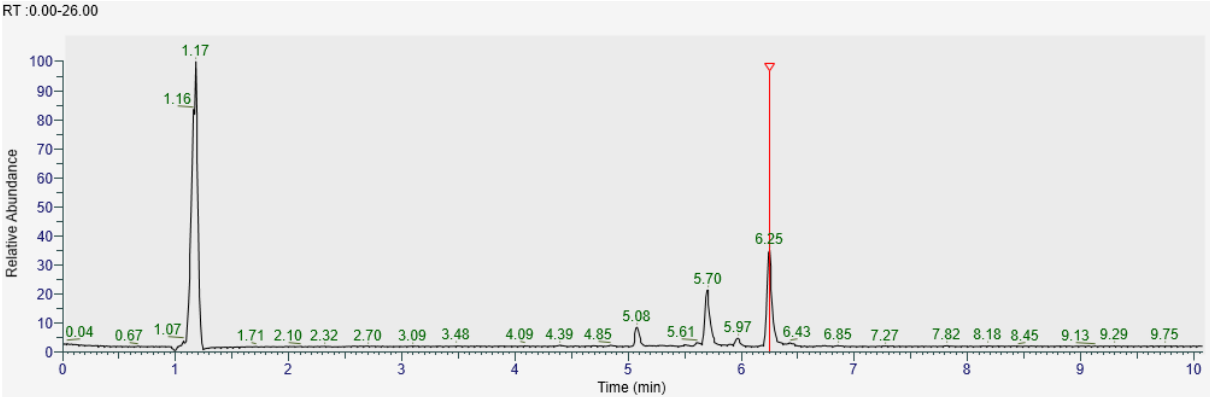

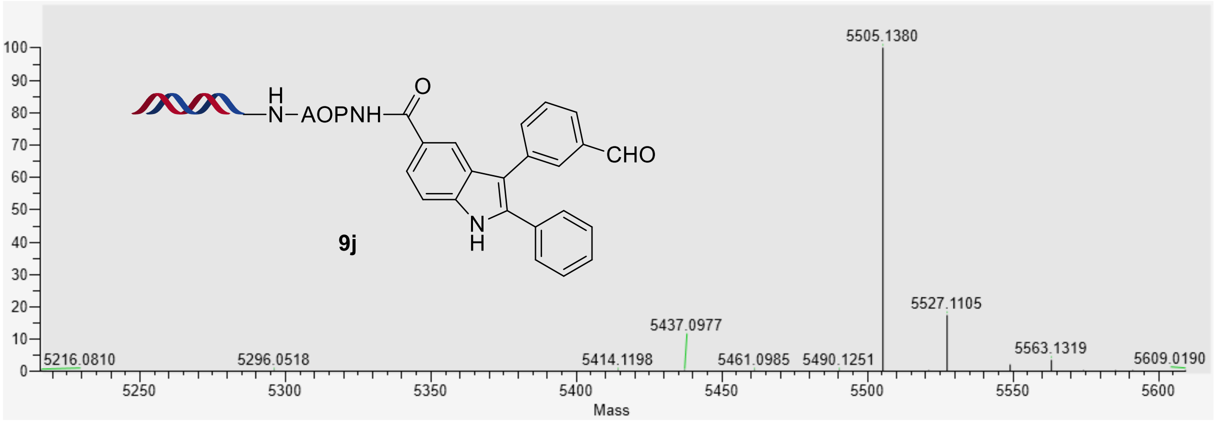

Calcd. for $C_{187}H_{249}N_{54}O_{108}P_{17}$ 5505.1192; found 5505.1380

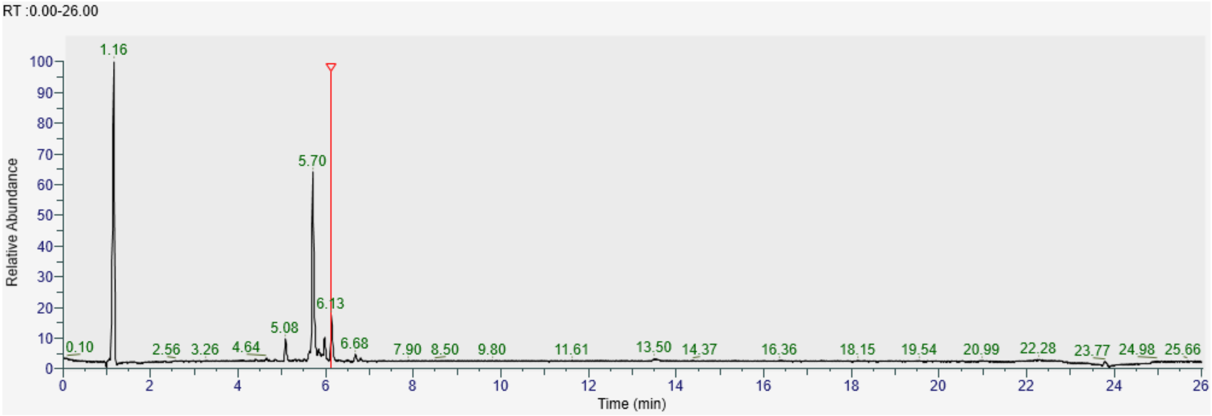

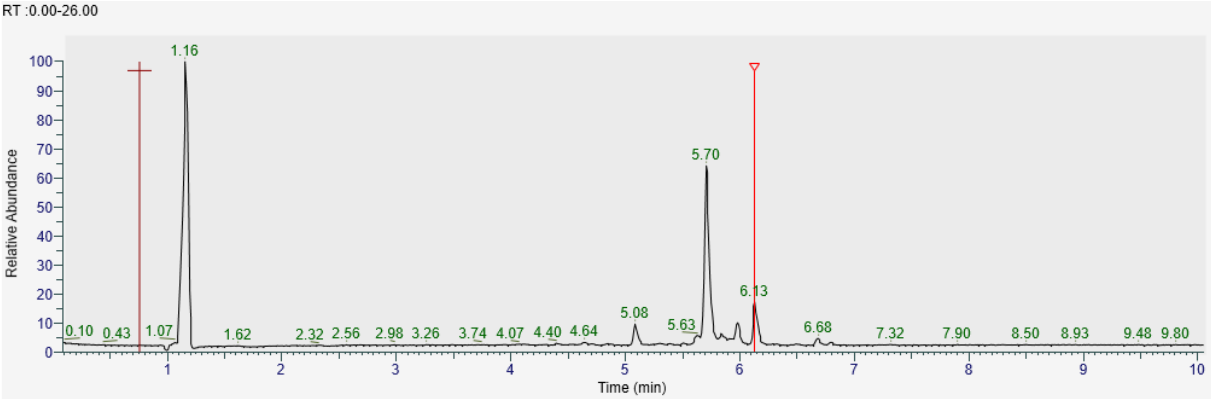

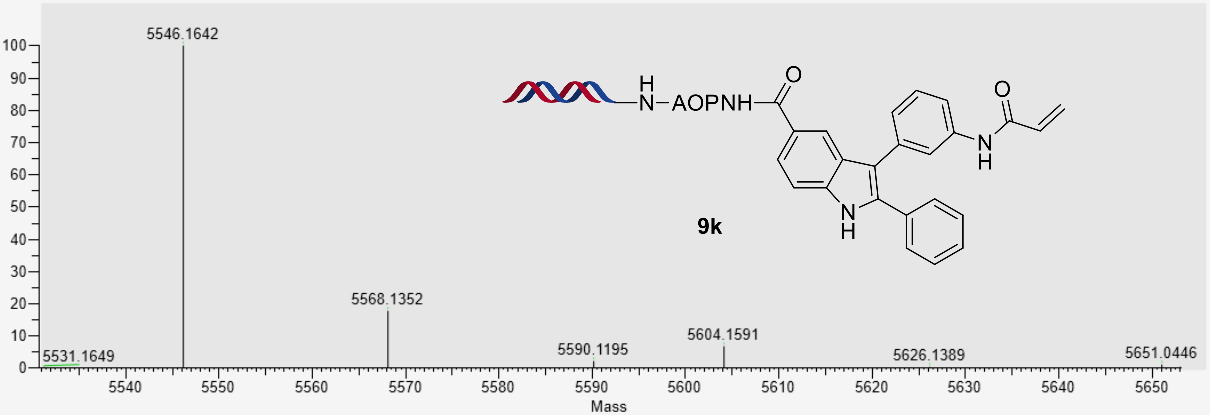

Calcd. for $C_{189}H_{252}N_{55}O_{108}P_{17}$ 5546.1458; found 5546.1642

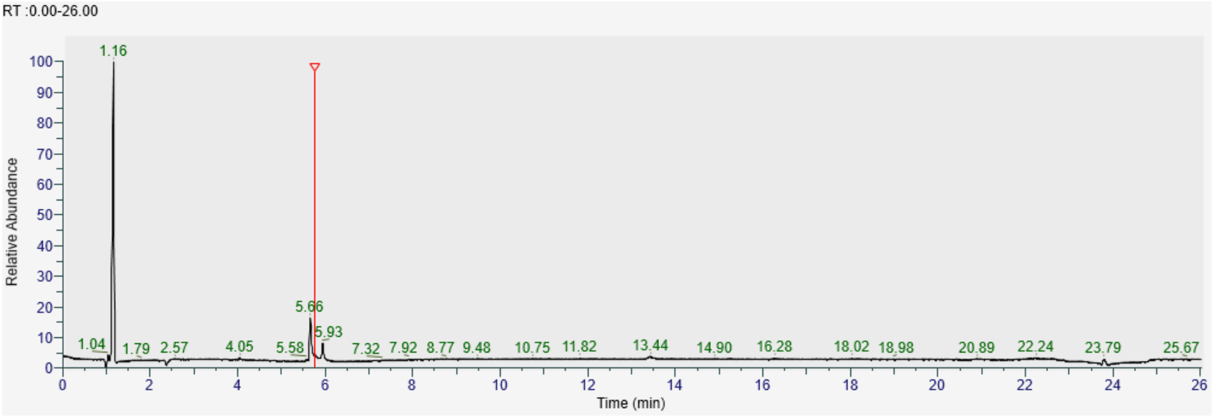

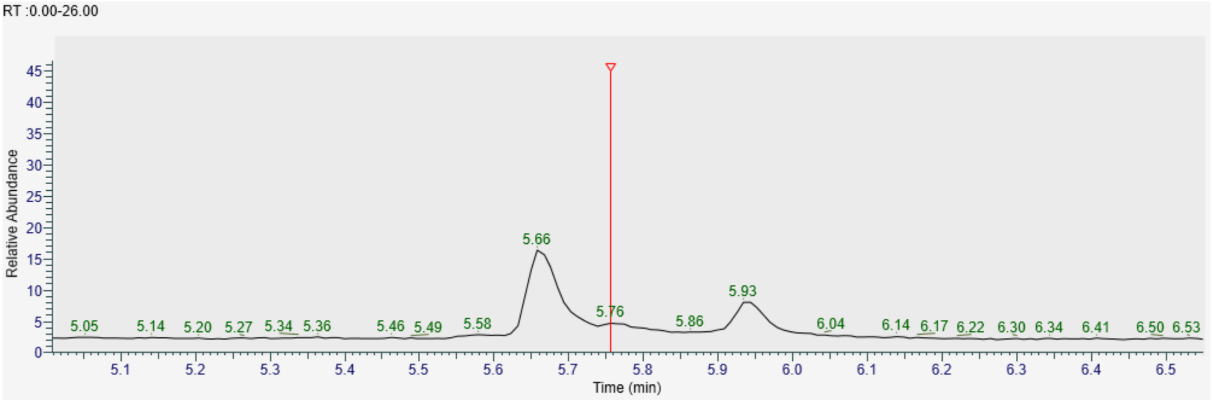

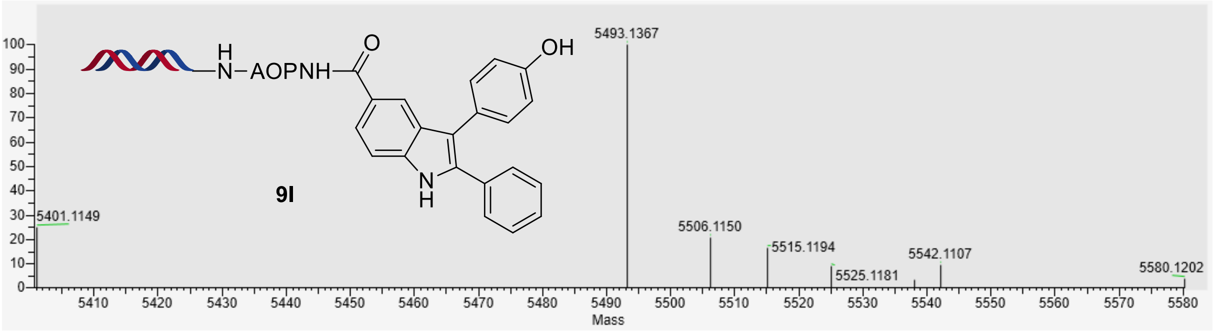

Calcd. for $C_{186}H_{249}N_{54}O_{108}P_{17}$ 5493.1192; found 5493.1367

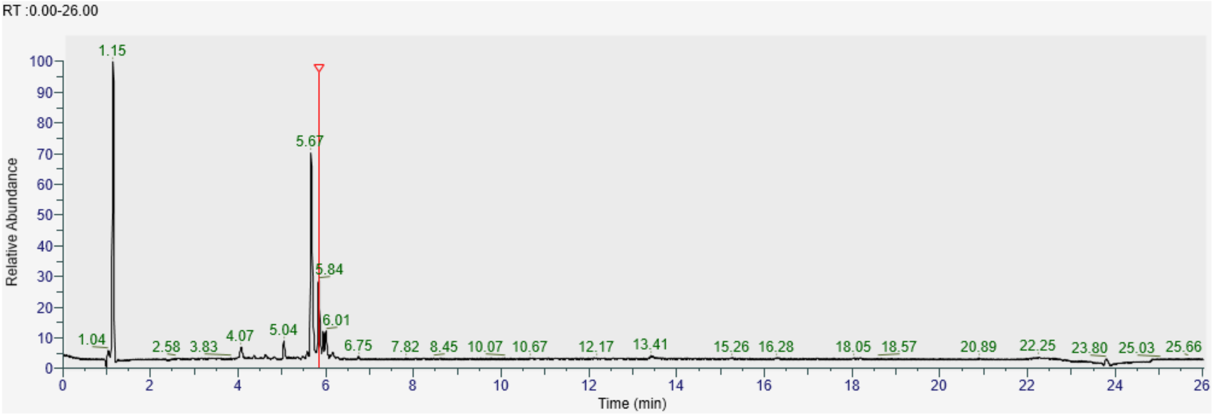

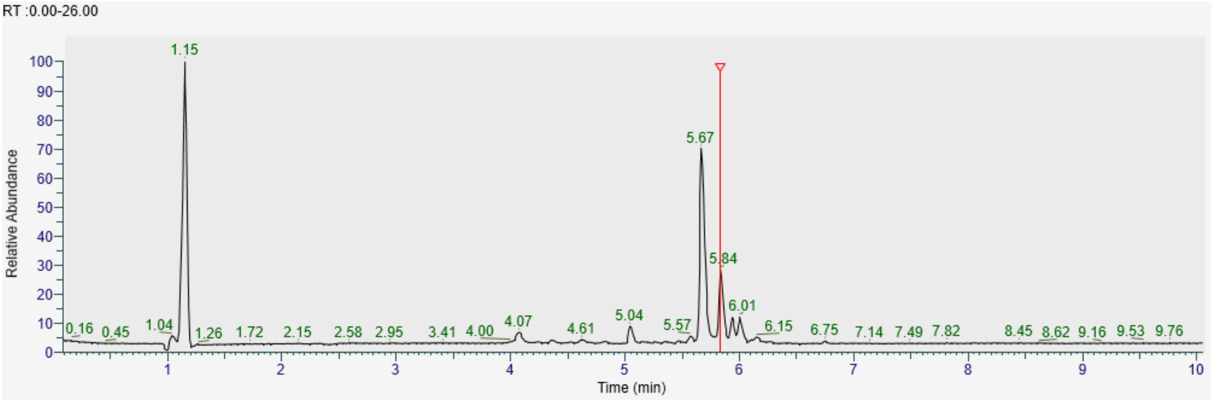

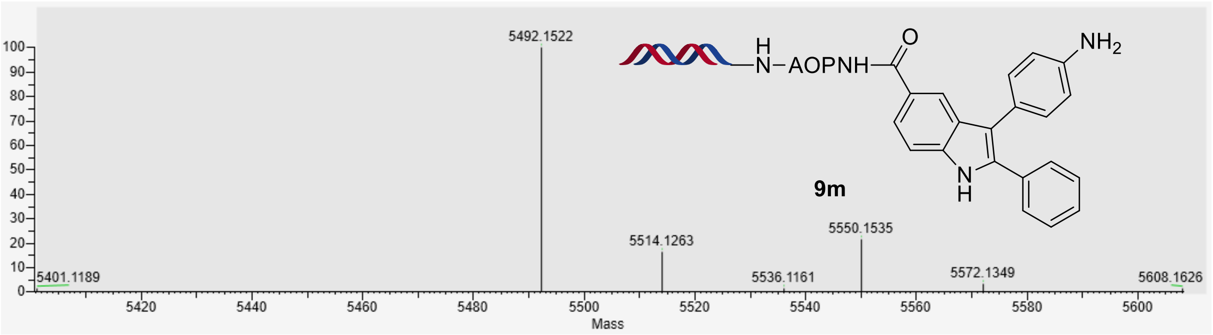

Calcd. for $C_{186}H_{250}N_{55}O_{107}P_{17}$ 5492.1351; found 5492.1522

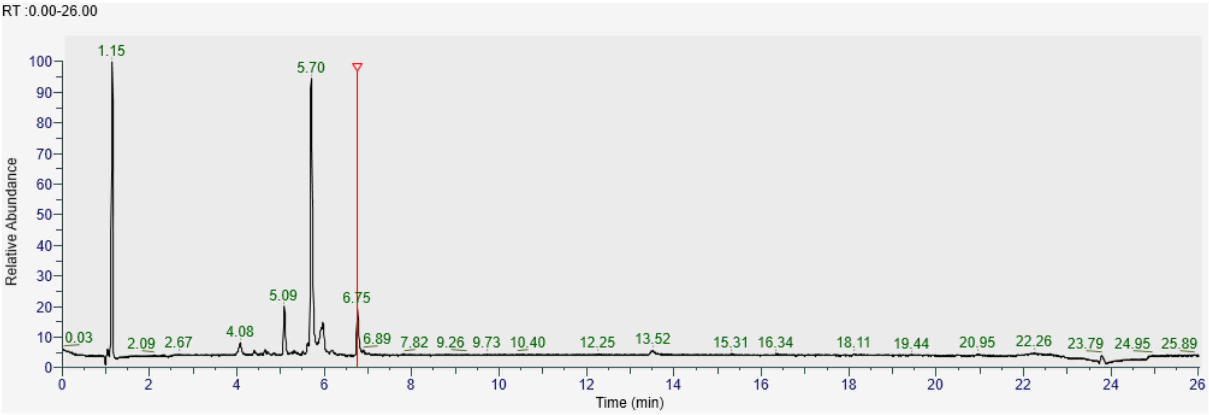

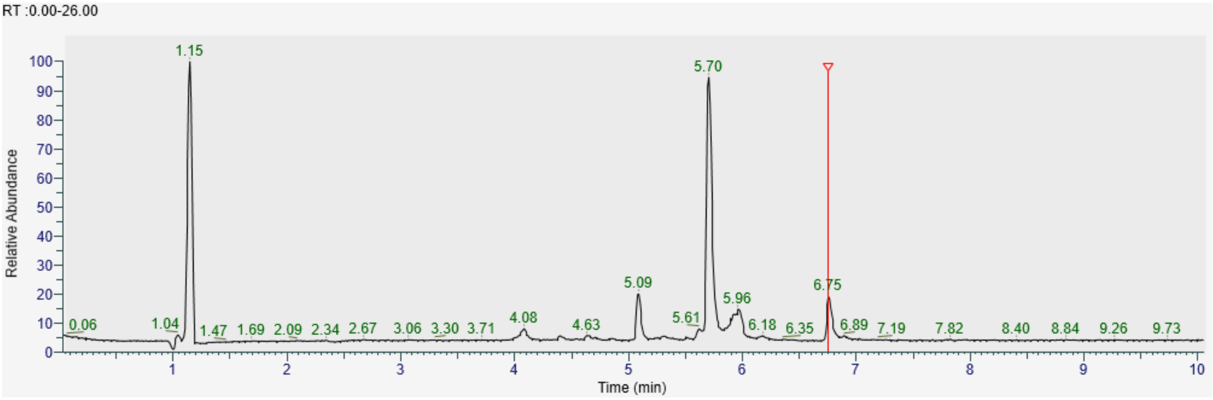

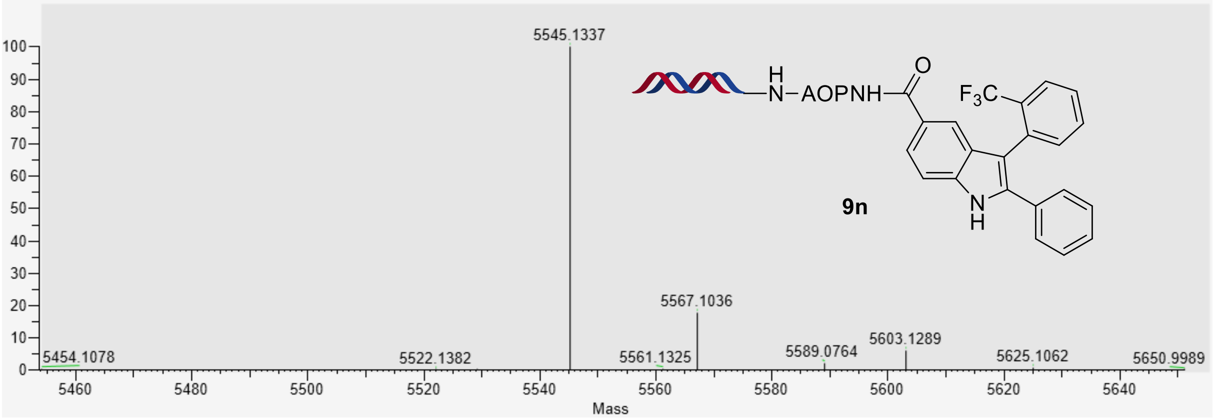

Calcd. for $C_{187}H_{248}F_3N_{54}O_{107}P_{17}$ 5545.1117; found 5545.1337

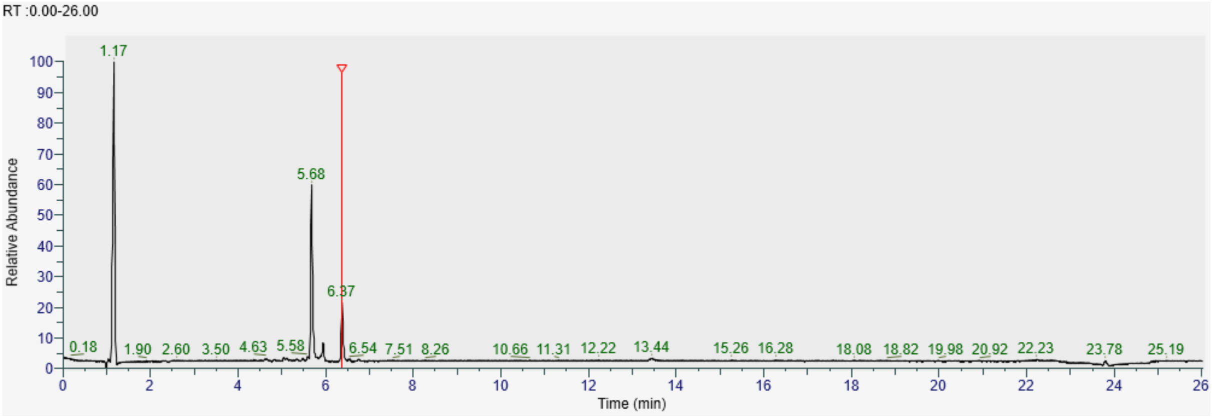

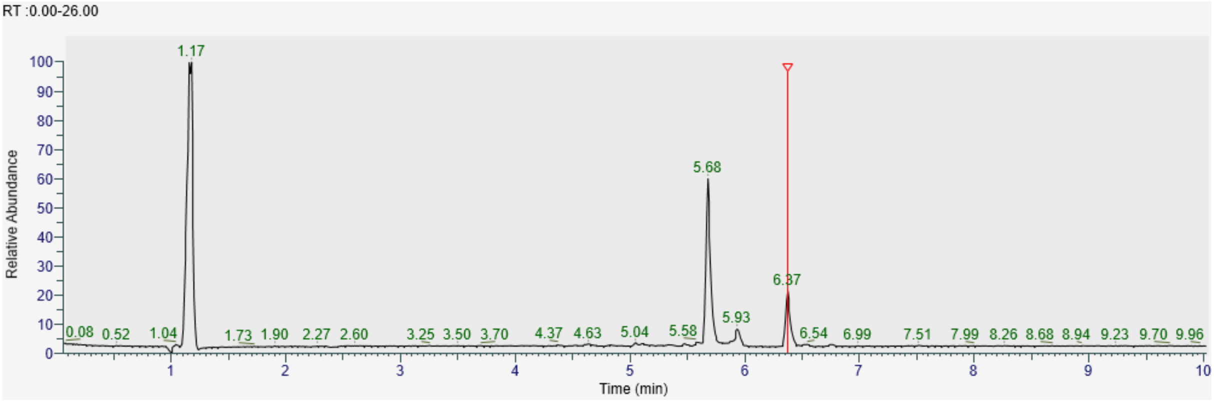

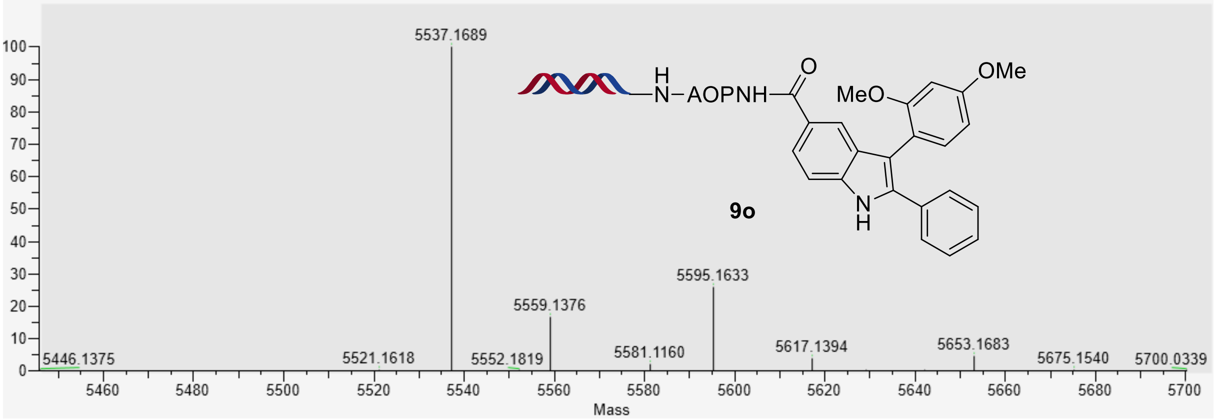

Calcd. for $C_{188}H_{253}N_{54}O_{109}P_{17}$ 5537.1454; found 5537.1689

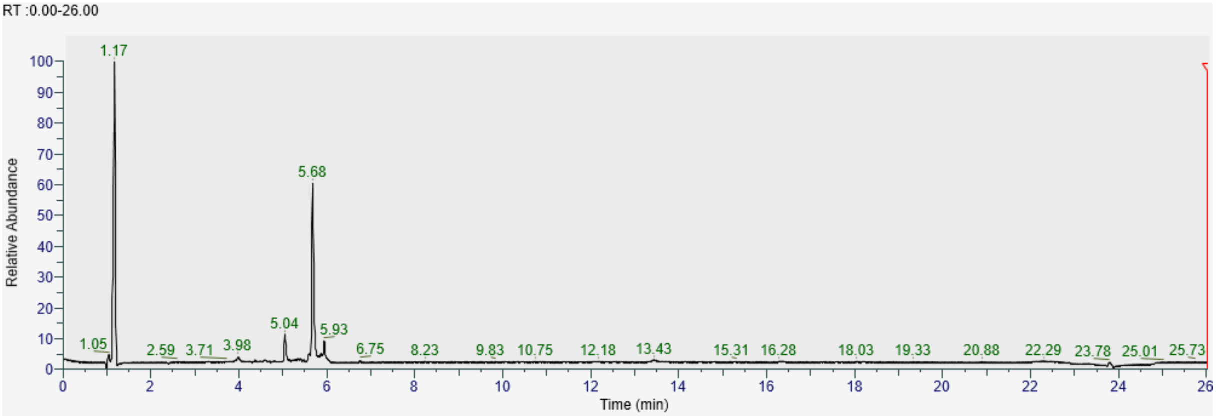

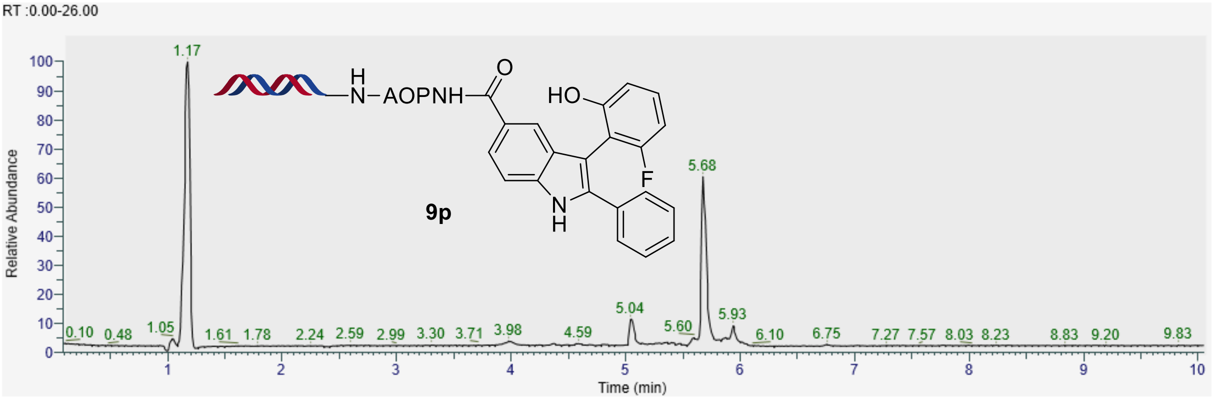

Calcd. for $C_{186}H_{248}N_{54}O_{108}P_{17}F$ 5511.1097; No product detected.

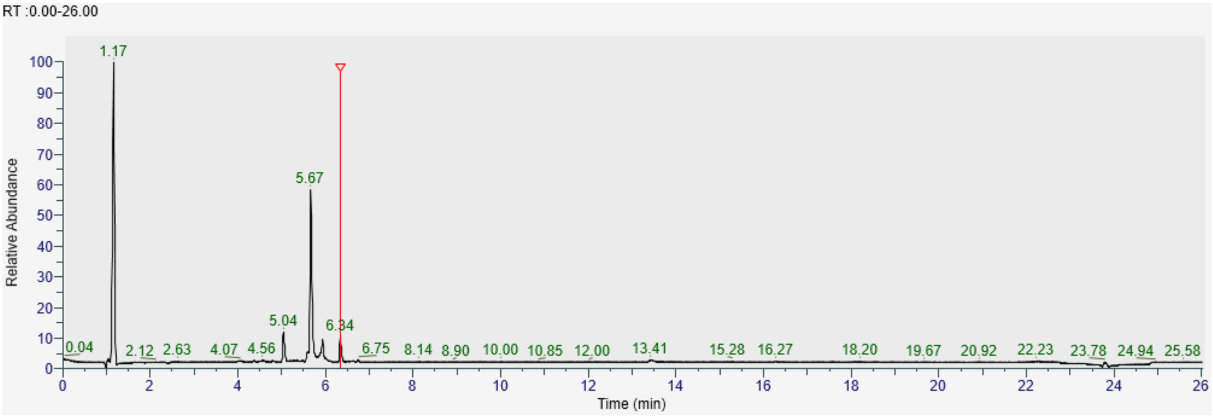

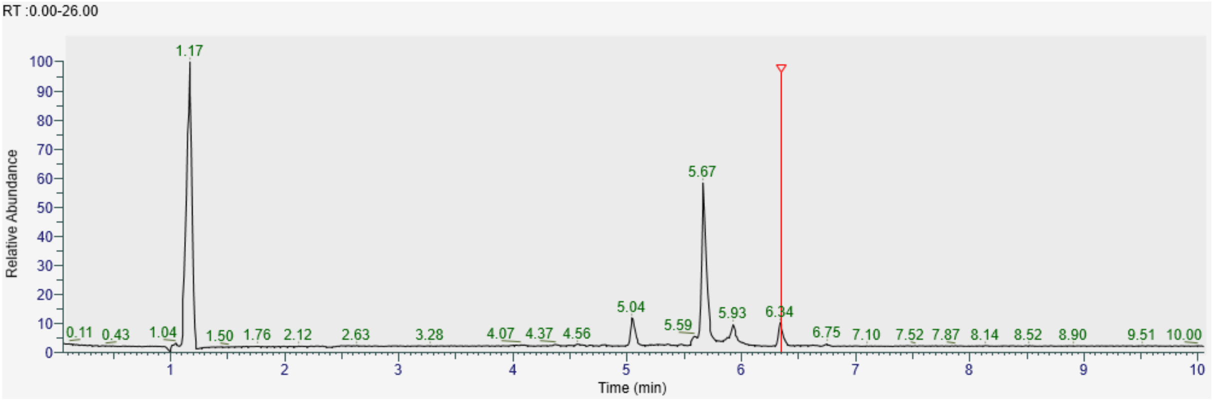

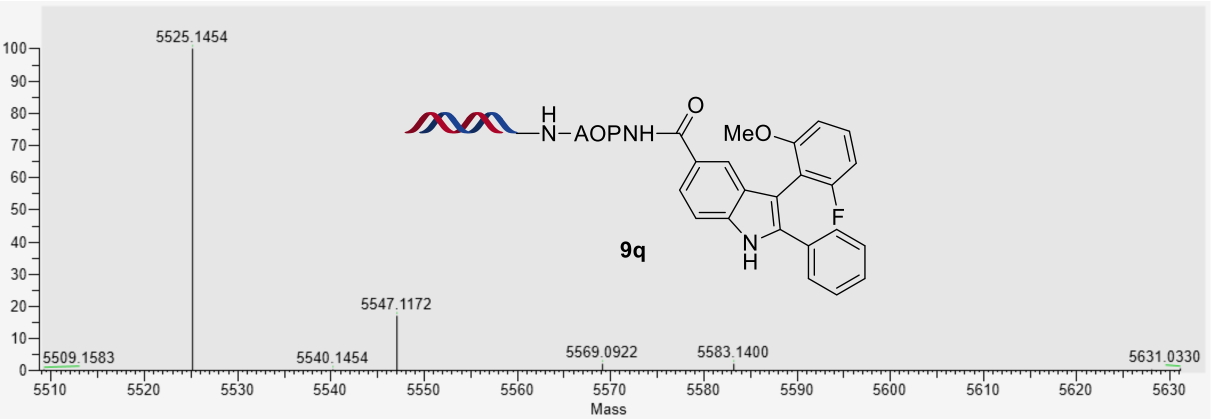

Calcd. for $C_{187}H_{250}FN_{54}O_{108}P_{17}$ 5525.1254; found 5525.1454

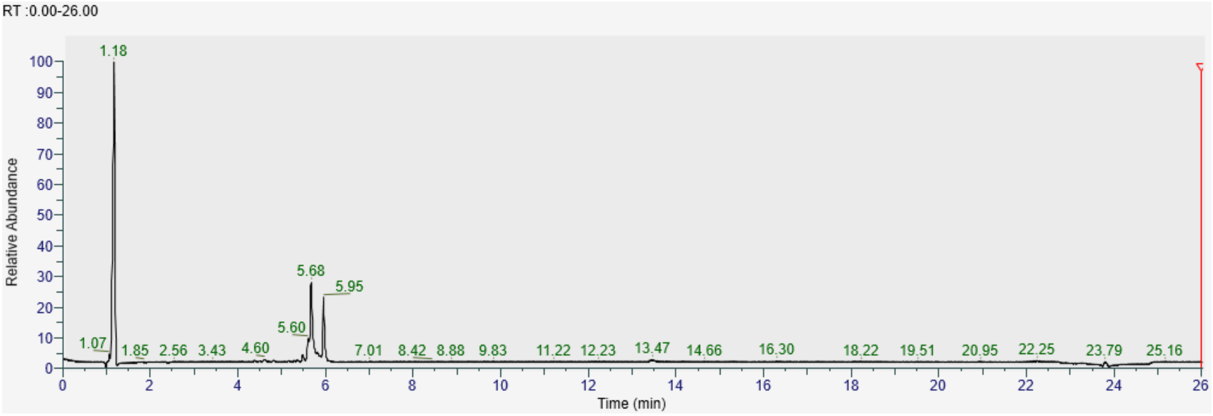

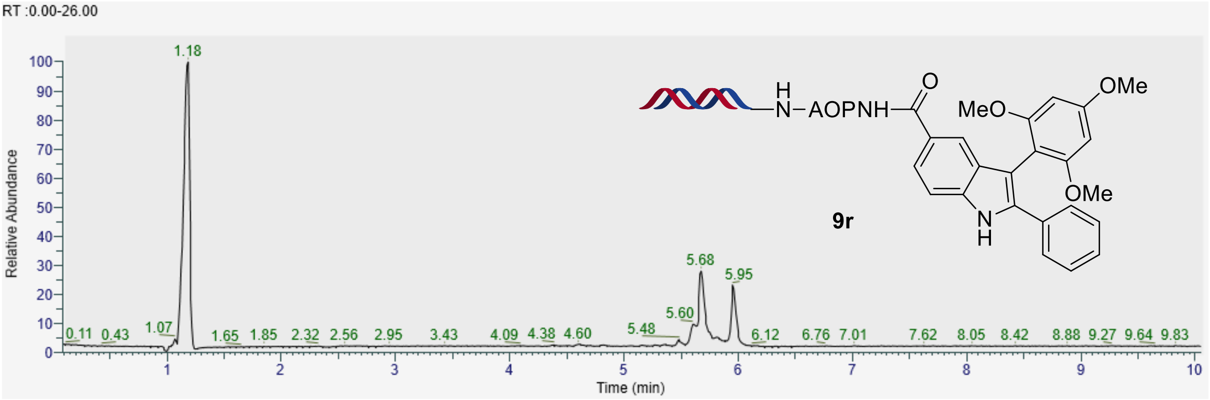

Calcd. for $C_{189}H_{257}N_{54}O_{110}P_{17}$ 5569.1716; No product detected.

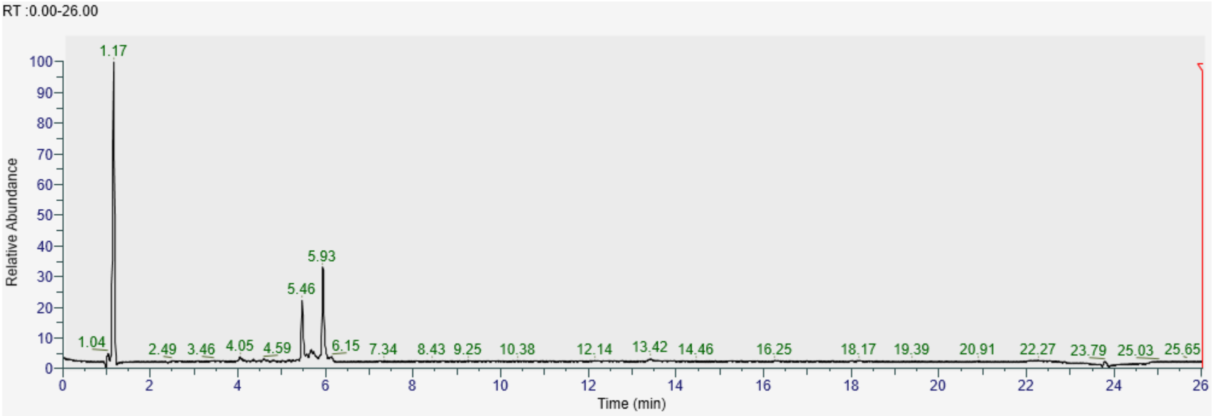

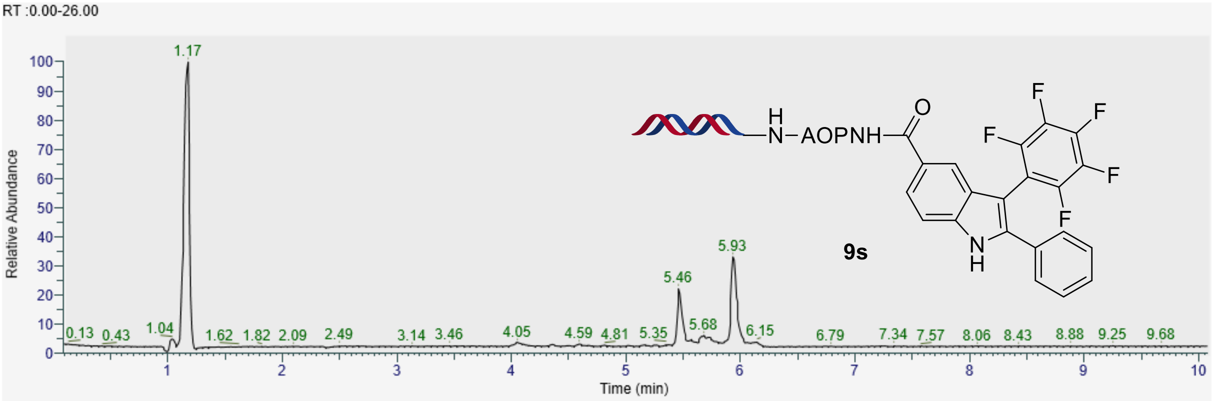

Calcd. for $C_{186}H_{244}N_{54}O_{107}P_{17}F_5$ 5567.0772; No product detected.

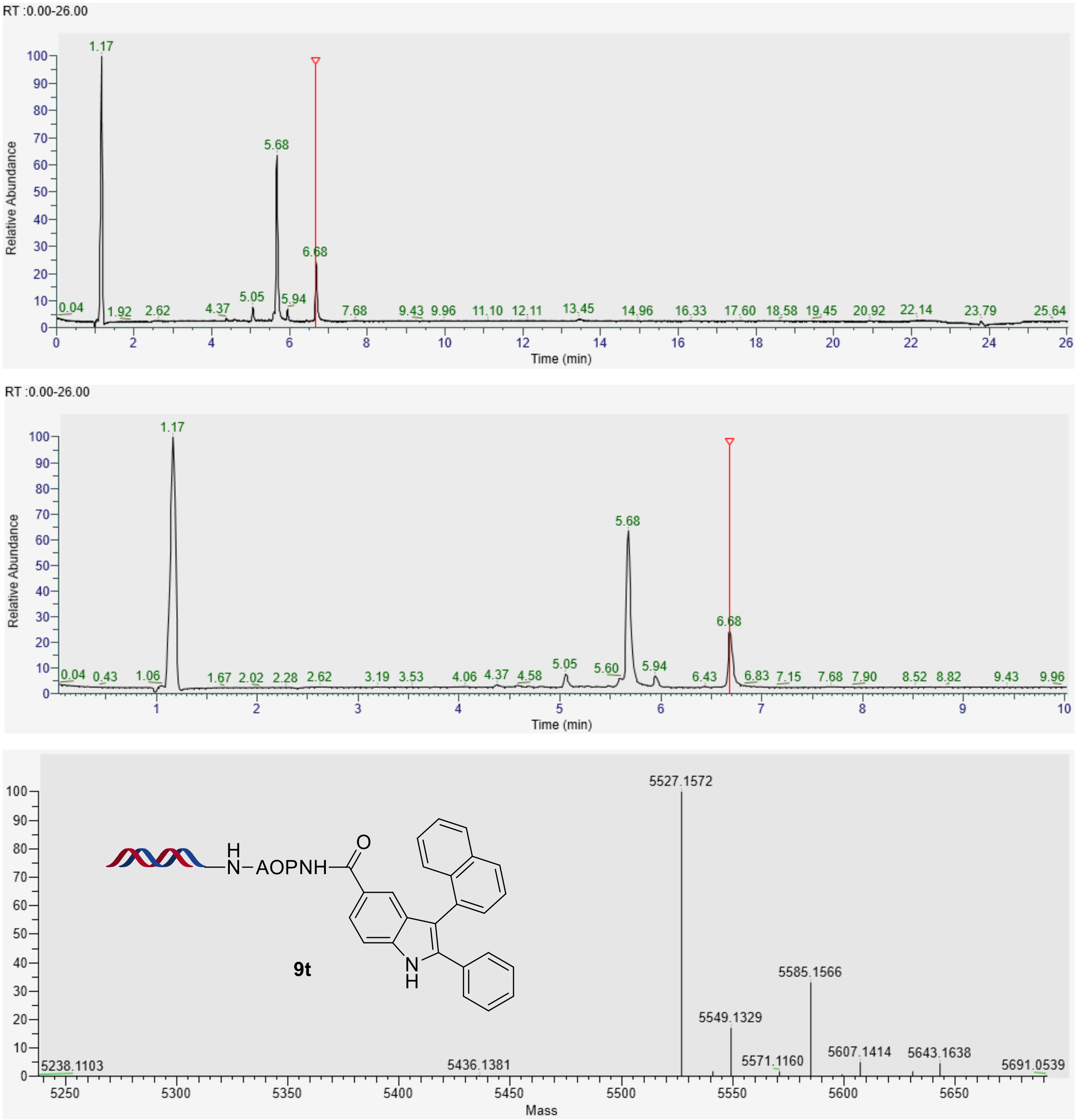

Calcd. for $C_{190}H_{251}N_{54}O_{107}P_{17}$ 5527.1399; found 5527.1572

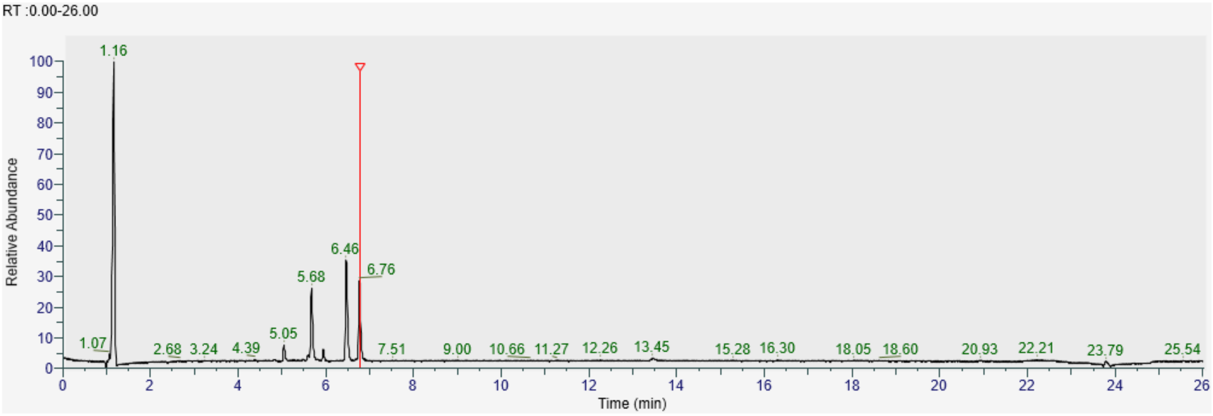

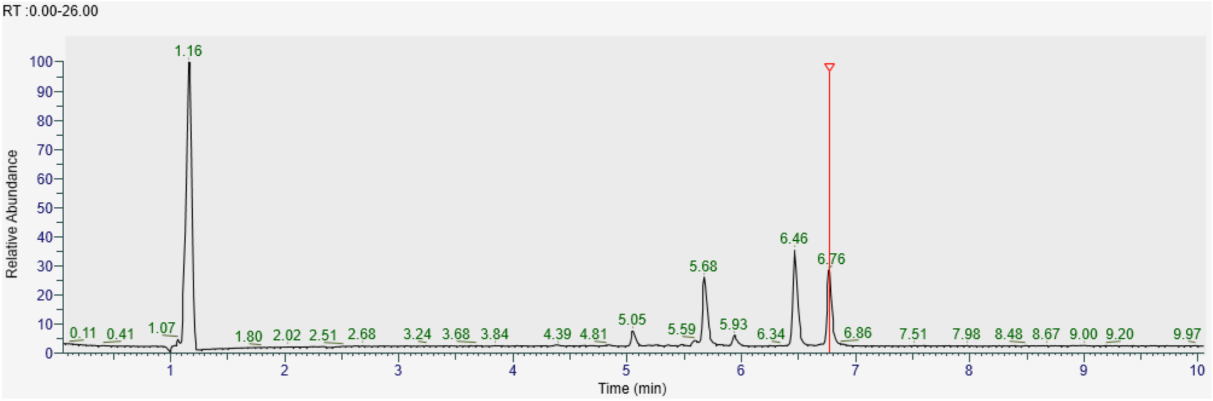

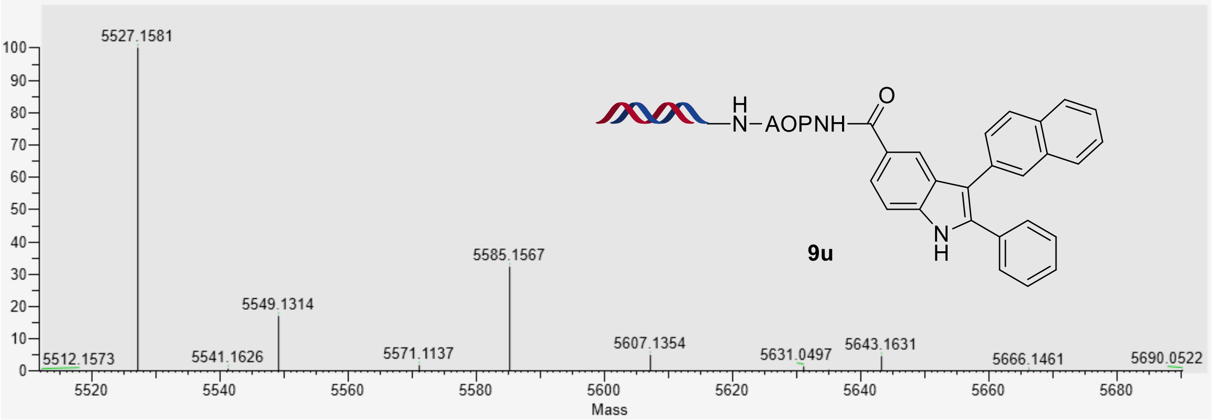

Calcd. for $C_{190}H_{251}N_{54}O_{107}P_{17}$ 5527.1399; found 5527.1581

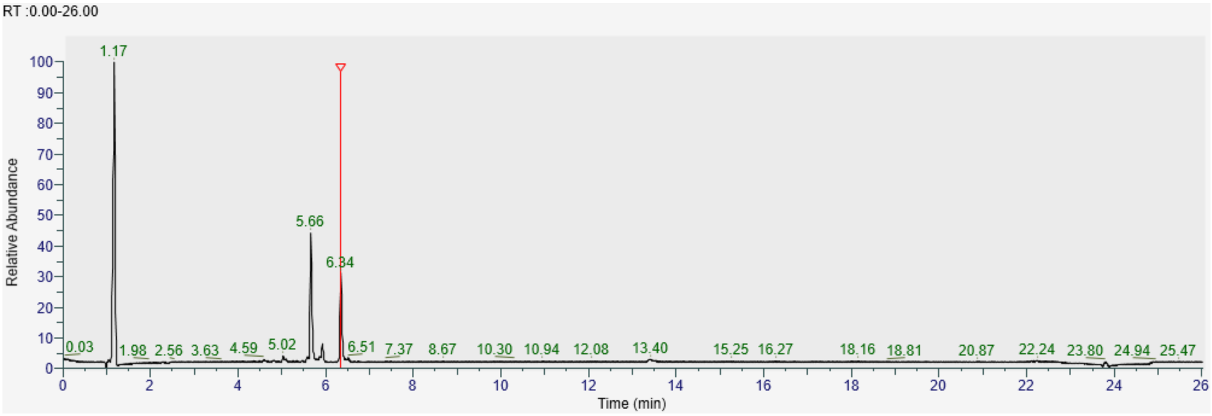

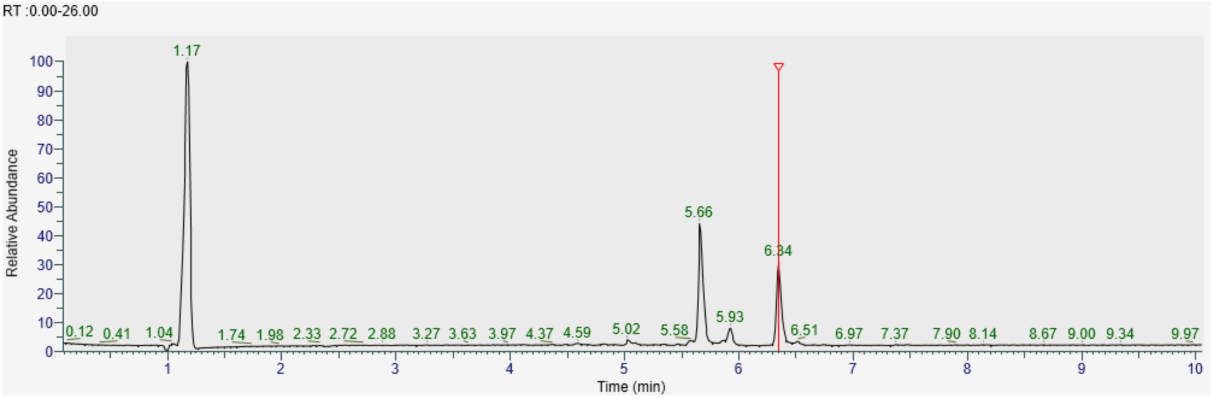

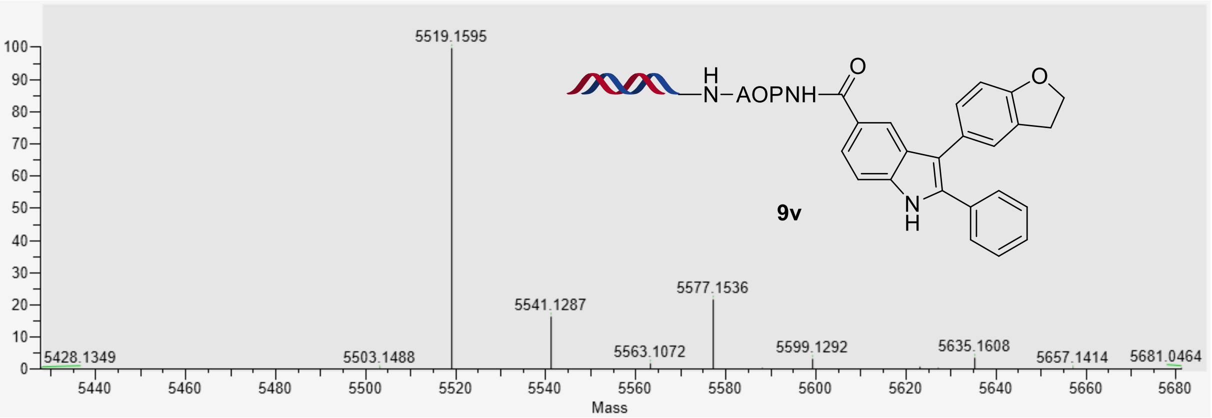

Calcd. for $C_{188}H_{251}N_{54}O_{108}P_{17}$ 5519.1348; found 5519.1595

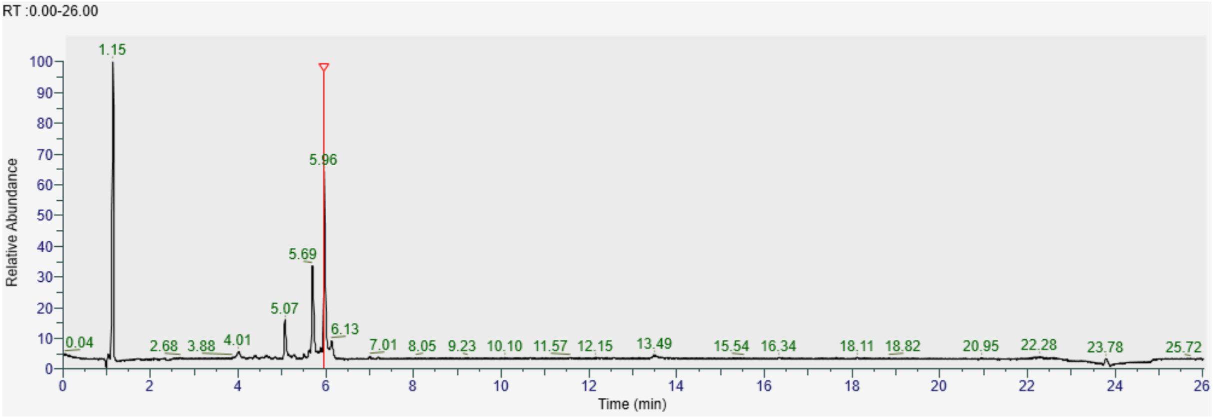

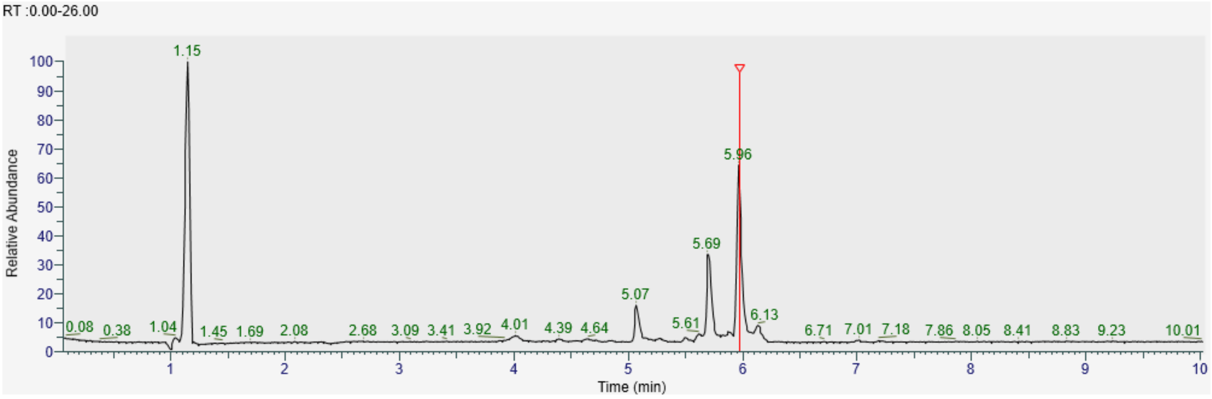

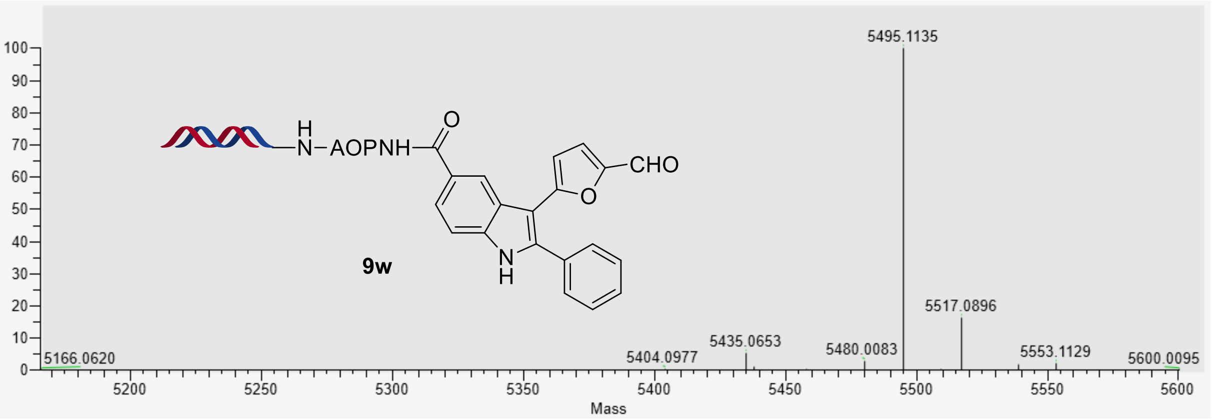

Calcd. for $C_{185}H_{247}N_{54}O_{109}P_{17}$ 5495.0985; found 5495.1135

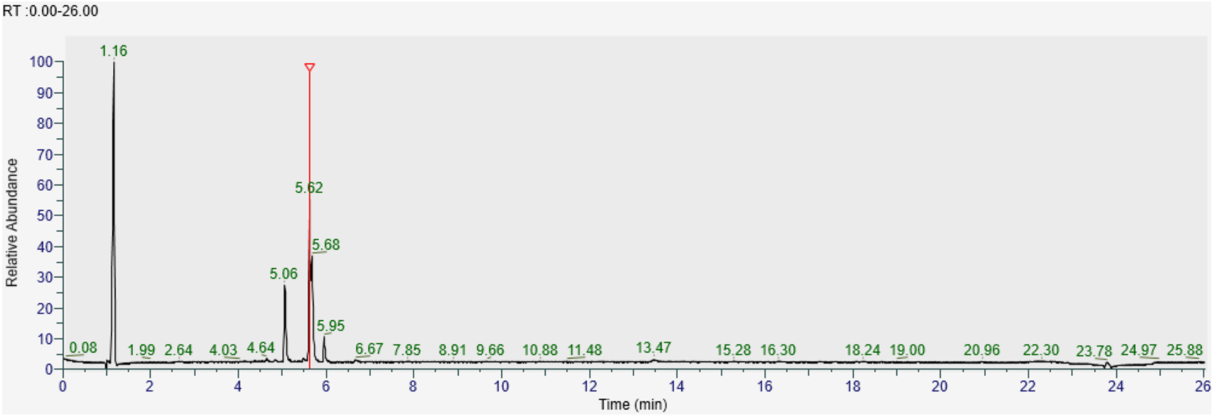

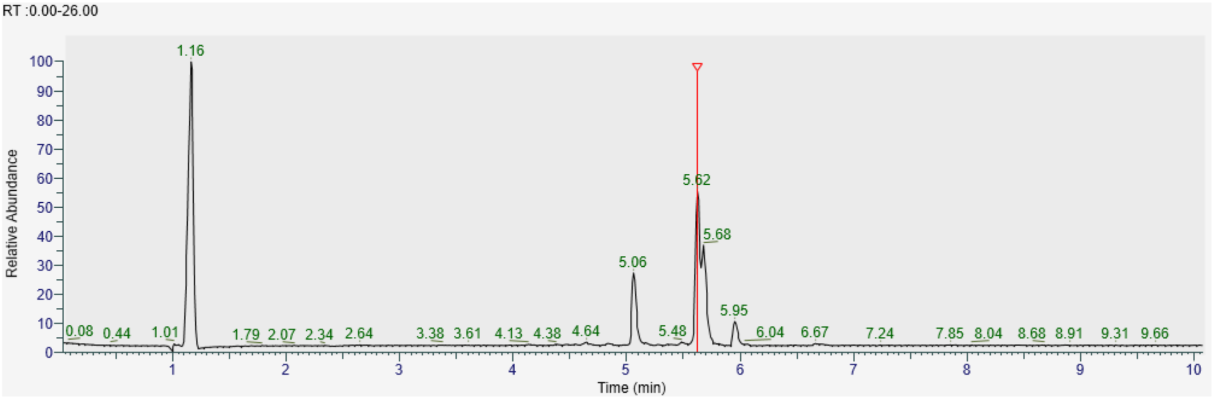

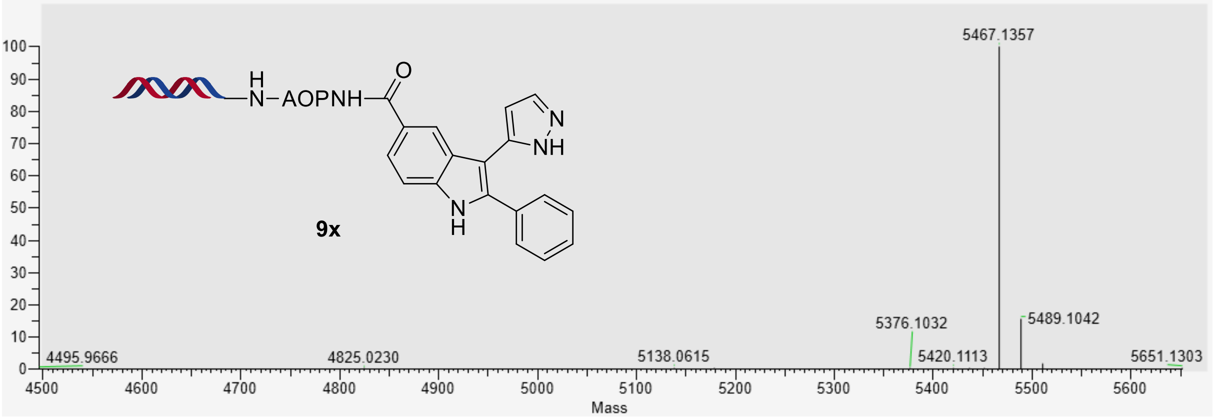

Calcd. for $C_{183}H_{247}N_{56}O_{107}P_{17}$ 5467.1148; found 5467.1357

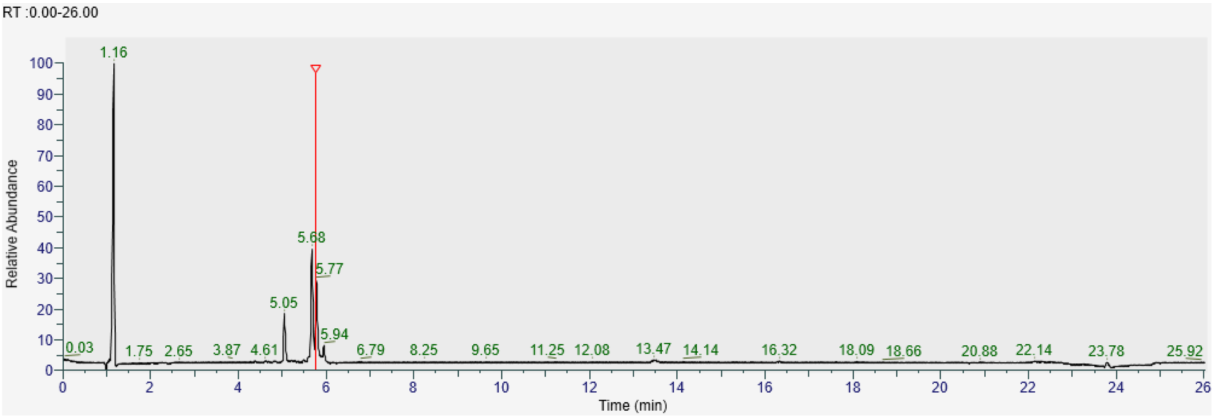

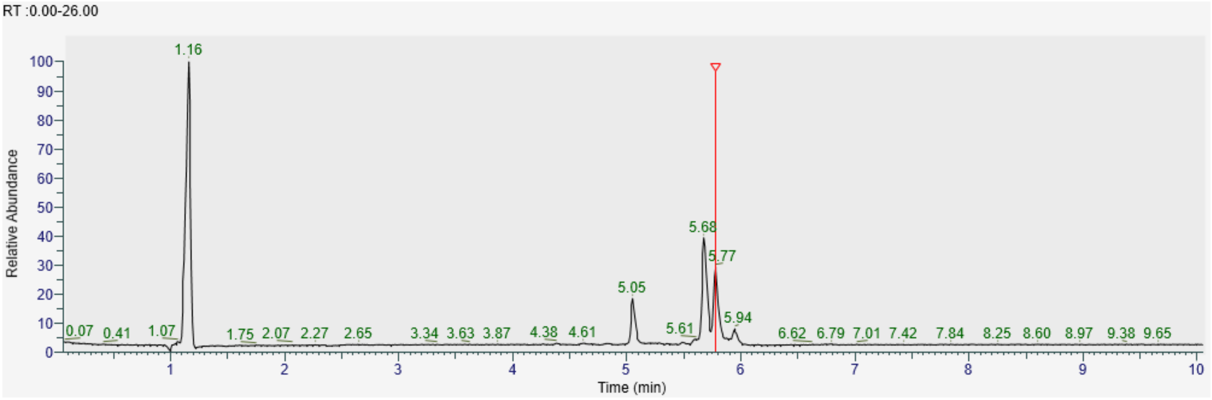

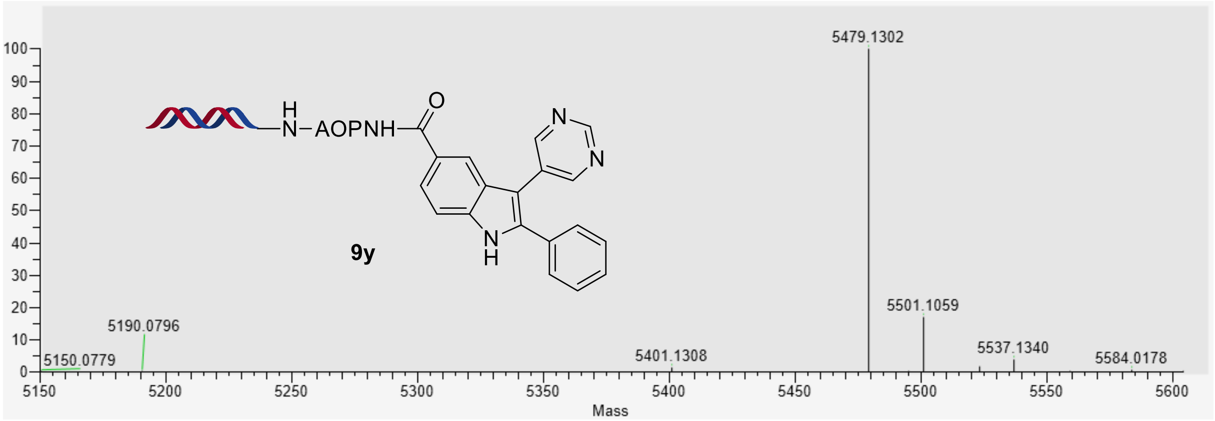

Calcd. for $C_{184}H_{247}N_{56}O_{107}P_{17}$ 5479.1148; found 5479.1302

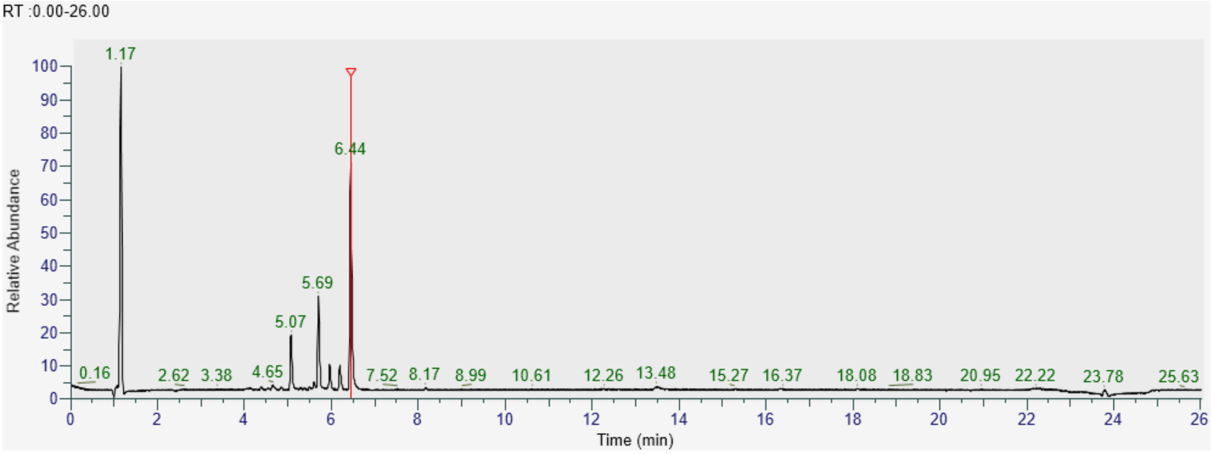

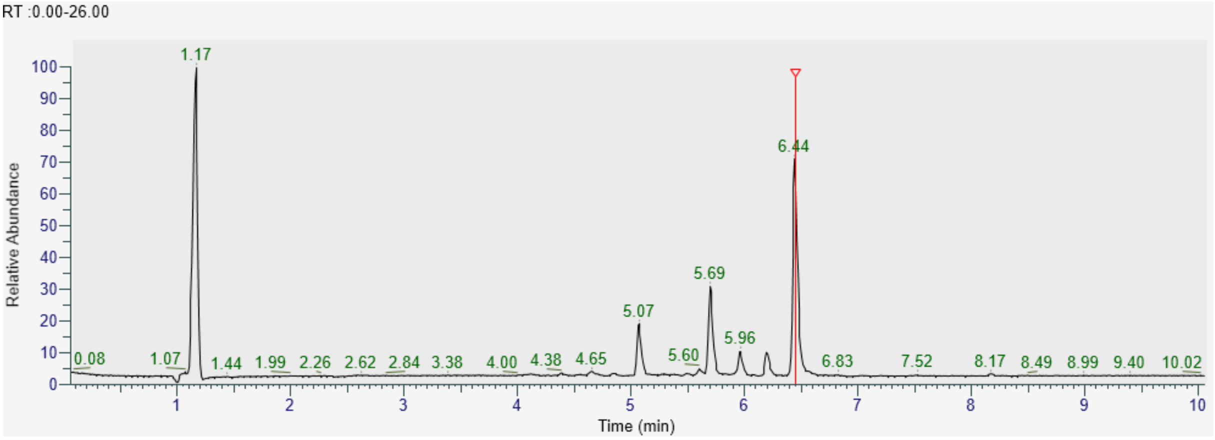

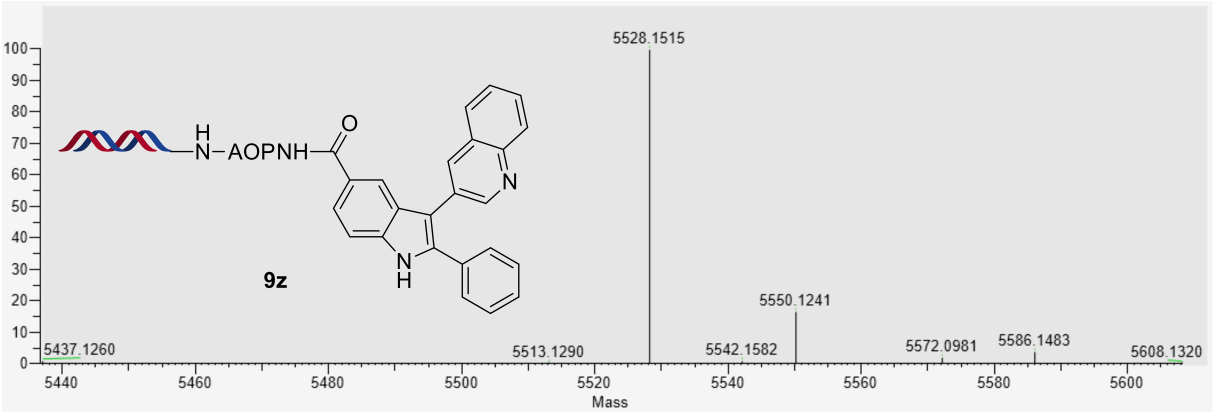

Calcd. for $C_{189}H_{250}N_{55}O_{107}P_{17}$ 5528.1352; found 5528.1515

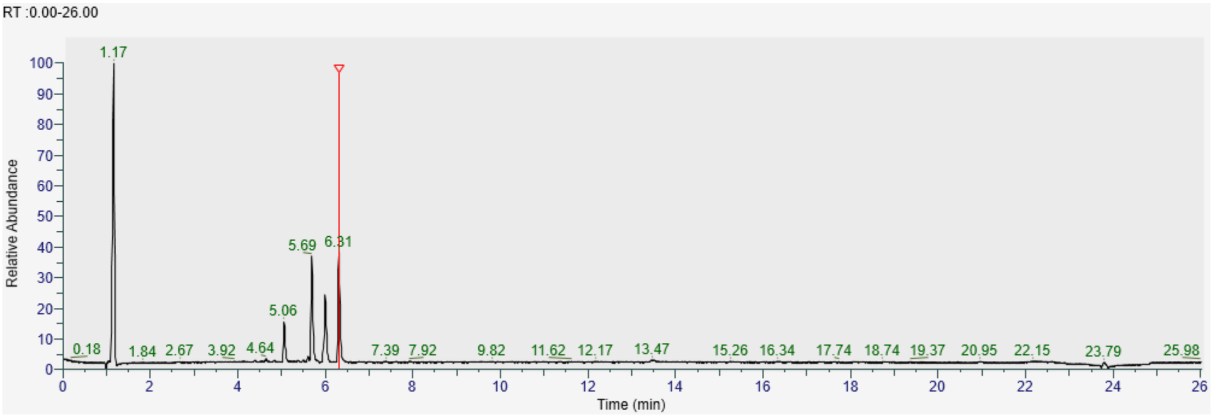

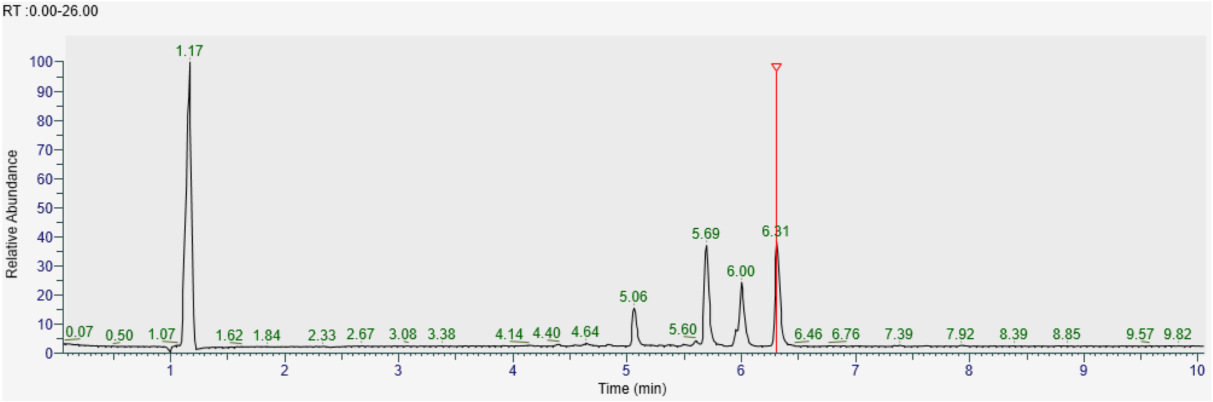

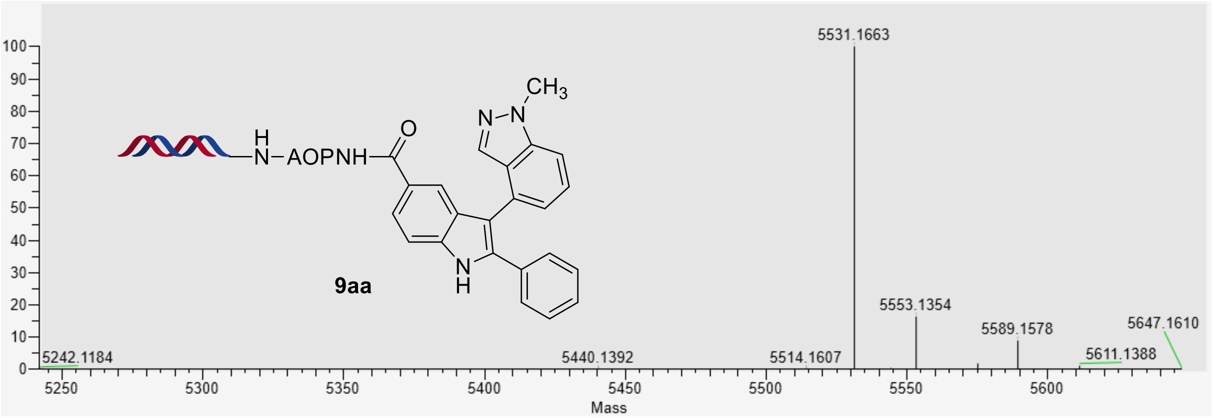

Calcd. for $C_{188}H_{251}N_{56}O_{107}P_{17}$ 5531.1461; found 5531.1663

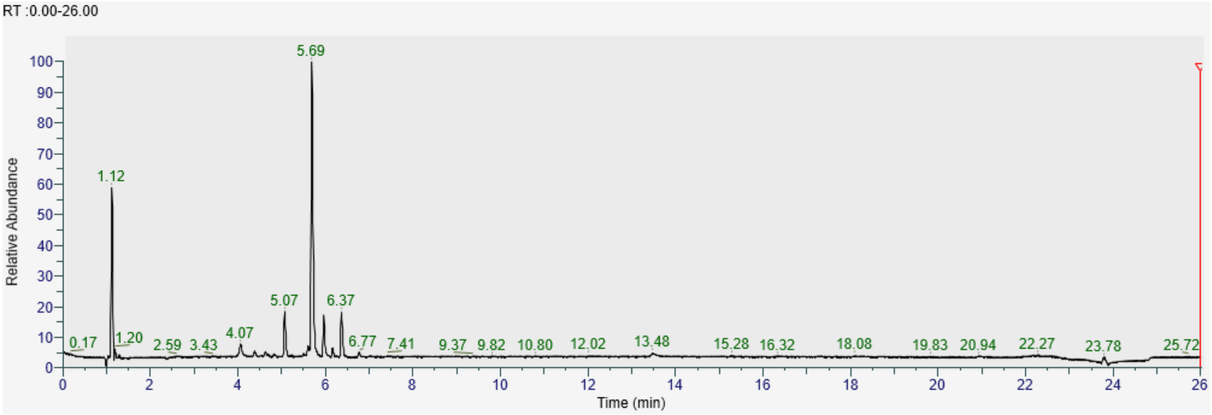

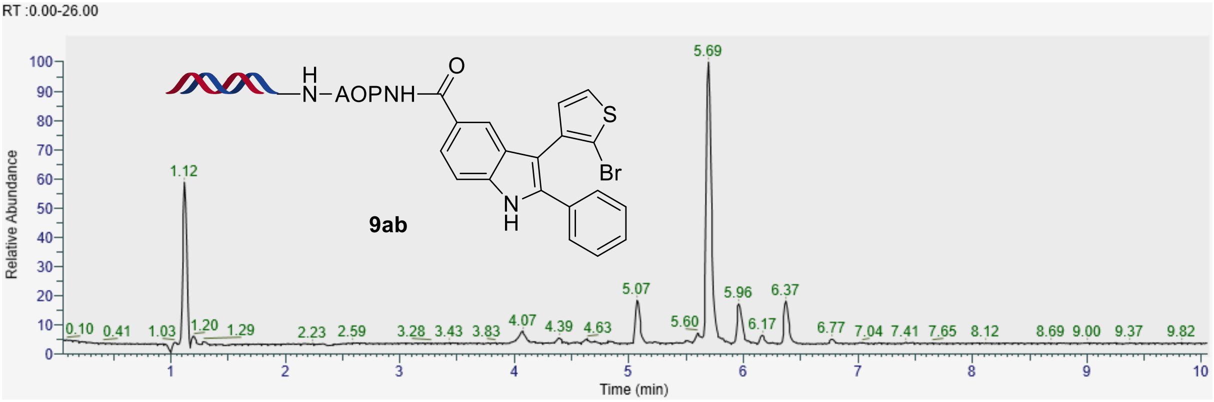

Calcd. for $C_{184}H_{246}N_{54}O_{107}P_{17}BrS$ 5560.9912; No product detected.

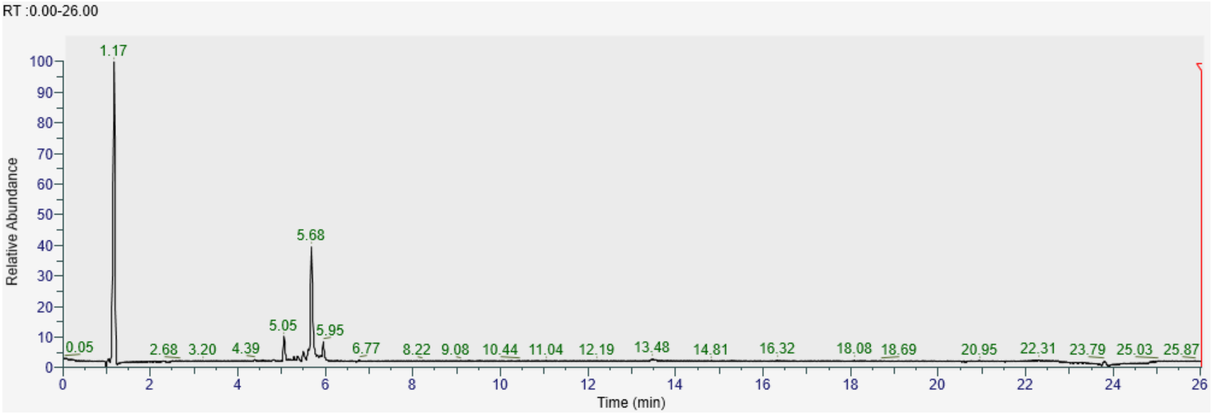

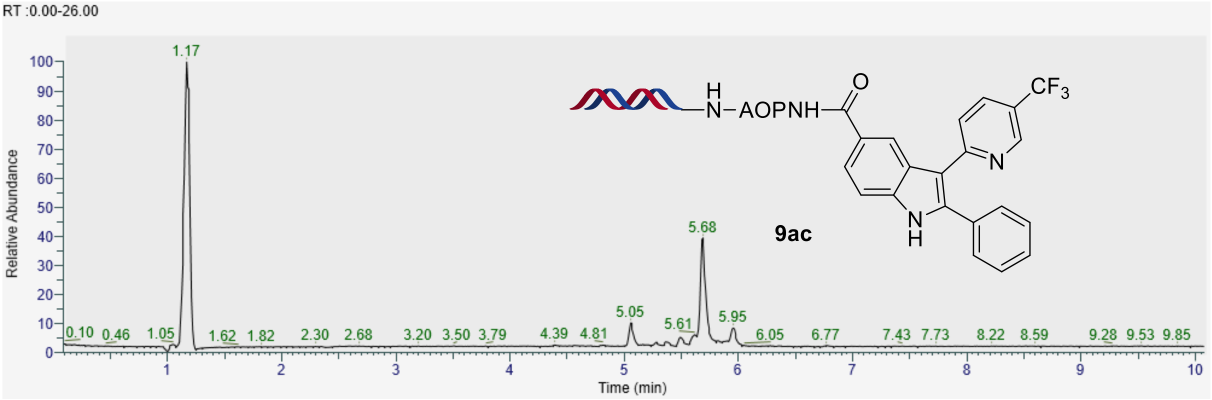

Calcd. for $C_{186}H_{247}N_{55}O_{107}P_{17}F_3$ 5546.1069; No product detected.

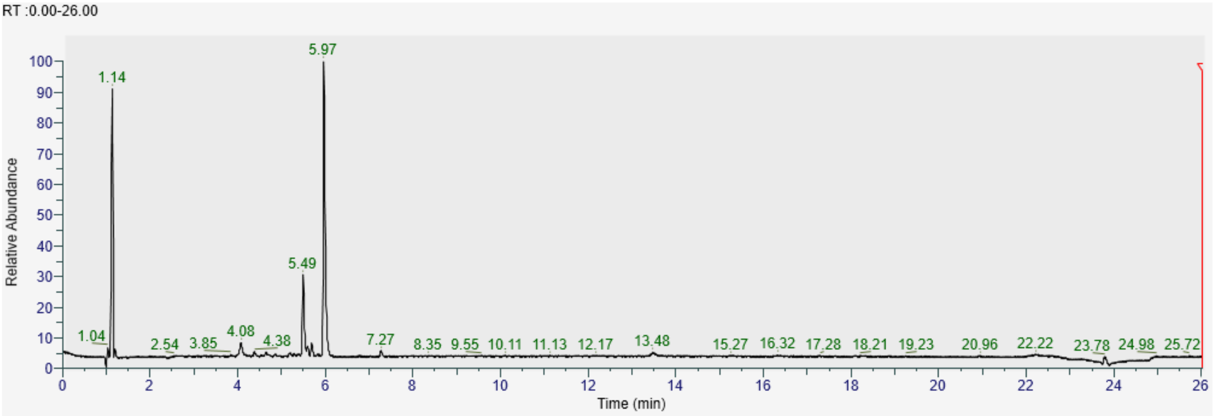

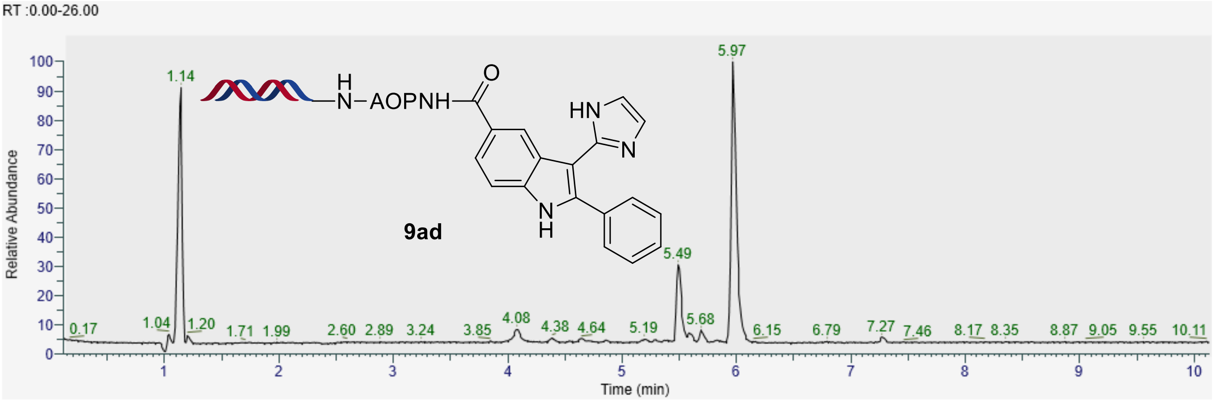

Calcd. for $C_{183}H_{247}N_{56}O_{107}P_{17}$ 5467.1148; No product detected.

10. NMR spectra

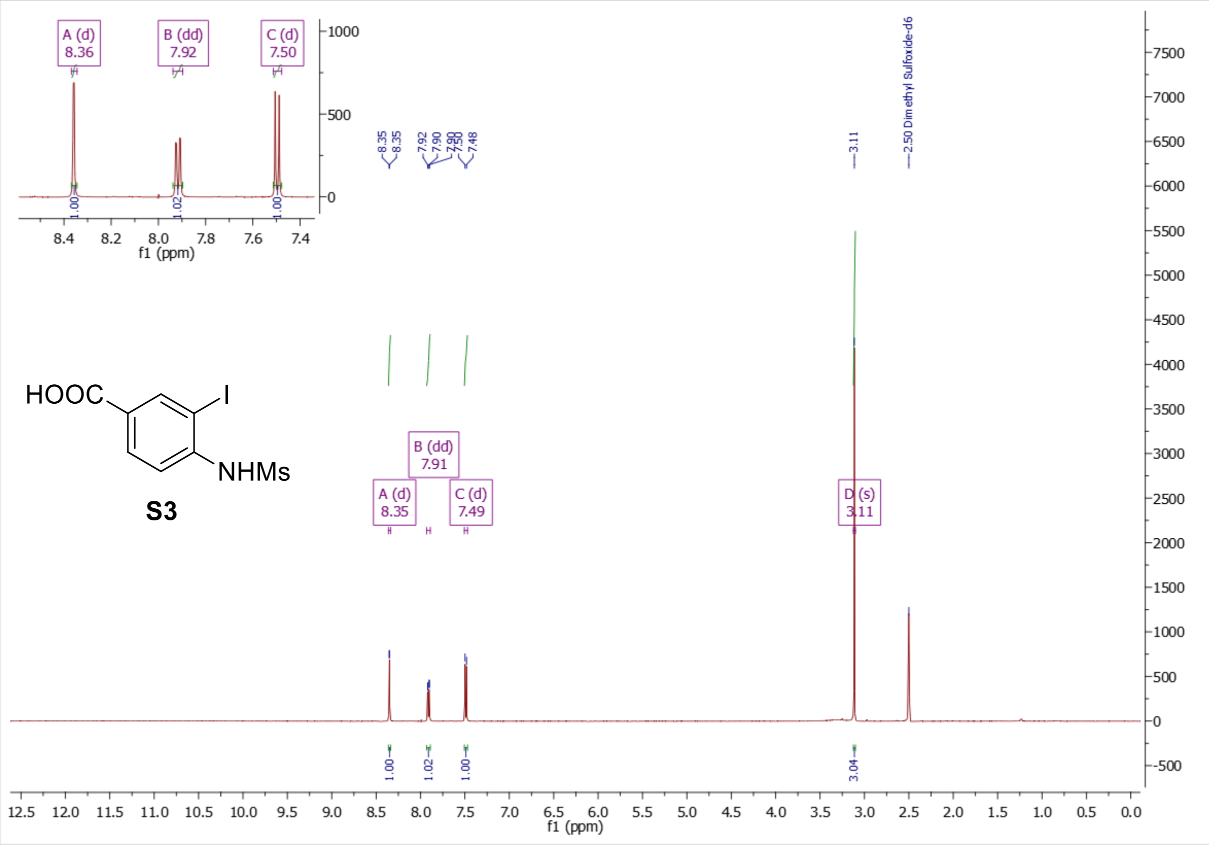

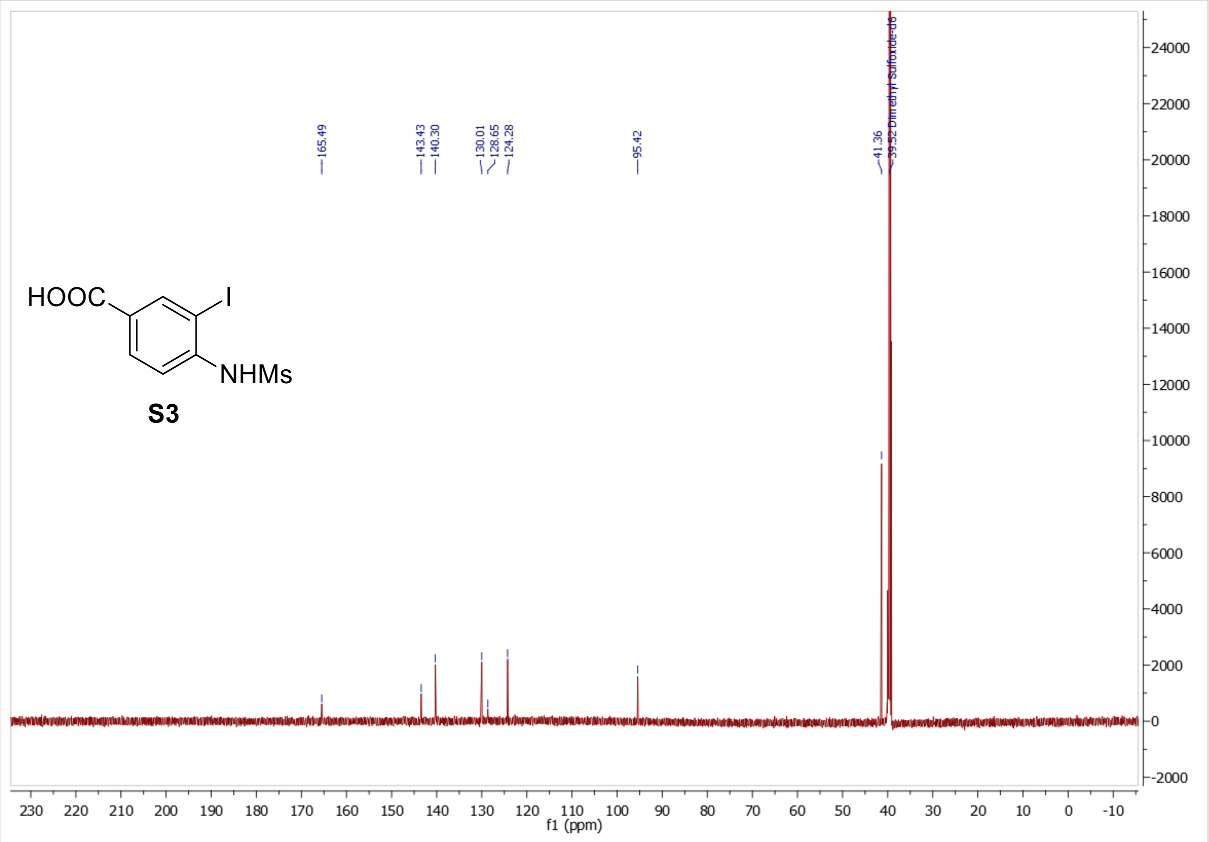

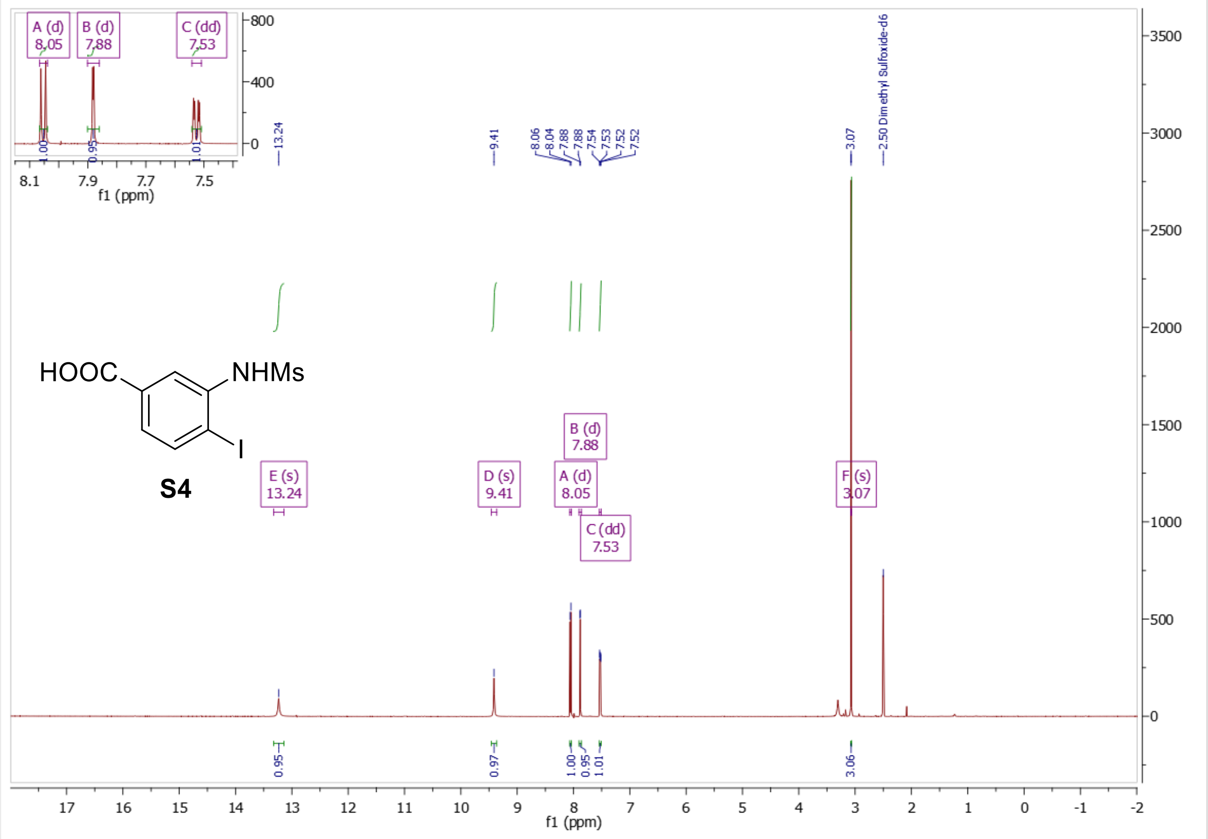

HOOC
NHMs
I
S4
A (d) 8.05
B (d) 7.88
C (dd) 7.53
D (s) 9.41
E (s) 13.24
F (s) 3.07
2.50 Dimethyl Sulfoxide-d6
f1 (ppm)

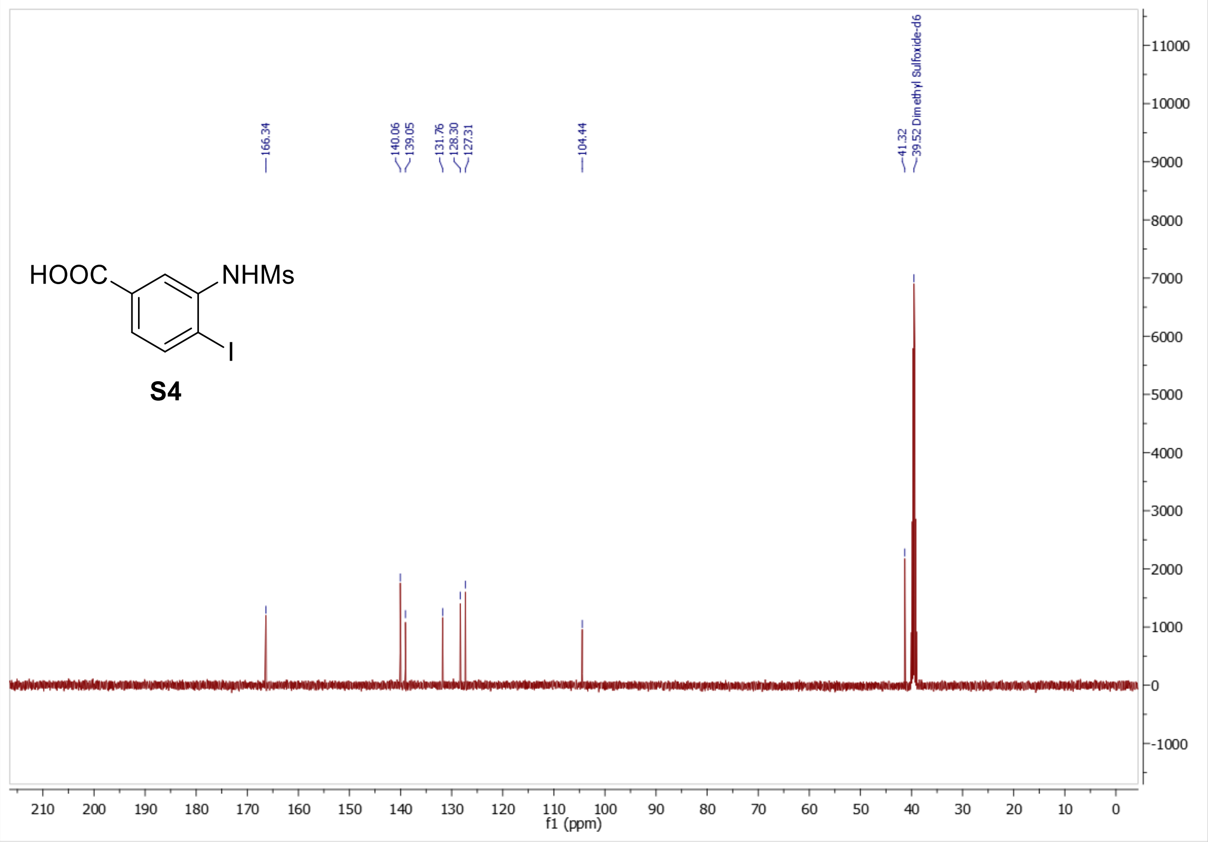

HOOC
NHMs
I
S4
166.34
140.06
139.05
131.76
128.30
127.31
104.44
41.32
39.52 Dimethyl Sulfoxide-d6
f1 (ppm)

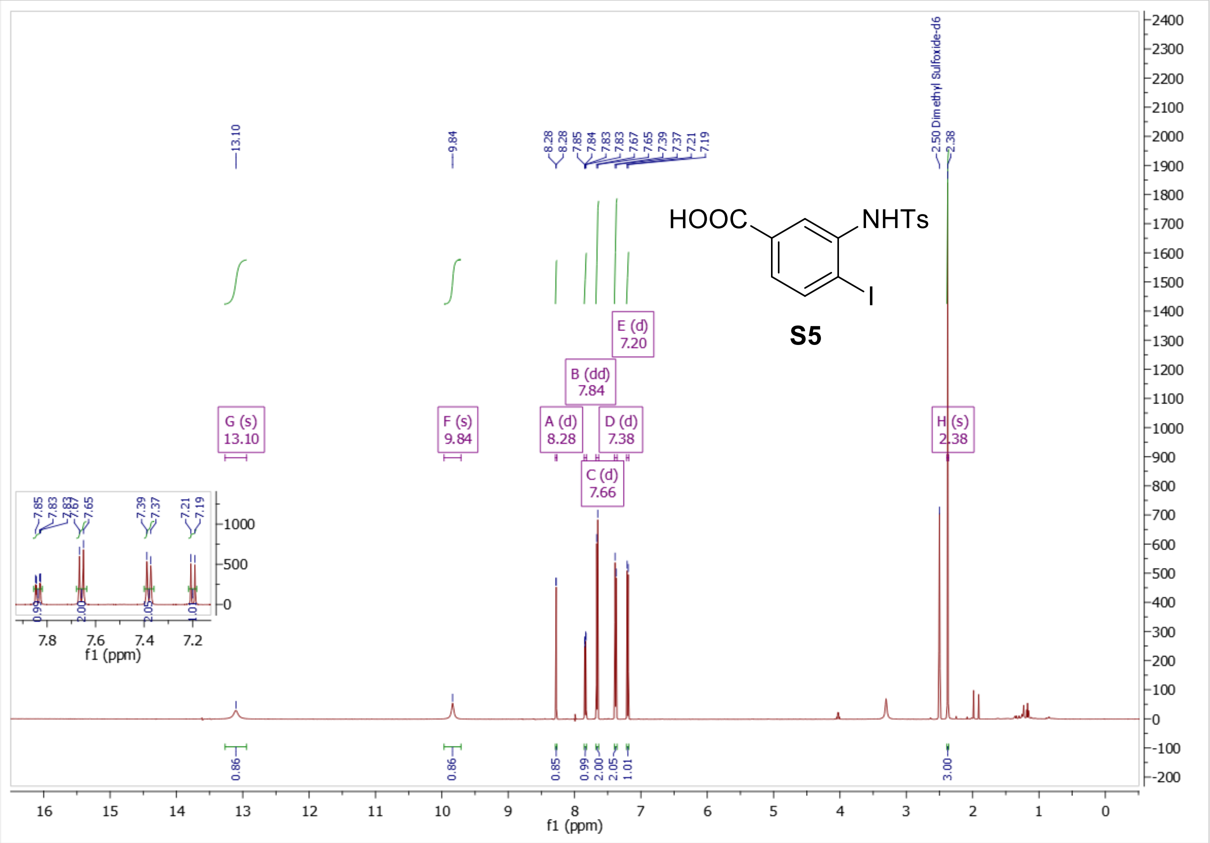
HOOC
NHTs
I
S5
f1 (ppm)

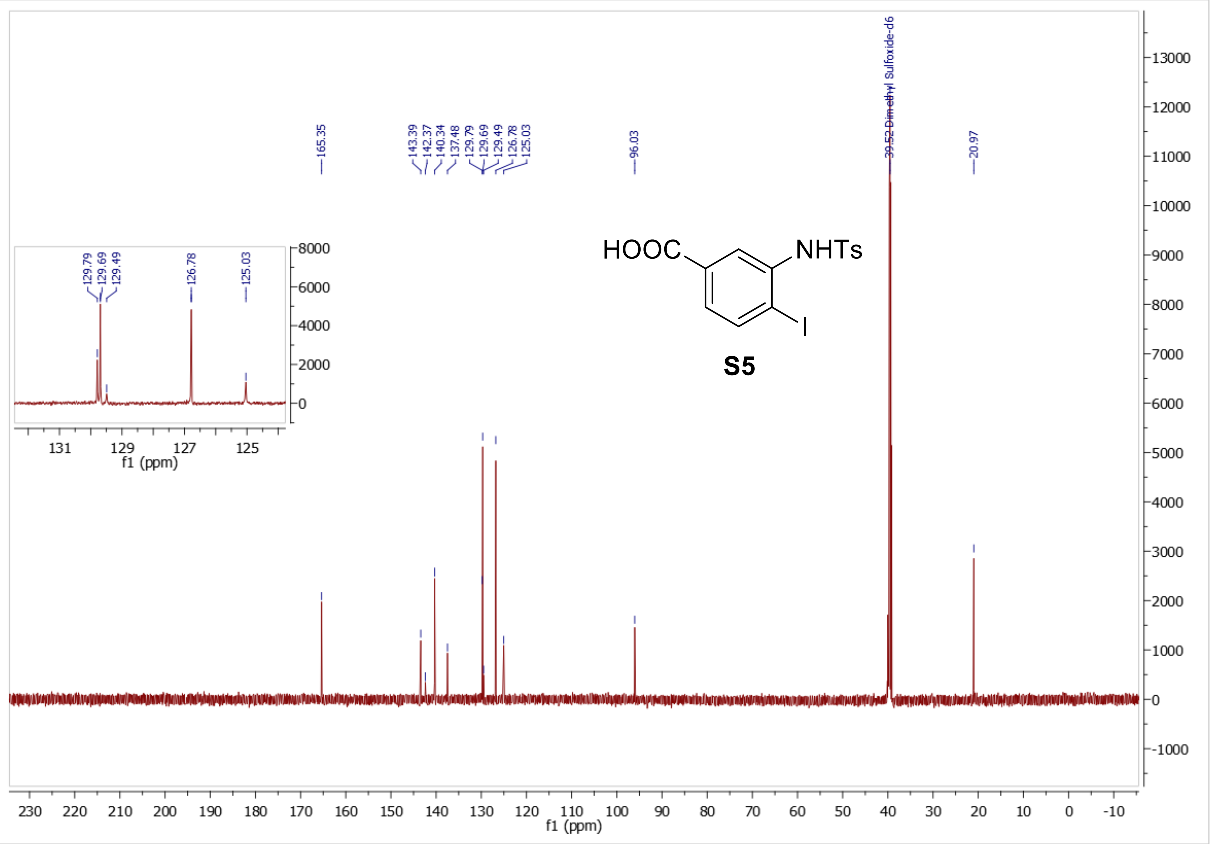
HOOC
NHTs
I
S5
f1 (ppm)

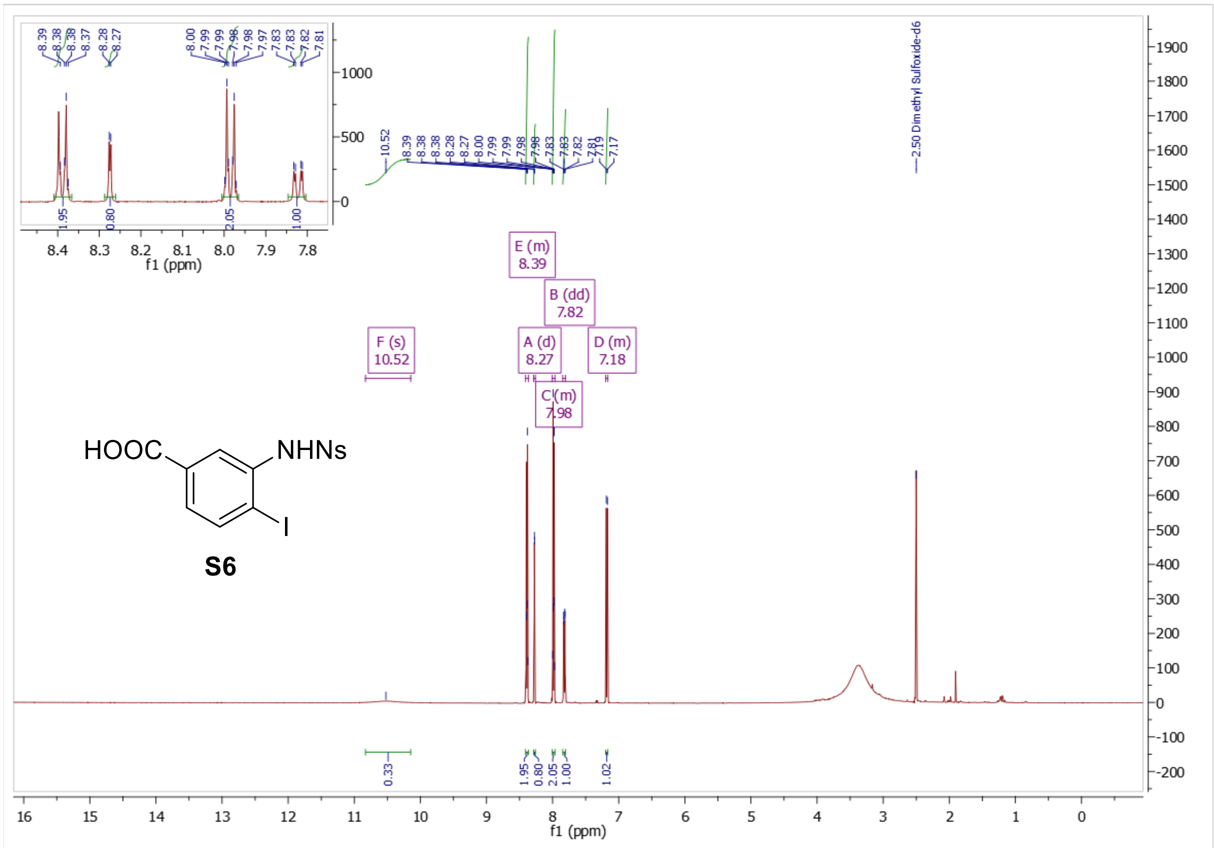
HOOC
NHNs
I
S6
F (s)
10.52
A (d)
8.27
E (m)
8.39
B (dd)
7.82
C (m)
7.98
D (m)
7.18
2.50 Dimethyl Sulfoxide-d6
f1 (ppm)

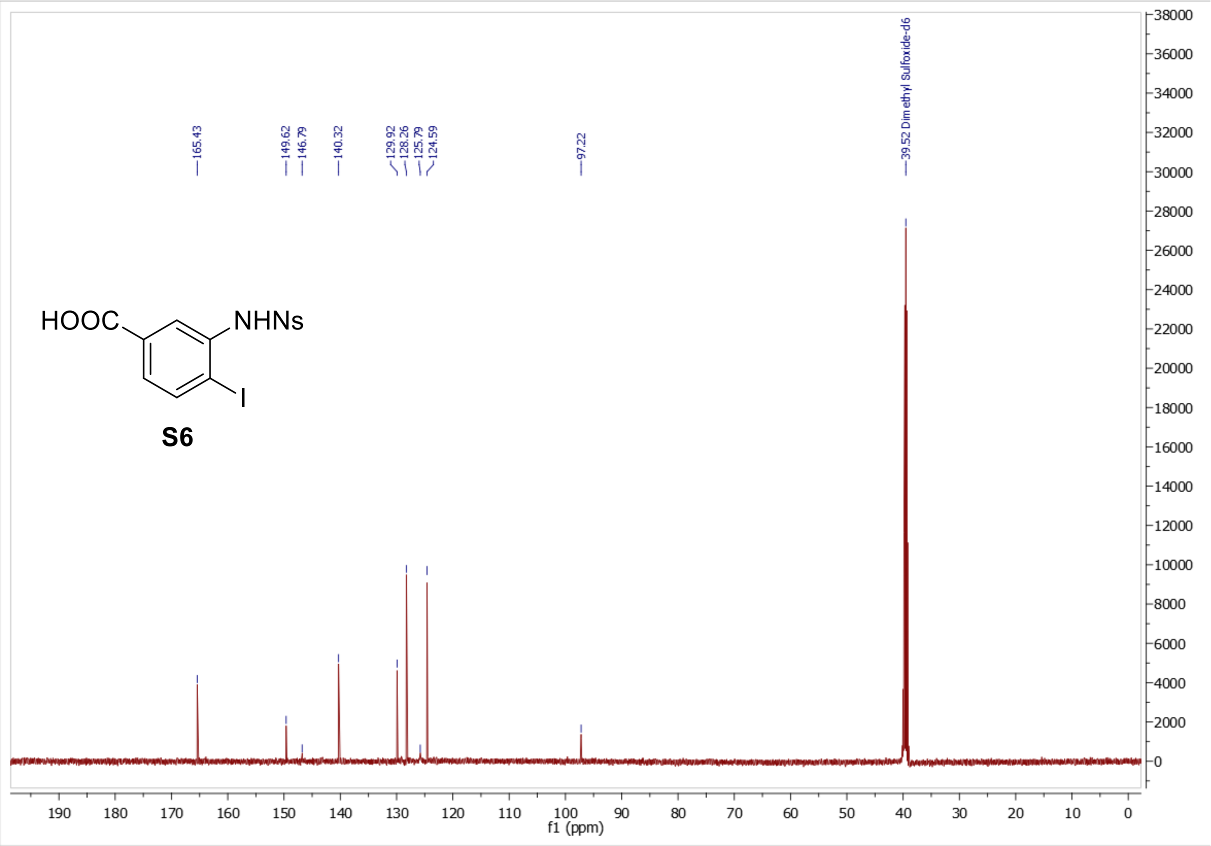
HOOC
NHNs
I
S6
165.43
149.62
146.79
140.32
129.92
128.26
125.79
124.59
97.22
39.52 Dimethyl Sulfoxide-d6
f1 (ppm)

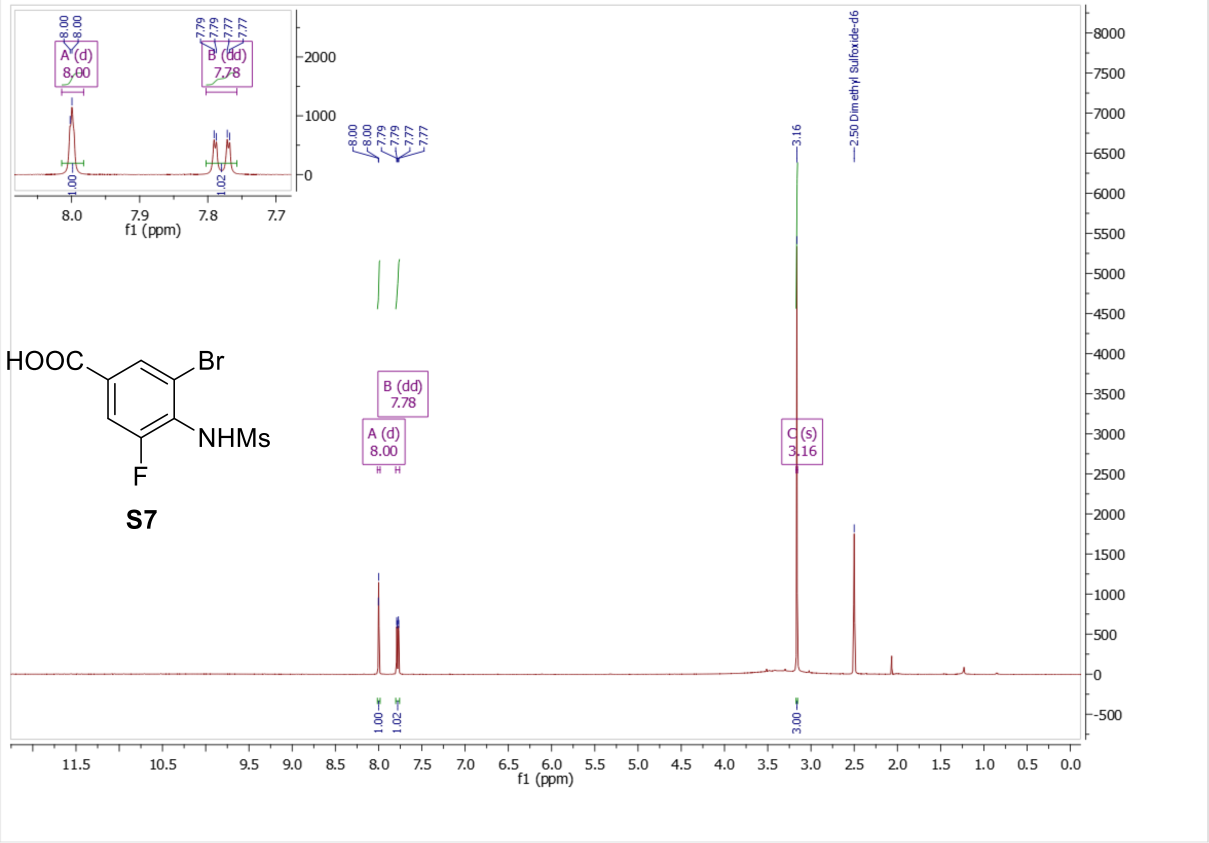

HOOC
Br
NHMs
F
S7
A (d)
8.00
B (dd)
7.78
C (s)
3.16
2.50 Dimethyl Sulfoxide-d6
f1 (ppm)

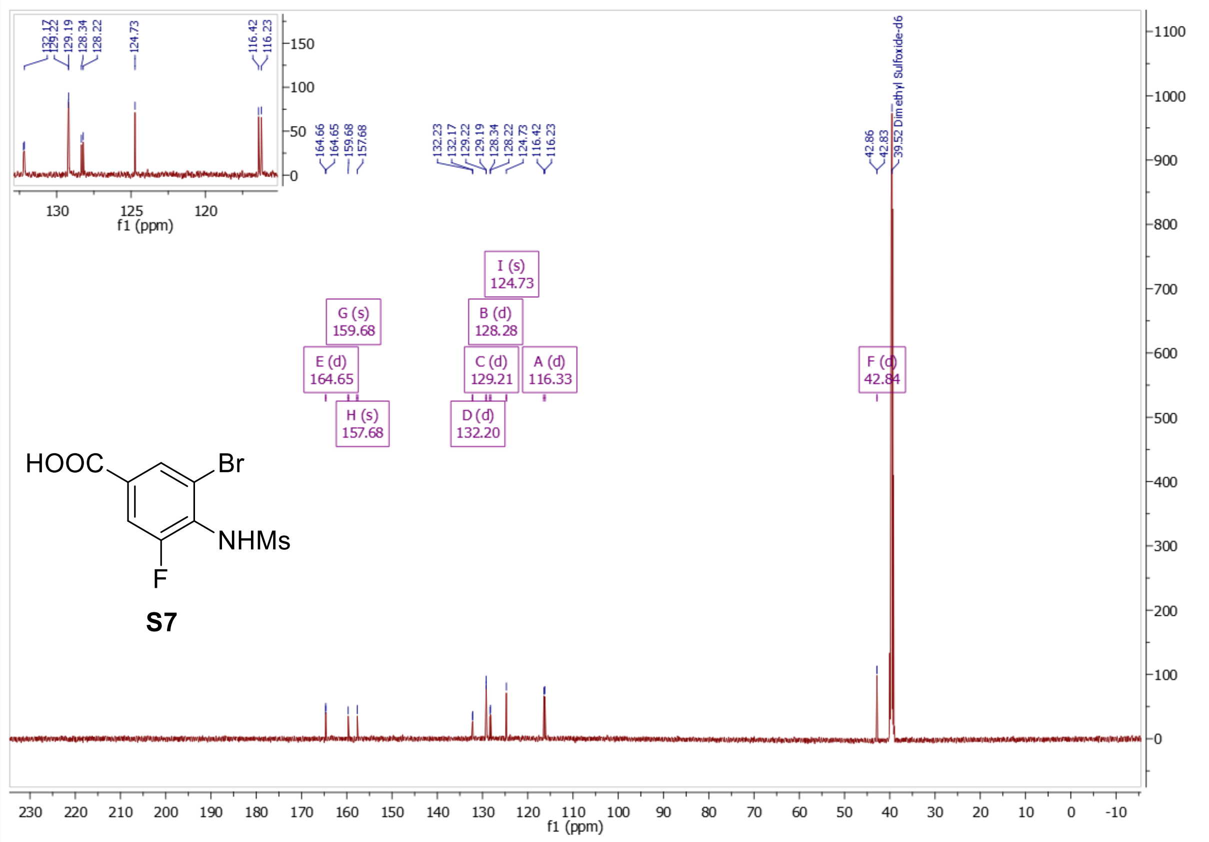

HOOC
Br
NHMs
F
S7
E (d)
164.65
G (s)
159.68
H (s)
157.68
D (d)
132.20
C (d)
129.21
B (d)
128.28
I (s)
124.73
A (d)
116.33
F (d)
42.84
39.52 Dimethyl Sulfoxide-d6
f1 (ppm)

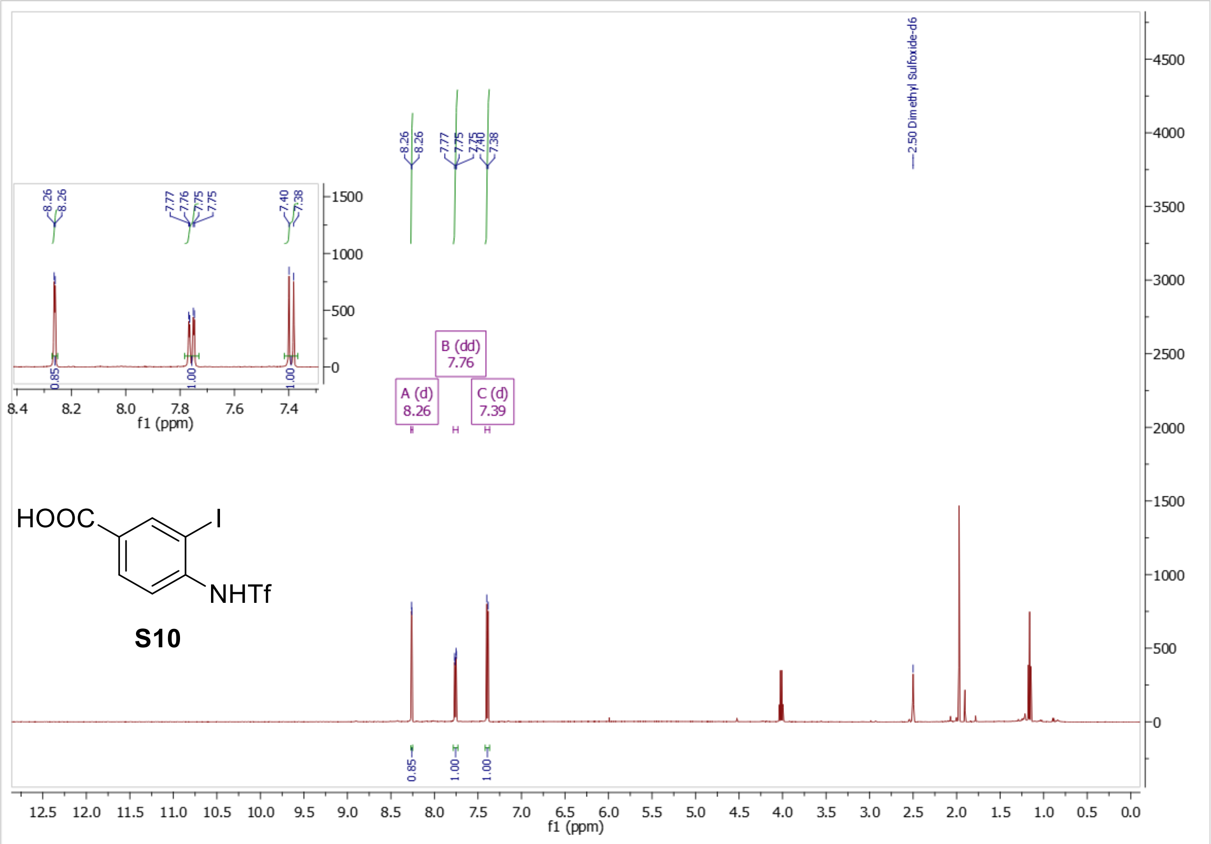
2.50 Dimethyl Sulfoxide-d6
8.26
8.26
7.77
7.75
7.40
7.38
A (d)
8.26
B (dd)
7.76
C (d)
7.39
0.85
1.00
1.00
HOOC
I
NHTf
S10
f1 (ppm)

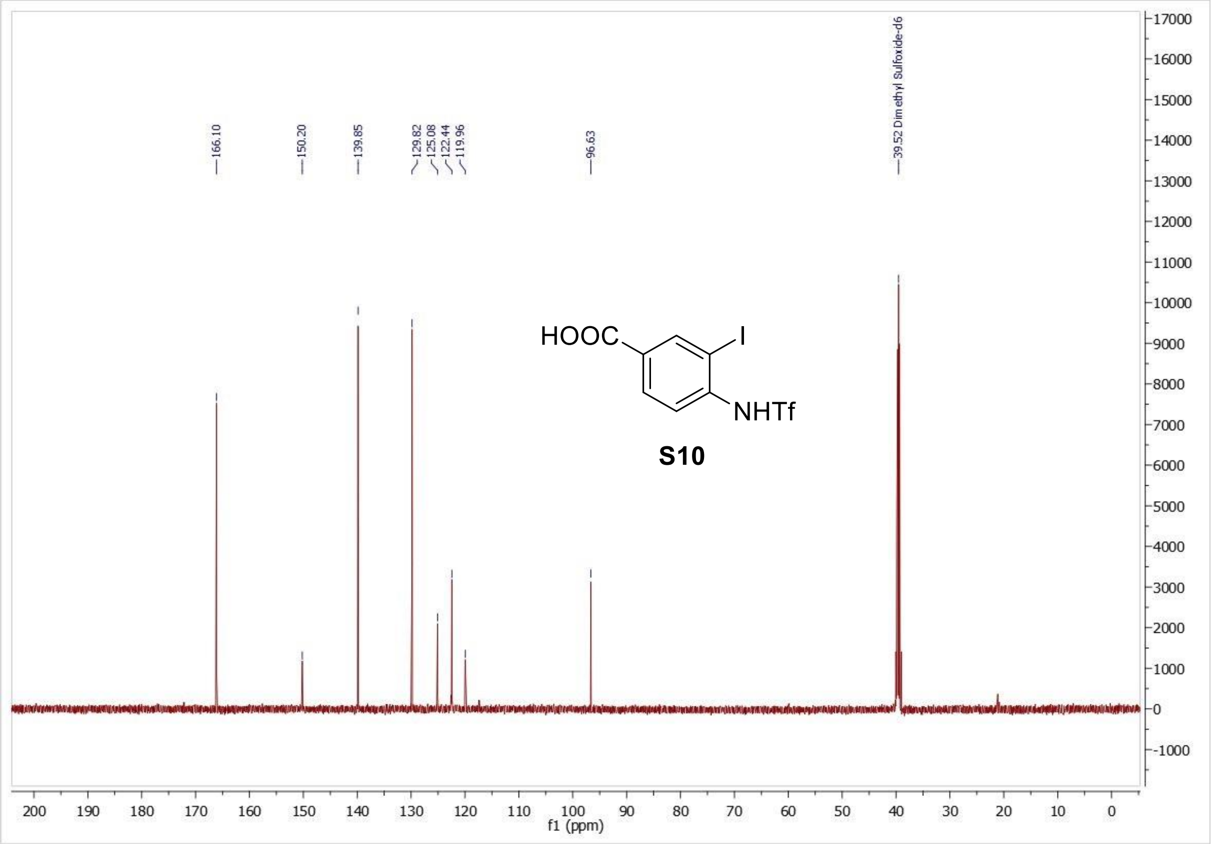
166.10
150.20
139.85
129.82
125.08
122.44
119.96
96.63
39.52 Dimethyl Sulfoxide-d6
HOOC
I
NHTf
S10
f1 (ppm)

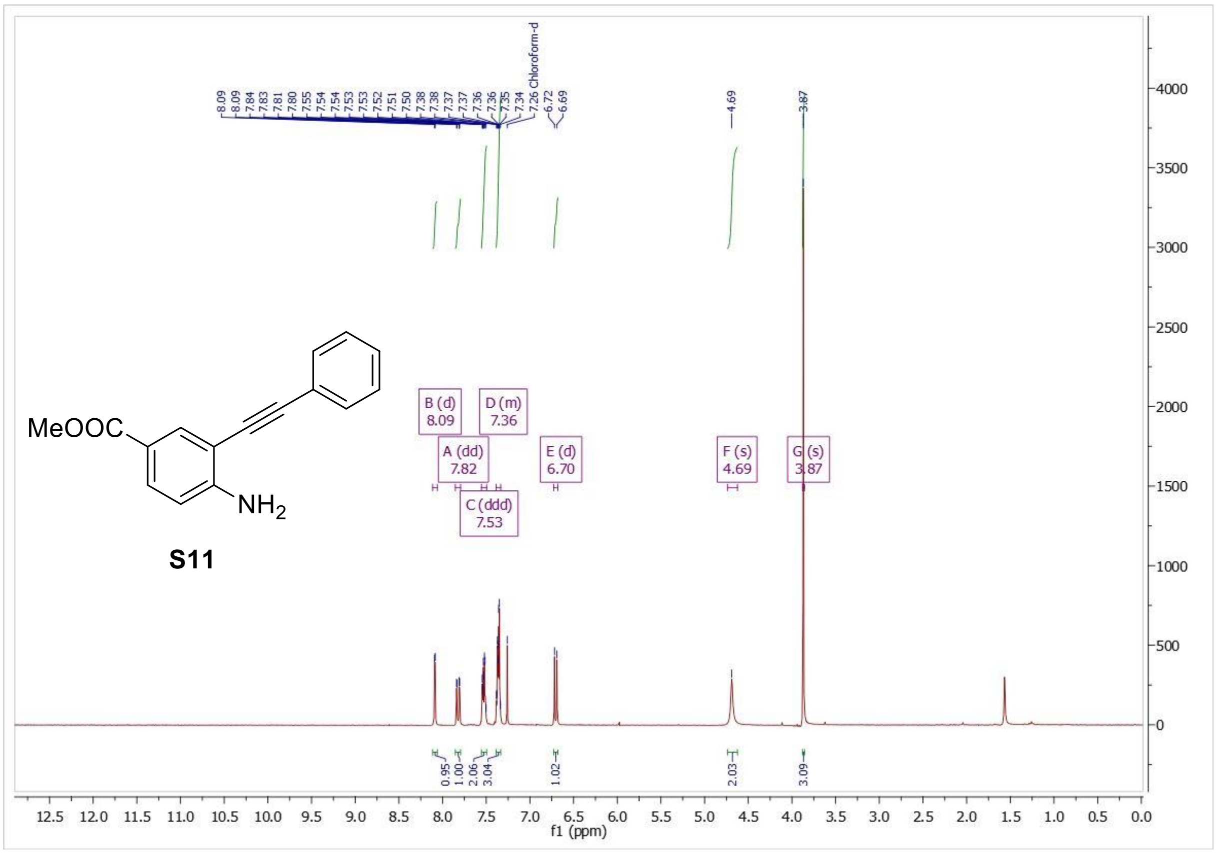
MeOOC
NH2
S11
B (d) 8.09
D (m) 7.36
A (dd) 7.82
C (ddd) 7.53
E (d) 6.70
F (s) 4.69
G (s) 3.87
7.26 Chloroform-d
0.95
1.00
2.06
3.04
1.02
2.03
3.09
f1 (ppm)

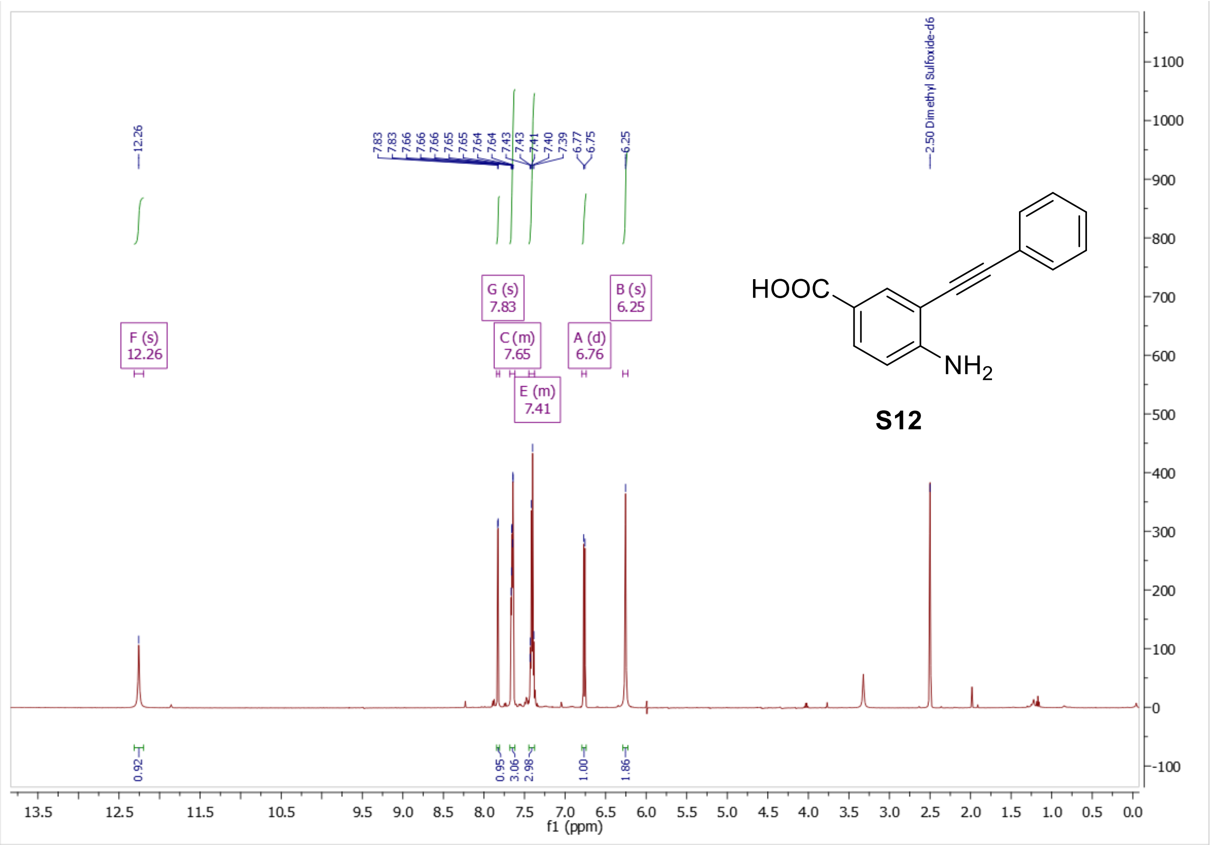

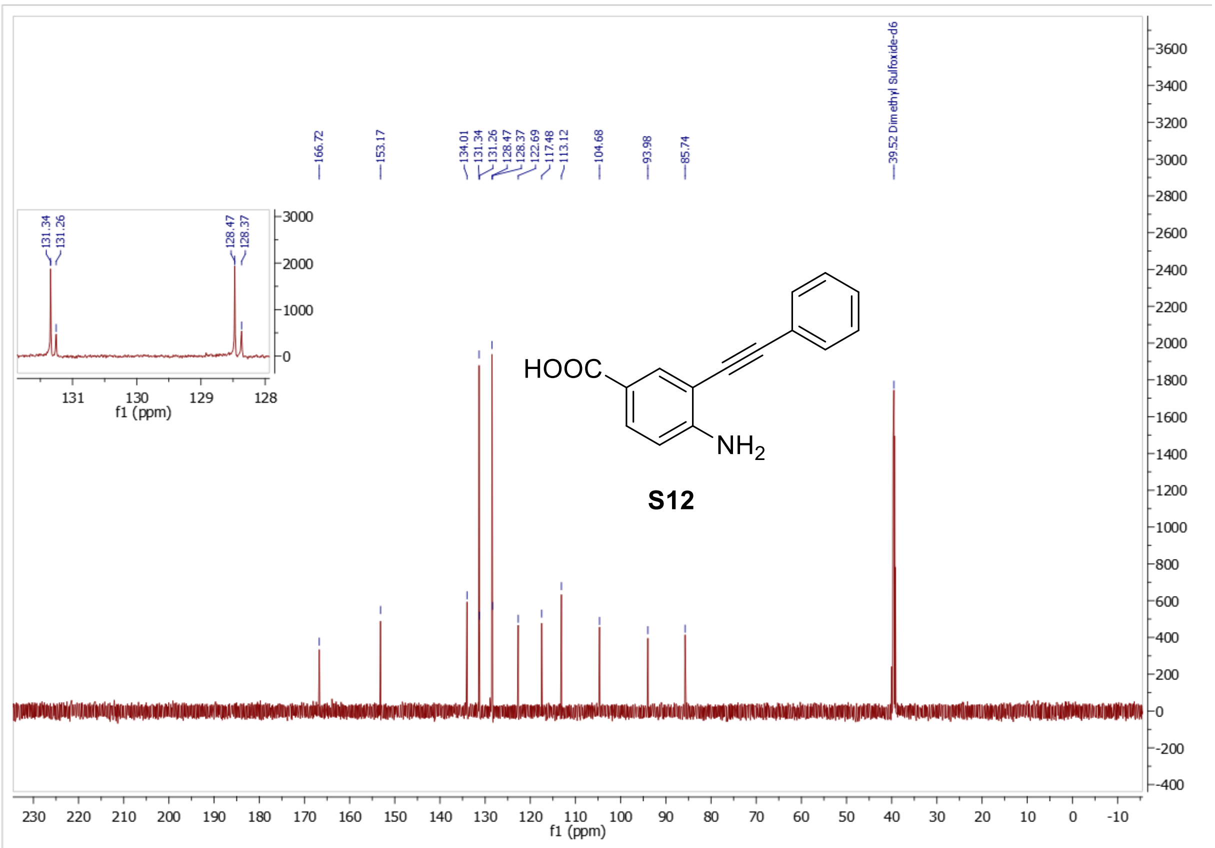

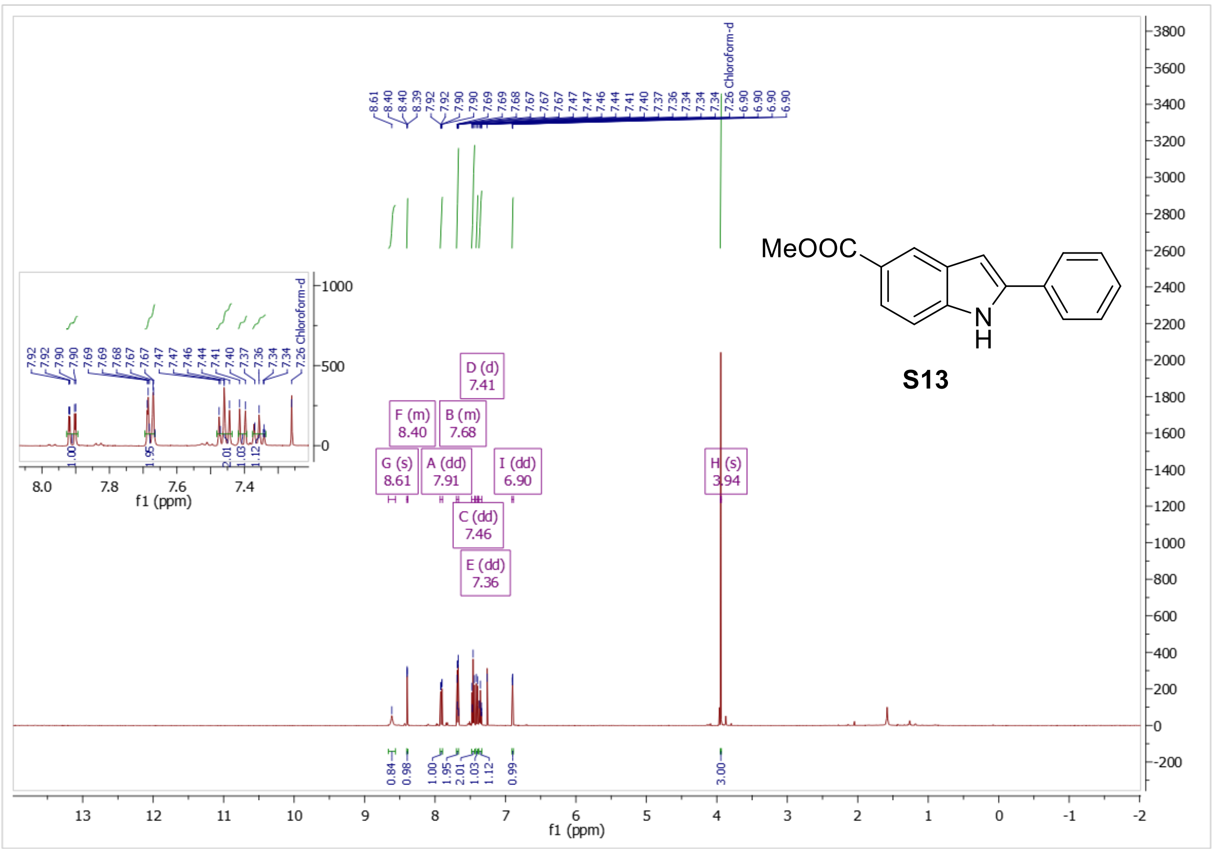
MeOOC
N
H
S13
G (s)
8.61
F (m)
8.40
A (dd)
7.91
B (m)
7.68
C (dd)
7.46
D (d)
7.41
E (dd)
7.36
I (dd)
6.90
H (s)
3.94
f1 (ppm)

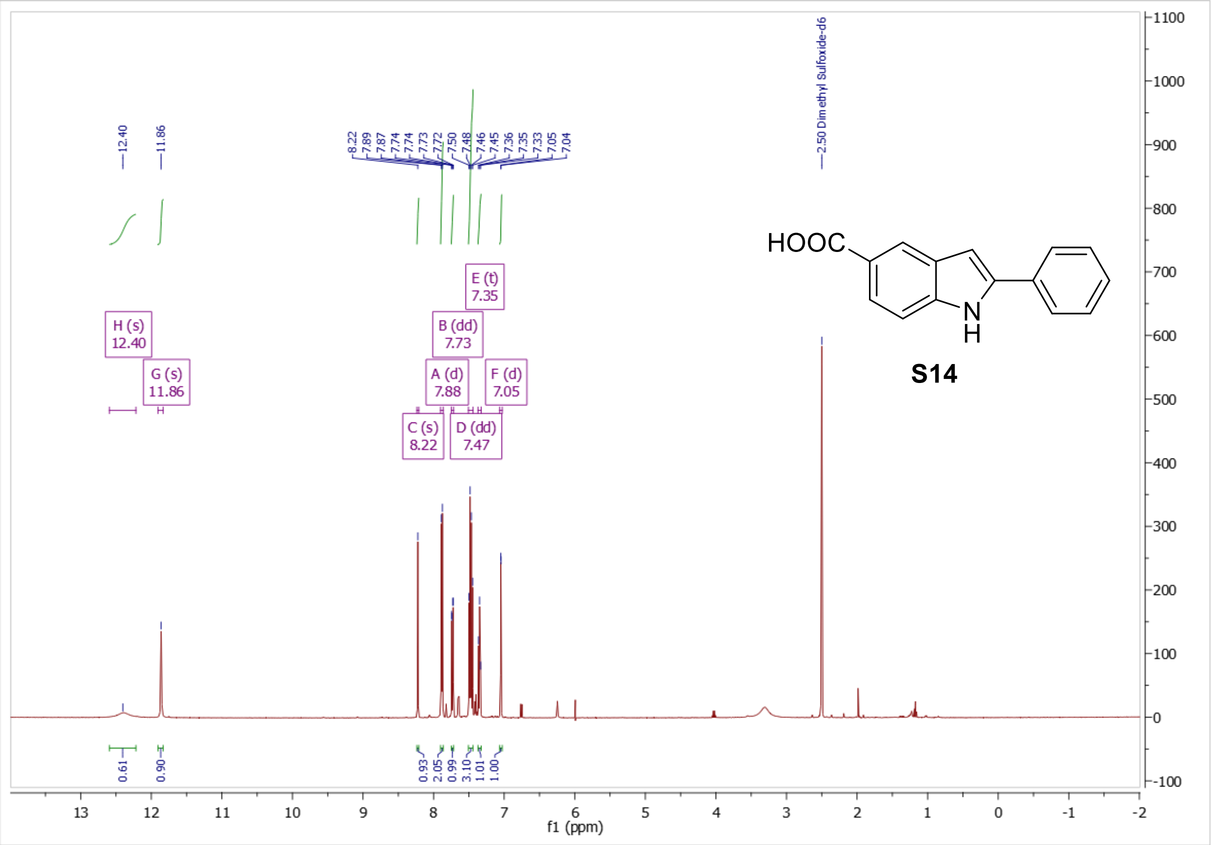

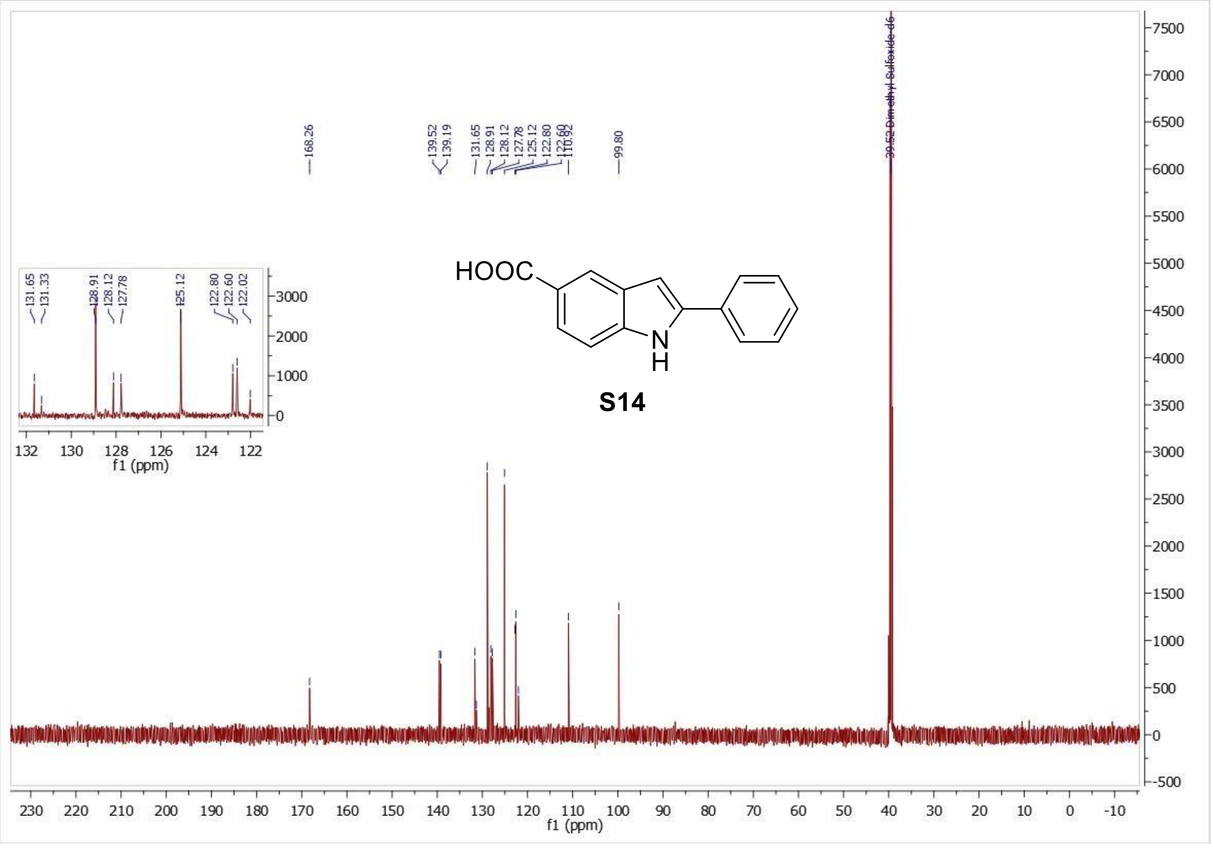

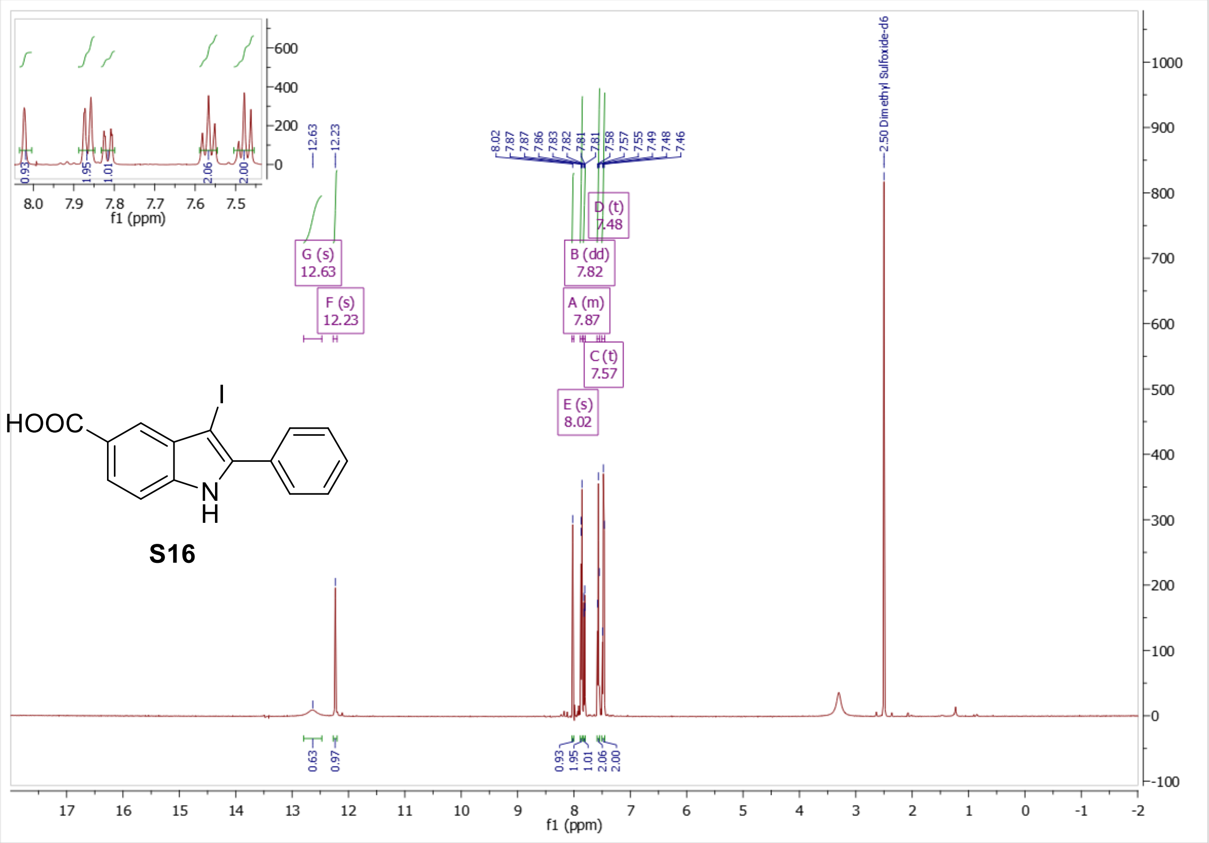
HOOC
I
N
H
S16
G (s) 12.63
F (s) 12.23
B (dd) 7.82
A (m) 7.87
C (t) 7.57
D (t) 7.48
E (s) 8.02
2.50 Dimethyl Sulfoxide-d6
f1 (ppm)

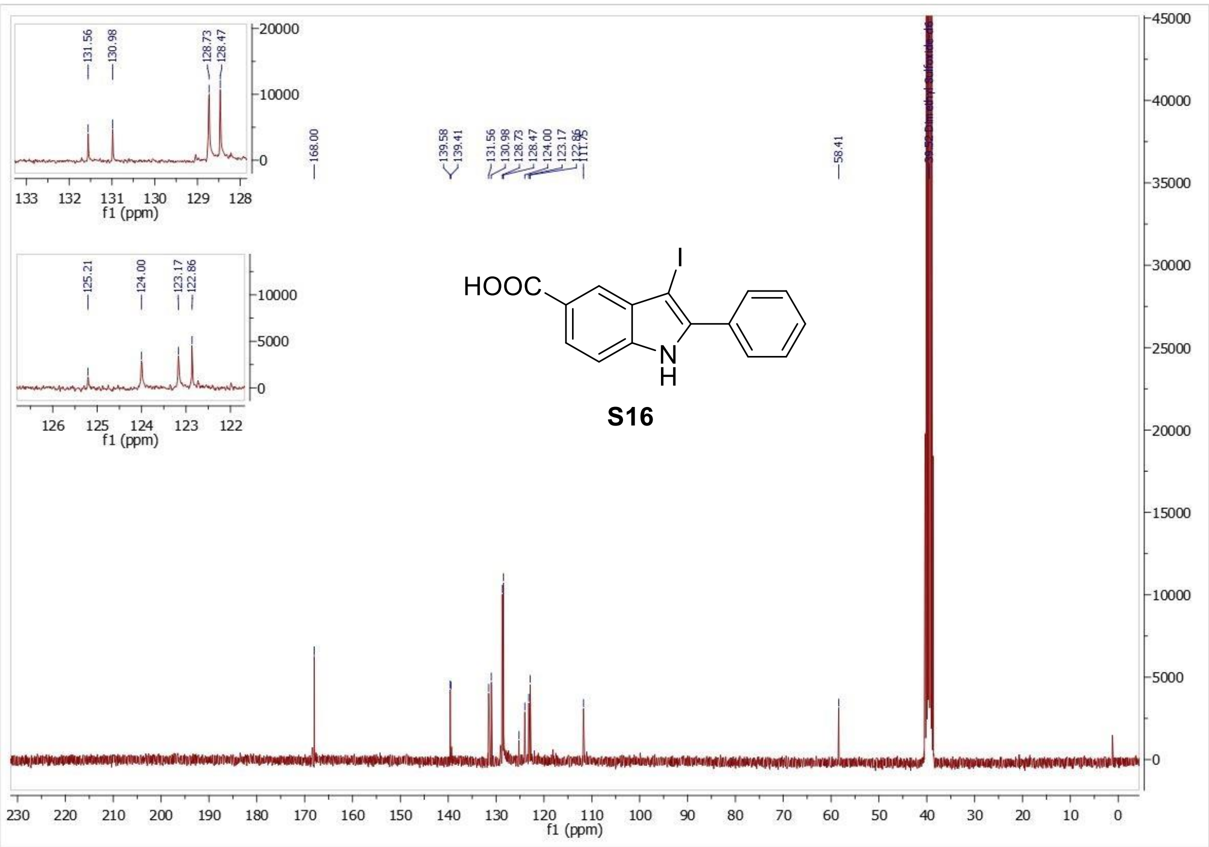
168.00
139.58
139.41
131.56
130.98
128.73
128.47
124.00
123.17
122.86
111.75
58.41
HOOC
I
N
H
S16
f1 (ppm)

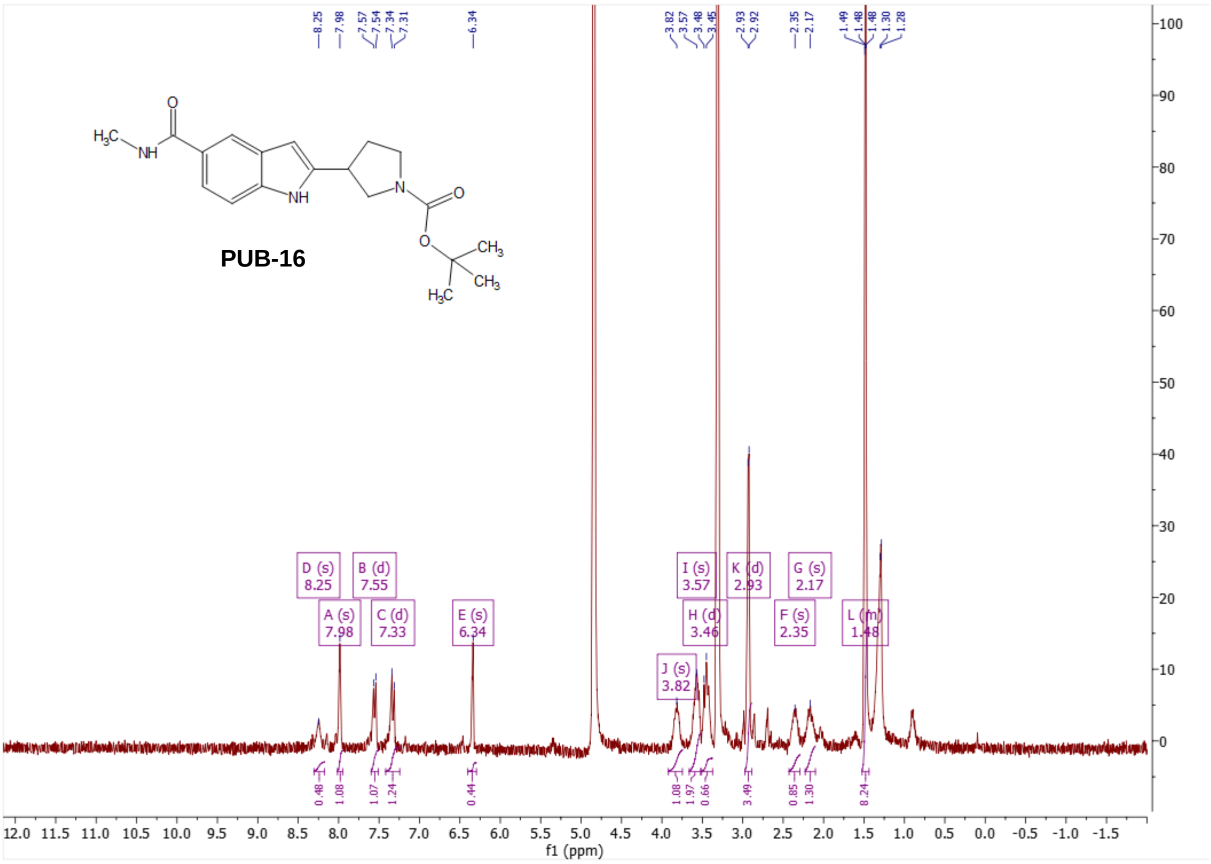
PUB-16
D (s) 8.25
B (d) 7.55
A (s) 7.98
C (d) 7.33
E (s) 6.34
I (s) 3.57
K (d) 2.93
G (s) 2.17
H (d) 3.46
F (s) 2.35
L (m) 1.48
J (s) 3.82
f1 (ppm)

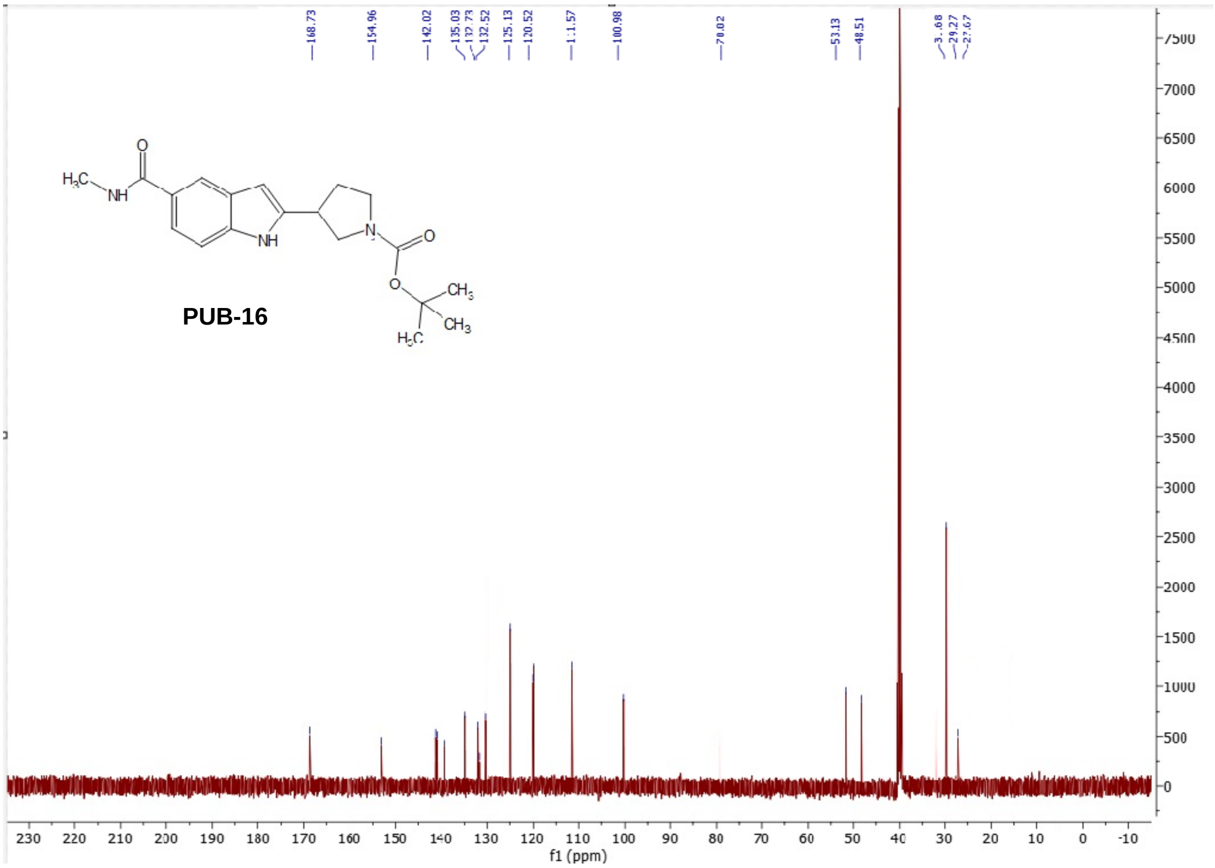
PUB-16
168.73
154.96
142.02
125.13
120.52
100.98
70.02
53.13
48.51
f1 (ppm)

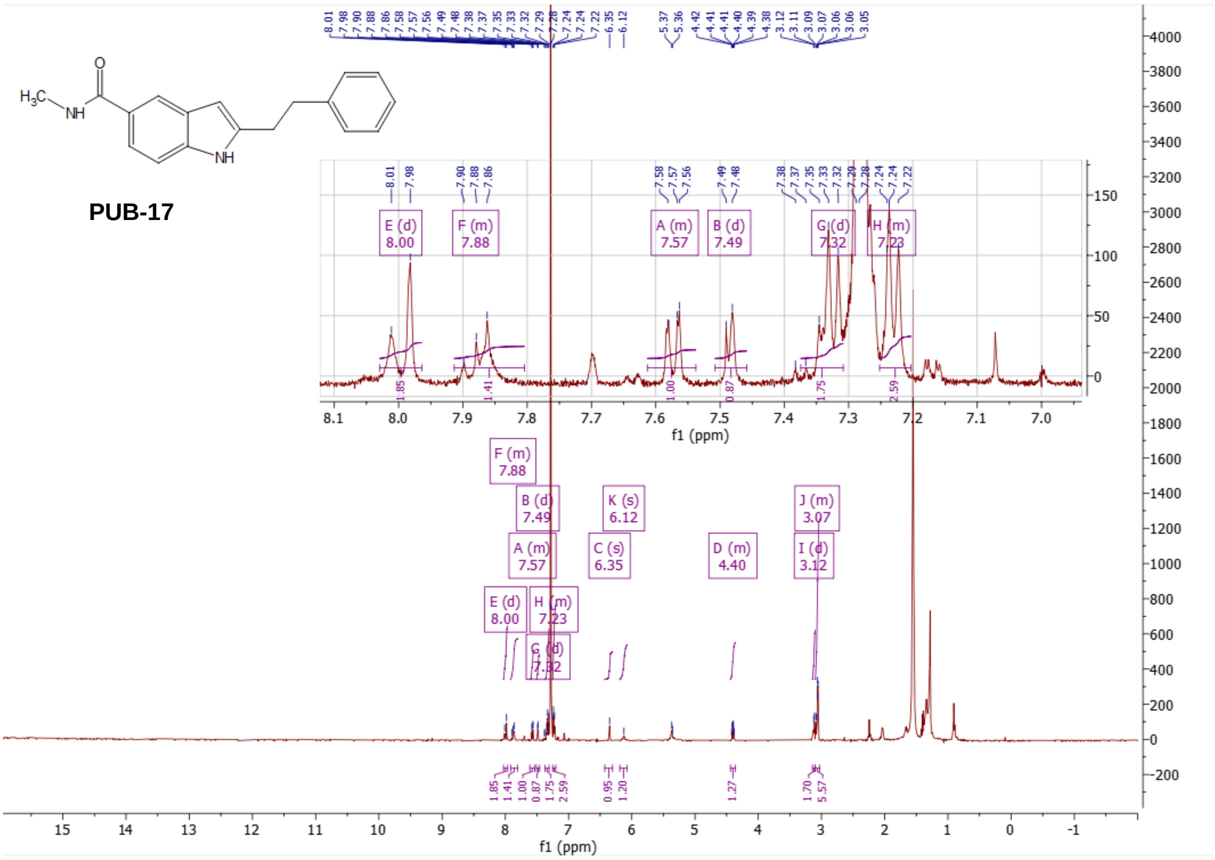
8.01 7.98 7.90 7.88 7.86 7.58 7.57 7.56 7.49 7.48 7.38 7.37 7.35 7.33 7.32 7.29 7.28 7.24 7.24 7.22 6.35 6.12
5.37 5.36 4.42 4.41 4.41 4.40 4.39 4.38 3.12 3.11 3.09 3.07 3.06 3.06 3.05
H3C NH O NH
PUB-17
E (d) 8.00
F (m) 7.88
A (m) 7.57
B (d) 7.49
G (d) 7.32
H (m) 7.23
K (s) 6.12
C (s) 6.35
D (m) 4.40
J (m) 3.07
I (d) 3.12
1.85 1.41 1.00 0.87 1.75 2.59 0.95 1.20 1.27 1.70 5.57
f1 (ppm)

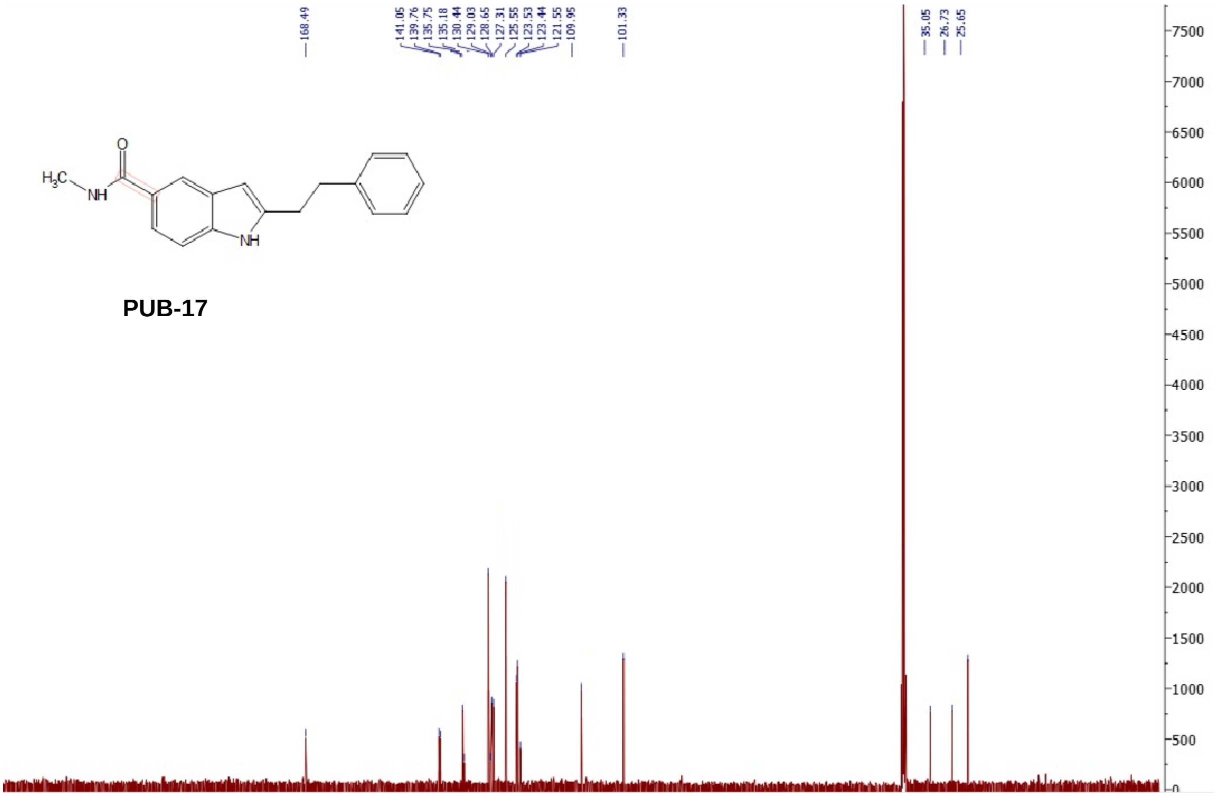
168.49
141.05 139.76 135.75 135.18 130.44 129.03 128.65 127.31 125.55 123.53 123.44 121.55 109.95
101.33
35.05 26.73 25.65
H3C NH O NH
PUB-17

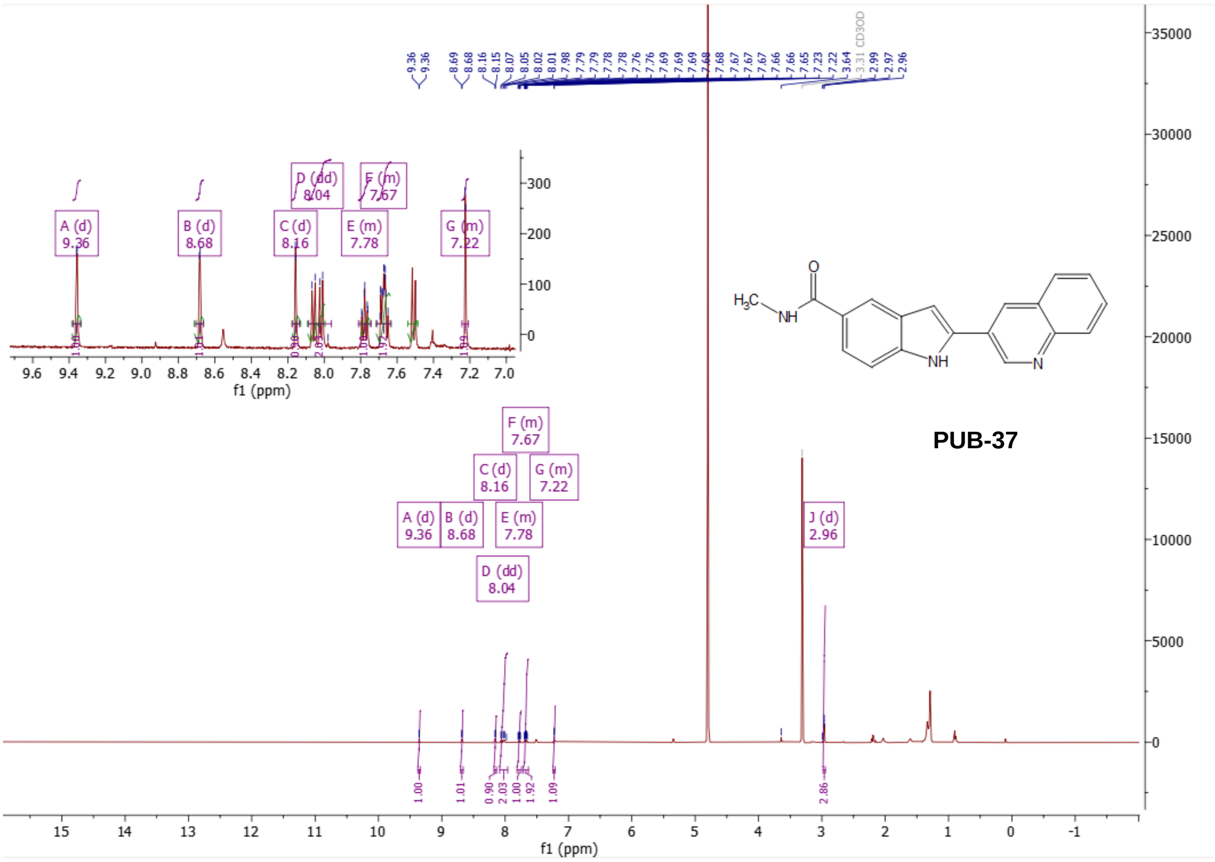
A (d)
9.36
B (d)
8.68
C (d)
8.16
D (dd)
8.04
E (m)
7.78
F (m)
7.67
G (m)
7.22
J (d)
2.96
CD3OD
f1 (ppm)
PUB-37

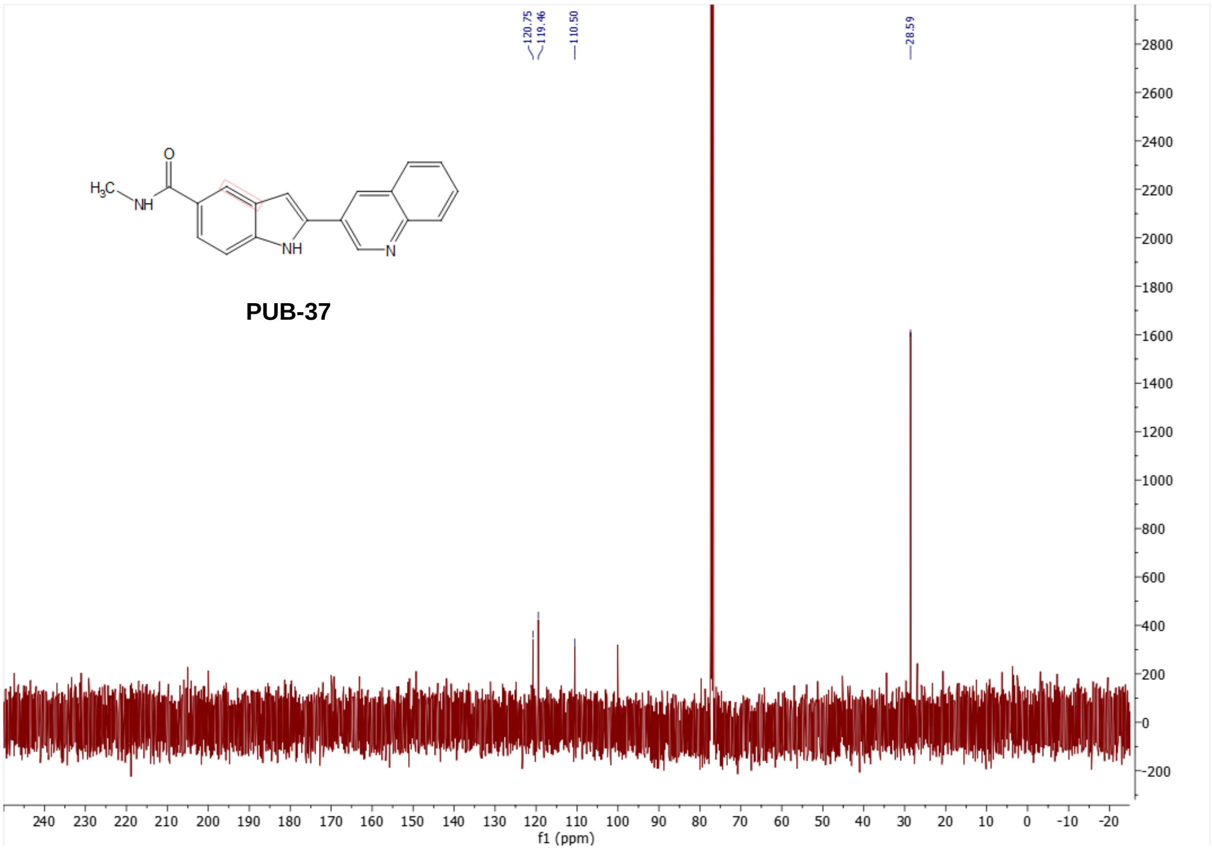
120.75
119.46
110.50
28.59
f1 (ppm)
PUB-37

11. References

[1] Cantagrel, G.; de Carné-Carnavalet, B.; Meyer, C.; Cossy J. Iron Trichloride-Promoted Cyclization of *o*-Alkynylaryl Isocyanates: Synthesis of 3-(Chloromethylene)oxindoles. *Org. Lett.* **2009**, *11*, 19, 4262–4265.

[2] Zeng, C.; Fang, S.; Guo, S.; Jiang, H.; Yang, S.; Wu, W. Palladium-Catalyzed Tandem Nucleophilic Addition/C–H Functionalization of Anilines and Bromoalkynes for the Synthesis of 2-Phenylindoles. *Org. Lett.* **2023**, *25*, 9, 1409–1414.

[3] Arun, V.; Pilania, M.; Kumar, D. Access to 2-Arylindoles via Decarboxylative C−C Coupling in Aqueous Medium and to Heteroaryl Carboxylates under Base-Free Conditions using Diaryliodonium Salts. *Chem. Asian J.* **2016**, *11*, 23, 3345–3349.

[4] Wu, Z.; Graybill, T. L.; Zeng, X.; Platchek, M.; Zhang, J.; Bodmer, V. Q.; Wisnoski, D. D.; Deng, J.; Coppo, F.T.; Yao, G.; Tamburino, A.; Scavello, G.; Franklin, G. J.; Mataruse, S.; Bedard, K. L.; Ding, Y.; Chai, J.; Summerfield, J.; Centrella, P. A.; Messer, J.A.; Pope, A. J.; Israel, D. I.; Cell-Based Selection Expands the Utility of DNA-Encoded Small-Molecule Library Technology to Cell Surface Drug Targets: Identification of Novel Antagonists of the NK3 Tachykinin Receptor. *ACS Comb. Sci.* **2015**, *17*, 12, 722−731.

[5] Bradford, M. M. A Rapid and Sensitive Method for the Quantitation of Microgram Quantities of Protein Utilizing the Principle of Protein-Dye Binding. *Anal. Biochem.* **1976**, *72*, 248–54.

[6] Levesque, D.; Diaz, J.; Pilon, C.; Martes, M.-P.; Giros, B.; Souil, E.; Schott, D.; Morgat, J.-L.; Schwartz, J.-C.; Sokoloff, P. Identification, Characterization, and Localization of the Dopamine D3 Receptor in Rat Brain Using 7-[3H]hydroxy-N,N-di-*n*-Propyl-2-Aminotetralin. *Proc. Natl. Acad. Sci. USA*. **1992**, 8155-8159.

[7] Cheng, Y.-C.; Prusoff, W. H. Relationship Between the Inhibition Constant (K_I) and the Concentration of Inhibitor Which Causes 50 percent Inhibition (I_{50}) of an Enzymatic Reaction. *Biochem. Pharmacol.* **1973**, *22*, 23, 3099-3108.

[8] Wang, H.; Zhao, G.; Zhang, T.; Li, Y.; Zhang, G.; Li, Y. Comparative Study of DNA Barcode Integrity Evaluation Approaches in the Early-Stage Development of DNA-Compatible Chemical Transformation. *ACS Pharmacol. Transl. Sci.* **2023**, *6*, 1724−1733.
